# Supplementary material for: TDP-43 Controls HIV-1 Viral Production and Virus Infectiveness
Source: Int J Mol Sci. 2023 Apr 21;24(8):7658. doi: 10.3390/ijms24087658 (PMC10142003; doi:10.3390/ijms24087658)

Figure S1. Western-blot replicates associated with Figure 1A

Cabrera-Rodríguez R., *et al.*

Replicate 1 as figure format

A

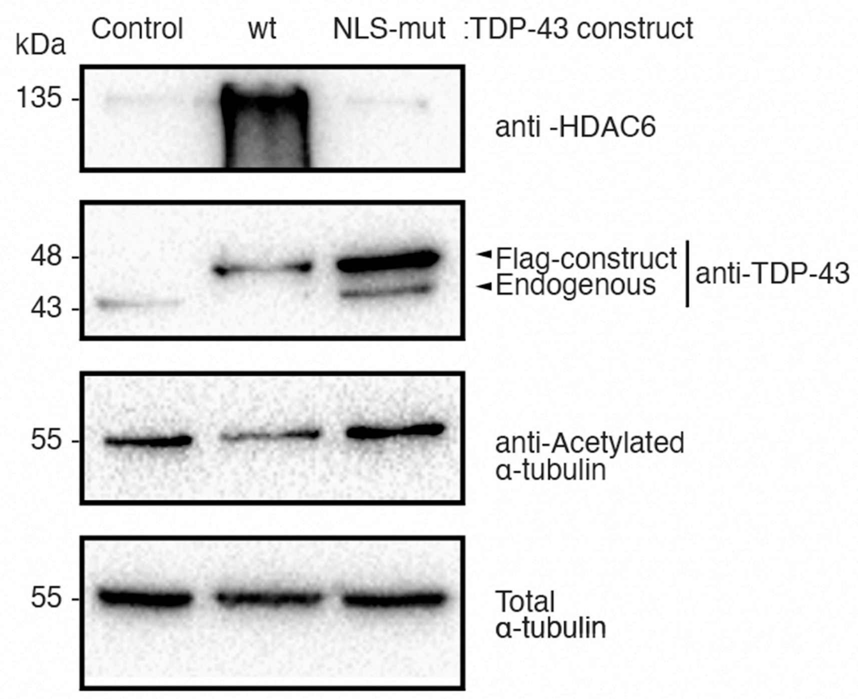

Replicate 2 as figure format

A

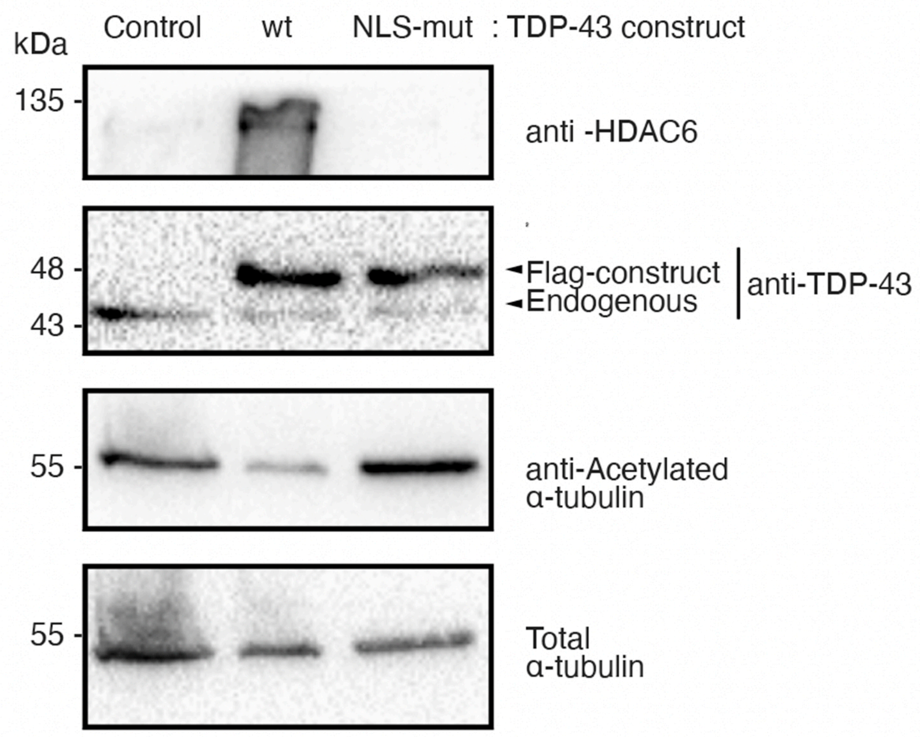

Replicate 3 as figure format

A

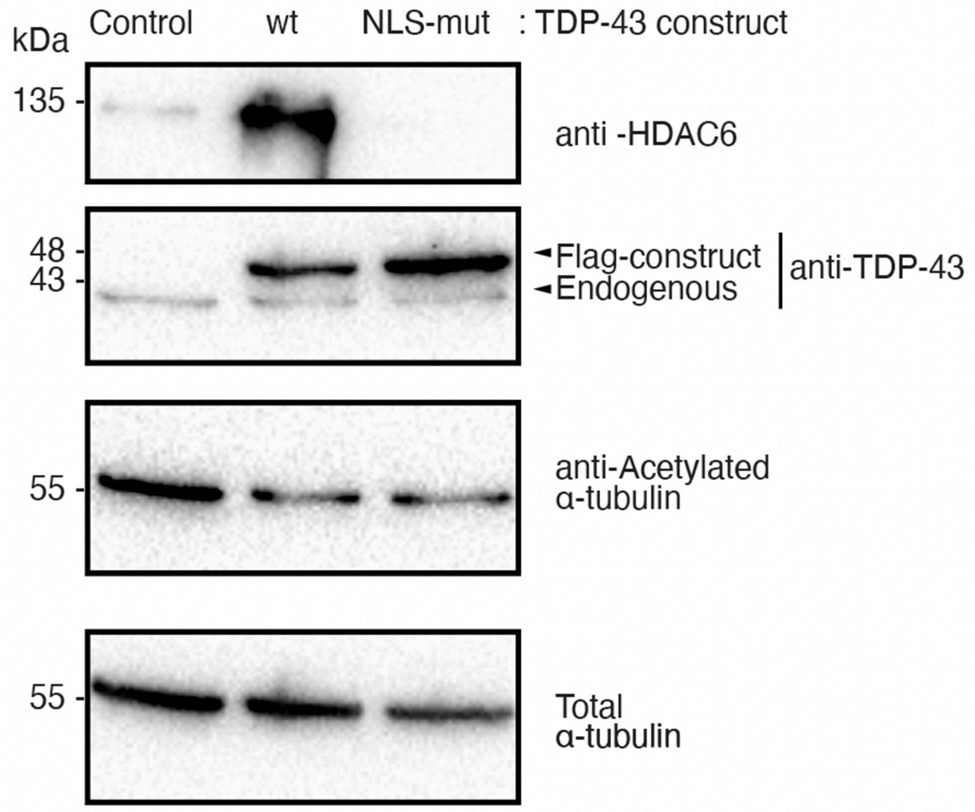

Figure S1. Replicate 1 HDAC6 complete gel   Western-blot associated with Figure 1A
Cabrera-Rodríguez, R., *et al.*

Control   wt   NLS-mut   : TDP-43 construct

135kDa-

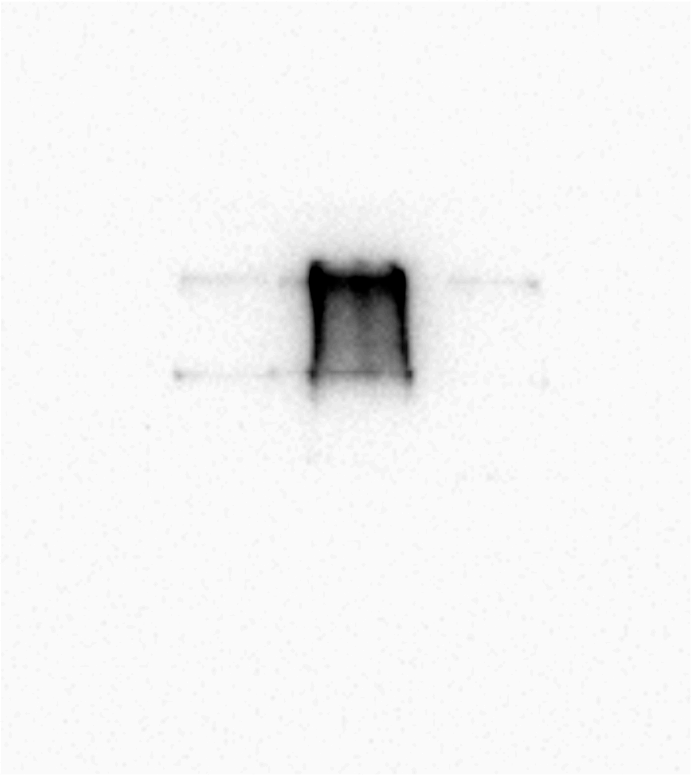

► Specific HDAC6 band

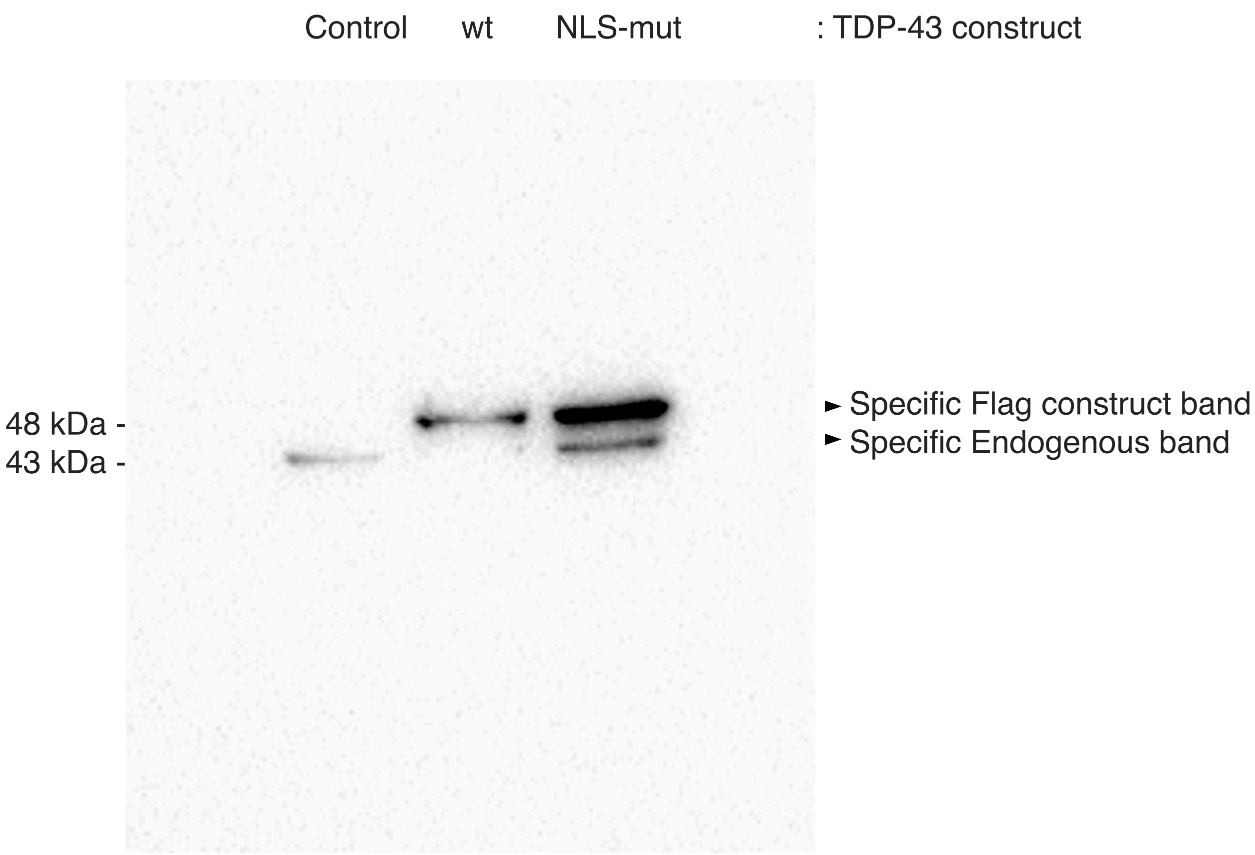
Control
wt
NLS-mut
: TDP-43 construct
48 kDa -
43 kDa -
► Specific Flag construct band
► Specific Endogenous band

Figure S1. Replicate 1 Acetylated α-tubulin complete gel   Western-blot associated with Figure 1A

Cabrera-Rodríguez, R., *et al.*

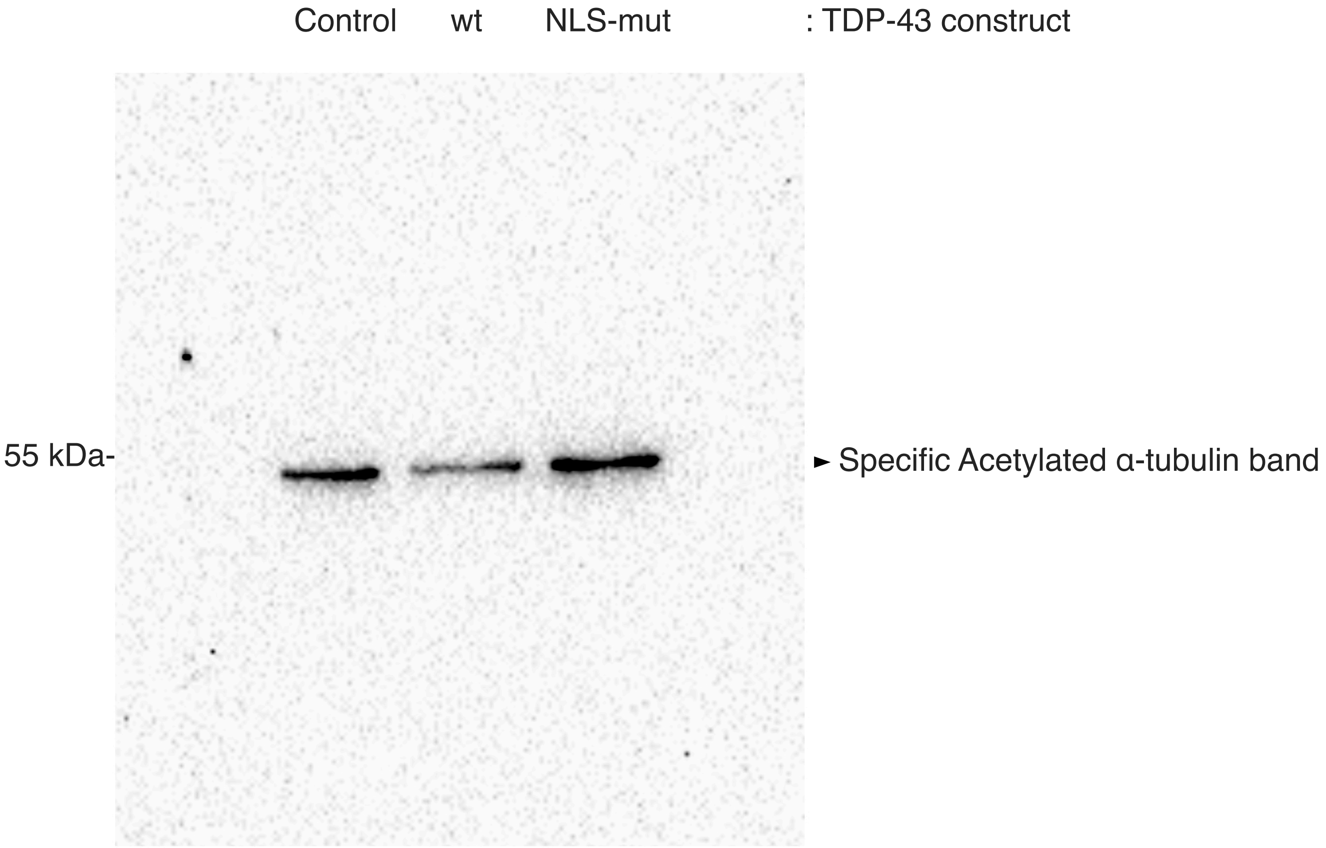

Figure S1. Replicate 1 Total α-tubulin complete gel Western-blot associated with Figure 1A

Cabrera-Rodríguez, R., *et al.*

Control wt NLS-mut : TDP-43 construct

55 kDa-

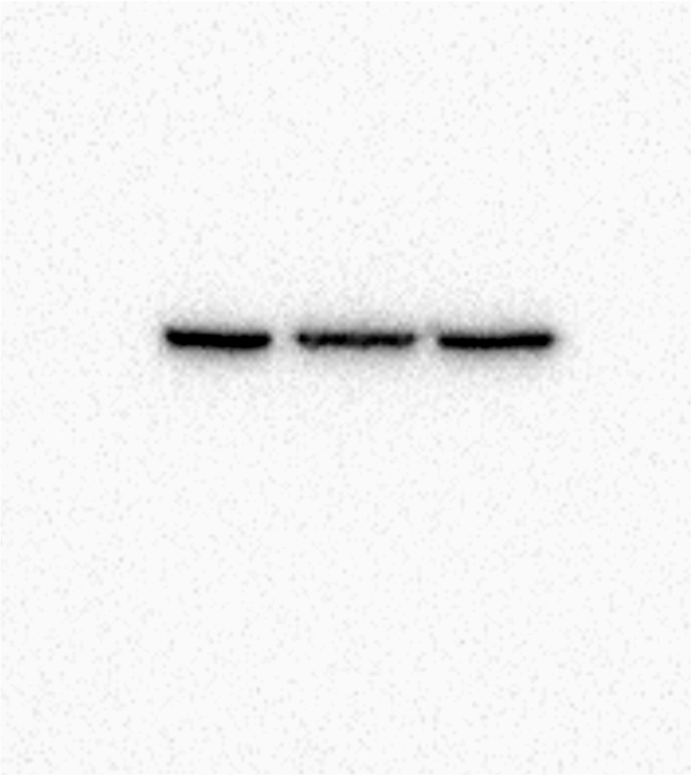

► Specific Total α-tubulin band

Figure S1. Replicate 2 HDAC6 complete gel Western-blot associated with Figure 1A
Cabrera-Rodríguez, R., *et al.*

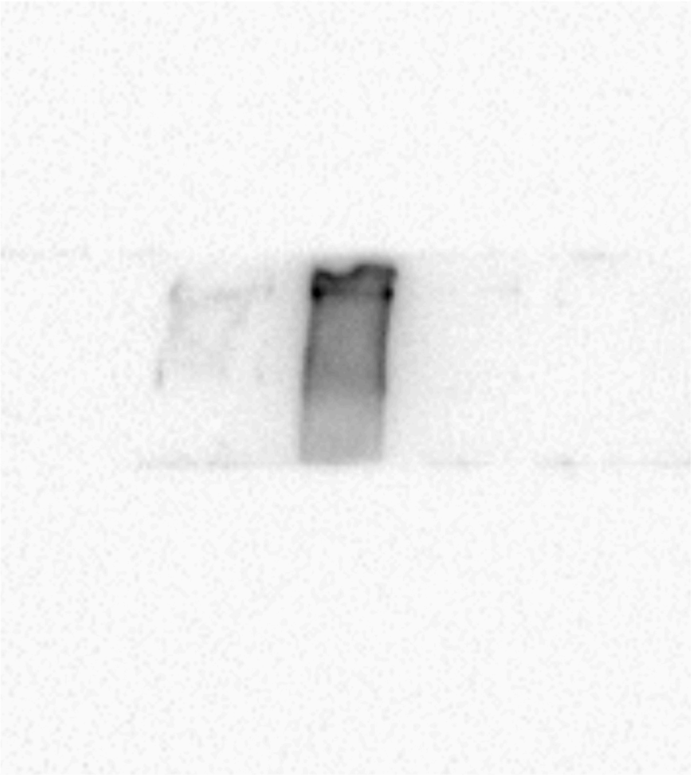

Figure S1. Replicate 2 TDP-43 complete gel Western-blot associated with Figure 1A
Cabrera-Rodríguez, R., *et al.*

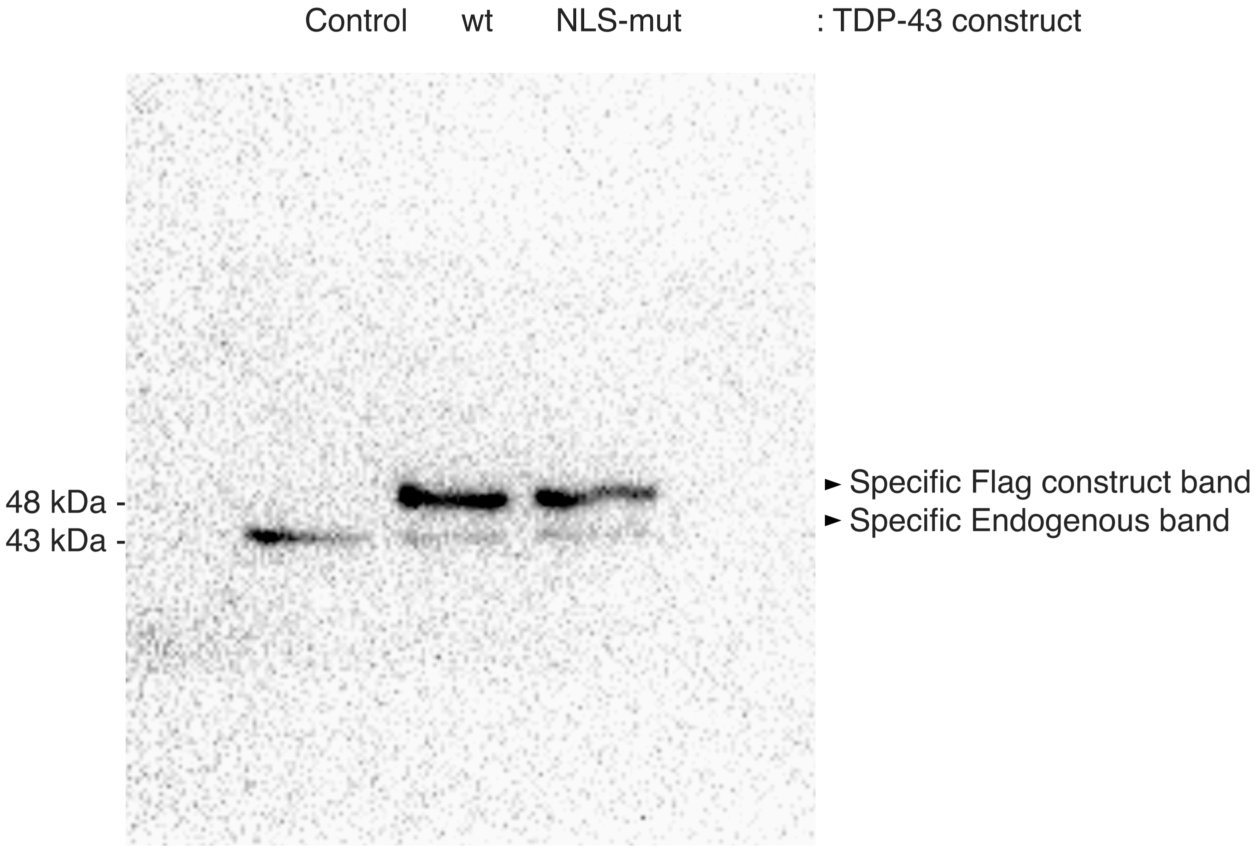

Figure S1. Replicate 2 Acetylated α-tubulin complete gel Western-blot associated with Figure 1A

Cabrera-Rodríguez, R., *et al.*

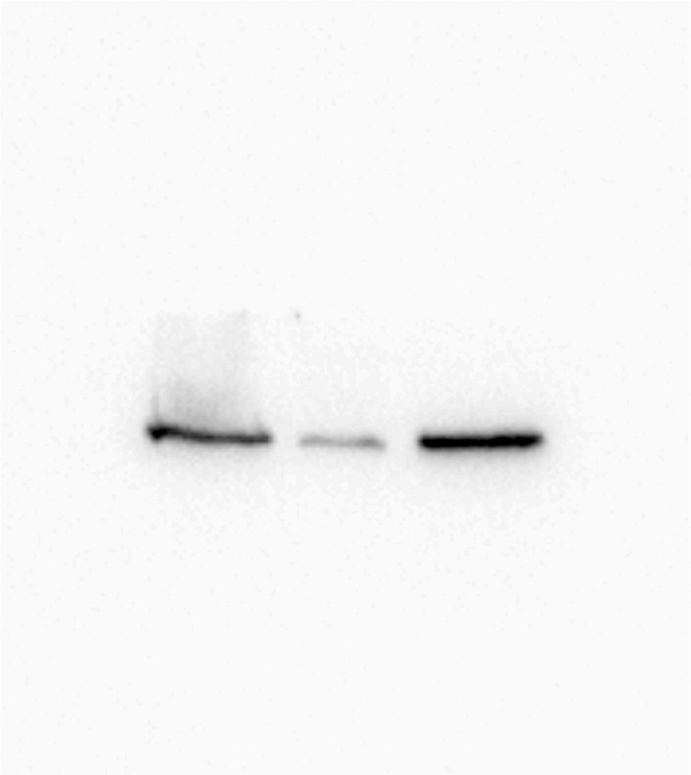

Figure S1. Replicate 2 Total α-tubulin complete gel   Western-blot associated with Figure 1A
Cabrera-Rodríguez, R., *et al.*

Control wt NLS-mut : TDP-43 construct

55 kDa-

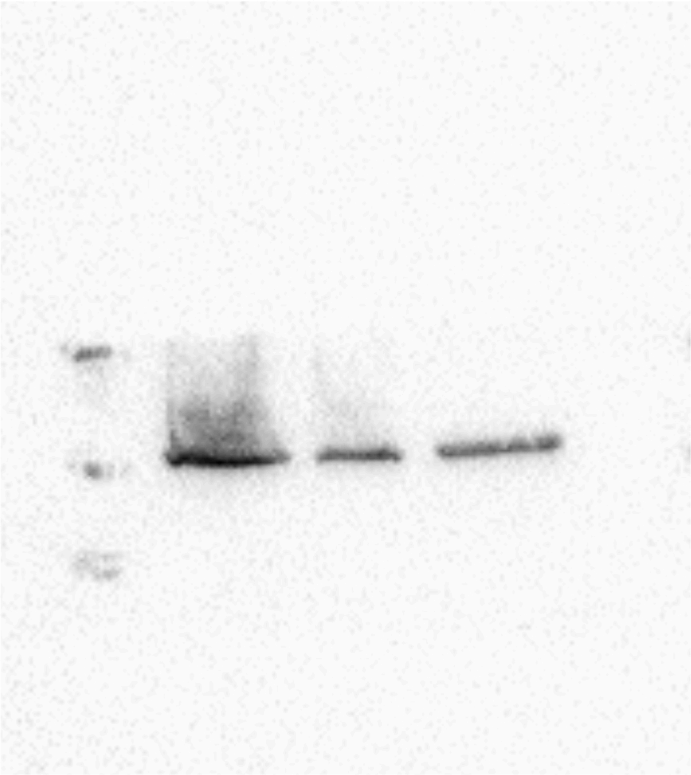

► Specific Total α-tubulin band

Figure S1. Replicate 3 HDAC6 complete gel Western-blot associated with Figure 1A
Cabrera-Rodríguez, R., *et al.*

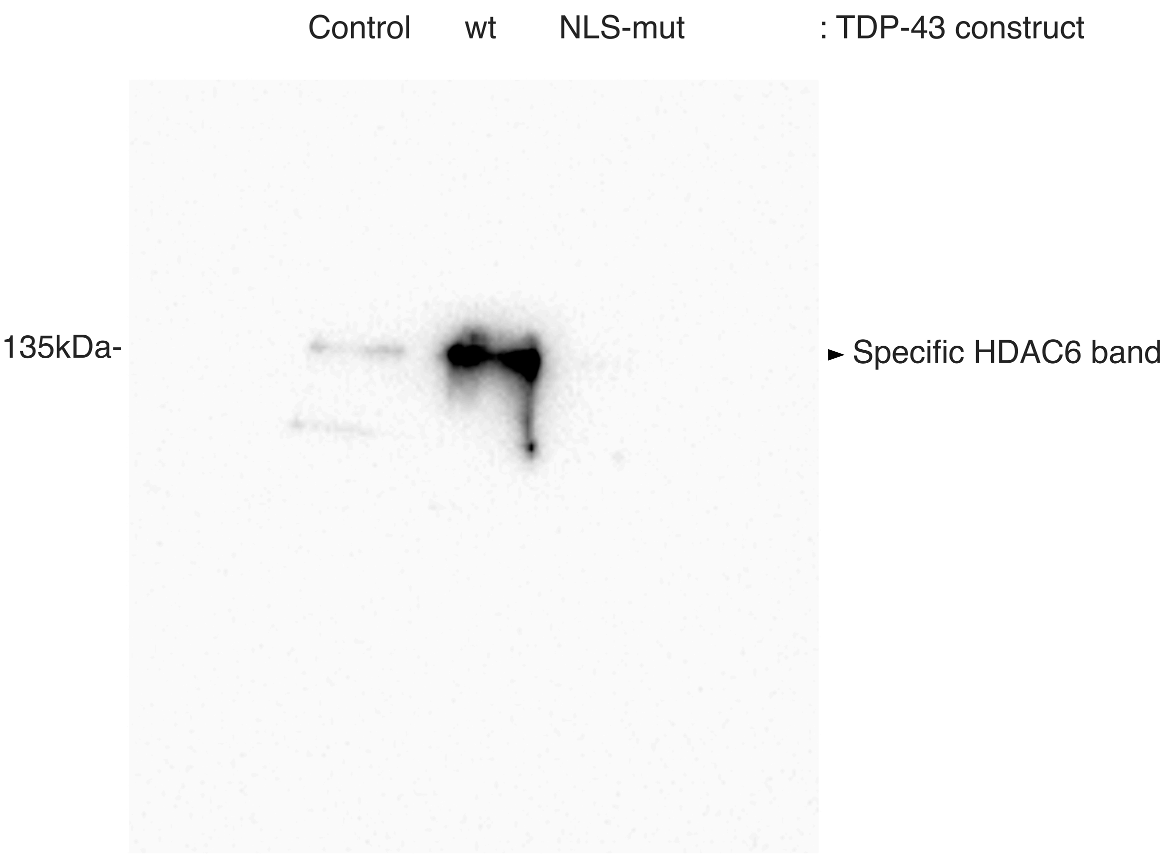

Figure S1. Replicate 3 TDP-43 complete gel Western-blot associated with Figure 1A

Cabrera-Rodríguez, R., *et al.*

Control wt NLS-mut : TDP-43 construct

48 kDa -
43 kDa -

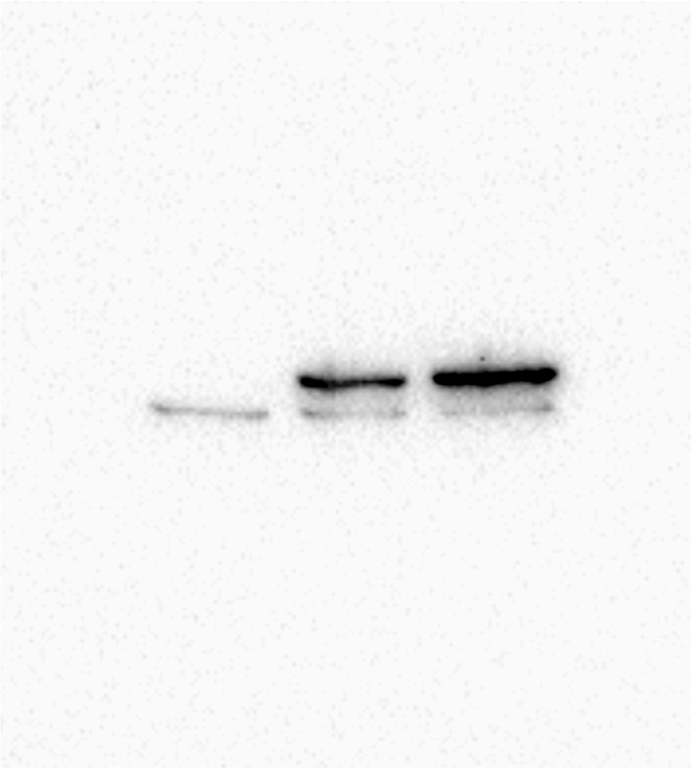

► Specific Flag construct band
► Specific Endogenous band

Figure S1. Replicate 3 Acetylated α-tubulin complete gel   Western-blot associated with Figure 1A

Cabrera-Rodríguez, R., *et al.*

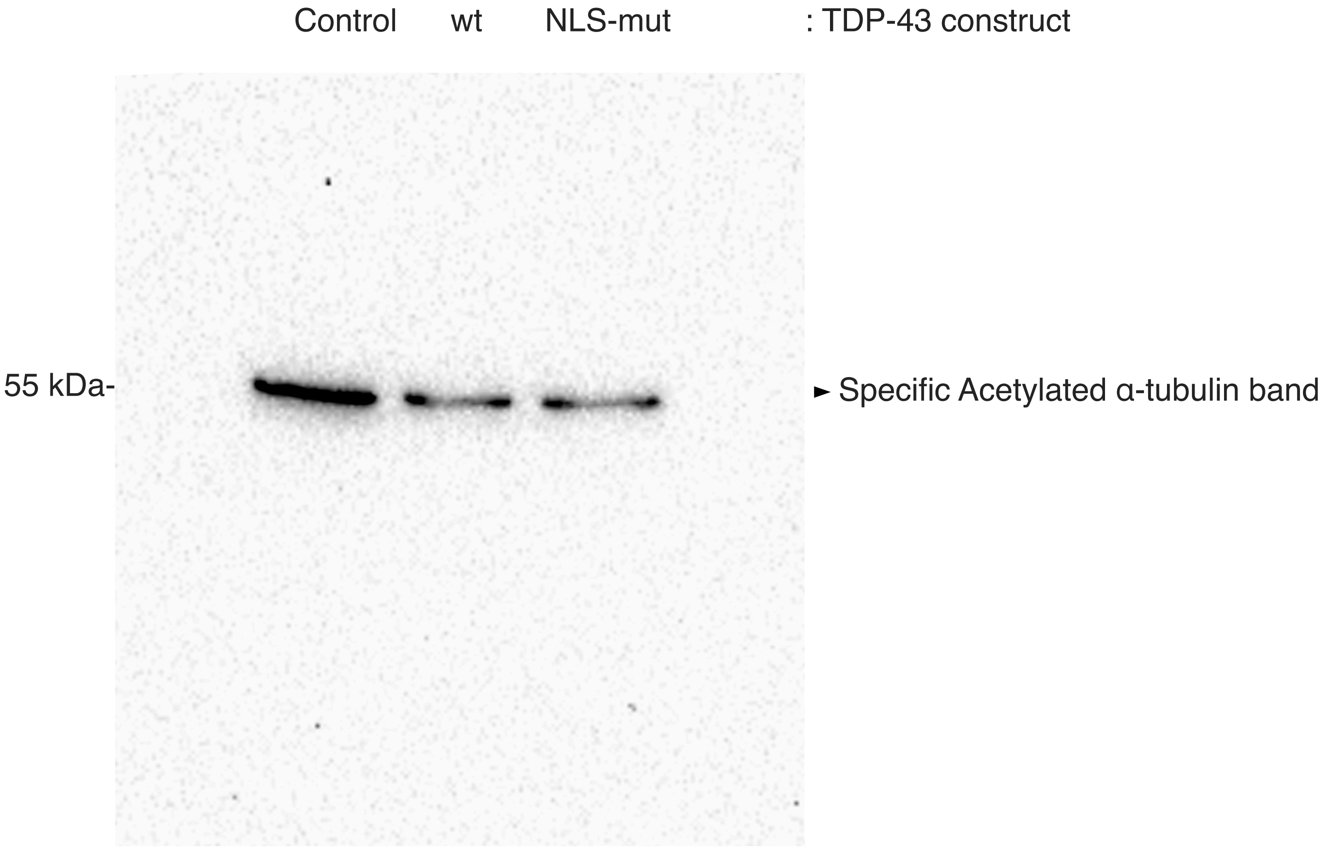

Figure S1. Replicate 3 Total α-tubulin complete gel   Western-blot associated with Figure 1A
Cabrera-Rodríguez, R., *et al.*

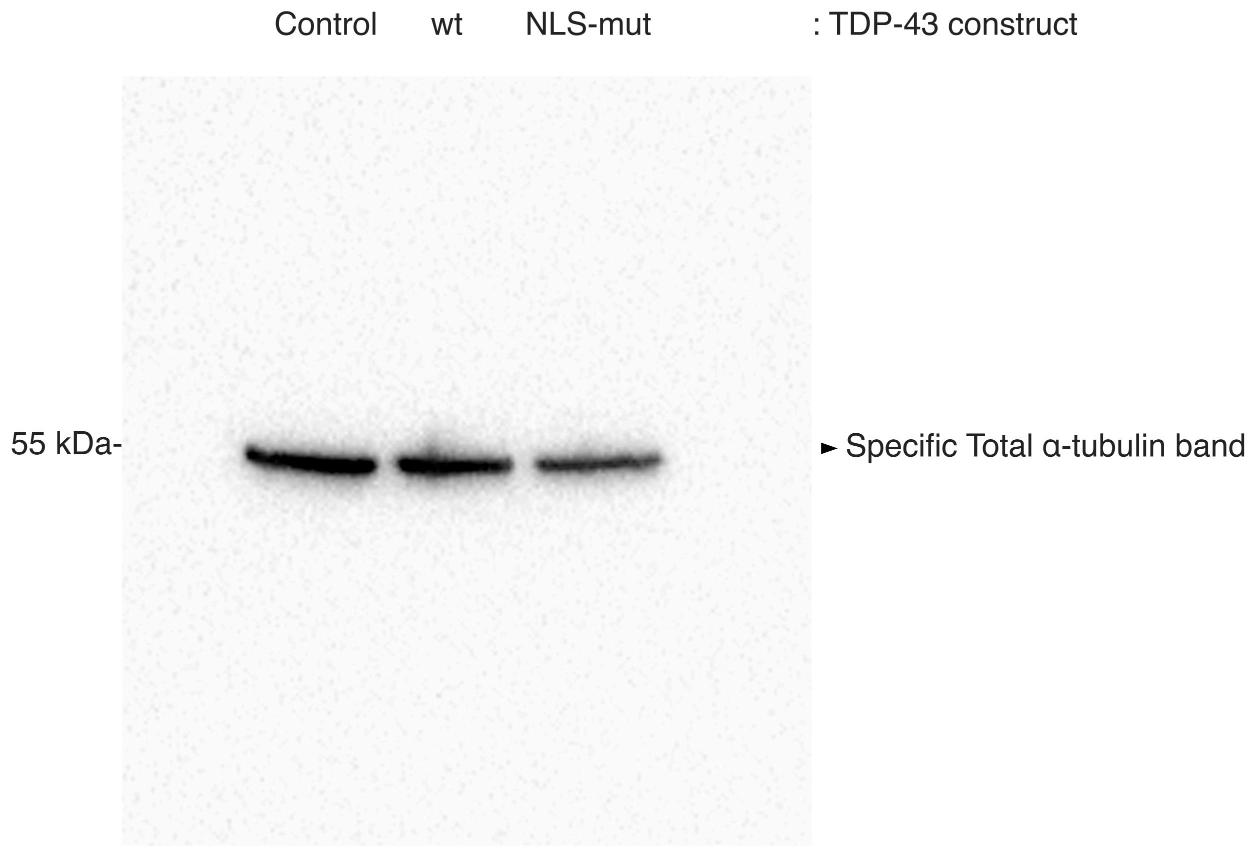

Figure S2A. Western-blot replicates associated with Figure 2A Cabrera-Rodríguez R., *et al.*

Replicate 1 as figure format

A

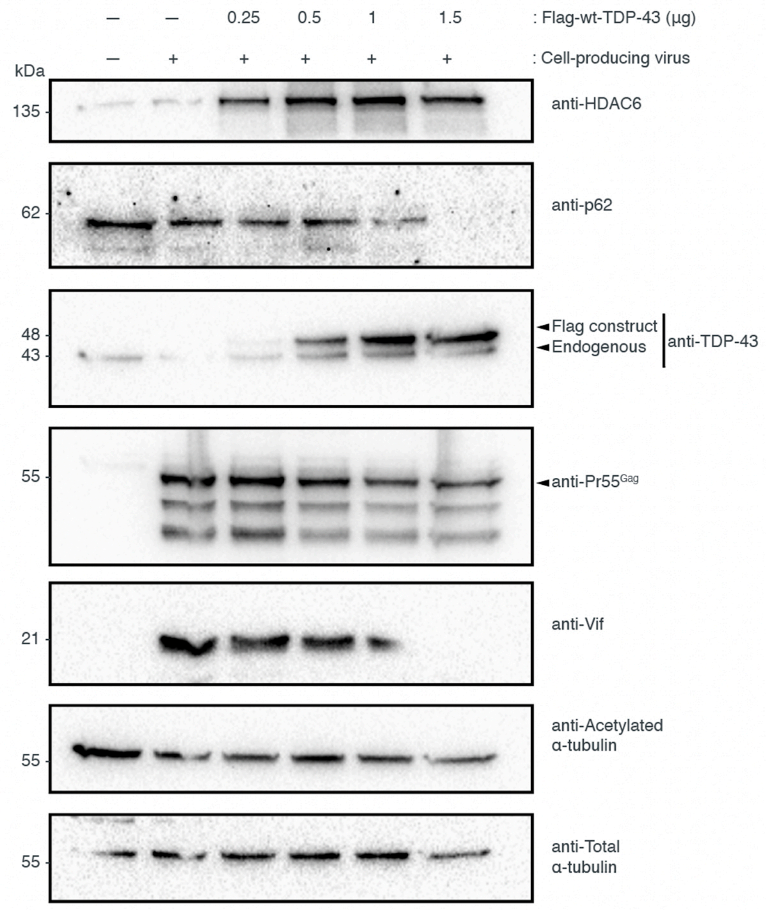

Replicate 2 as figure format

A

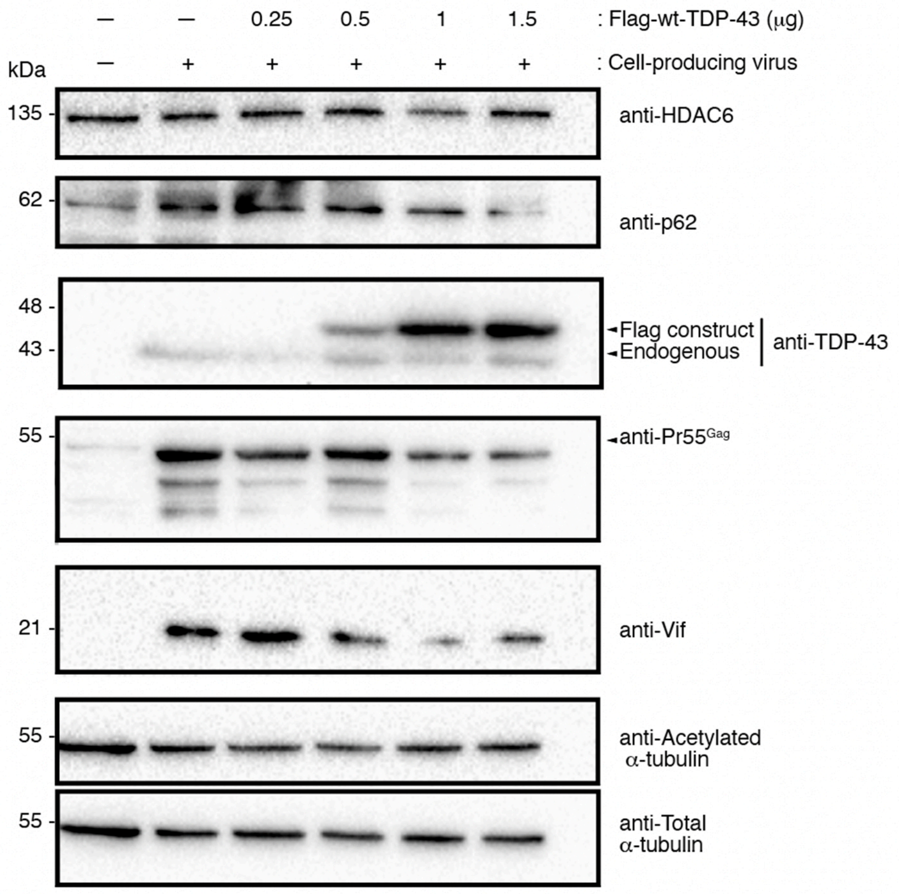

Replicate 3 as figure format

A

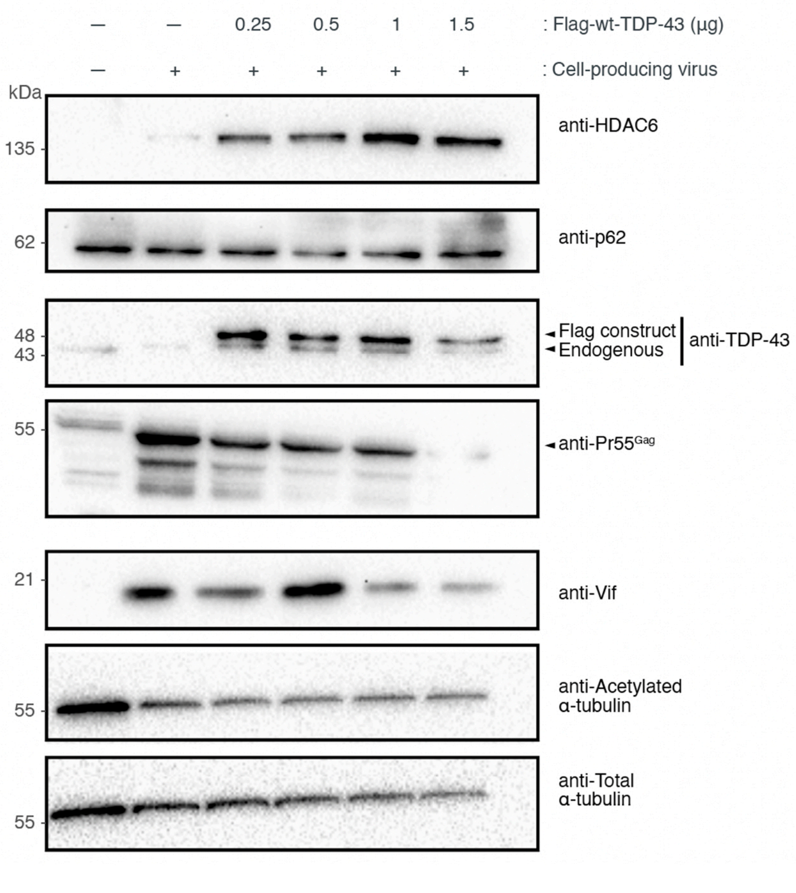

Figure S2A. Replicate 1 HDAC6 complete gel   Western-blot associated with Figure 2A
Cabrera-Rodríguez, R., *et al.*

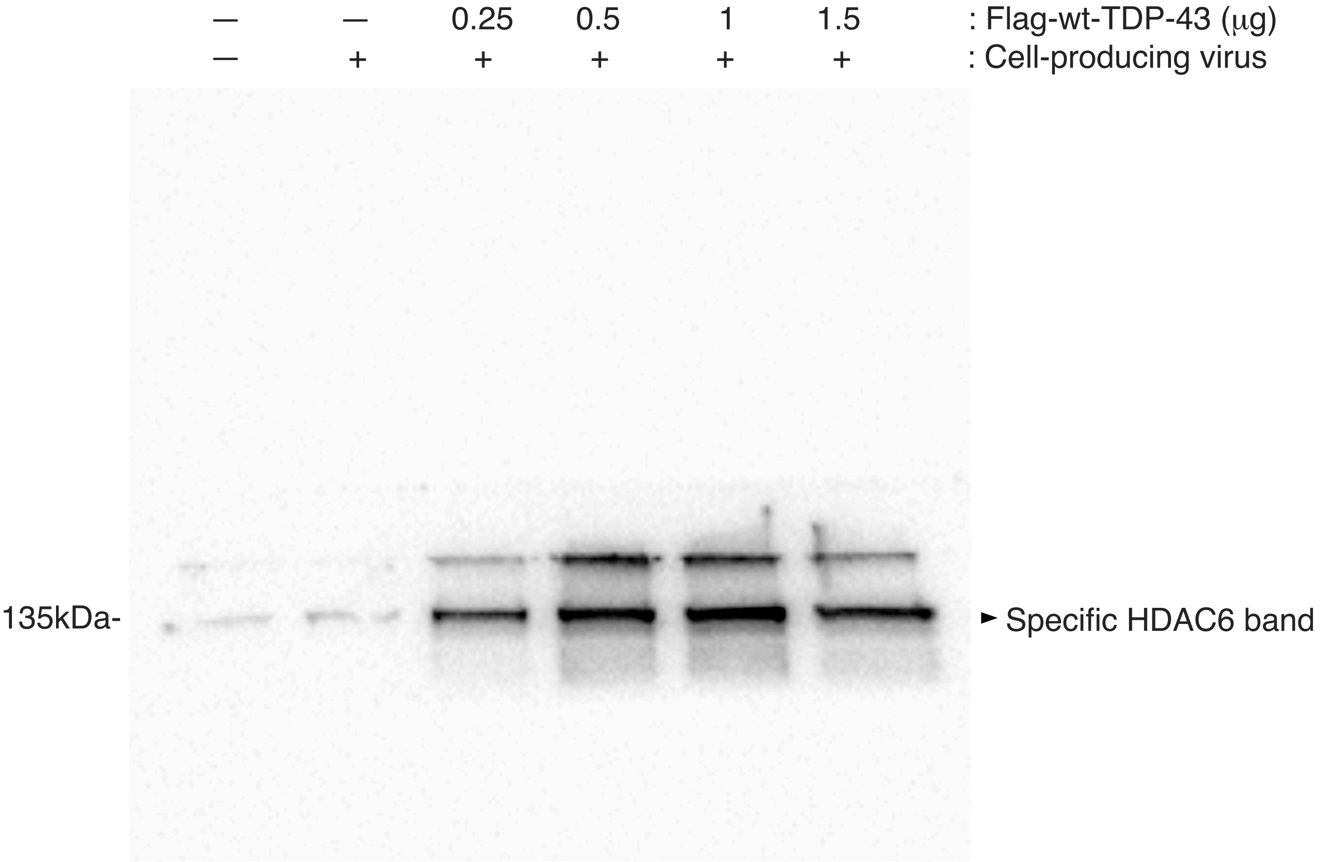

Figure S2A. Replicate 1 p62 complete gel   Western-blot associated with Figure 2A
Cabrera-Rodríguez, R., *et al.*

—	—	0.25	0.5	1	1.5	: Flag-wt-TDP-43 (μg)
—	+	+	+	+	+	: Cell-producing virus

62 kDa -

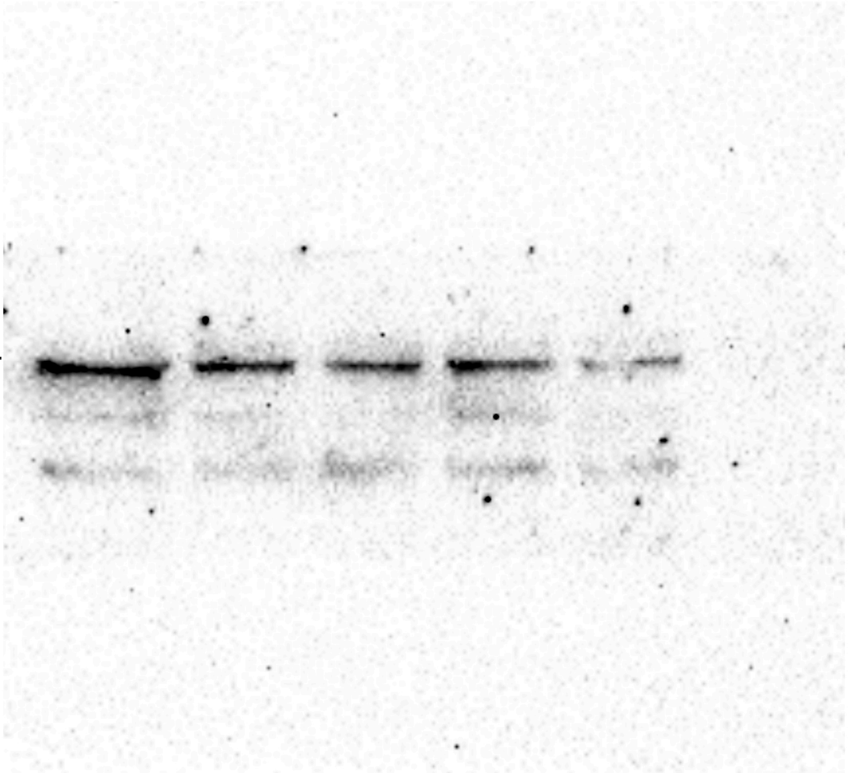

► Specific p62 band

Figure S2A. Replicate 1 TDP-43 complete gel   Western-blot associated with Figure 2A
Cabrera-Rodríguez, R., *et al.*

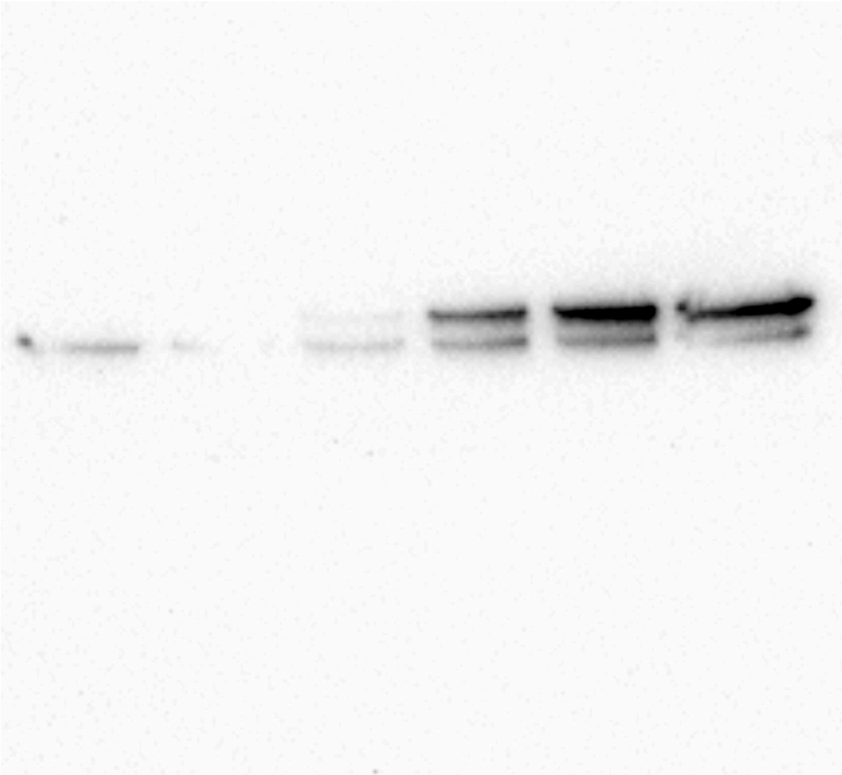

Figure S2A. Replicate 1 $Pr55^{Gag}$ complete gel Western-blot associated with Figure 2A

Cabrera-Rodríguez, R., *et al.*

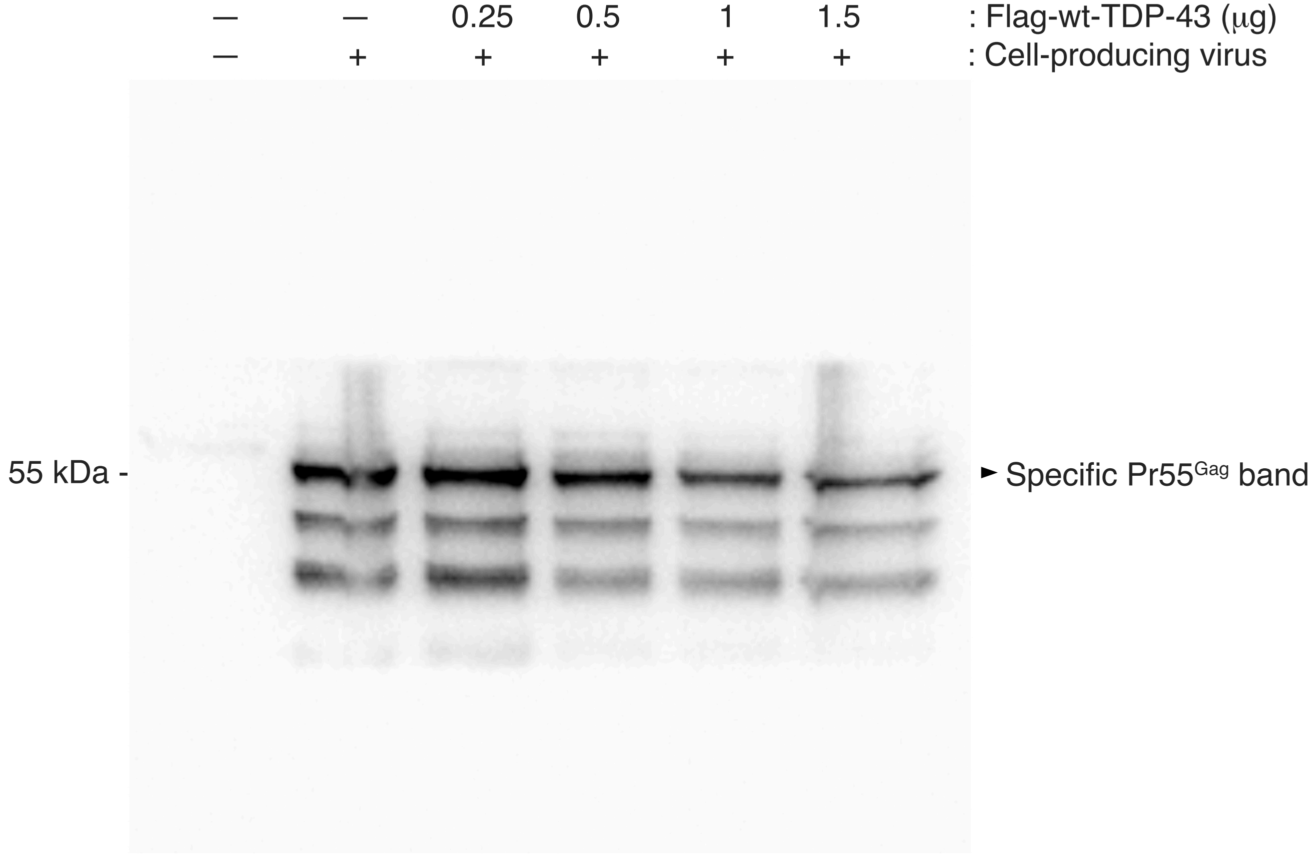

Figure S2A. Replicate 1 Vif complete gel   Western-blot associated with Figure 2A
Cabrera-Rodríguez, R., *et al.*

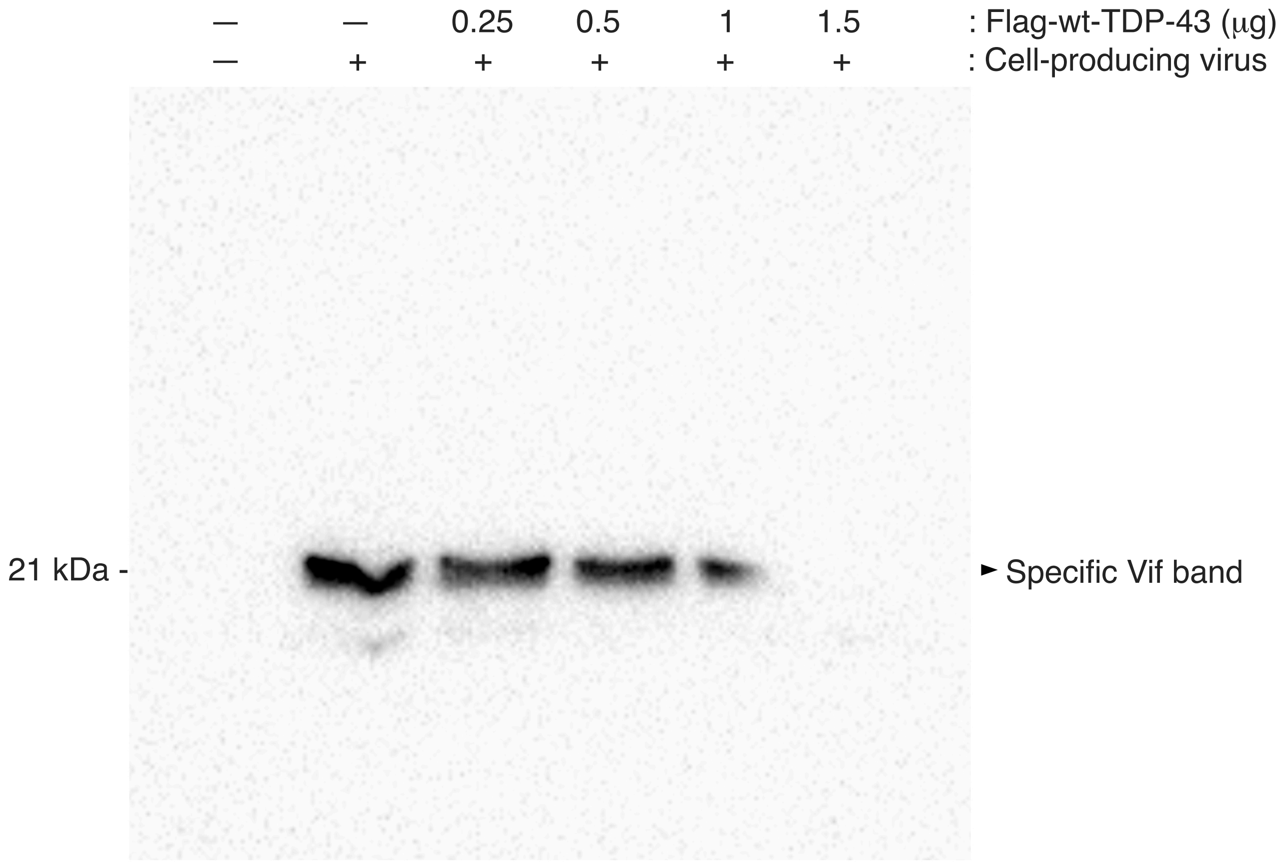

Figure S2A. Replicate 1 Acetylated α-tubulin complete gel   Western-blot associated with Figure 2A

Cabrera-Rodríguez, R., *et al.*

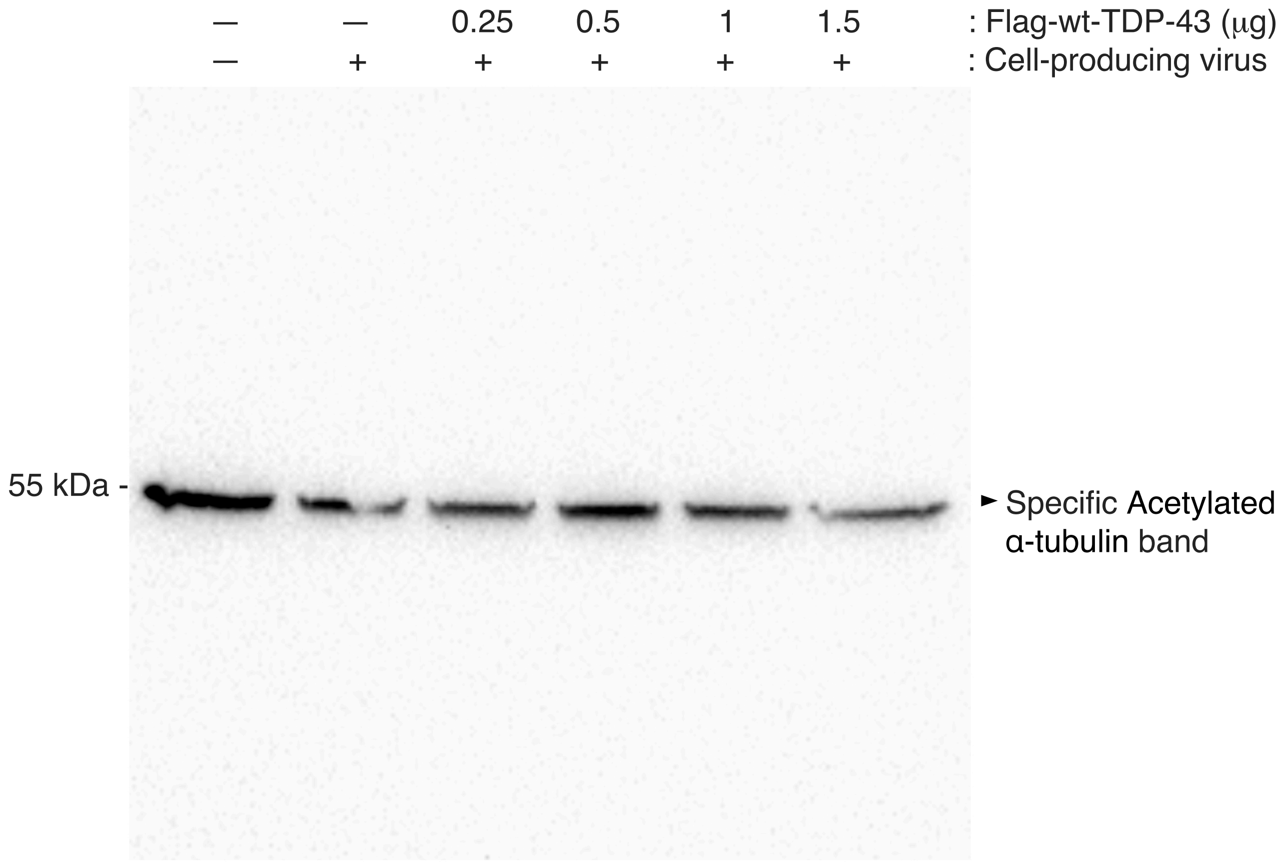

Figure S2A. Replicate 1 Total α-tubulin complete gel Western-blot associated with Figure 2A
Cabrera-Rodríguez, R., *et al.*

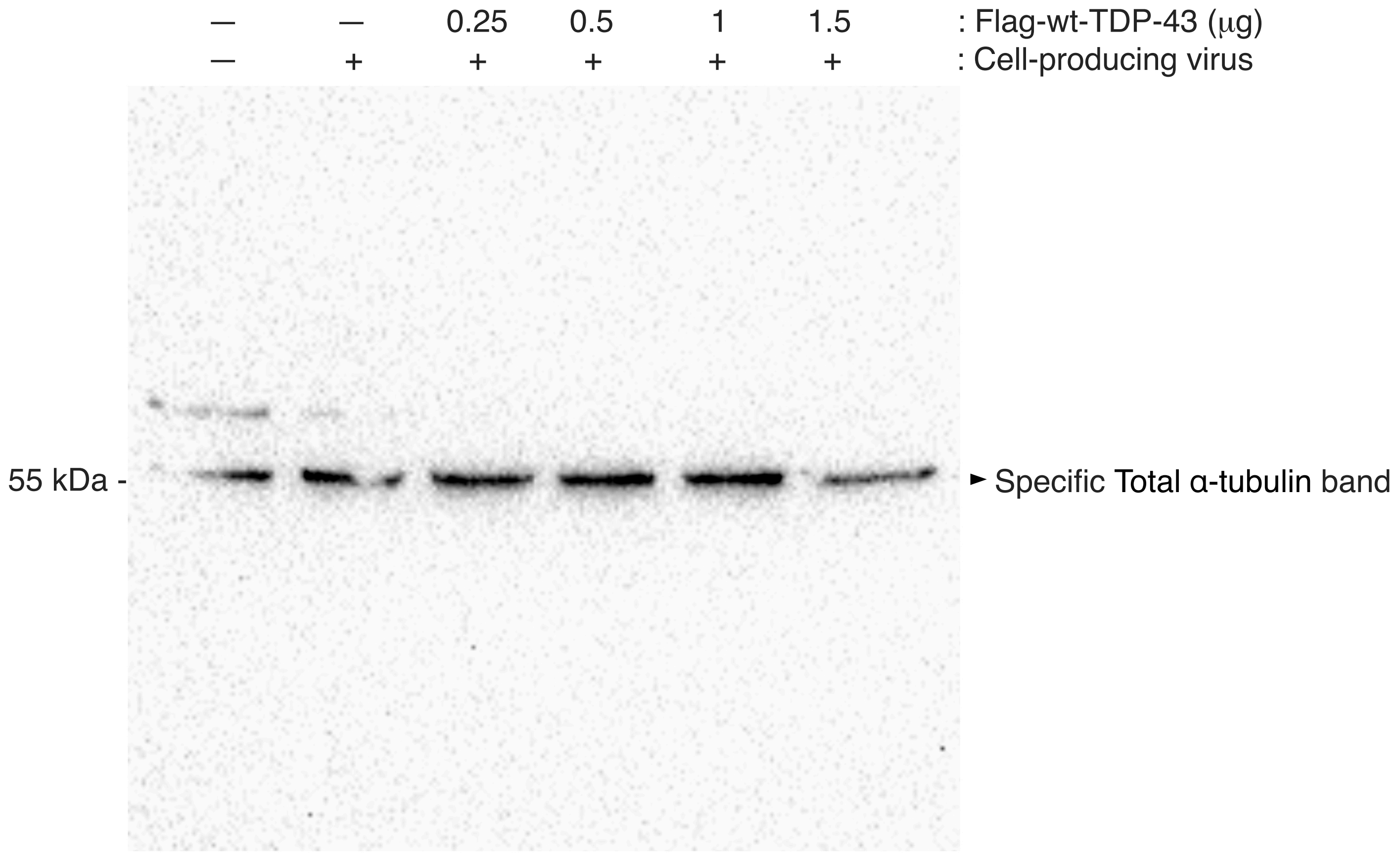

Figure S2A. Replicate 2 HDAC6 complete gel Western-blot associated with Figure 2A
Cabrera-Rodríguez, R., *et al.*

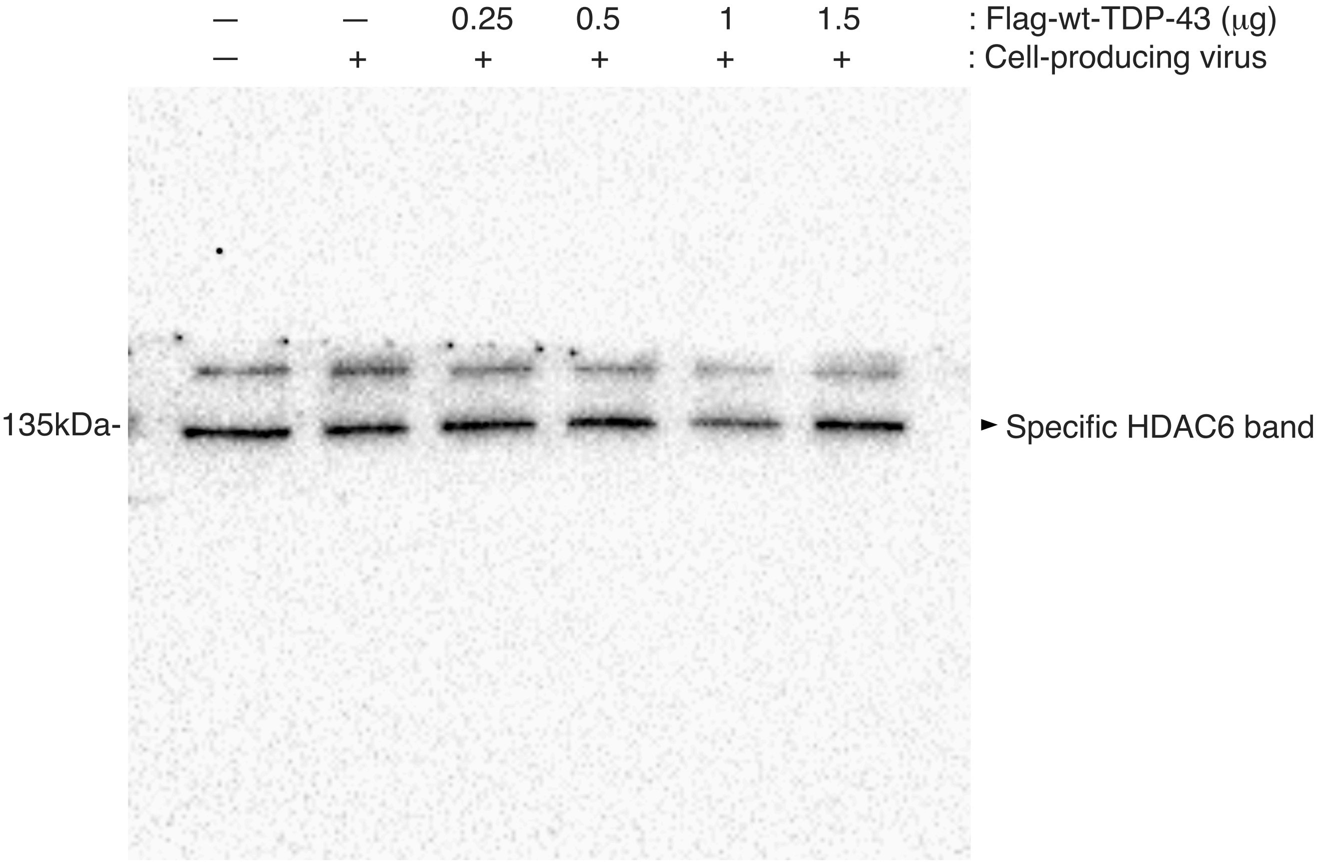

Figure S2A. Replicate 2 p62 complete gel   Western-blot associated with Figure 2A
Cabrera-Rodríguez, R., *et al.*

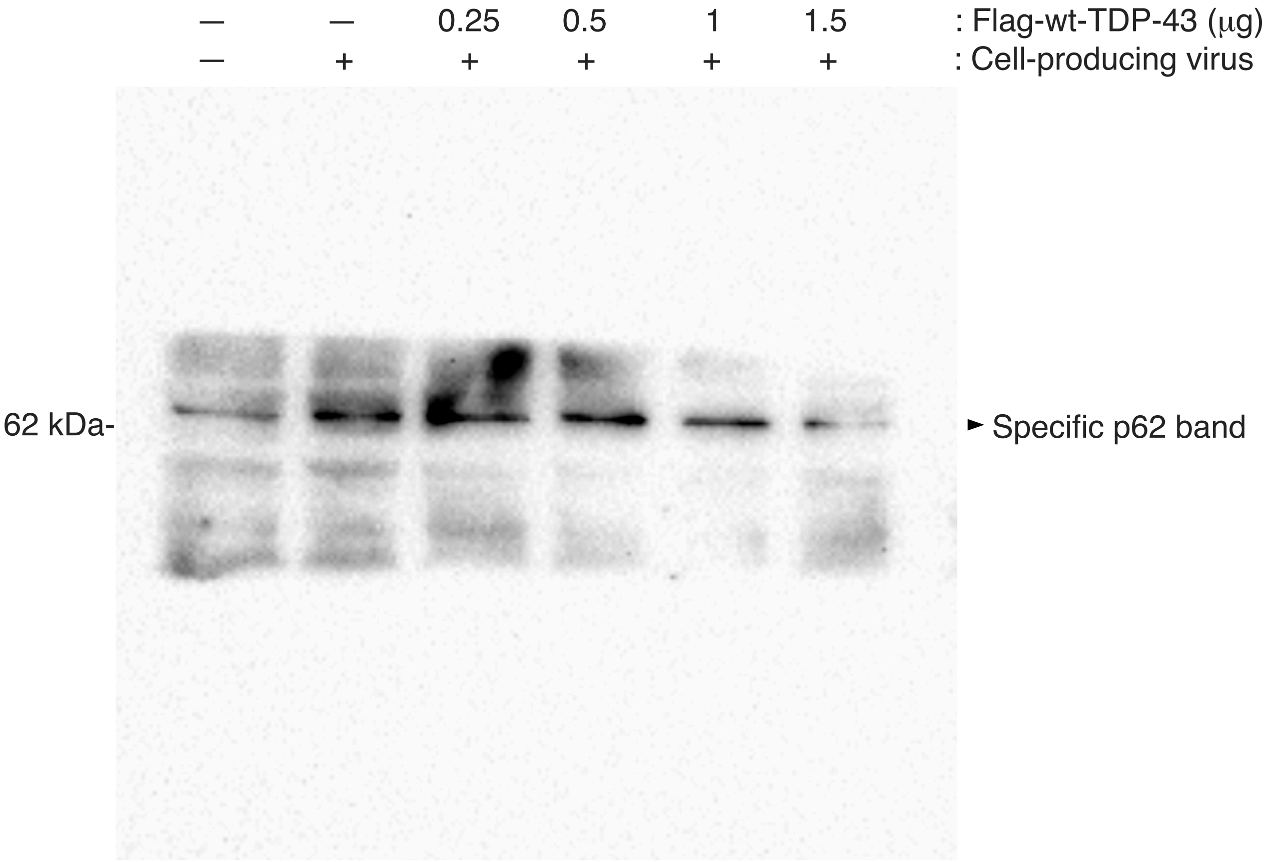

Figure S2A. Replicate 2 TDP-43 complete gel   Western-blot associated with Figure 2A
Cabrera-Rodríguez, R., *et al.*

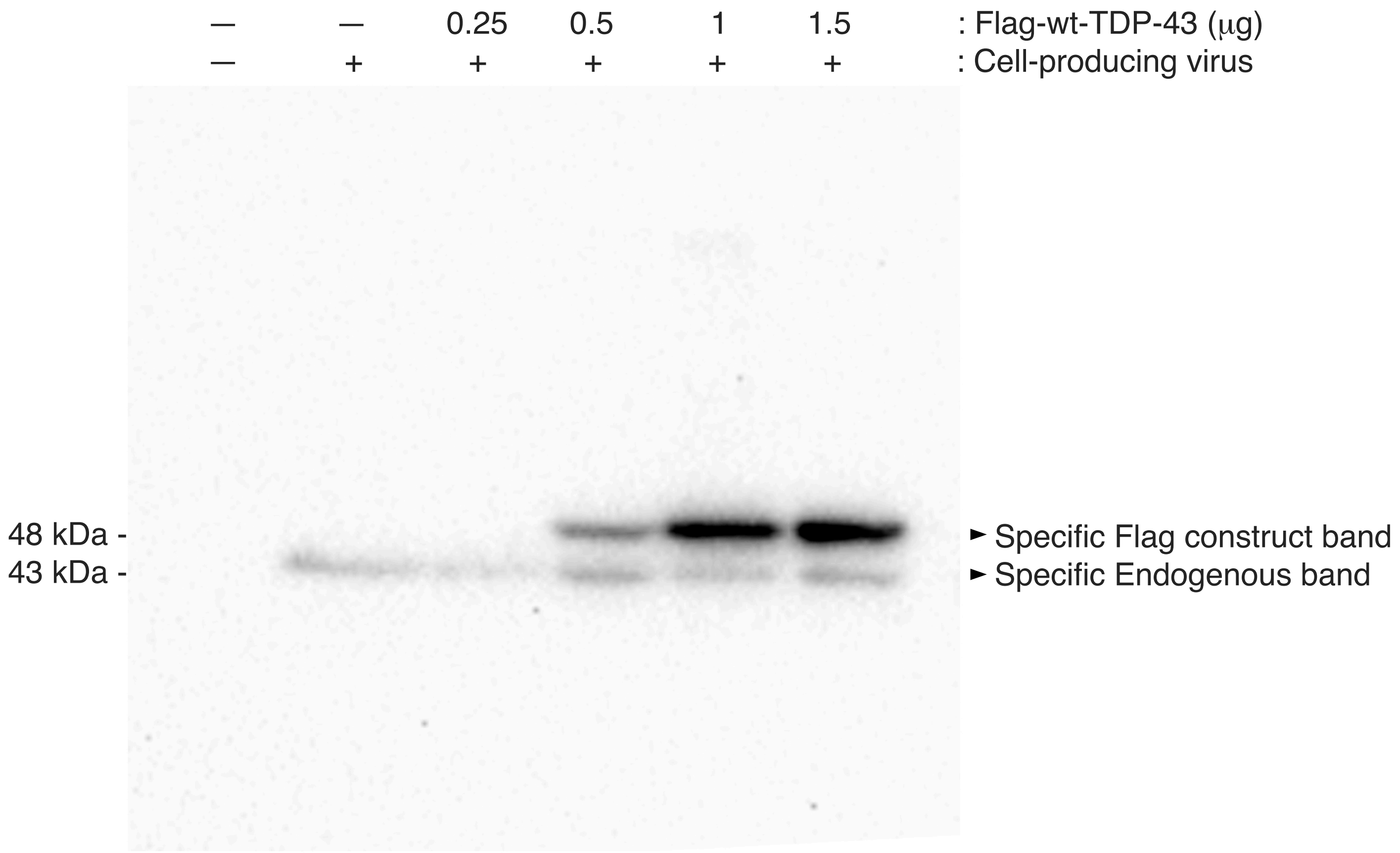

Figure S2A. Replicate 2 Pr55^{Gag} complete gel Western-blot associated with Figure 2A

Cabrera-Rodríguez, R., *et al.*

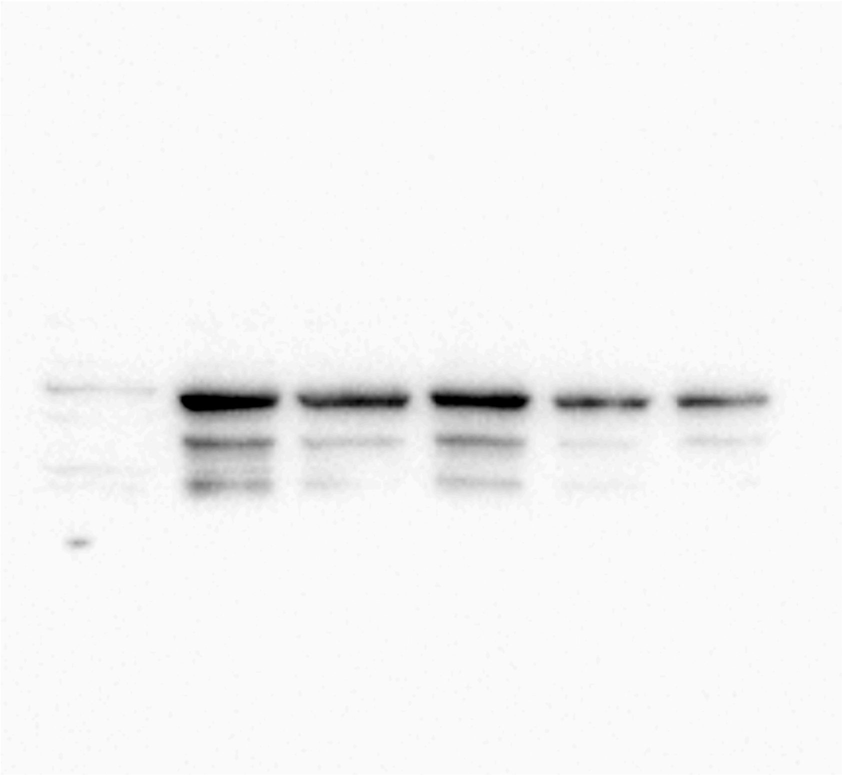

Figure S2A. Replicate 2 Vif complete gel   Western-blot associated with Figure 2A
Cabrera-Rodríguez, R., *et al.*

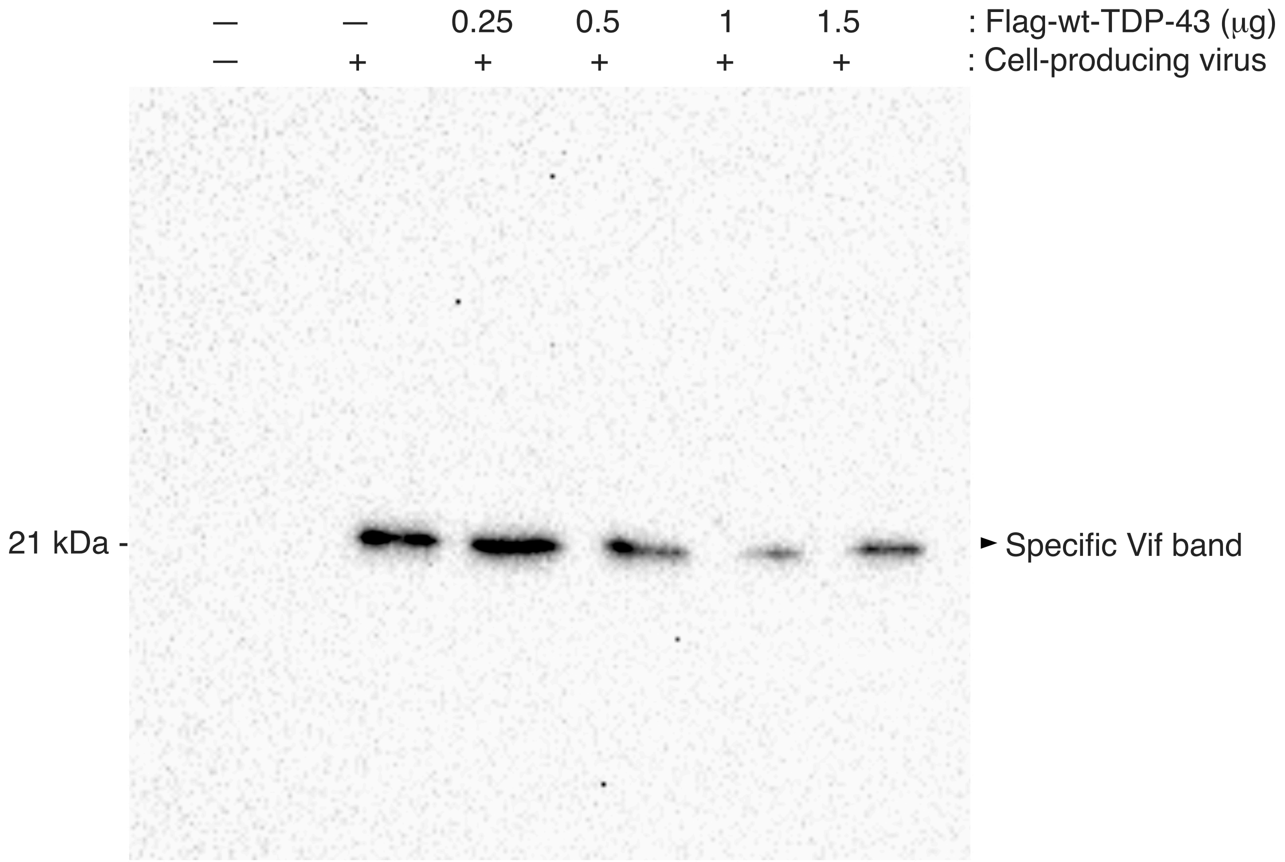

Figure S2A. Replicate 2 Acetylated α-tubulin complete gel Western-blot associated with Figure 2A

Cabrera-Rodríguez, R., *et al.*

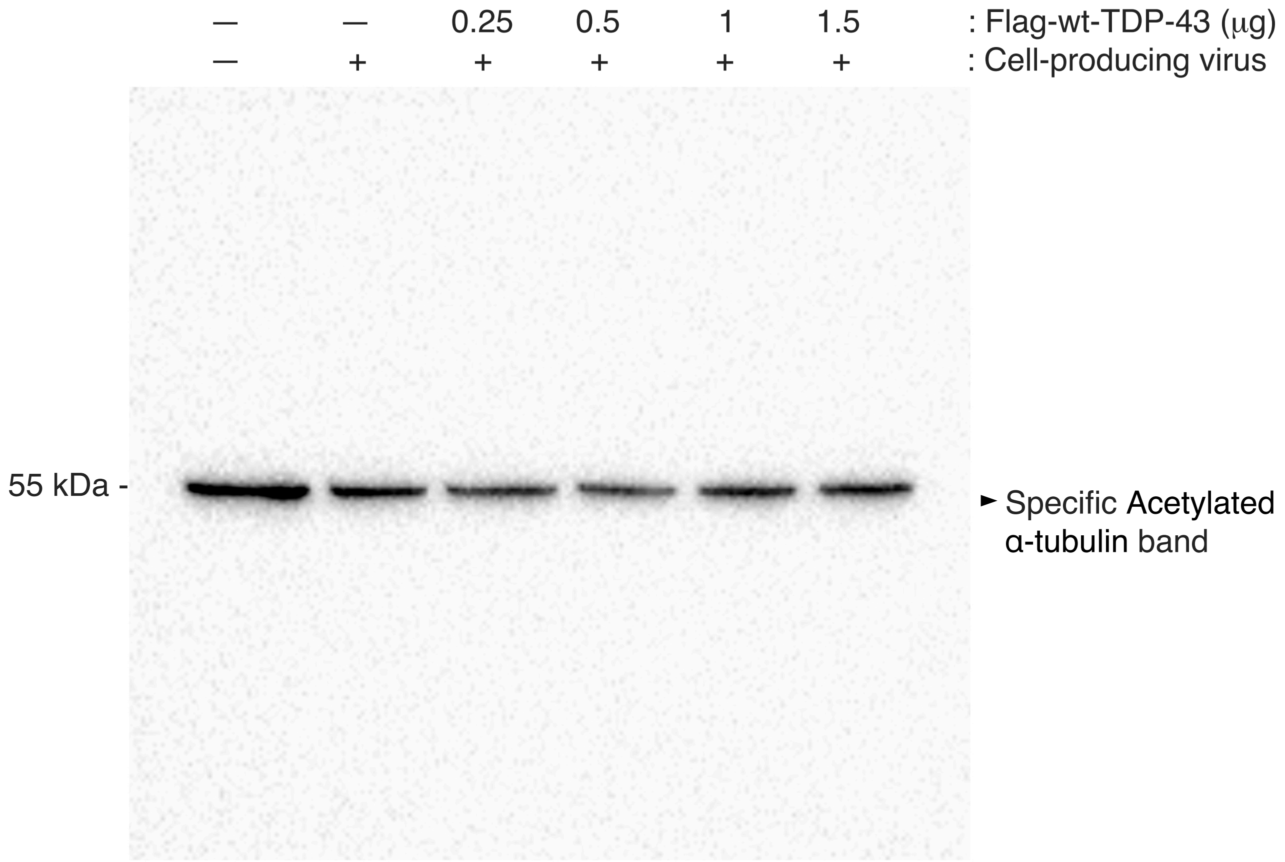

Figure S2A. Replicate 2 Total α-tubulin complete gel Western-blot associated with Figure 2A

Cabrera-Rodríguez, R., *et al.*

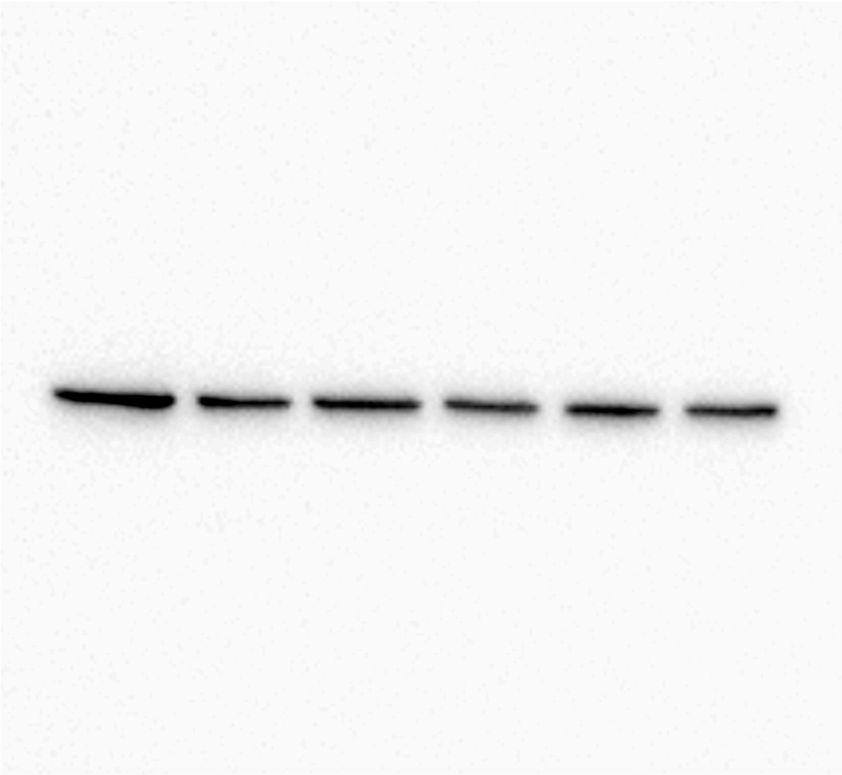

Figure S2A. Replicate 3 HDAC6 complete gel Western-blot associated with Figure 2A
Cabrera-Rodríguez, R., *et al.*

—	—	0.25	0.5	1	1.5	: Flag-wt-TDP-43 (μg)
—	+	+	+	+	+	: Cell-producing virus

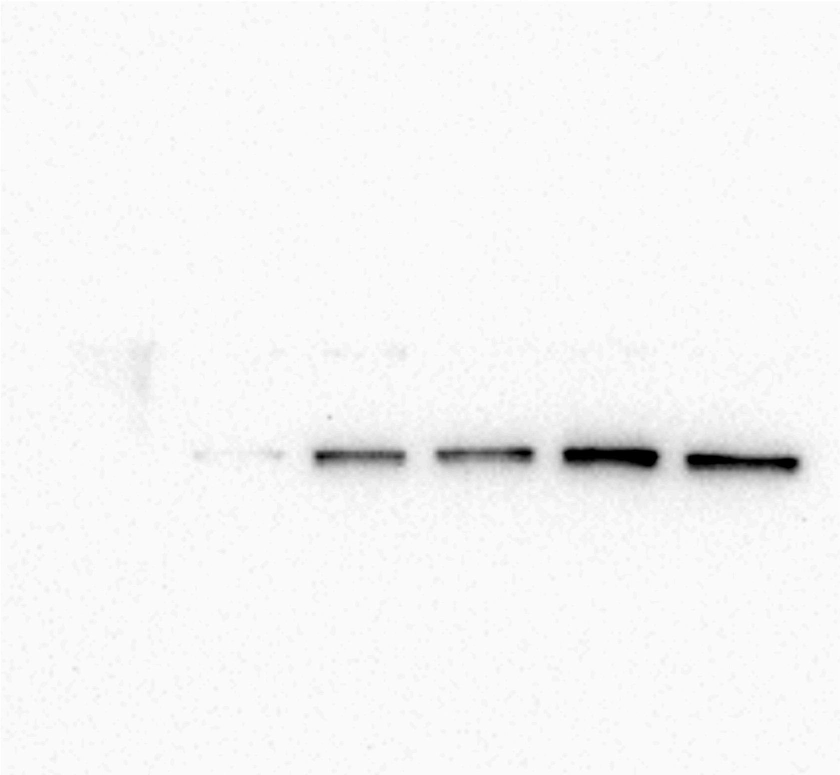

135kDa-

► Specific HDAC6 band

Figure S2A. Replicate 3 p62 complete gel Western-blot associated with Figure 2A
Cabrera-Rodríguez, R., *et al.*

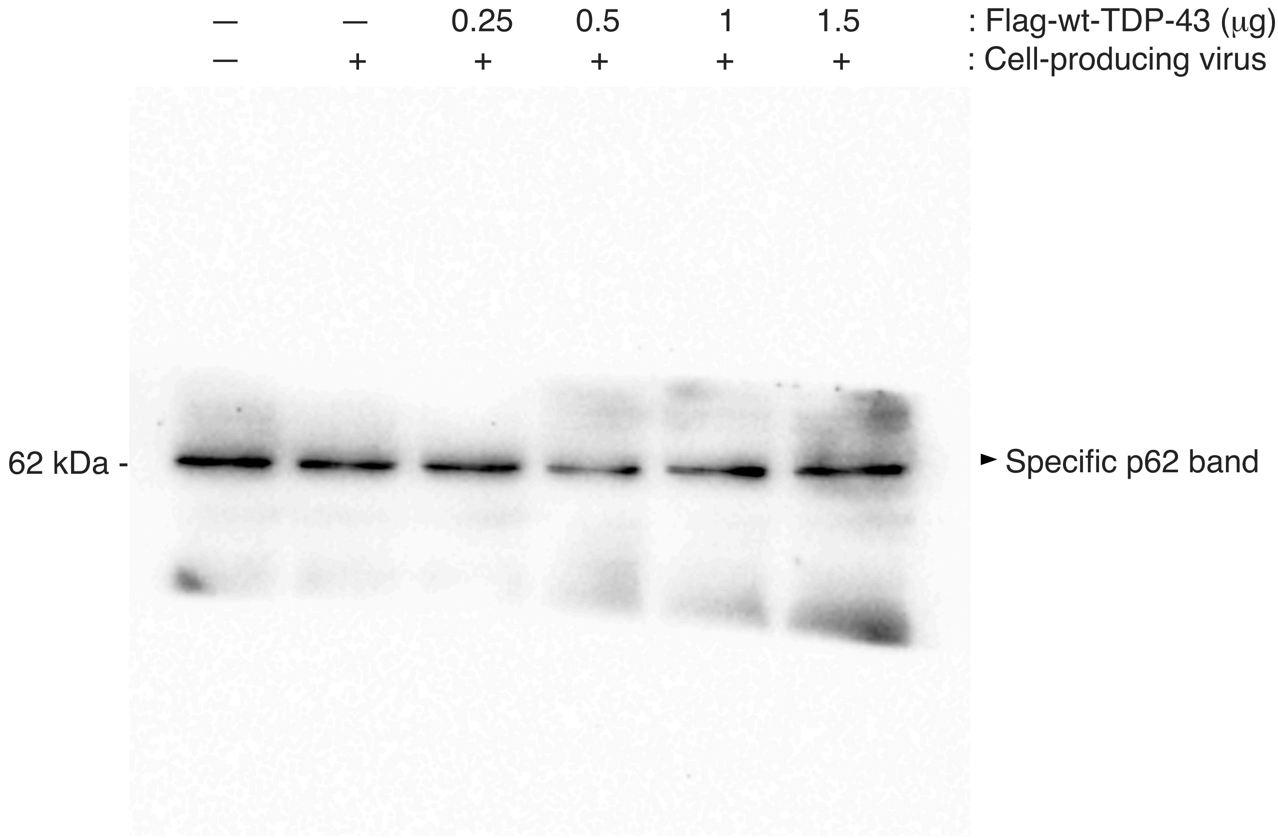

Figure S2A. Replicate 3 TDP-43 complete gel   Western-blot associated with Figure 2A
Cabrera-Rodríguez, R., *et al.*

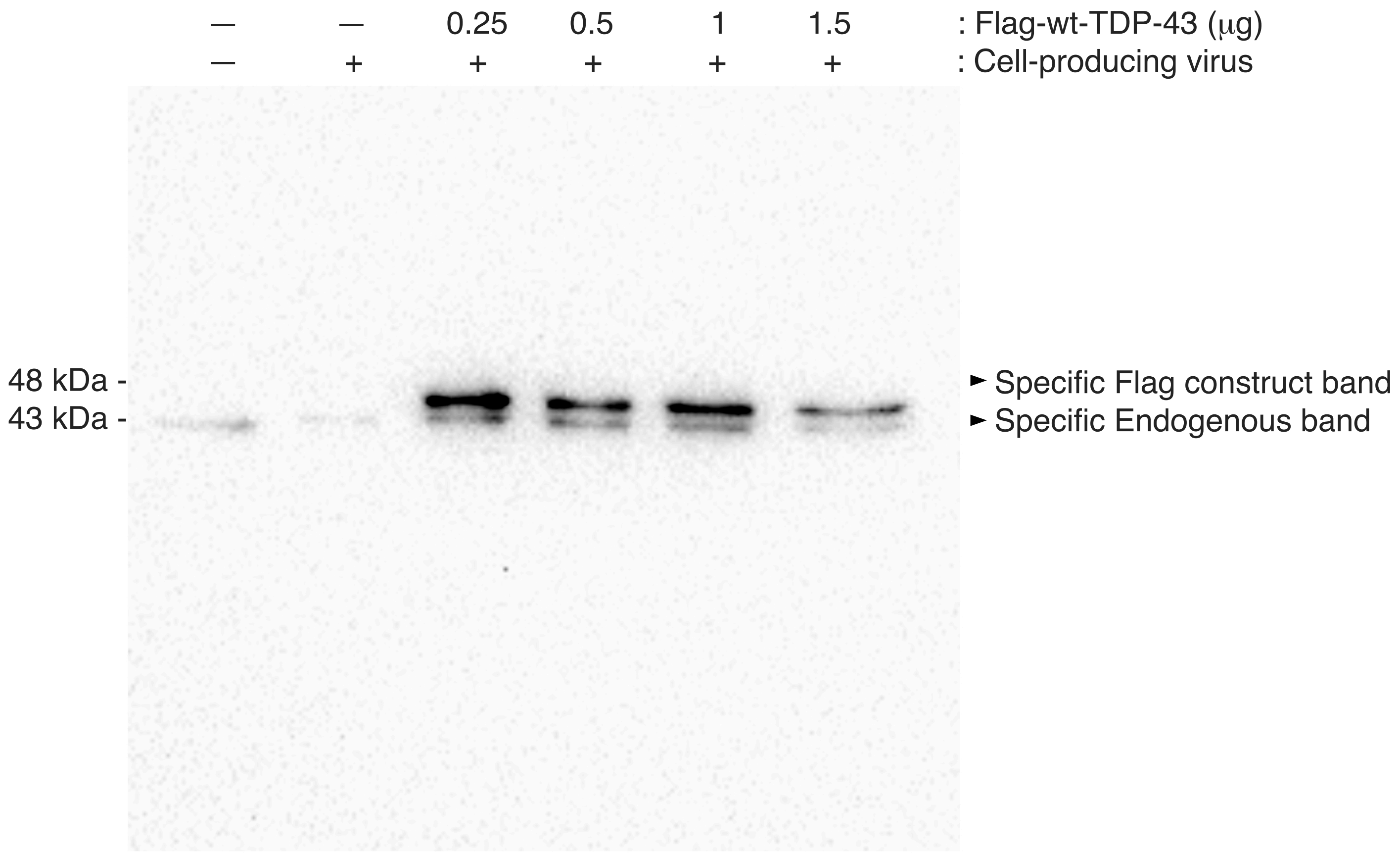

Figure S2A. Replicate 3 Pr55^{Gag} complete gel Western-blot associated with Figure 2A

Cabrera-Rodríguez, R., *et al.*

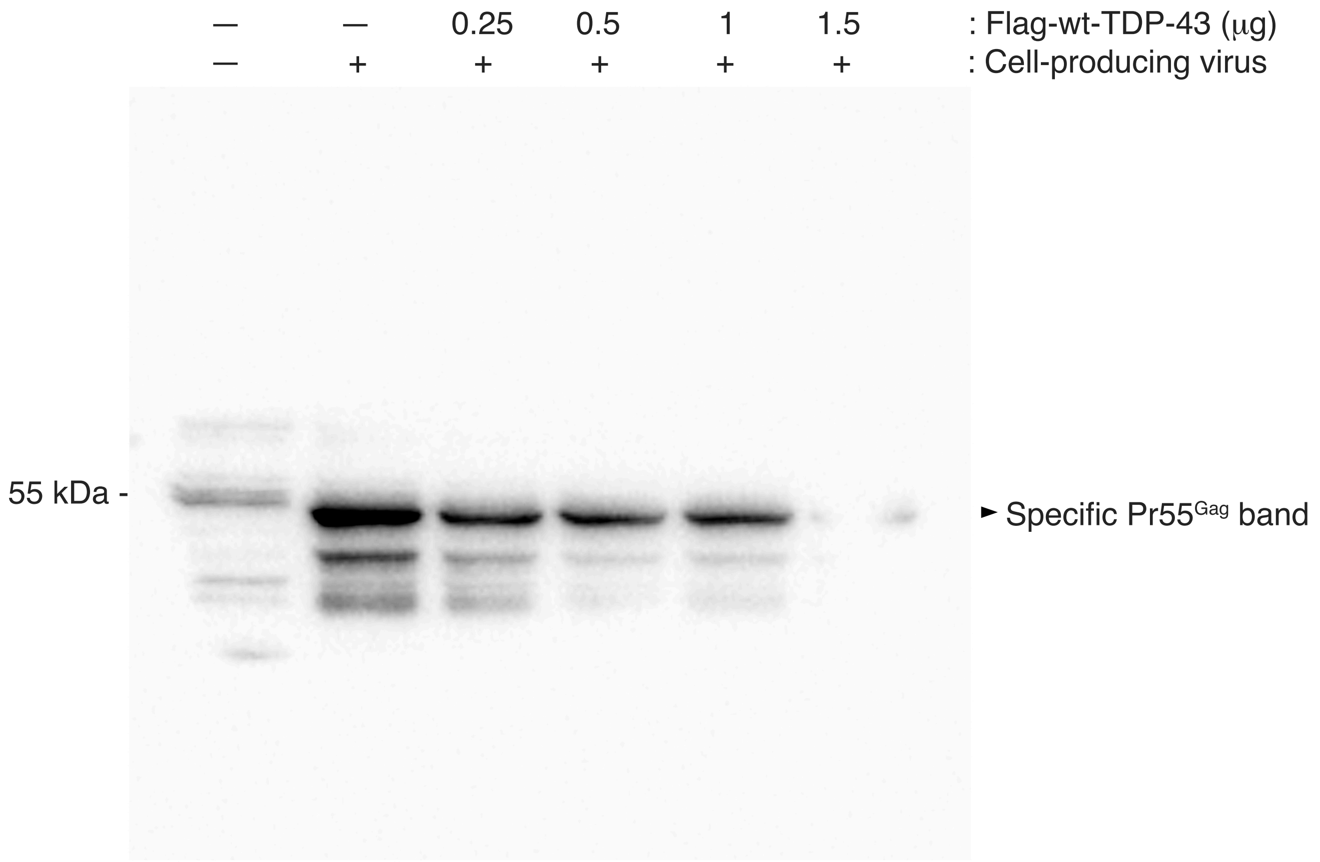

Figure S2A. Replicate 3 Vif complete gel Western-blot associated with Figure 2A

Cabrera-Rodríguez, R., *et al.*

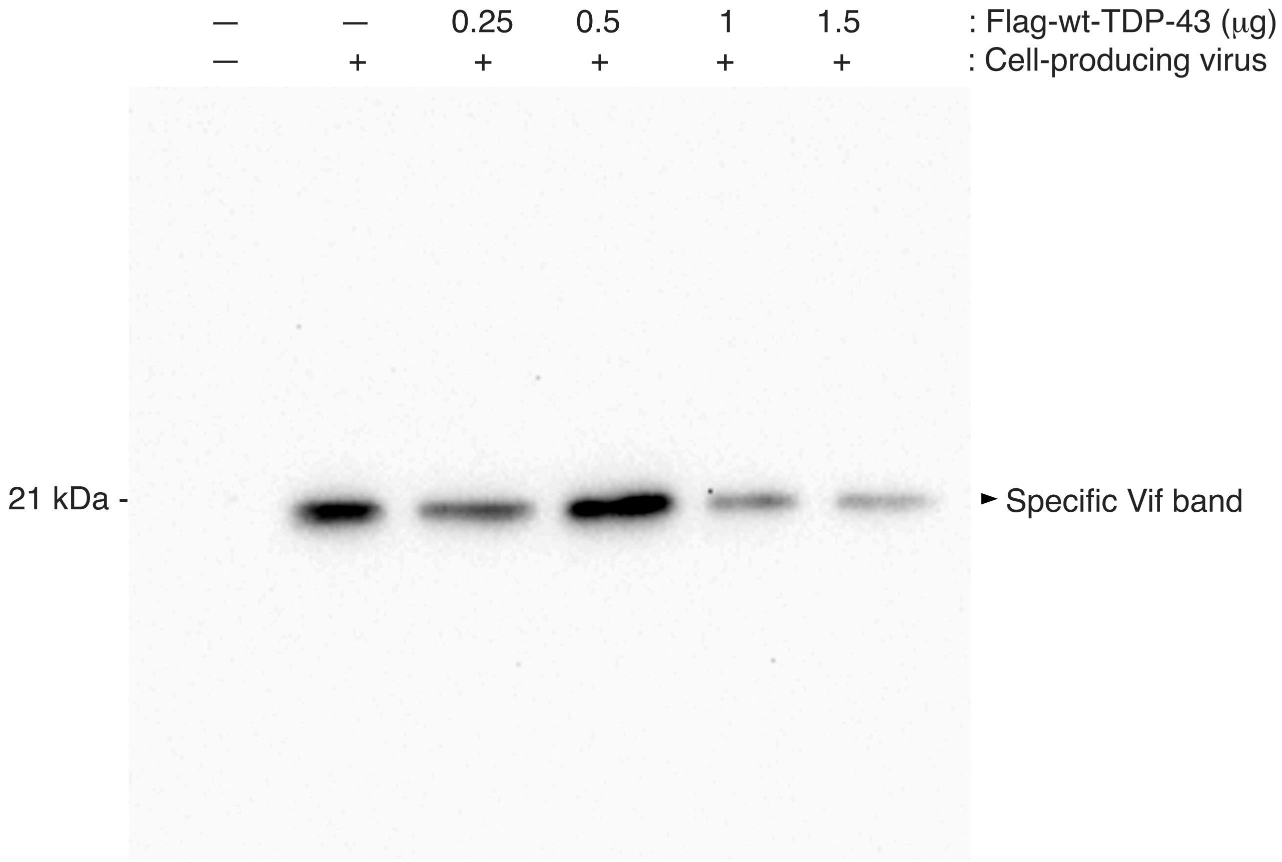

Figure S2A. Replicate 3 Acetylated α-tubulin complete gel   Western-blot associated with Figure 2A

Cabrera-Rodríguez, R., *et al.*

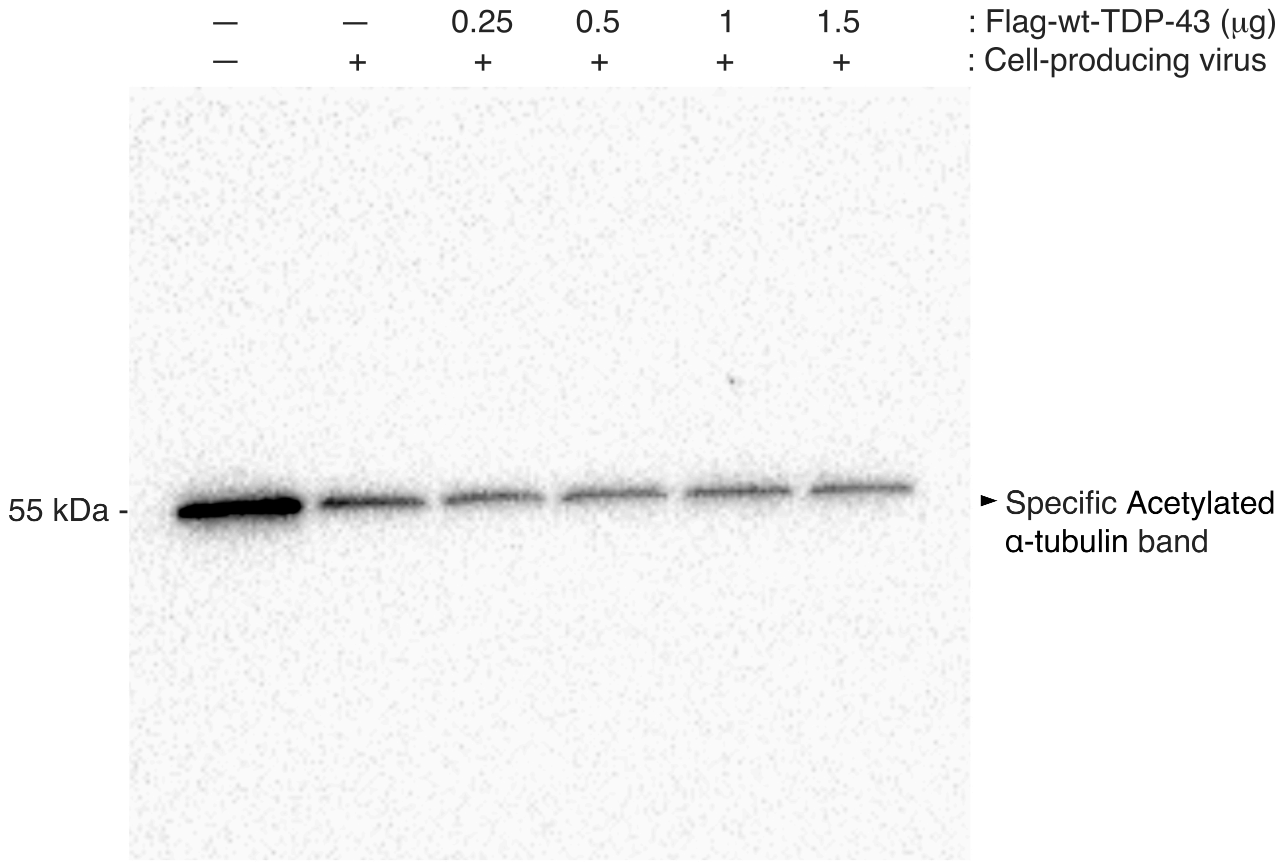

Figure S2A. Replicate 3 Total α-tubulin complete gel   Western-blot associated with Figure 2A
Cabrera-Rodríguez, R., *et al.*

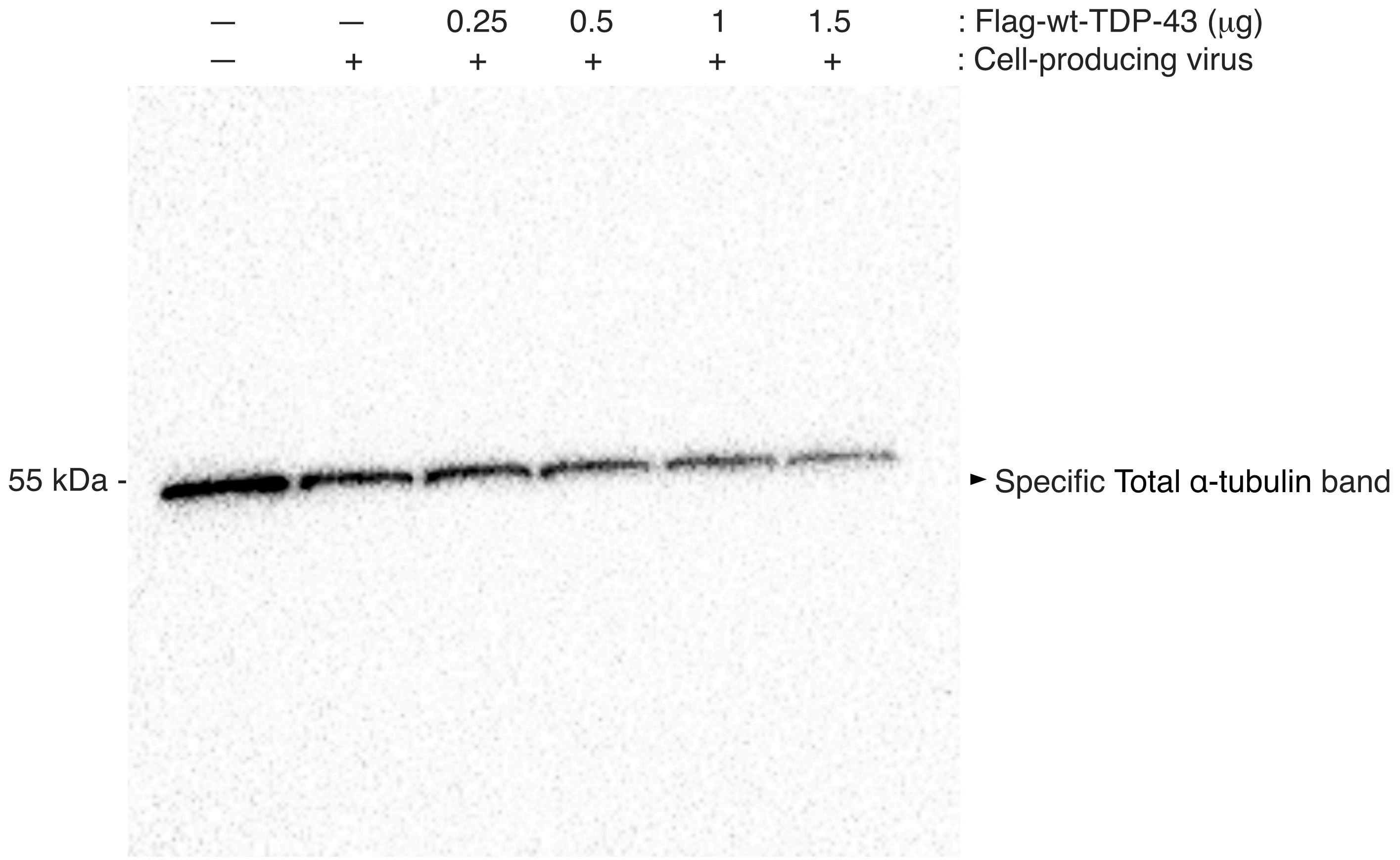

Figure S2B. Western-blot replicates associated with Figure 2B Cabrera-Rodríguez R., *et al.*

Replicate 1 as figure format

B

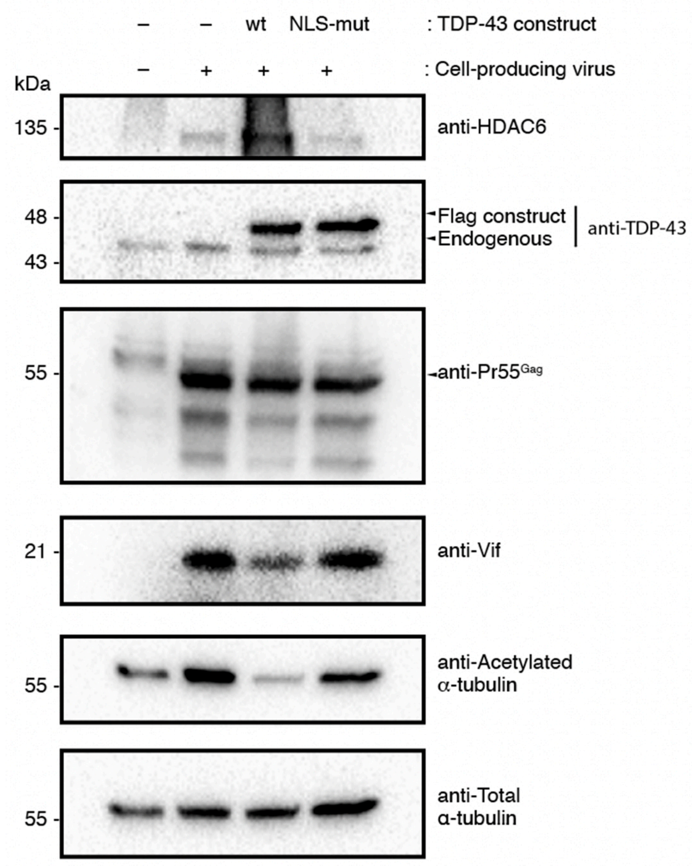

Replicate 2 as figure format

B

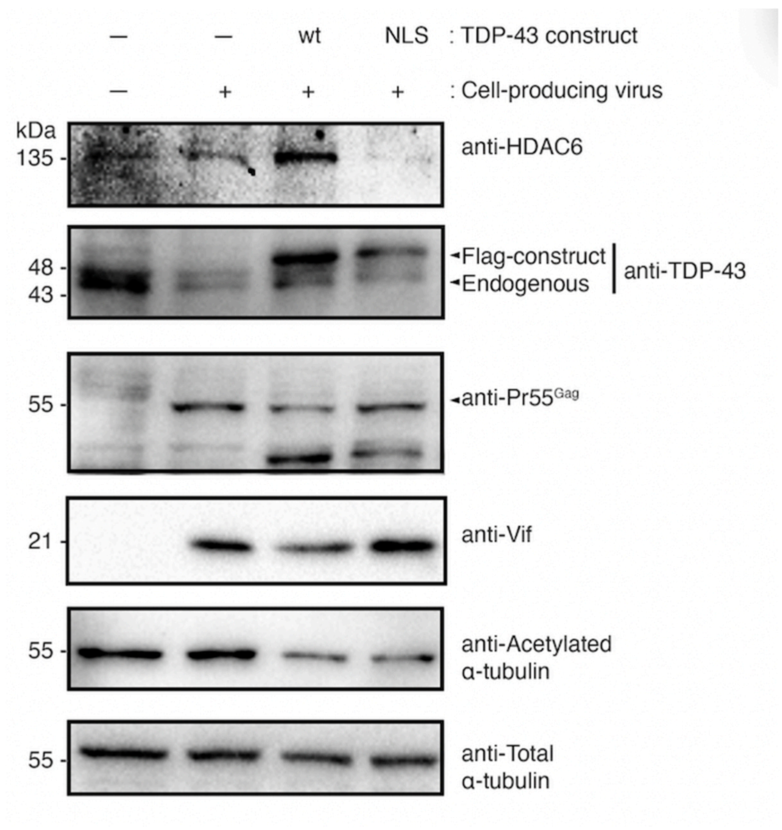

Replicate 3 as figure format

B

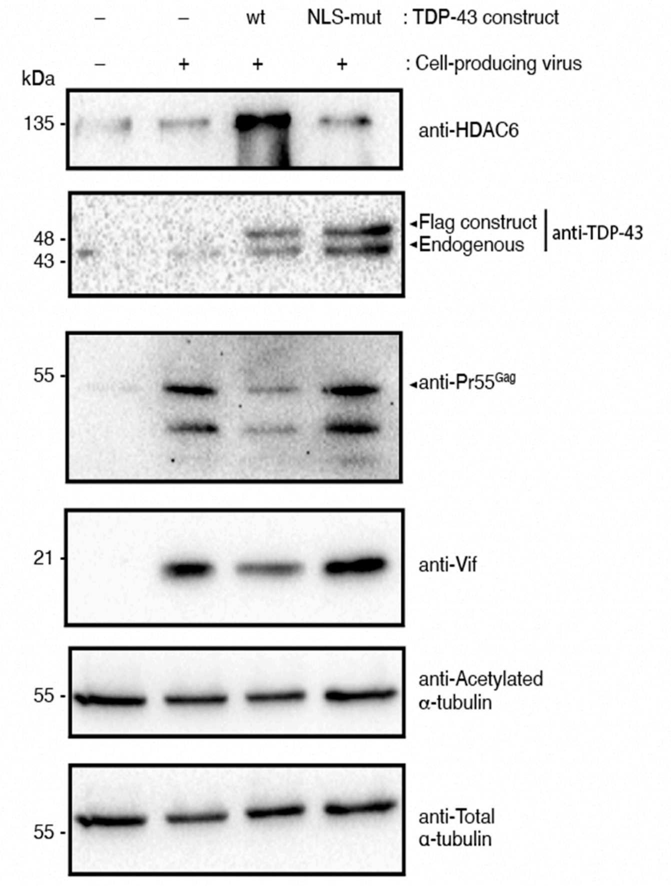

Figure S2B. Replicate 1 HDAC6 complete gel   Western-blot associated with Figure 2B
Cabrera-Rodríguez, R., *et al.*

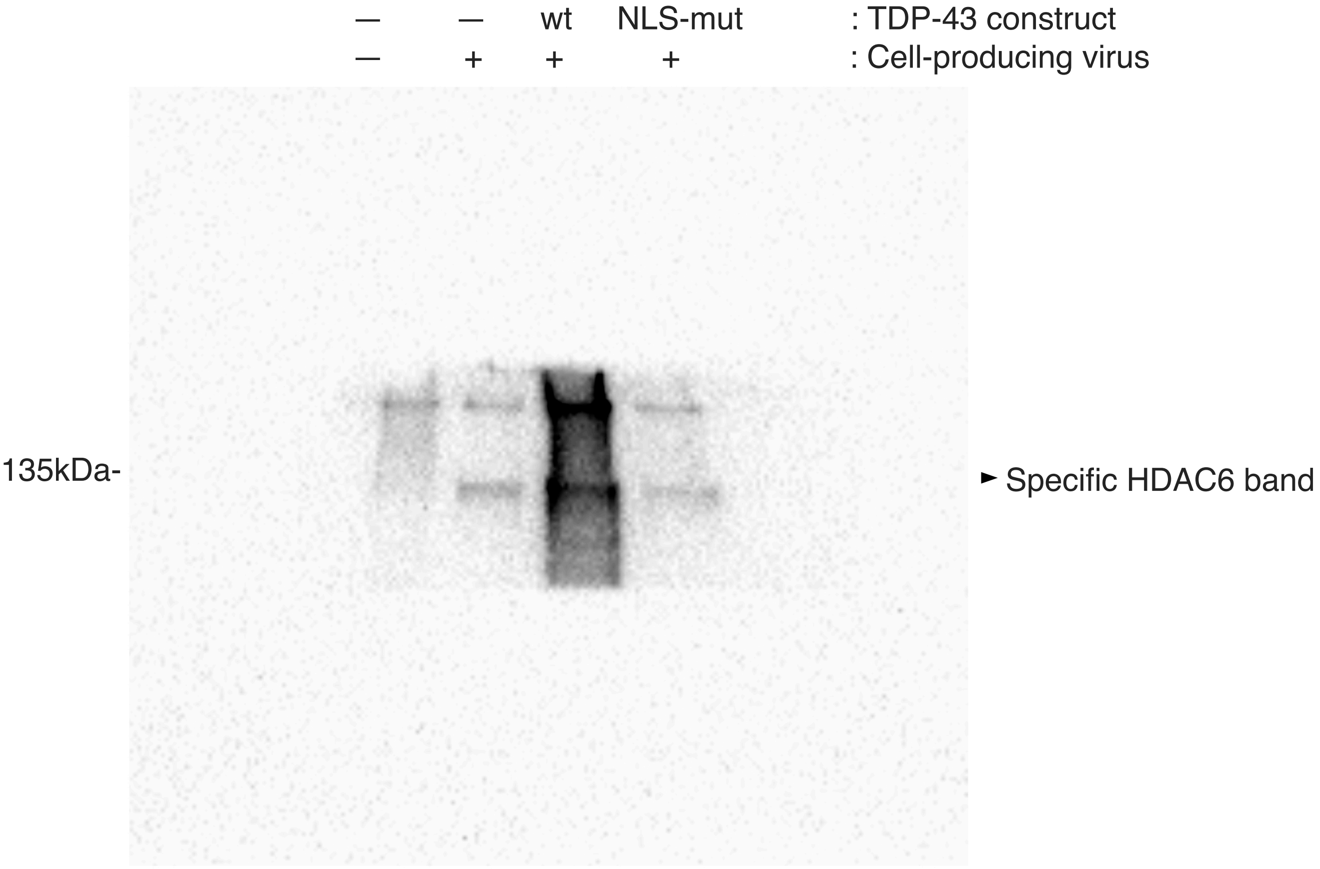

Figure S2B. Replicate 1 TDP-43 complete gel   Western-blot associated with Figure 2B
Cabrera-Rodríguez, R., *et al.*

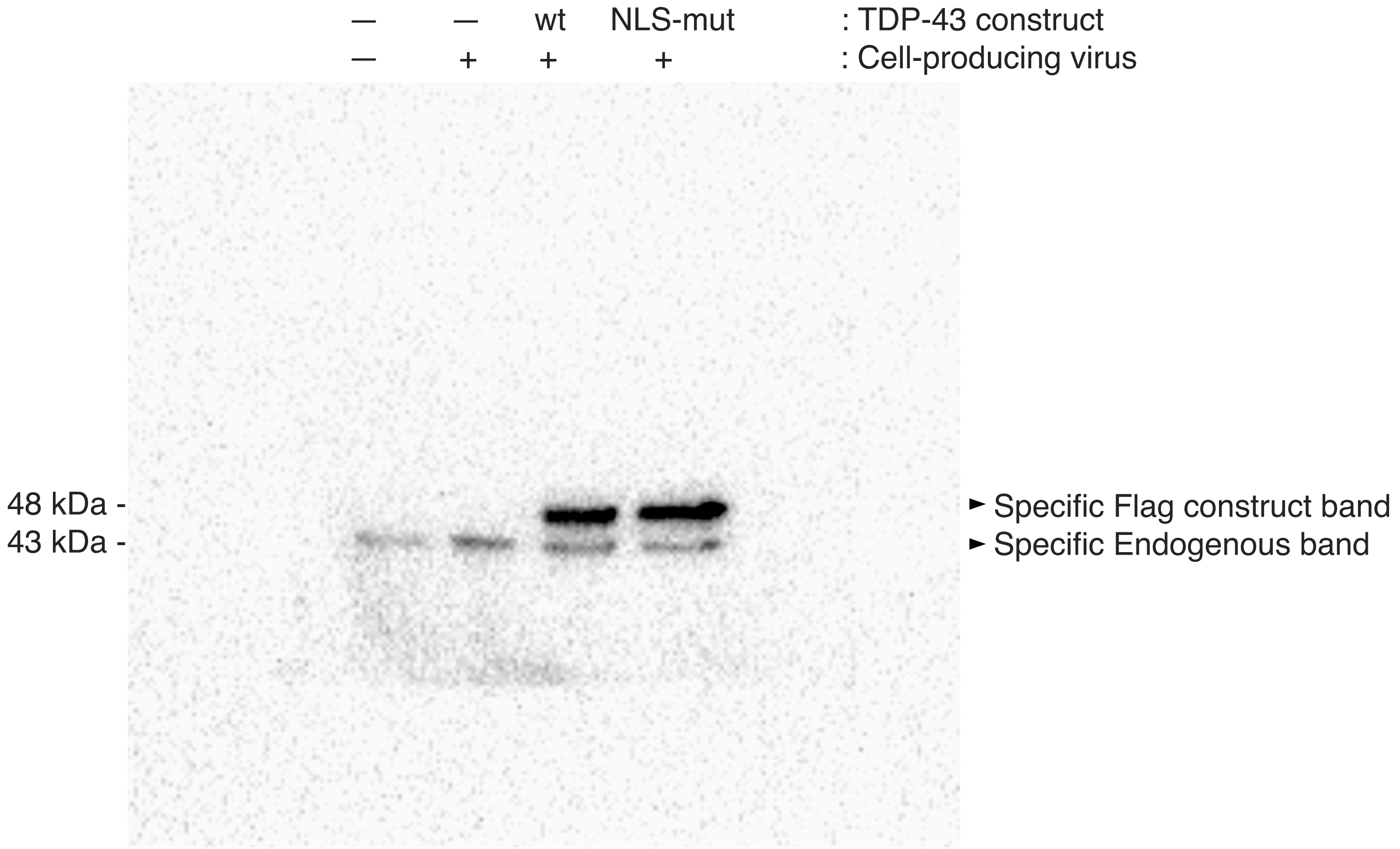

Figure S2B. Replicate 1 Pr55^{Gag} complete gel   Western-blot associated with Figure 2B
Cabrera-Rodríguez, R., *et al.*

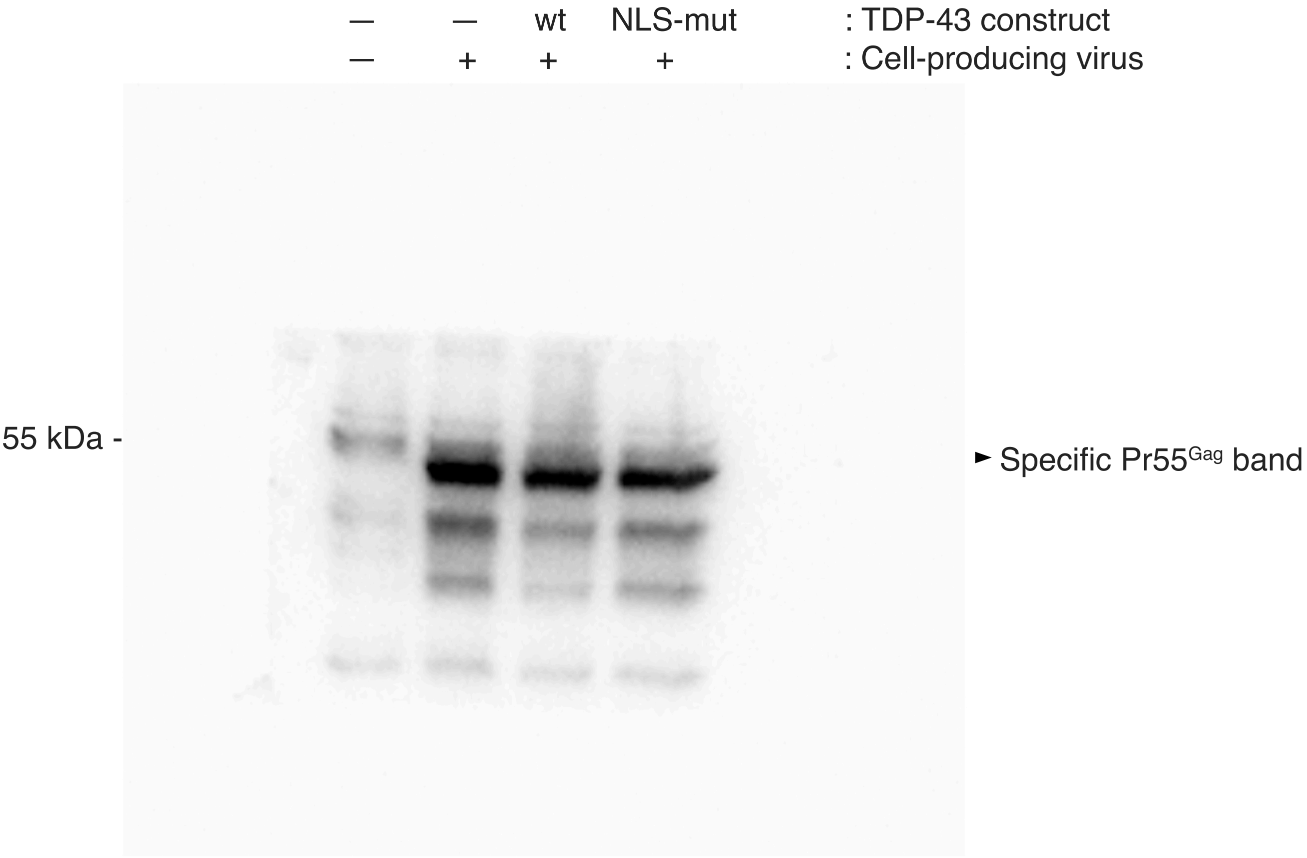

Figure S2B. Replicate 1 Vif complete gel   Western-blot associated with Figure 2B
Cabrera-Rodríguez, R., *et al.*

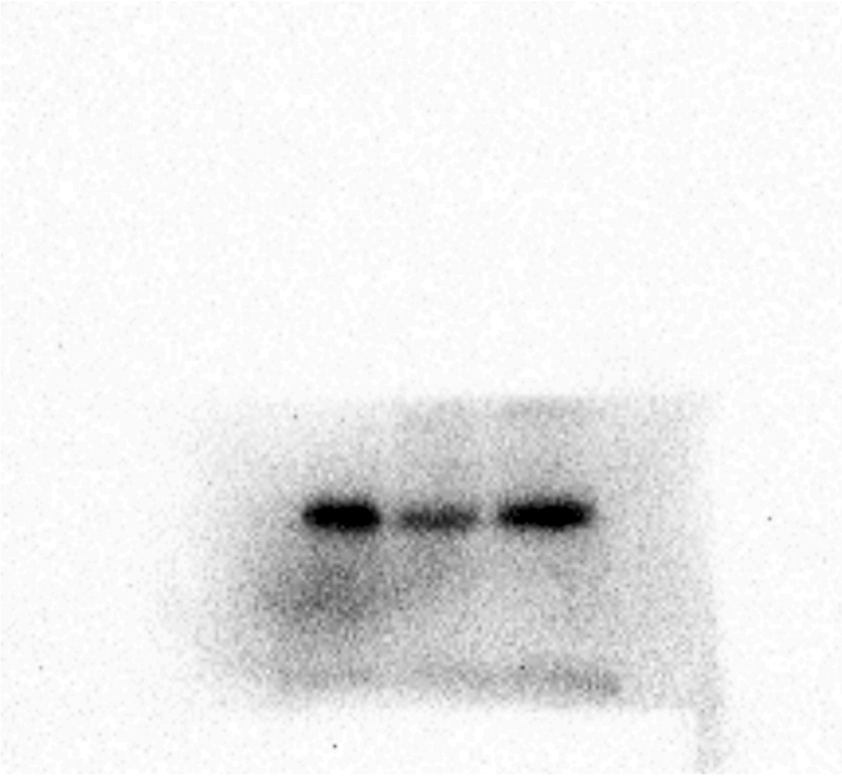

Figure S2B. Replicate 1 Acetylated α-tubulin complete gel Western-blot associated with Figure 2B

Cabrera-Rodríguez, R., *et al.*

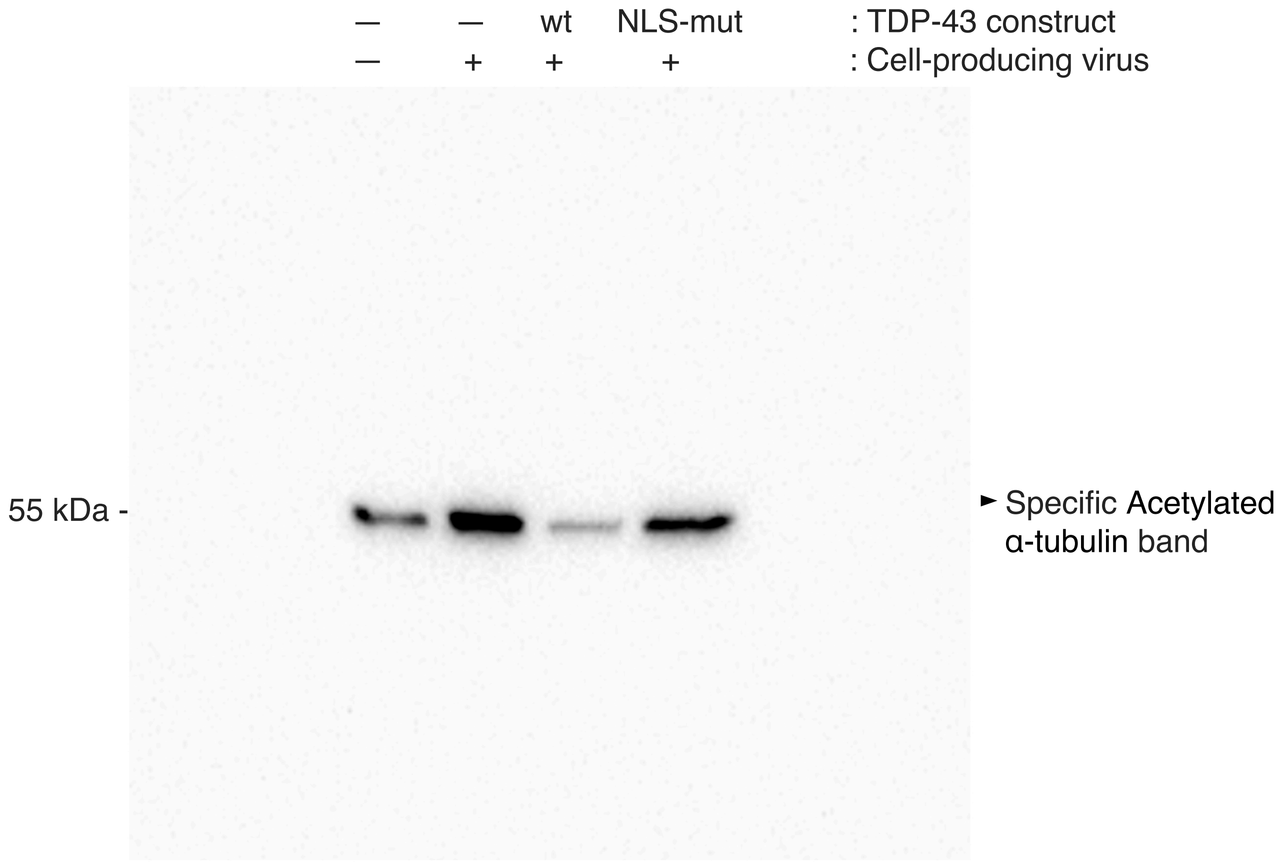

Figure S2B. Replicate 1 Total α-tubulin complete gel Western-blot associated with Figure 2B
Cabrera-Rodríguez, R., *et al.*

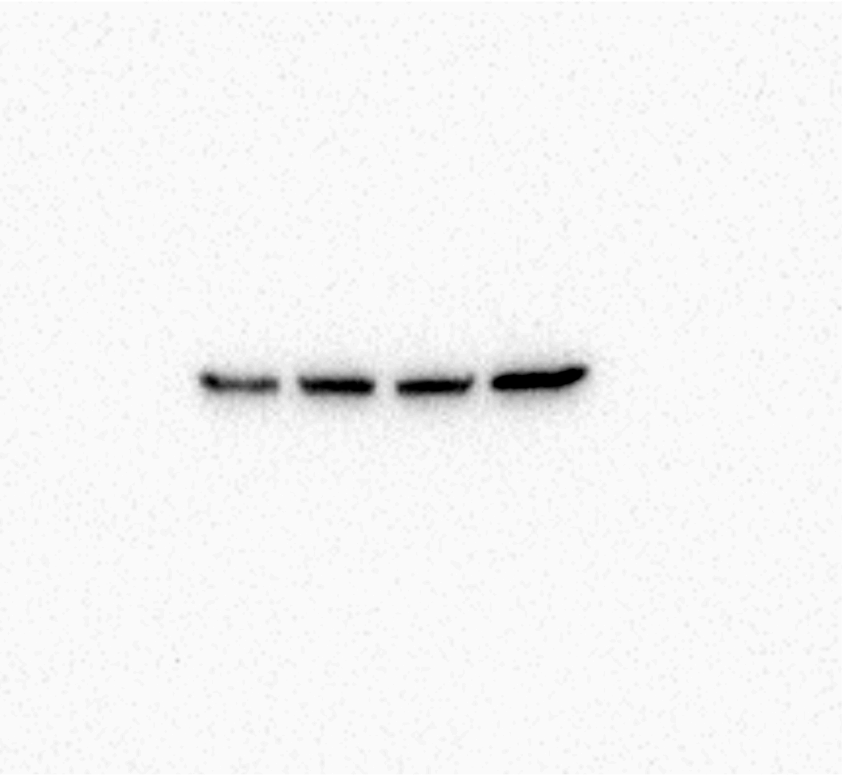

Figure S2B. Replicate 2 HDAC6 complete gel   Western-blot associated with Figure 2B
Cabrera-Rodríguez, R., *et al.*

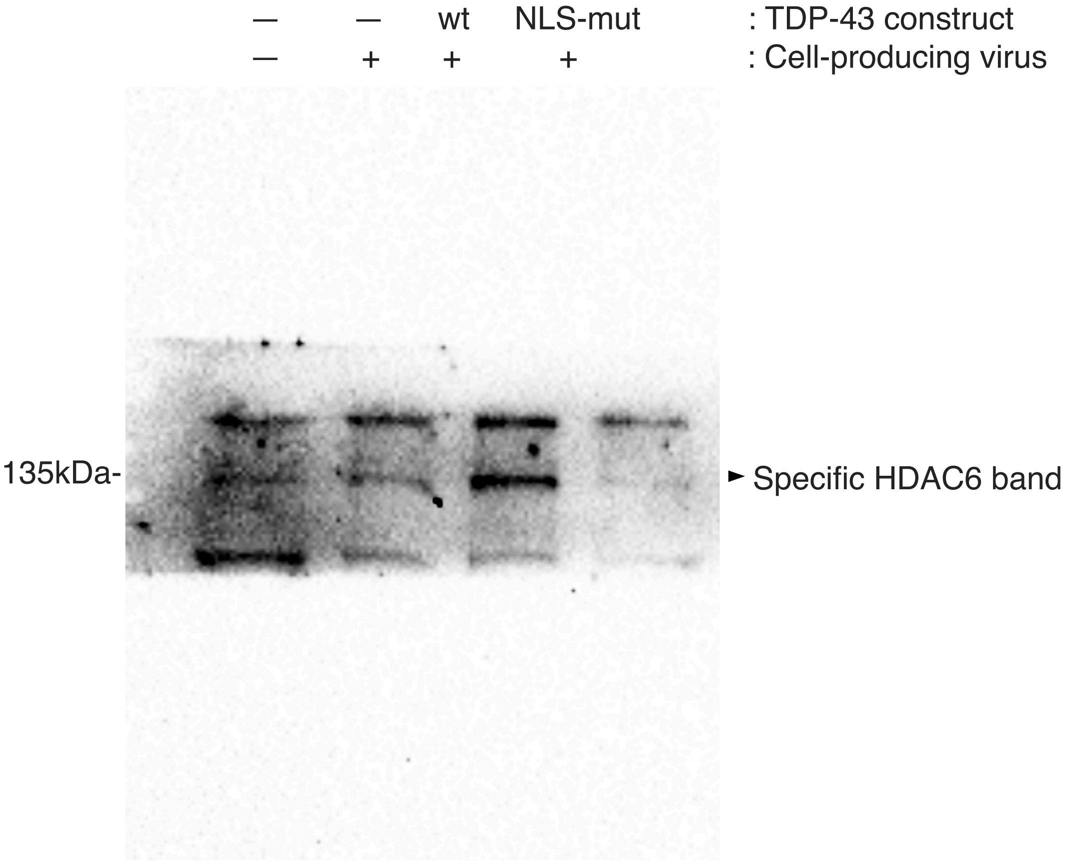

Figure S2B. Replicate 2 TDP-43 complete gel Western-blot associated with Figure 2B
Cabrera-Rodríguez, R., *et al.*

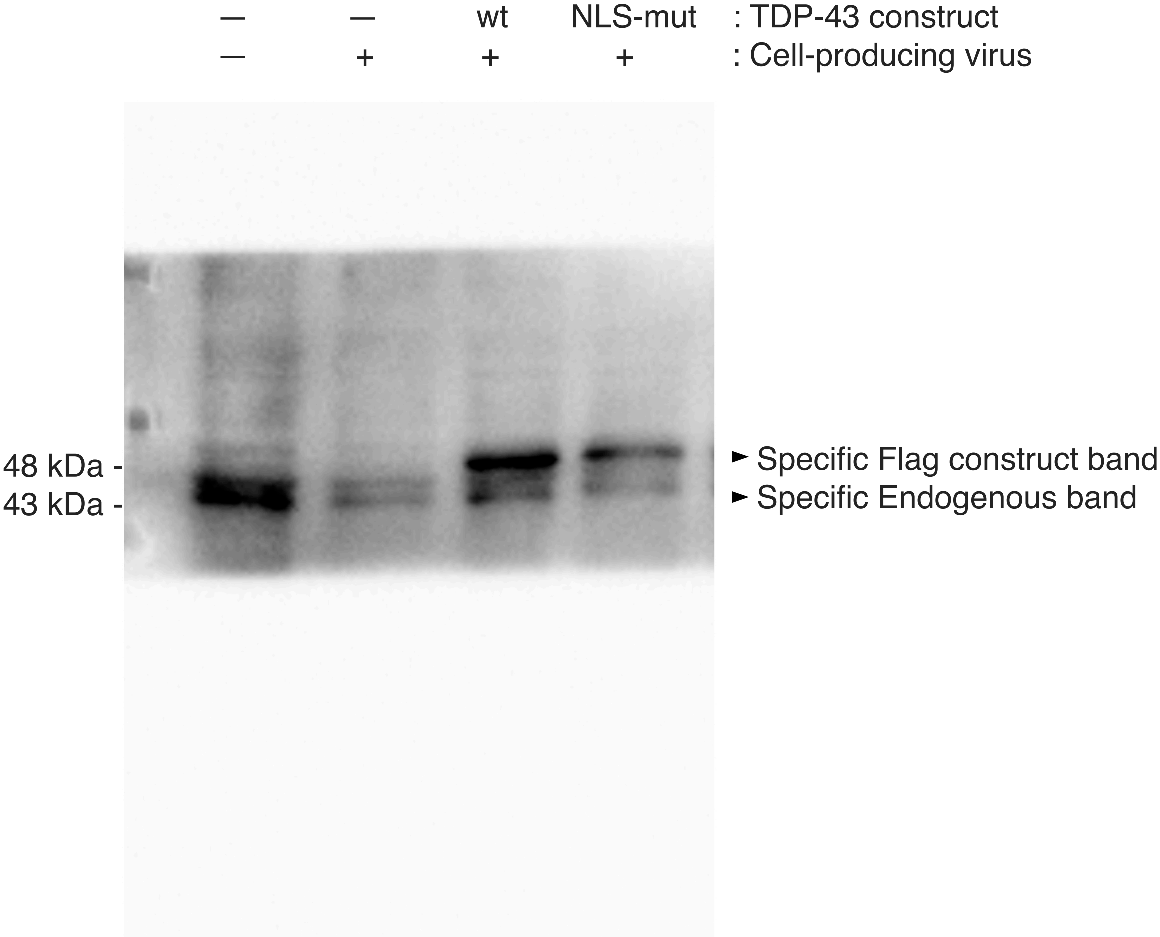

Figure S2B. Replicate 2 $Pr55^{Gag}$ complete gel   Western-blot associated with Figure 2B
Cabrera-Rodríguez, R., *et al.*

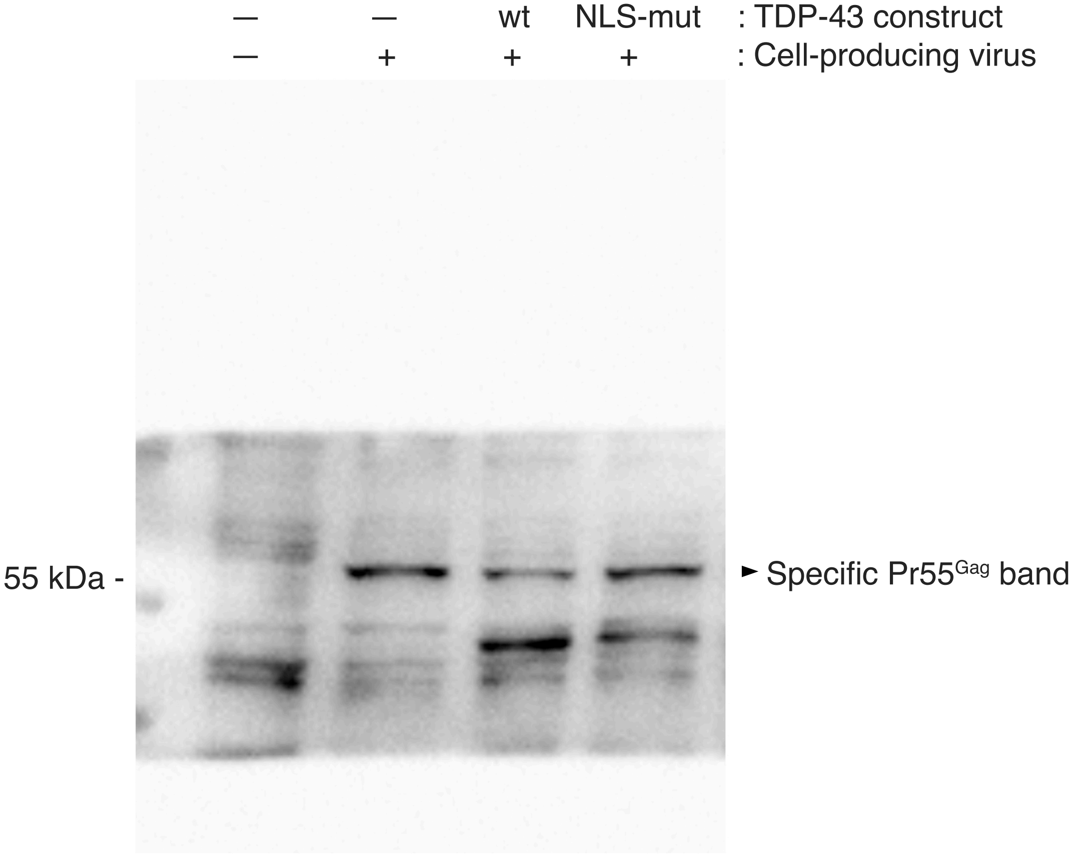

Figure S2B. Replicate 2 Vif complete gel   Western-blot associated with Figure 2B
Cabrera-Rodríguez, R., *et al.*

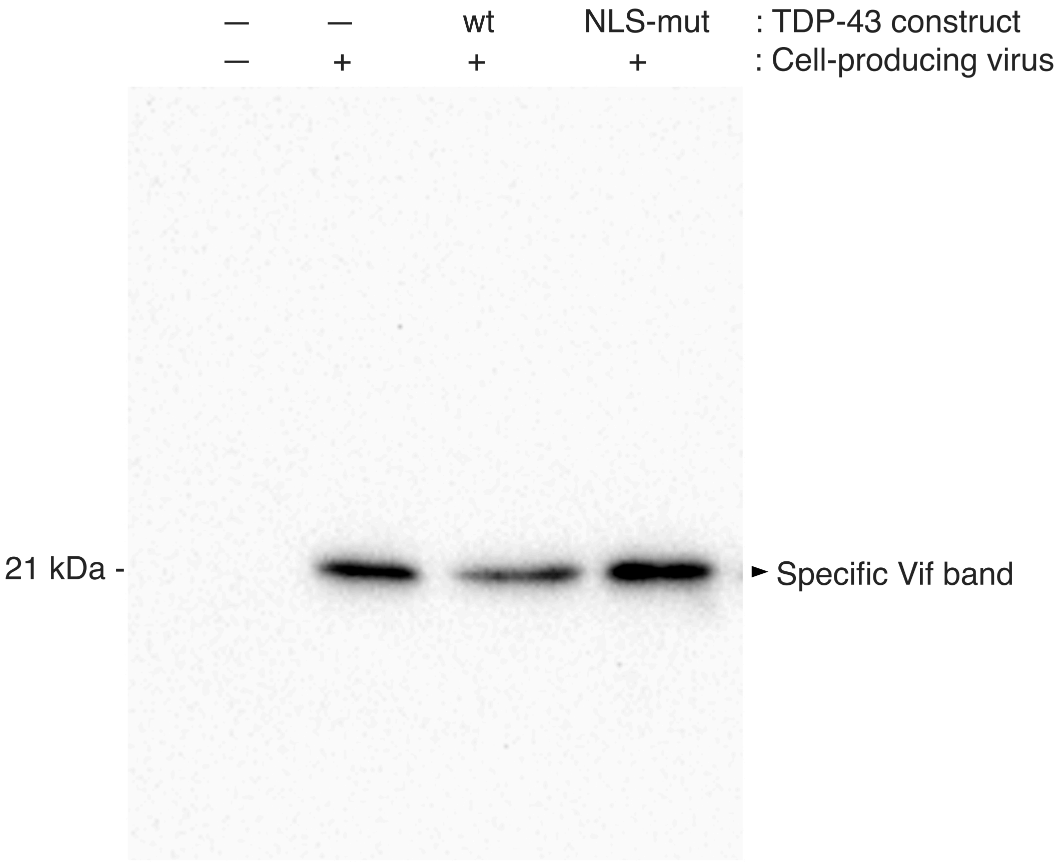

Figure S2B. Replicate 2 Acetylated α-tubulin complete gel Western-blot associated with Figure 2B
Cabrera-Rodríguez, R., *et al.*

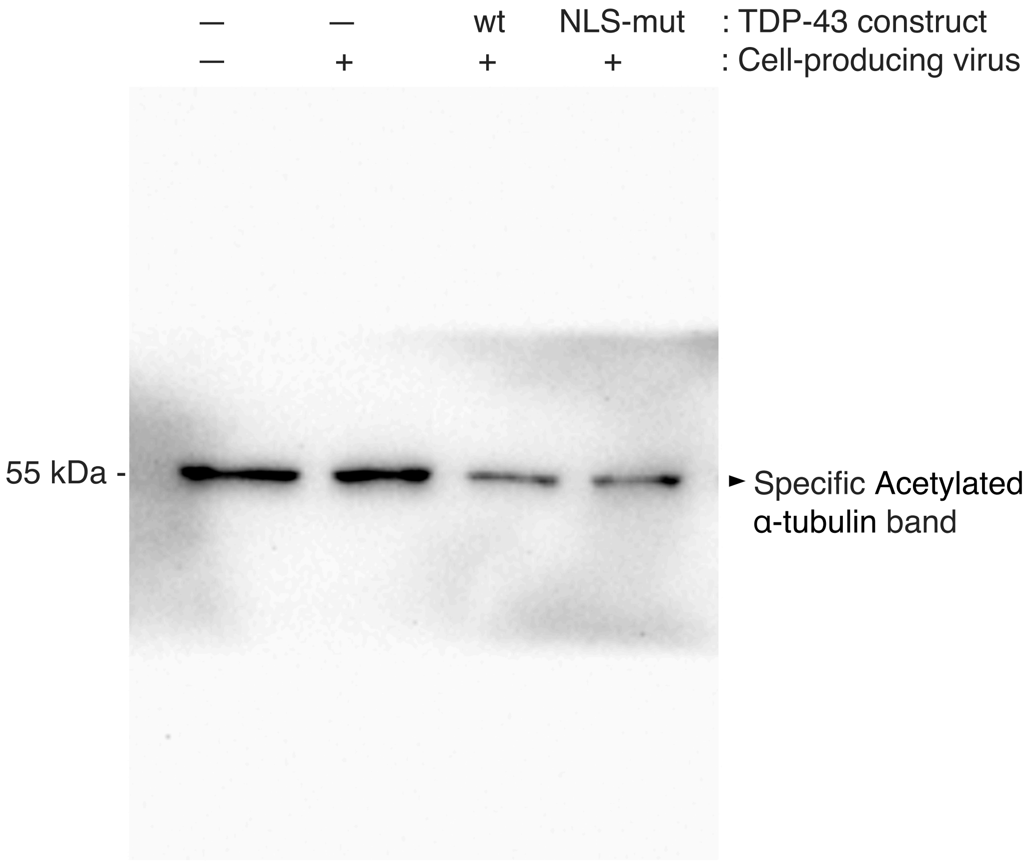

Figure S2B. Replicate 2 Total α-tubulin complete gel Western-blot associated with Figure 2B
Cabrera-Rodríguez, R., *et al.*

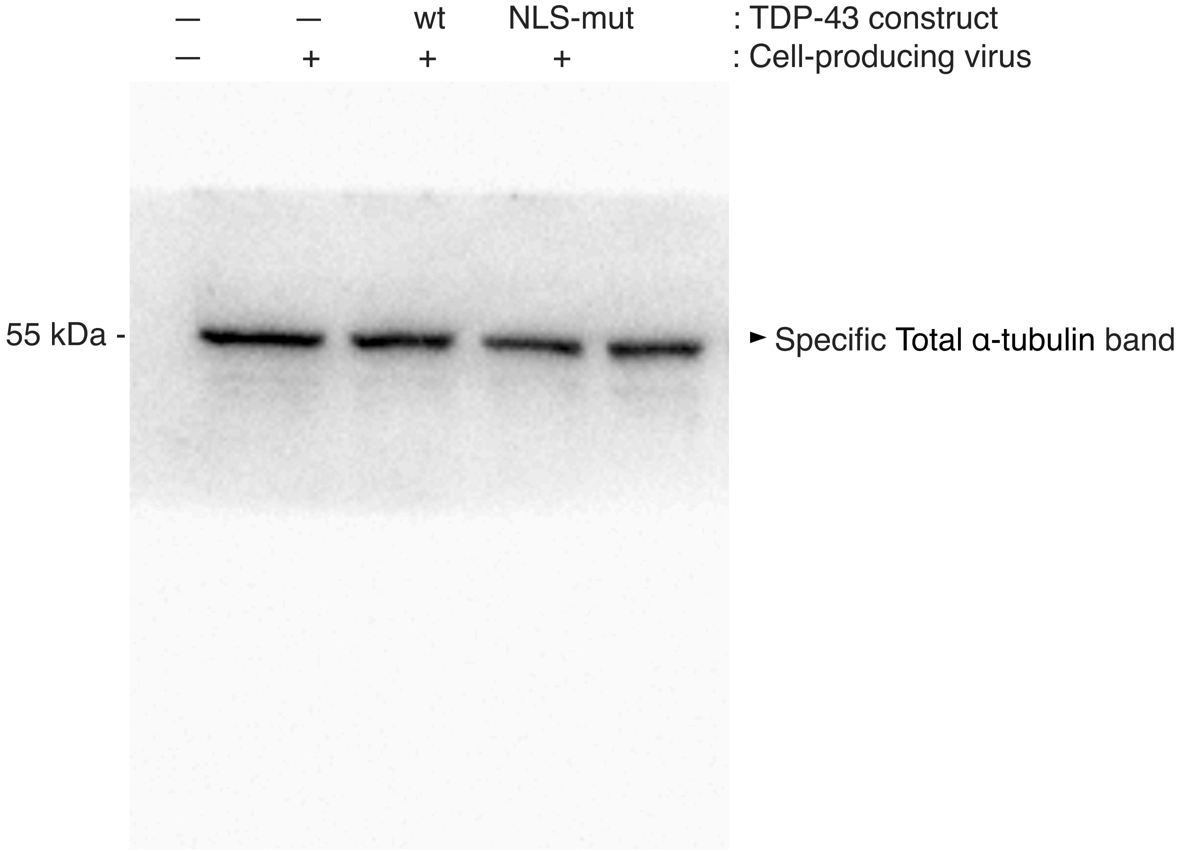

Figure S2B. Replicate 3 HDAC6 complete gel Western-blot associated with Figure 2B
Cabrera-Rodríguez, R., *et al.*

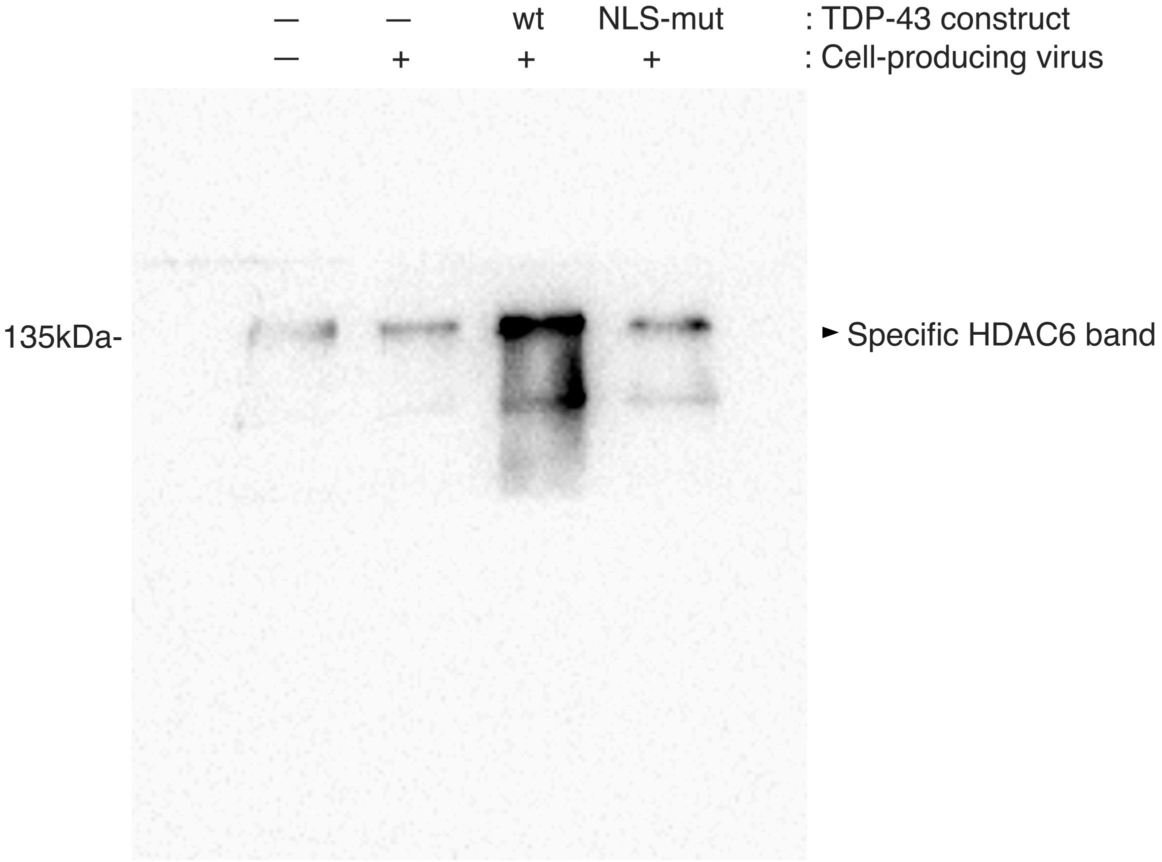

Figure S2B. Replicate 3 TDP-43 complete gel Western-blot associated with Figure 2B
Cabrera-Rodríguez, R., *et al.*

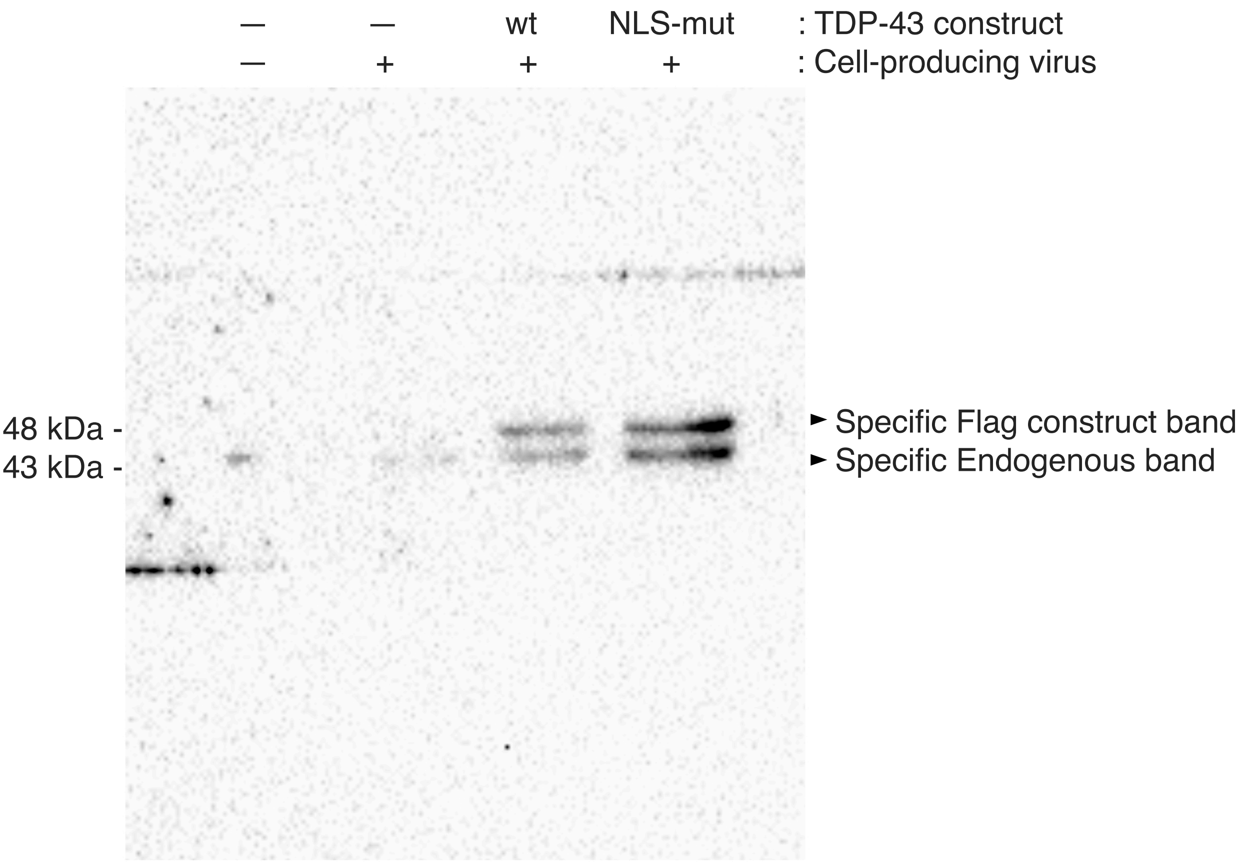

Figure S2B. Replicate 3 $Pr55^{Gag}$ complete gel   Western-blot associated with Figure 2B
Cabrera-Rodríguez, R., *et al.*

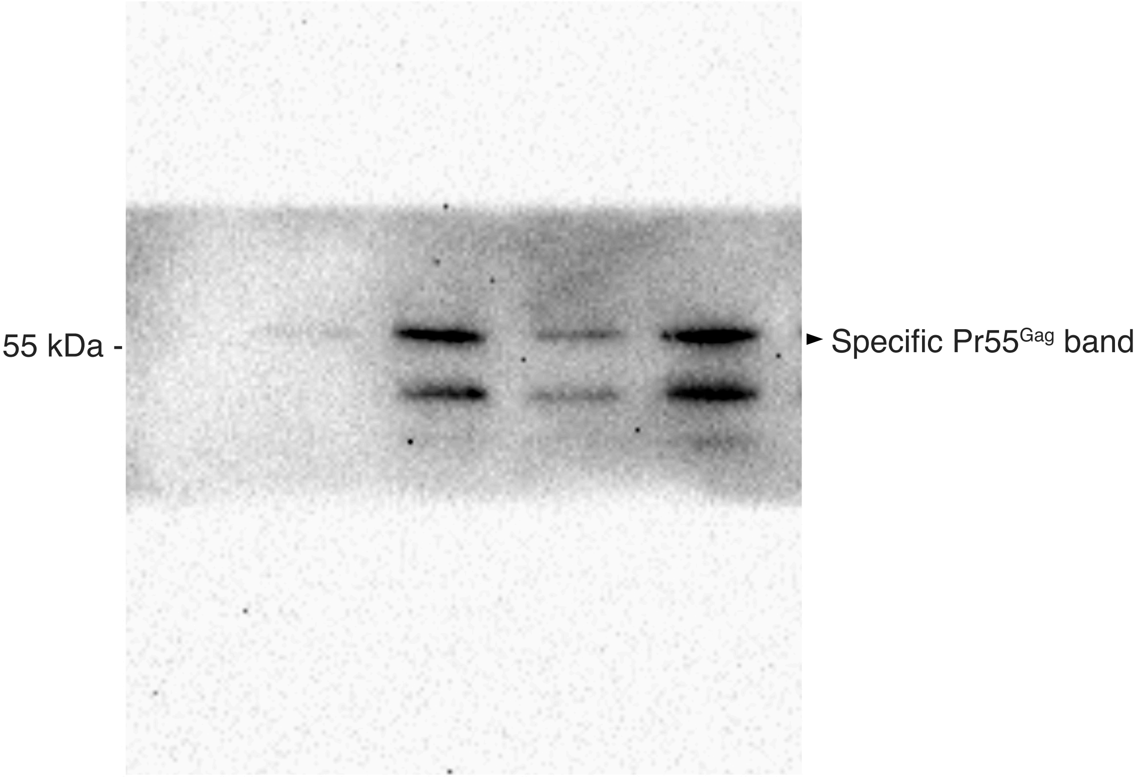

Figure S2B. Replicate 3 Vif complete gel Western-blot associated with Figure 2B

Cabrera-Rodríguez, R., *et al.*

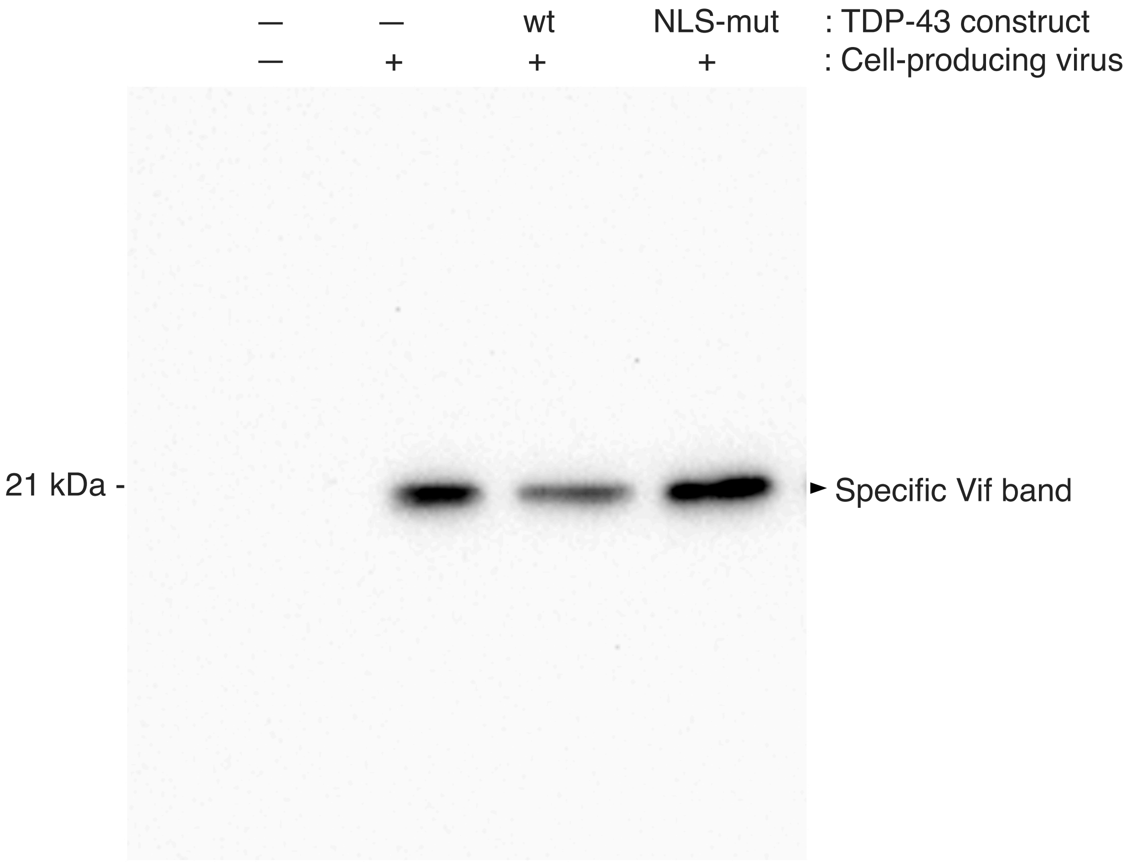

Figure S2B. Replicate 3 Acetylated α-tubulin complete gel   Western-blot associated with Figure 2B
Cabrera-Rodríguez, R., *et al.*

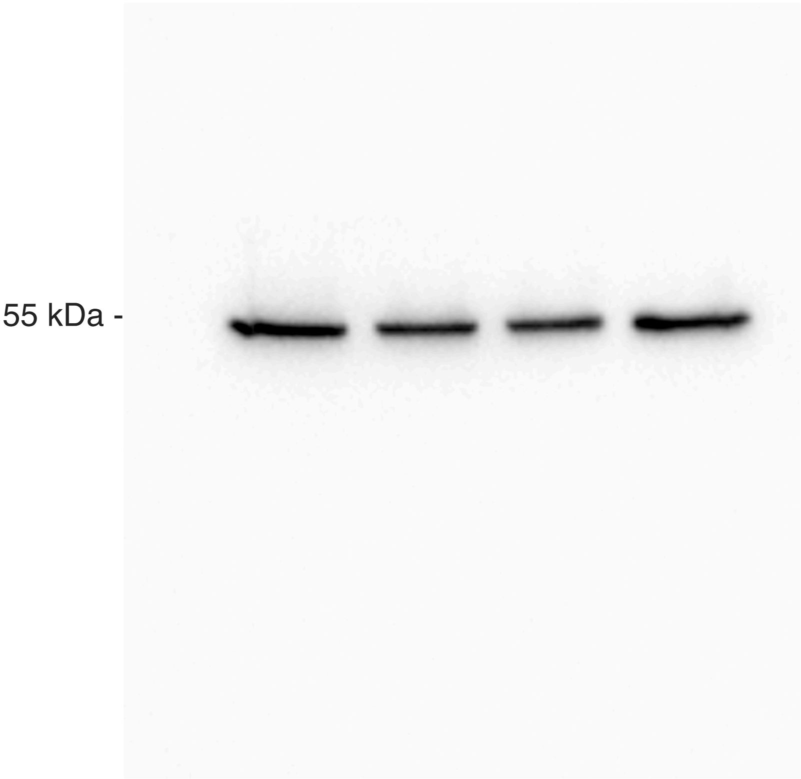

Figure S2B. Replicate 3 Total α-tubulin complete gel

Western-blot associated with Figure 2B
Cabrera-Rodríguez, R., *et al.*

—	—	wt	NLS-mut	: TDP-43 construct
—	+	+	+	: Cell-producing virus

55 kDa -

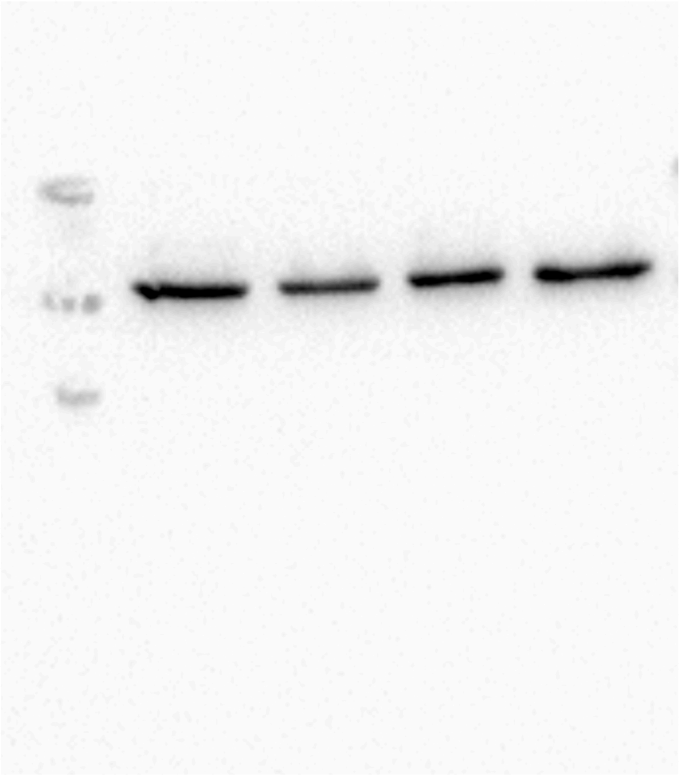

► Specific Total α-tubulin band

Replicate 1 as figure format

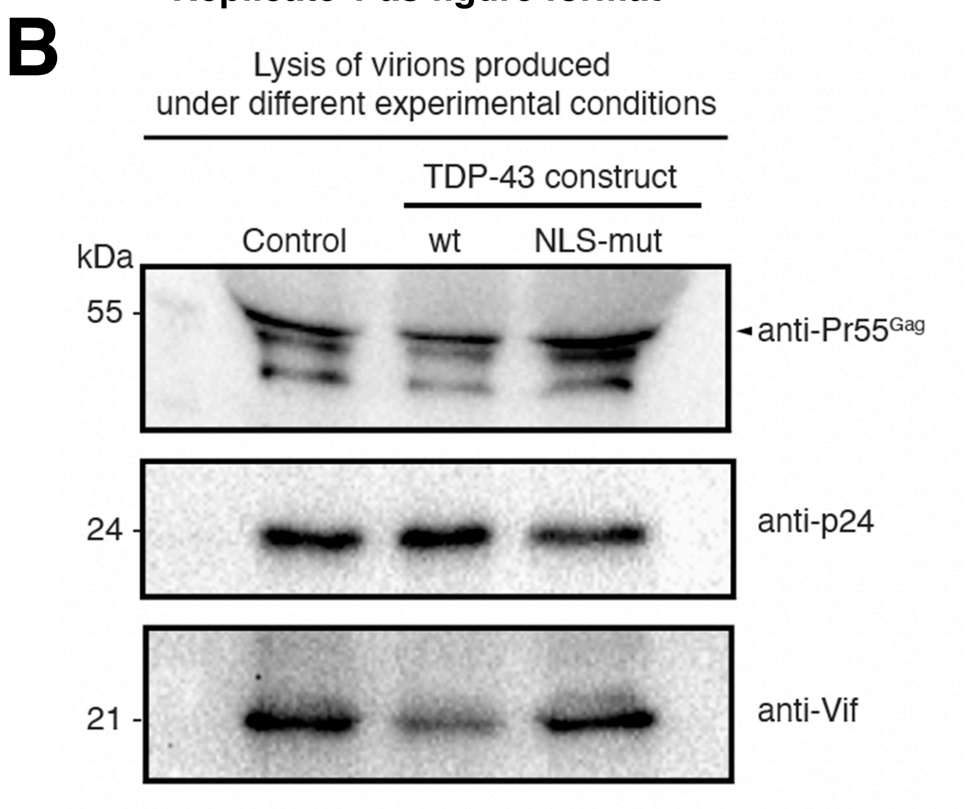

Replicate 2 as figure format

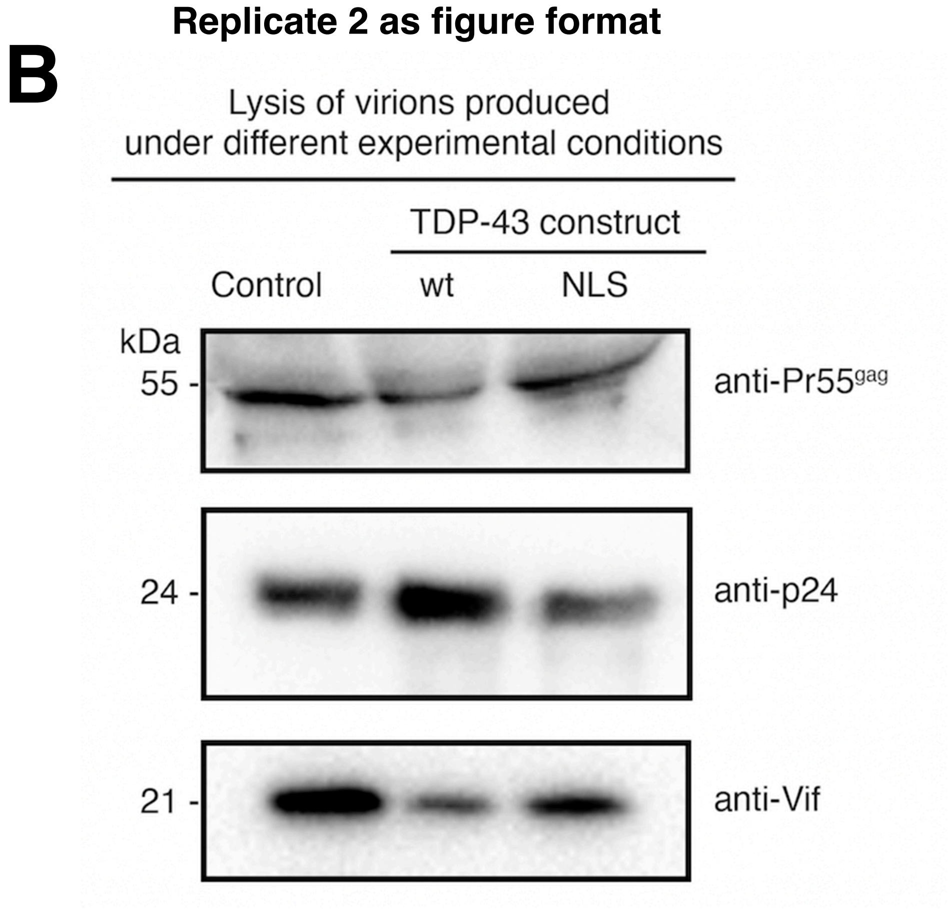

Replicate 3 as figure format

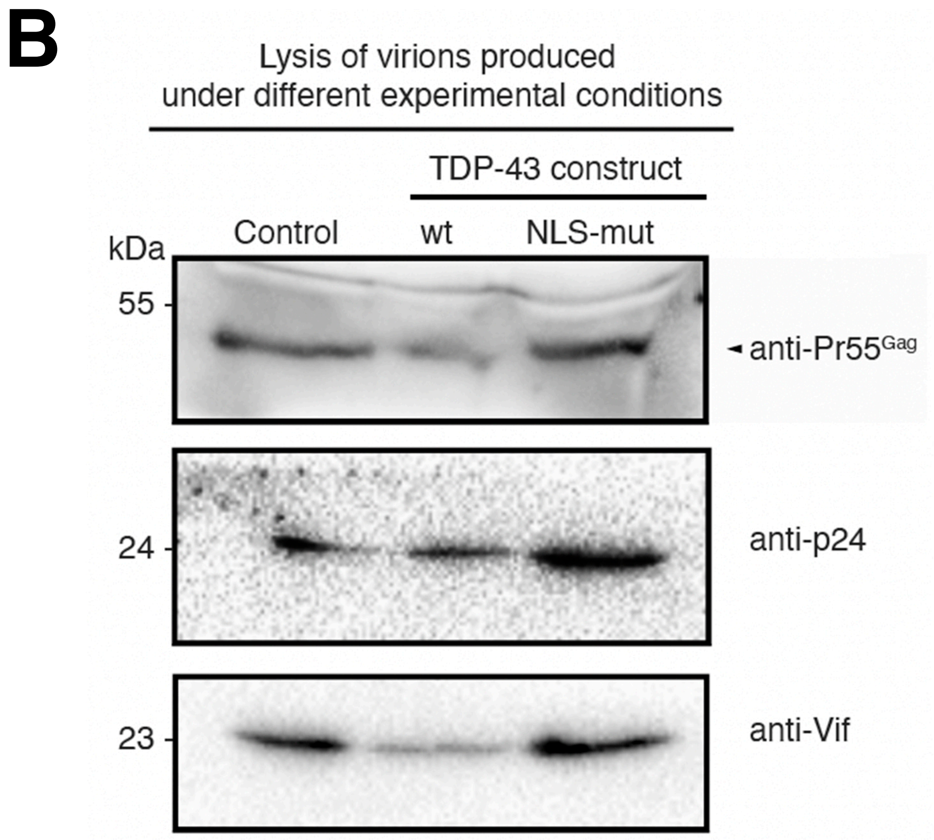

Figure S3. Replicate 1 $Pr55^{Gag}$ complete gel Western-blot associated with Figure 3B
Cabrera-Rodríguez, R., *et al.*

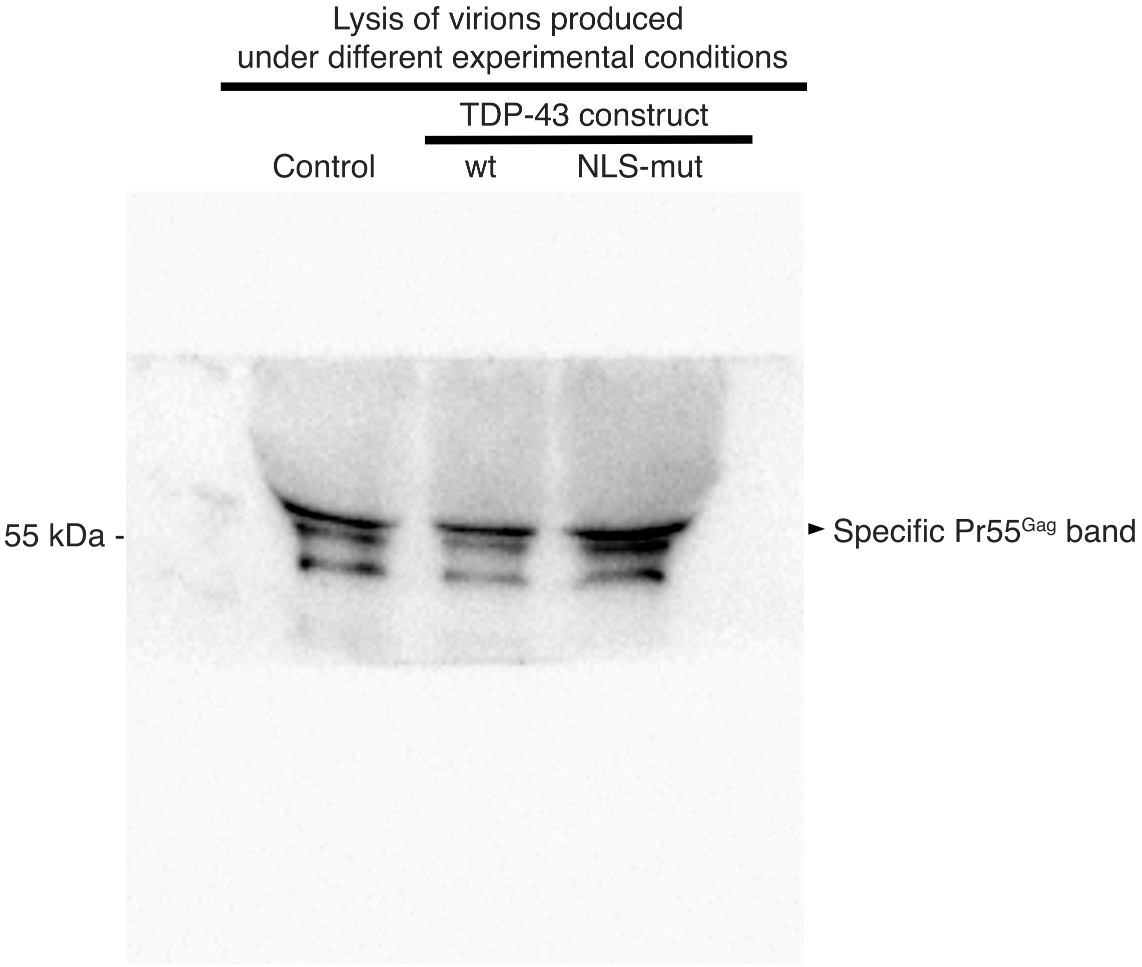

Figure S3. Replicate 1 p24 complete gel   Western-blot associated with Figure 3B
Cabrera-Rodríguez, R., *et al.*

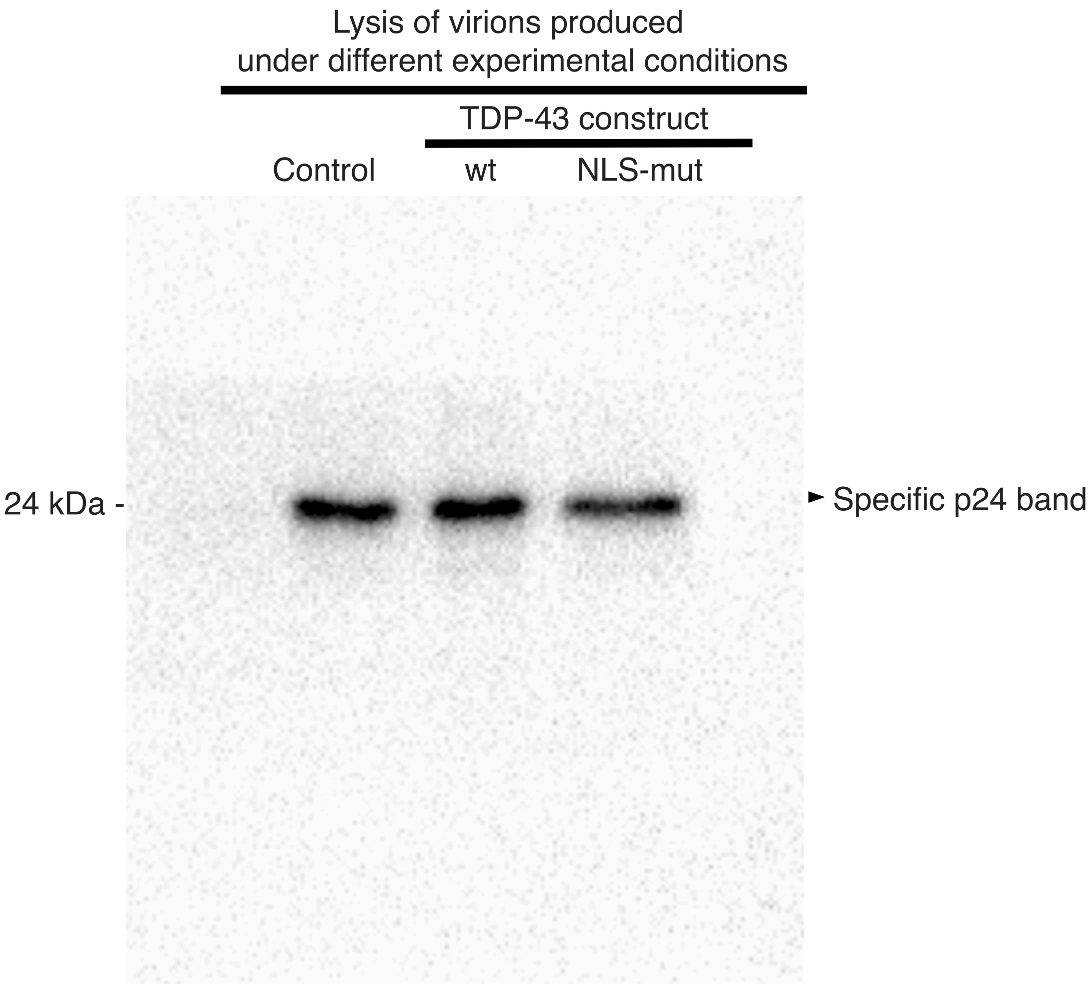

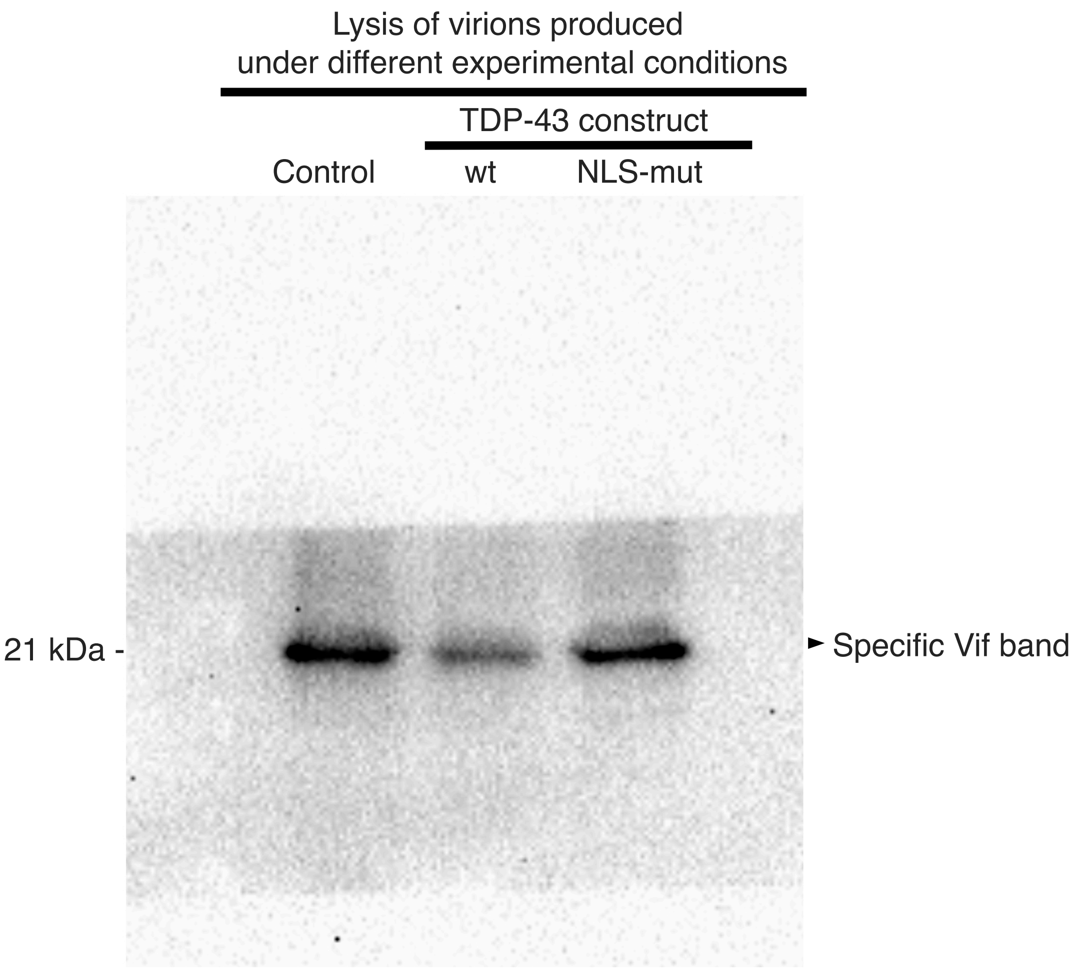
Lysis of virions produced
under different experimental conditions
TDP-43 construct
Control
wt
NLS-mut
21 kDa -
Specific Vif band

Figure S3. Replicate 2 $Pr55^{Gag}$ complete gel

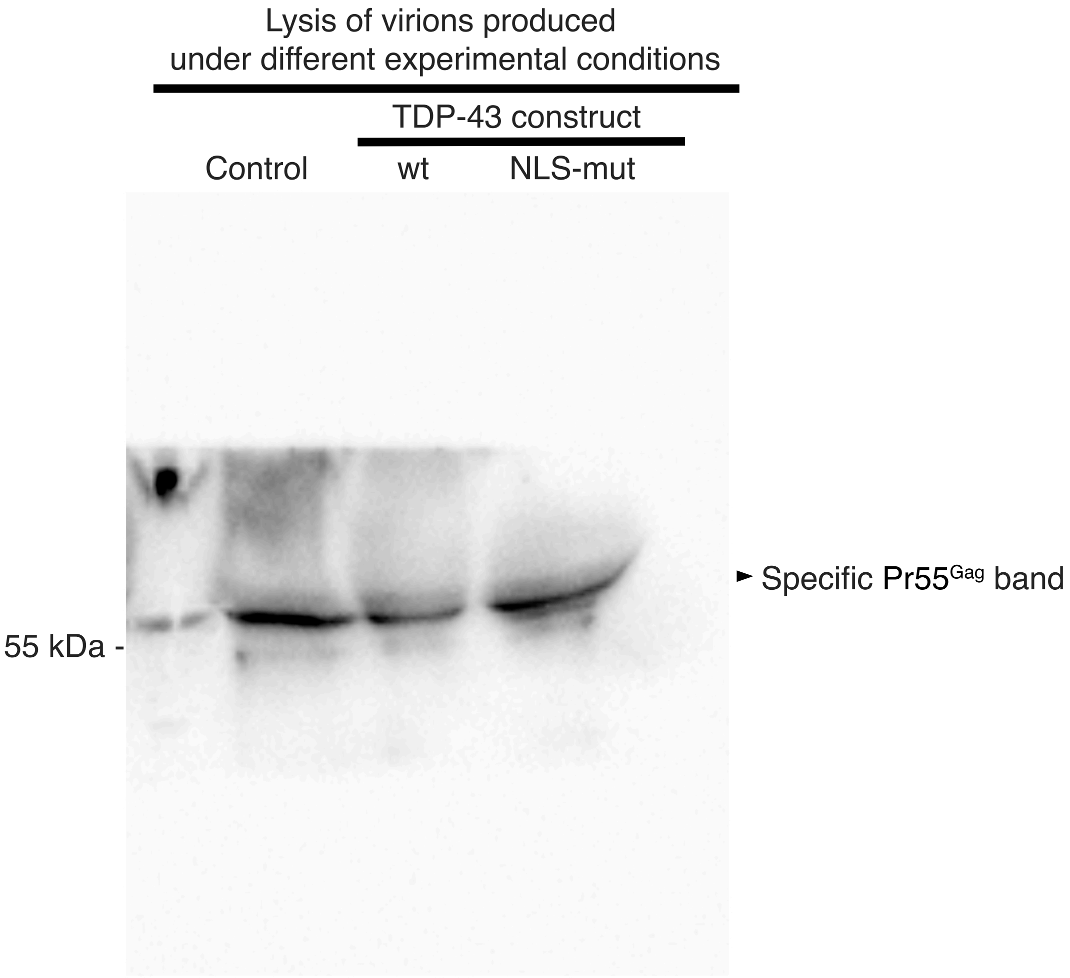

Figure S3. Replicate 2 p24 complete gel Western-blot associated with Figure 3B
Cabrera-Rodríguez, R., *et al.*

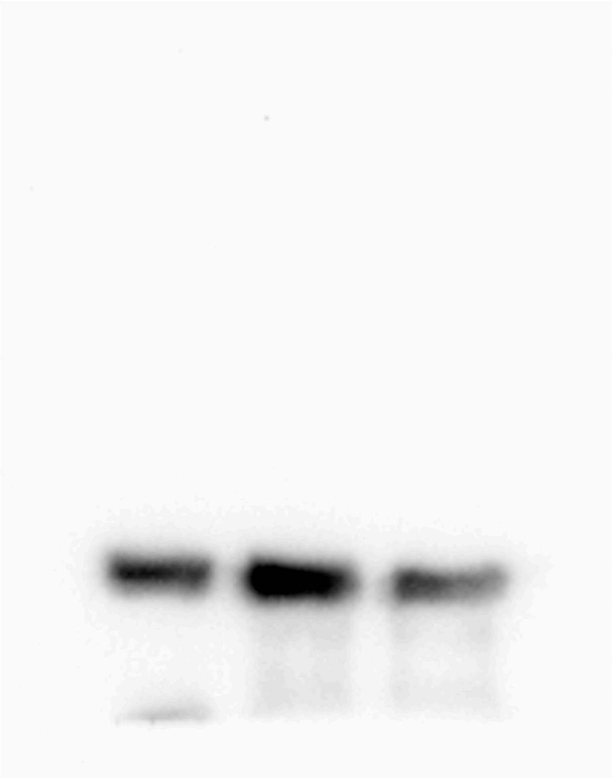

Figure S3. Replicate 2 Vif complete gel   Western-blot associated with Figure 3B
Cabrera-Rodríguez, R., *et al.*

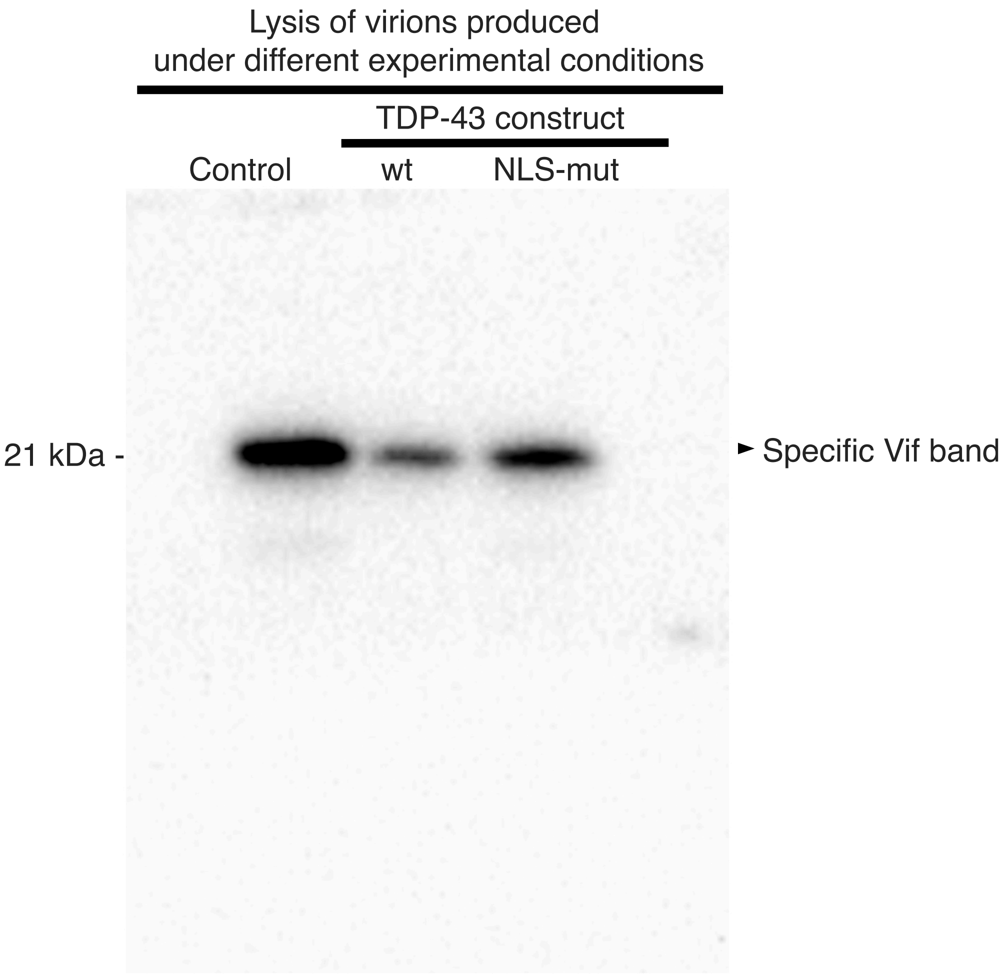

Figure S3. Replicate 3 Pr55^{Gag} complete gel Western-blot associated with Figure 3B
Cabrera-Rodríguez, R., *et al.*

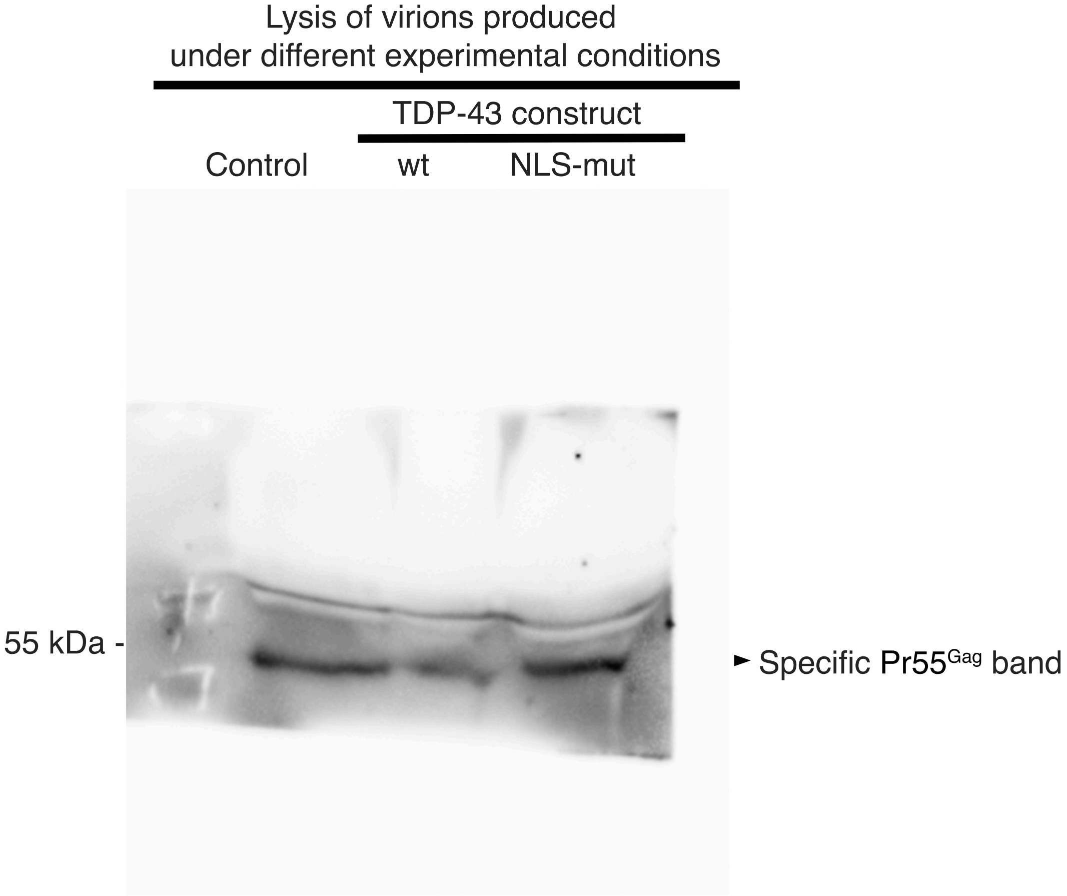

Figure S3. Replicate 3 p24 complete gel Western-blot associated with Figure 3B
Cabrera-Rodríguez, R., *et al.*

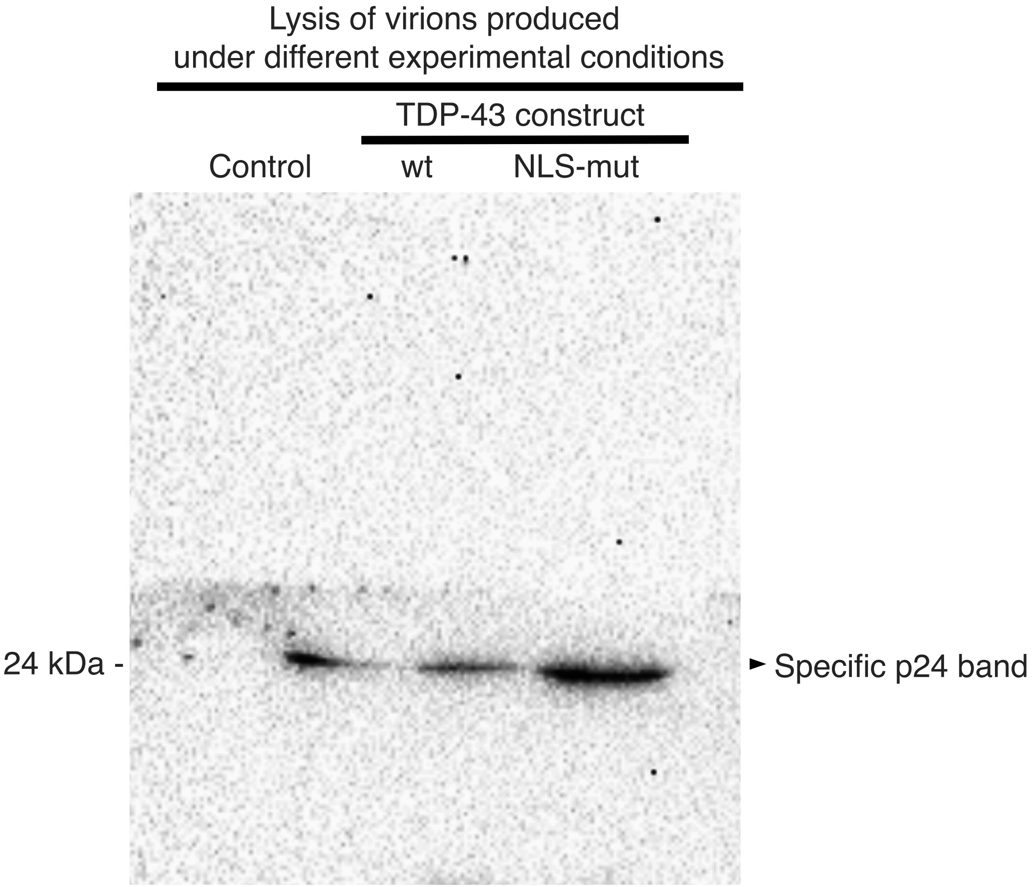

Figure S3. Replicate 3 Vif complete gel   Western-blot associated with Figure 3B
Cabrera-Rodríguez, R., *et al.*

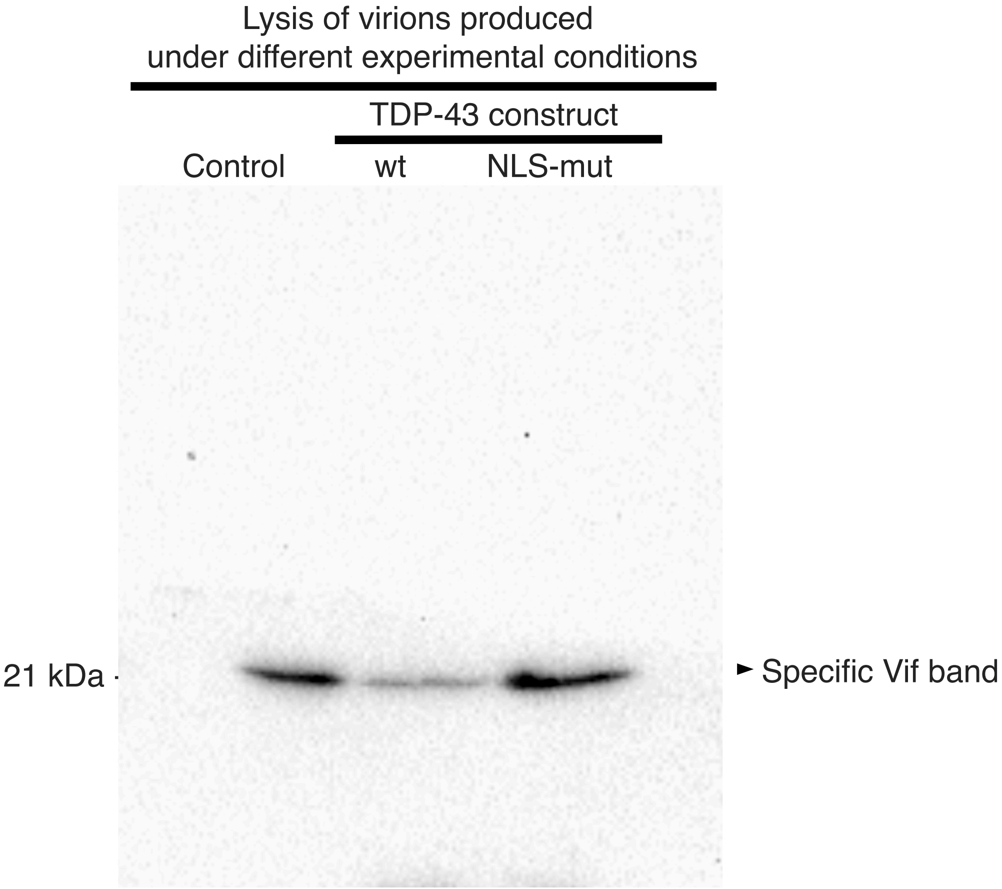

Figure S4. Western-blot replicates associated with Figure 4 Cabrera-Rodríguez R., *et al.*

Replicate 1 as figure format

A

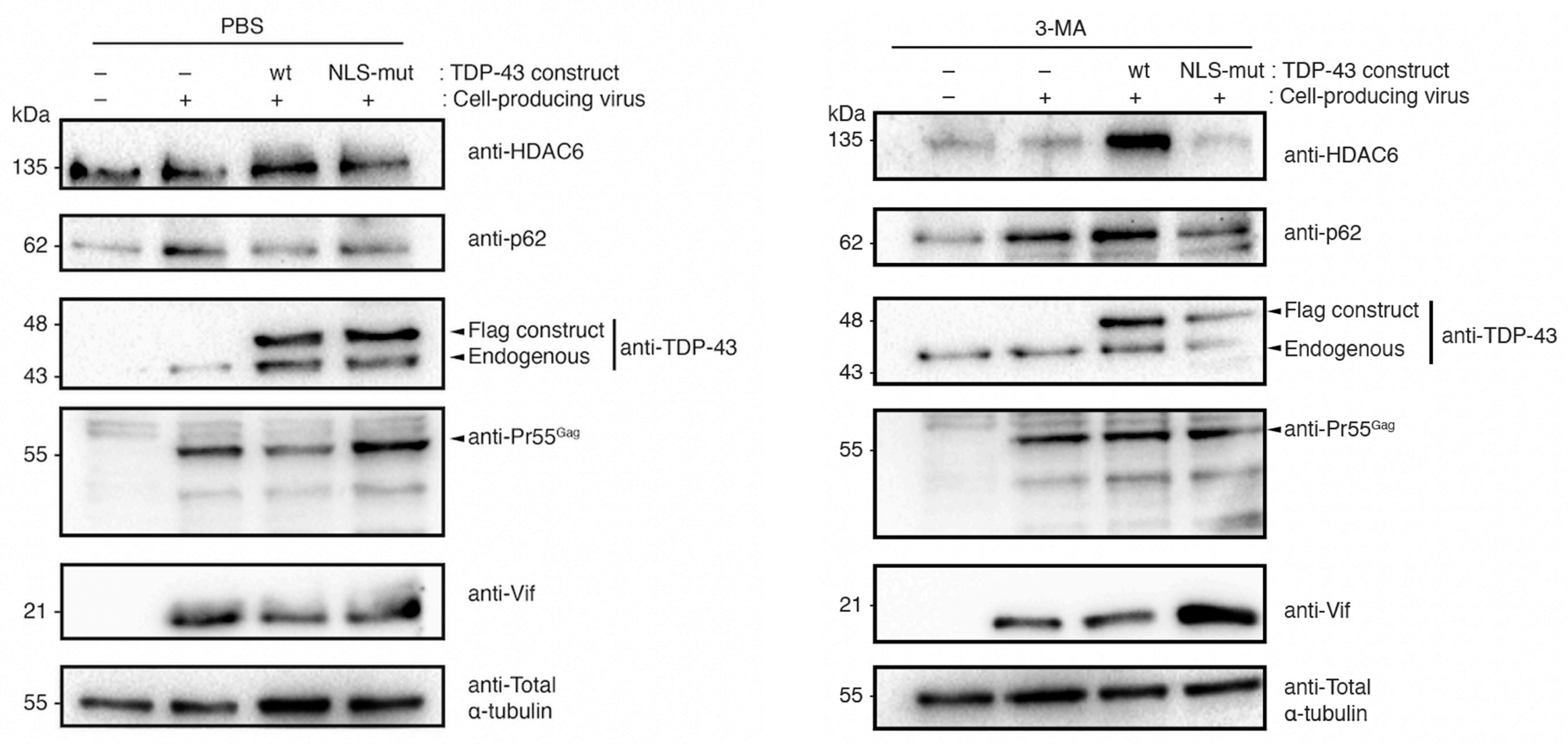

Replicate 2 as figure format

A

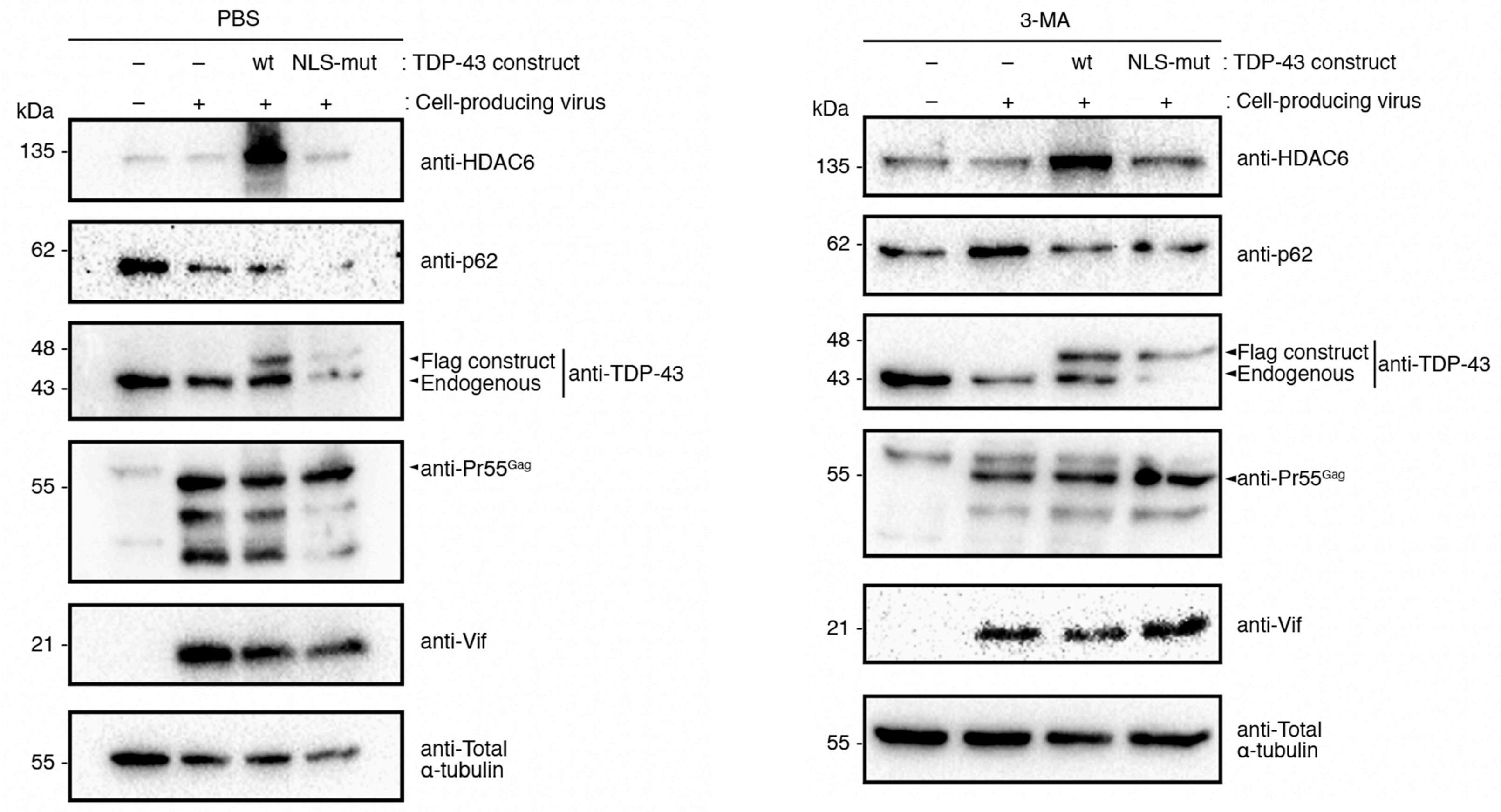

Replicate 3 as figure format

A

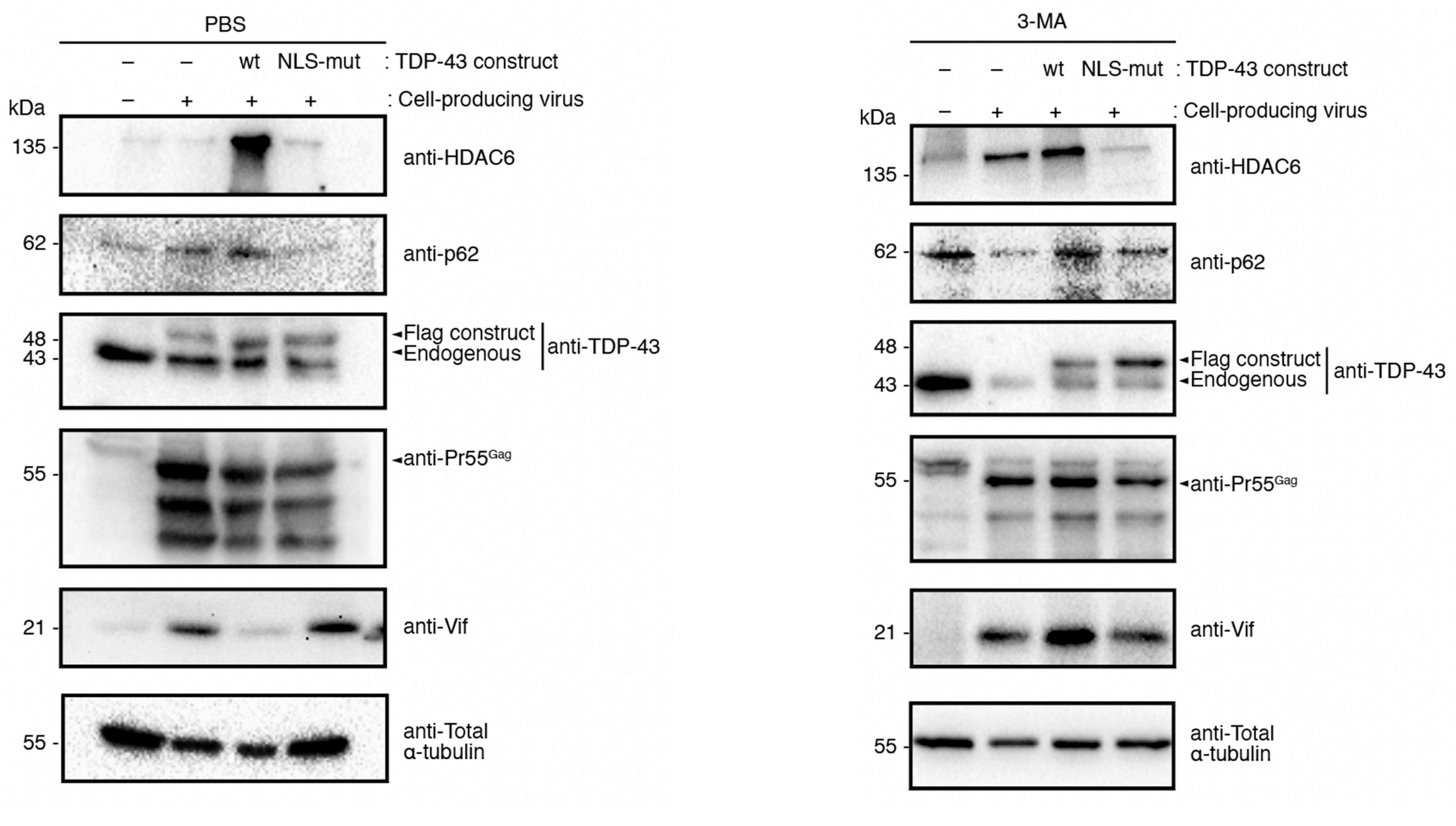

Figure S4. Replicate 1 HDAC6 complete gel Western-blot associated with Figure 4A

Cabrera-Rodríguez, R., *et al.*

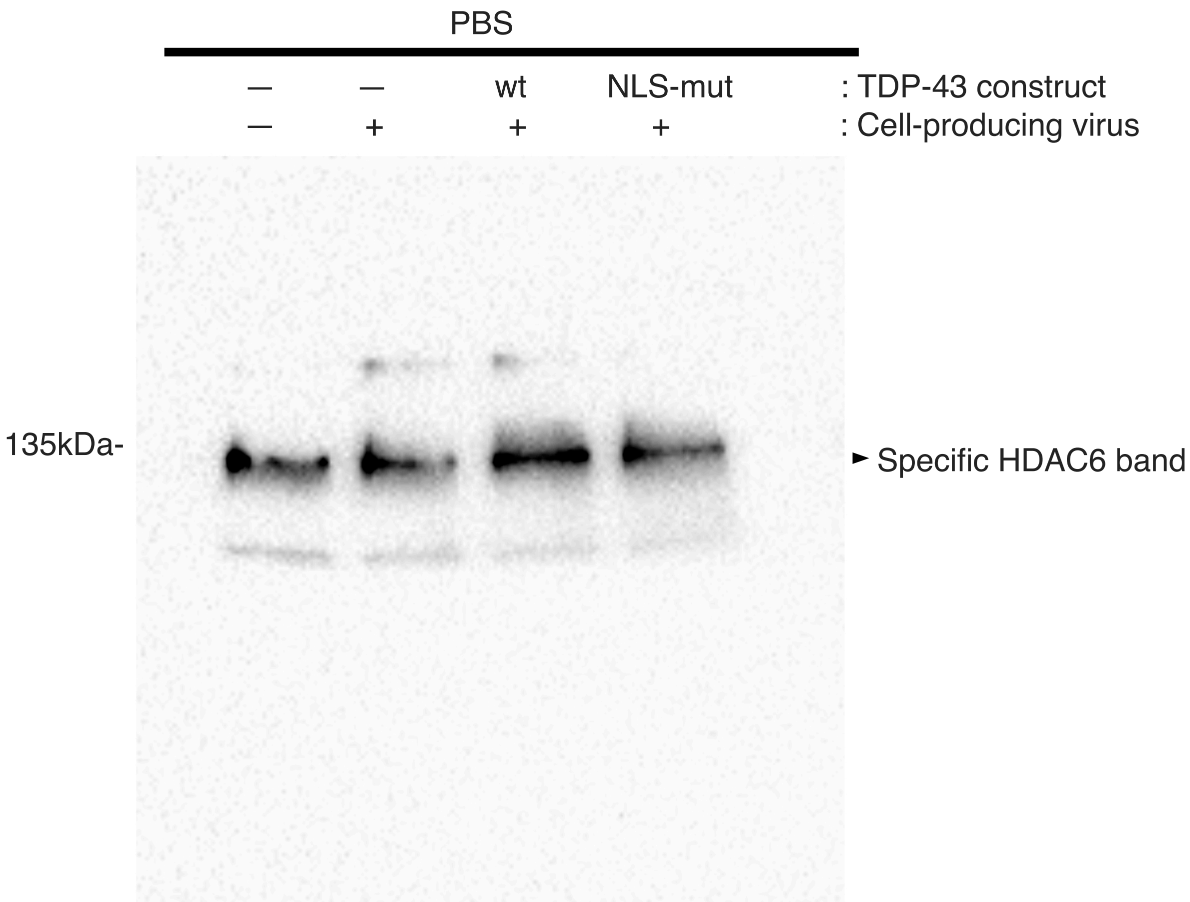

Figure S4. Replicate 1 p62 complete gel   Western-blot associated with Figure 4A
Cabrera-Rodríguez, R., *et al.*

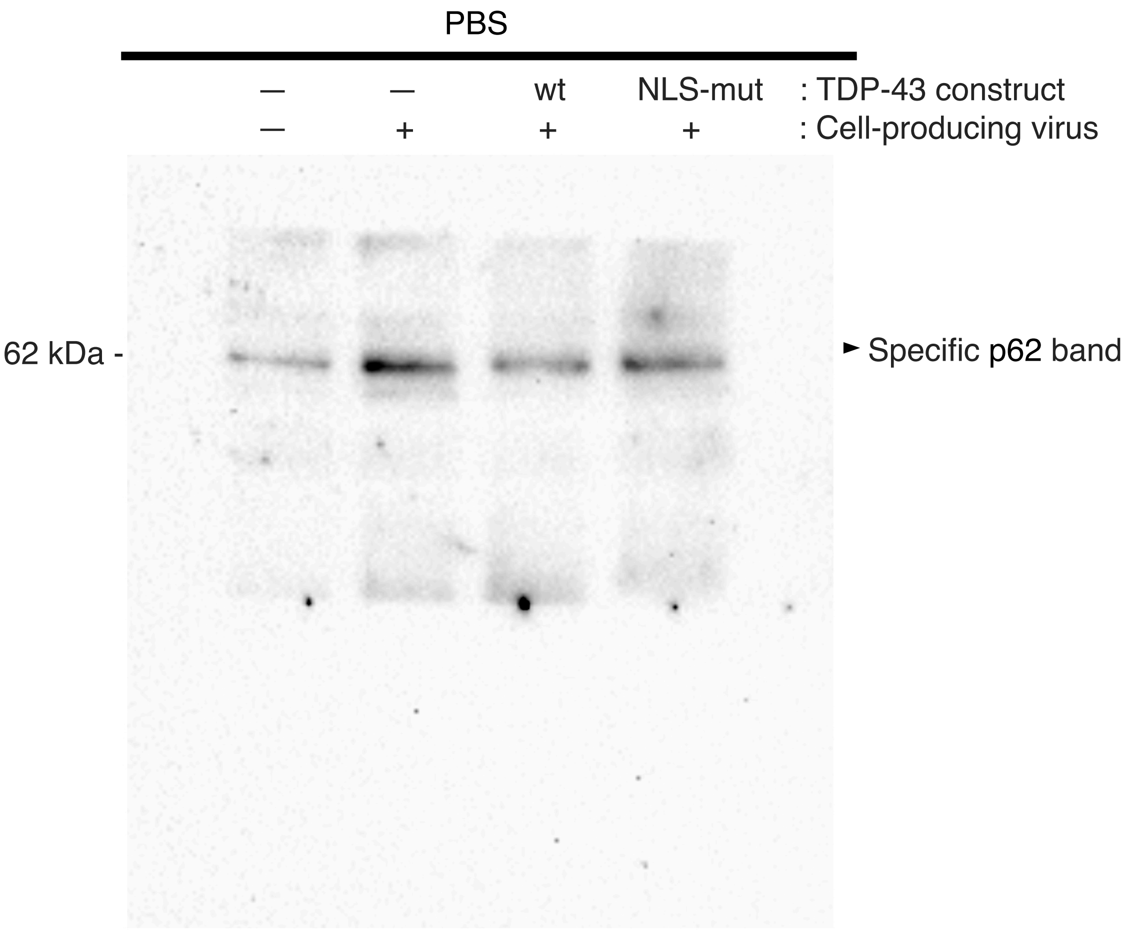

Figure S4. Replicate 1 TDP-43 complete gel Western-blot associated with Figure 4A
Cabrera-Rodríguez, R., *et al.*

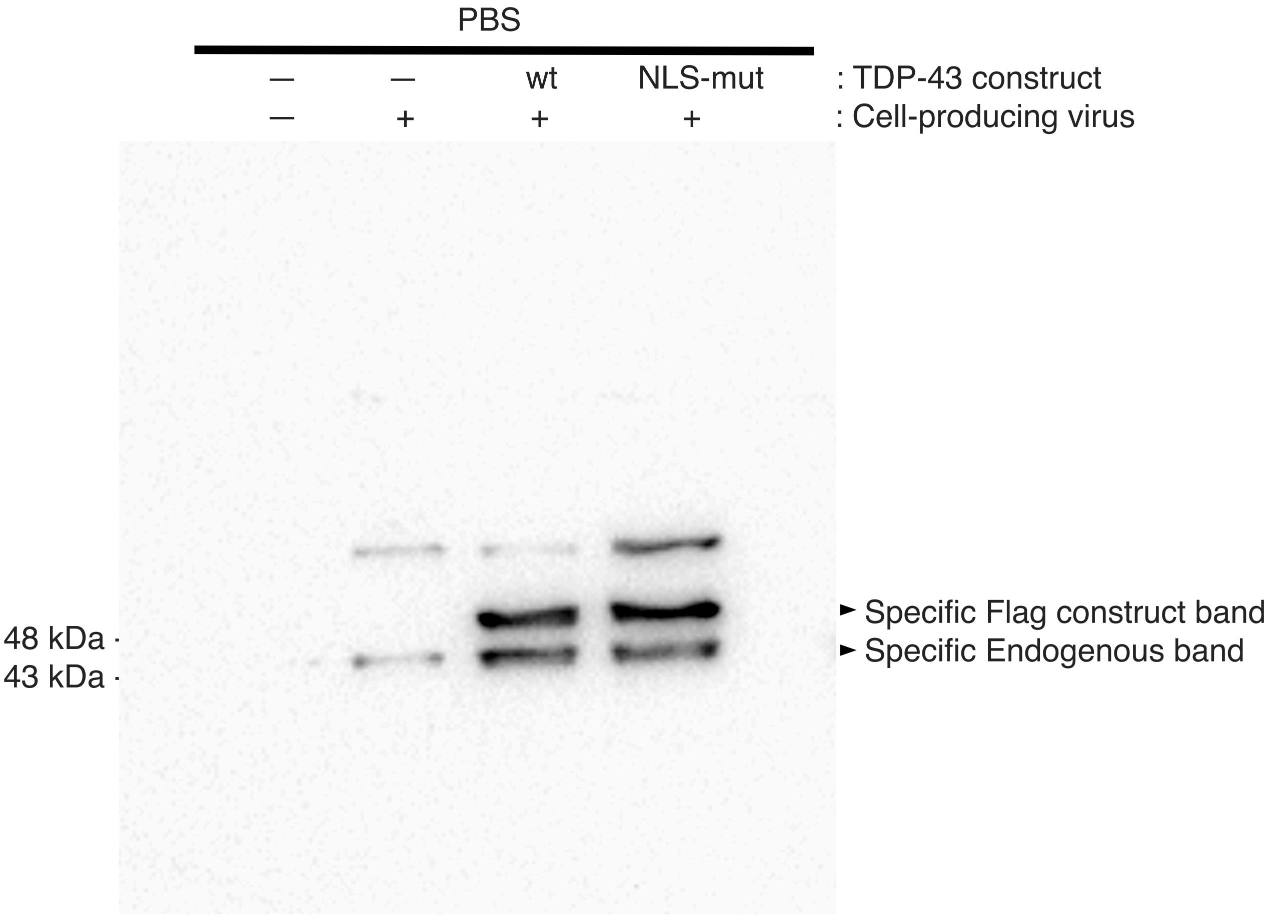

Figure S4. Replicate 1 Pr55^{Gag} complete gel   Western-blot associated with Figure 4A

Cabrera-Rodríguez, R., *et al.*

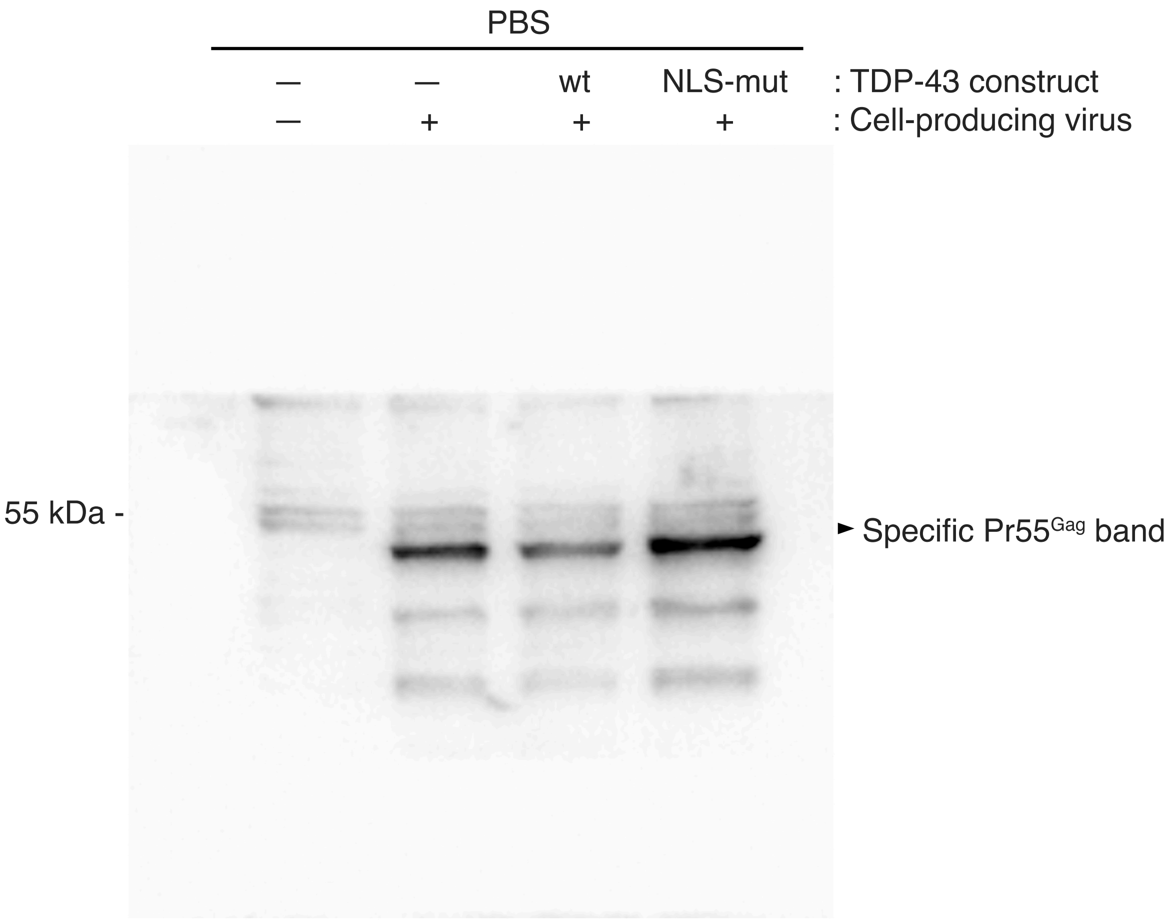

Figure S4. Replicate 1 Vif complete gel   Western-blot associated with Figure 4A

Cabrera-Rodríguez, R., *et al.*

PBS

— | — | wt | NLS-mut | : TDP-43 construct

— | + | + | + | : Cell-producing virus

21 kDa -

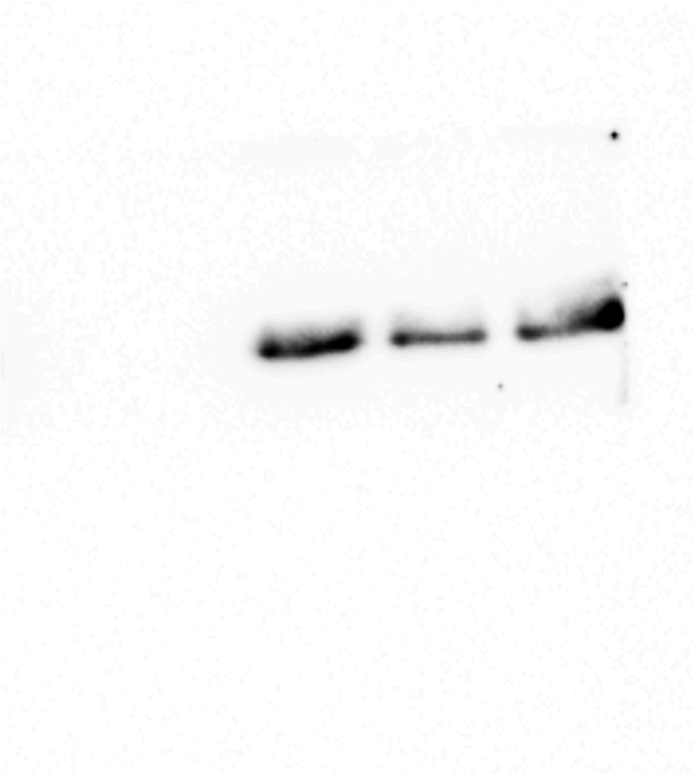

► Specific Vif band

Figure S4. Replicate 1 Total α-tubulin complete gel Western-blot associated with Figure 4A

Cabrera-Rodríguez, R., *et al.*

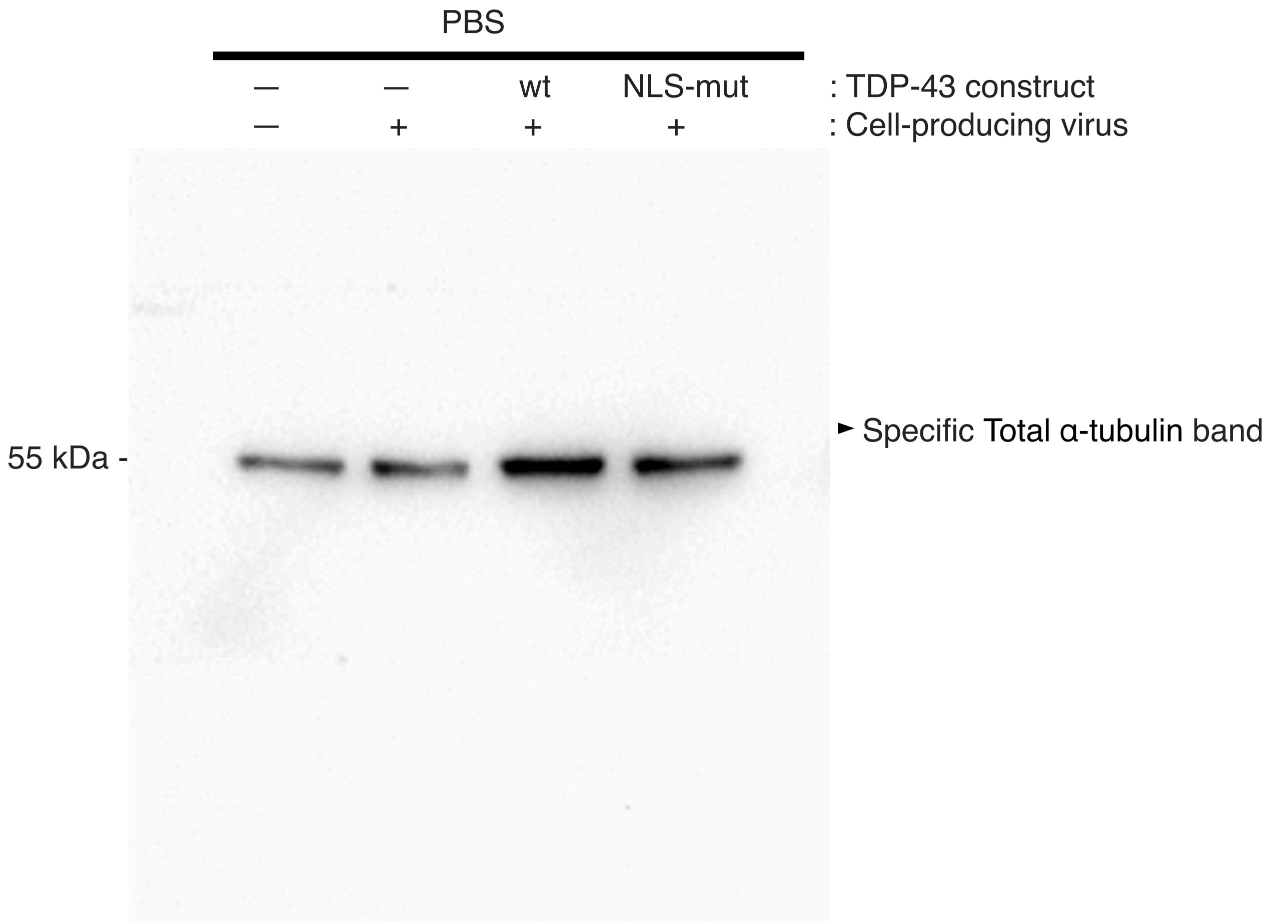

Figure S4. Replicate 1 HDAC6 complete gel Western-blot associated with Figure 4A
Cabrera-Rodríguez, R., *et al.*

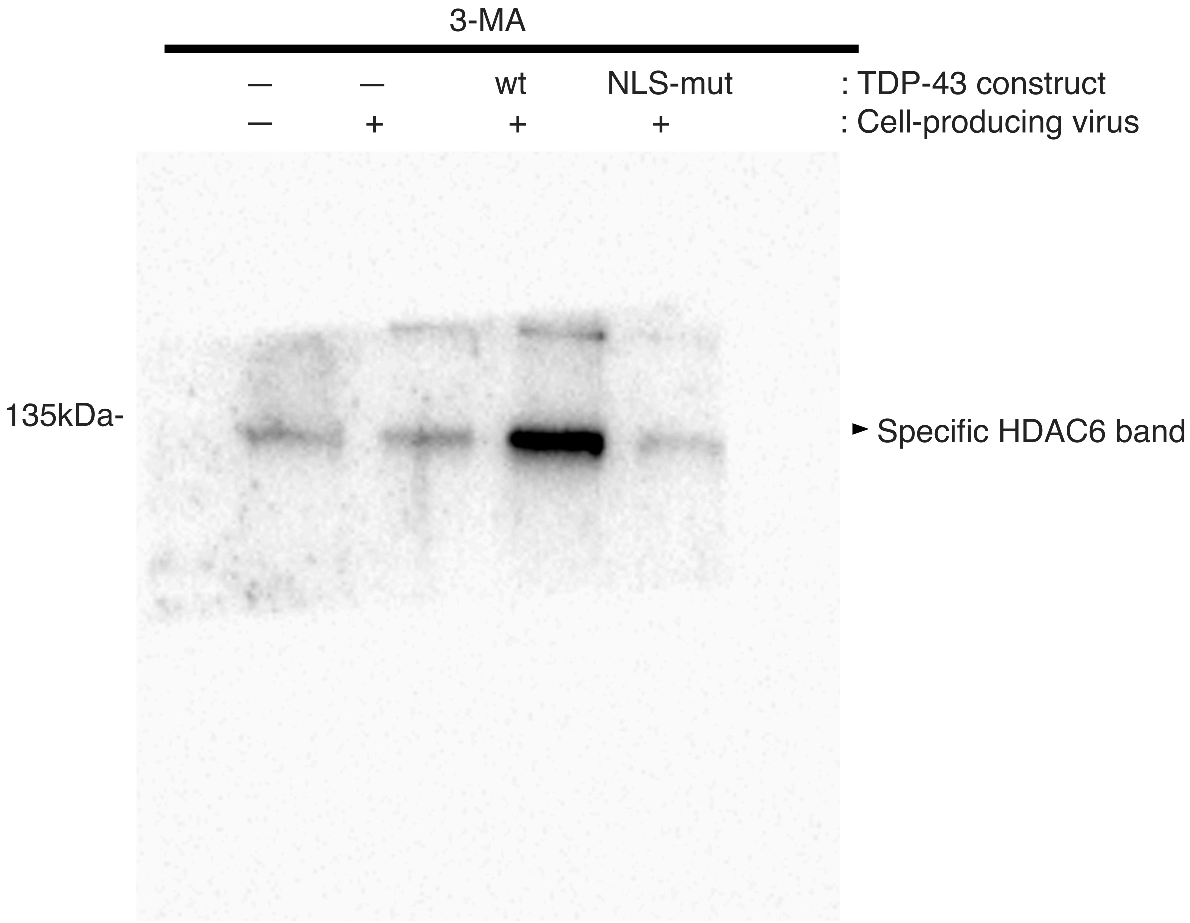

Figure S4. Replicate 1 p62 complete gel   Western-blot associated with Figure 4A
Cabrera-Rodríguez, R., *et al.*

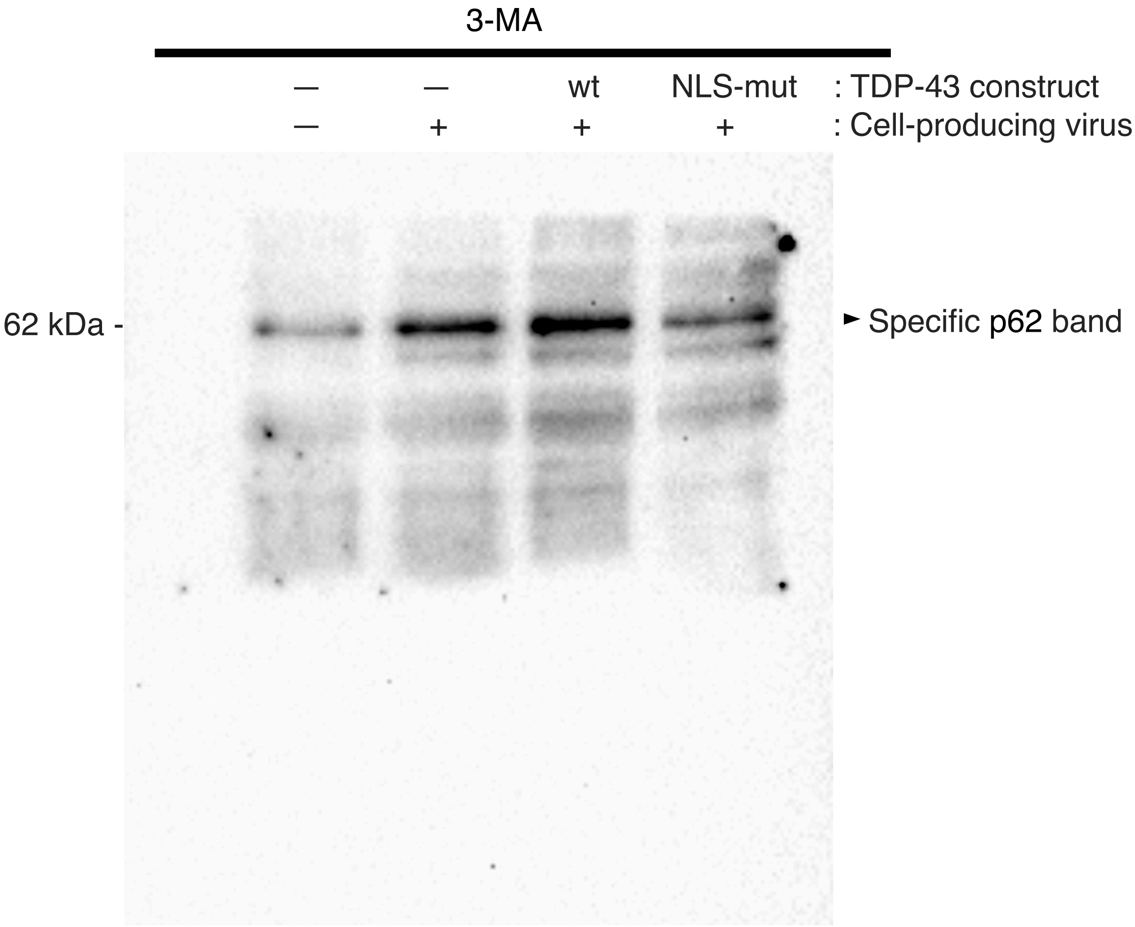

Figure S4. Replicate 1 TDP-43 complete gel   Western-blot associated with Figure 4A
Cabrera-Rodríguez, R., *et al.*

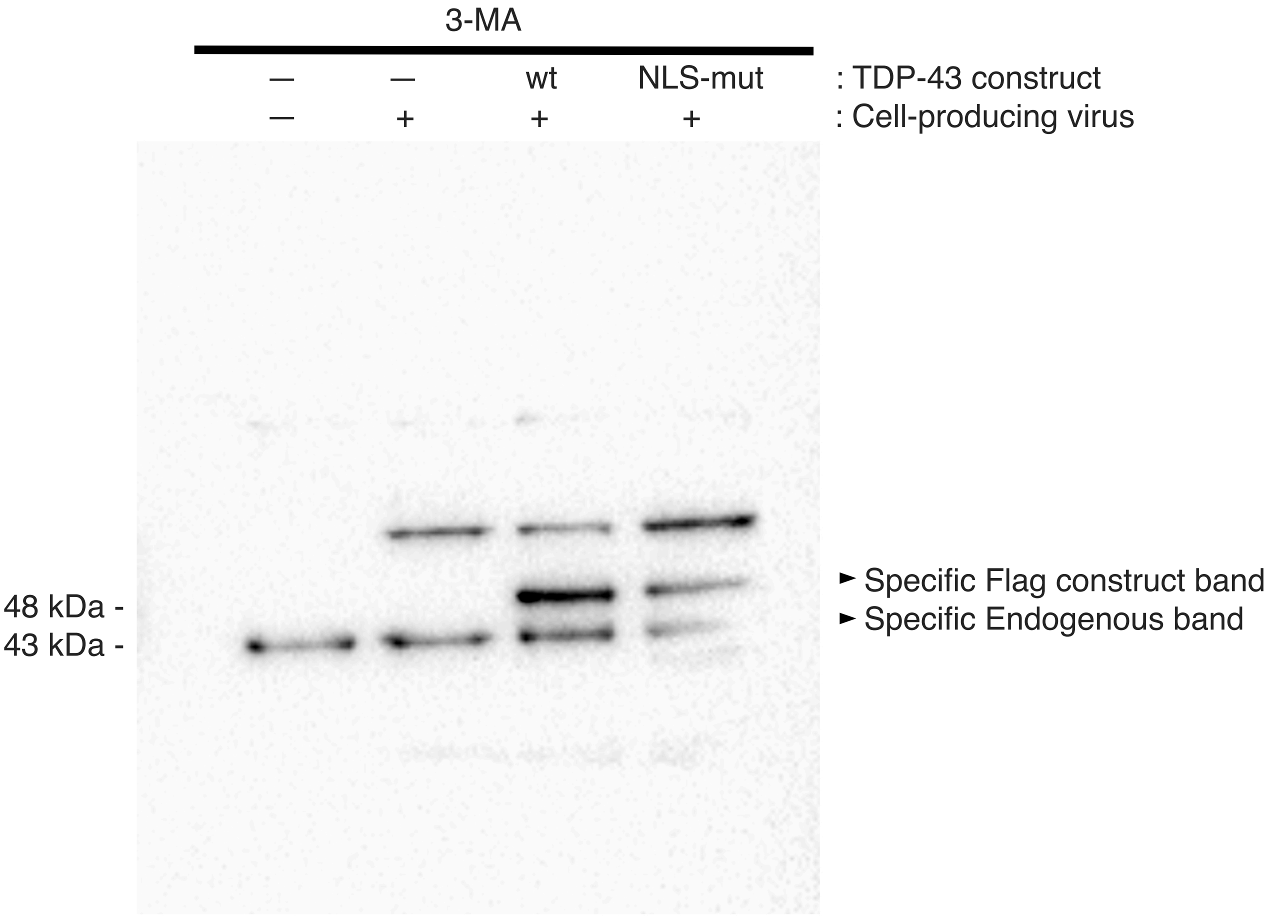

Figure S4. Replicate 1 Pr55^{Gag} complete gel Western-blot associated with Figure 4A

Cabrera-Rodríguez, R., *et al.*

3-MA

—	—	wt	NLS-mut	: TDP-43 construct
—	+	+	+	: Cell-producing virus

55 kDa -

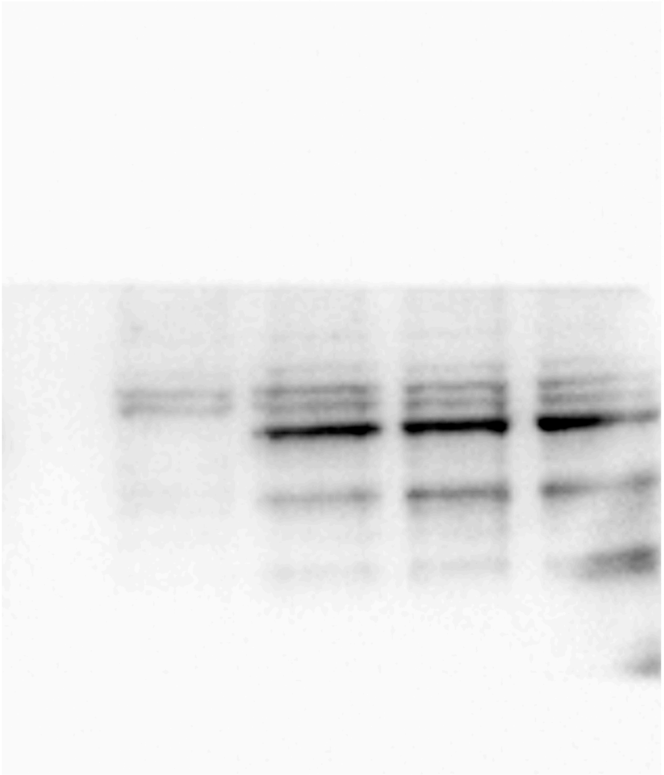

► Specific Pr55^{Gag} band

Figure S4. Replicate 1 Vif complete gel   Western-blot associated with Figure 4A
Cabrera-Rodríguez, R., *et al.*

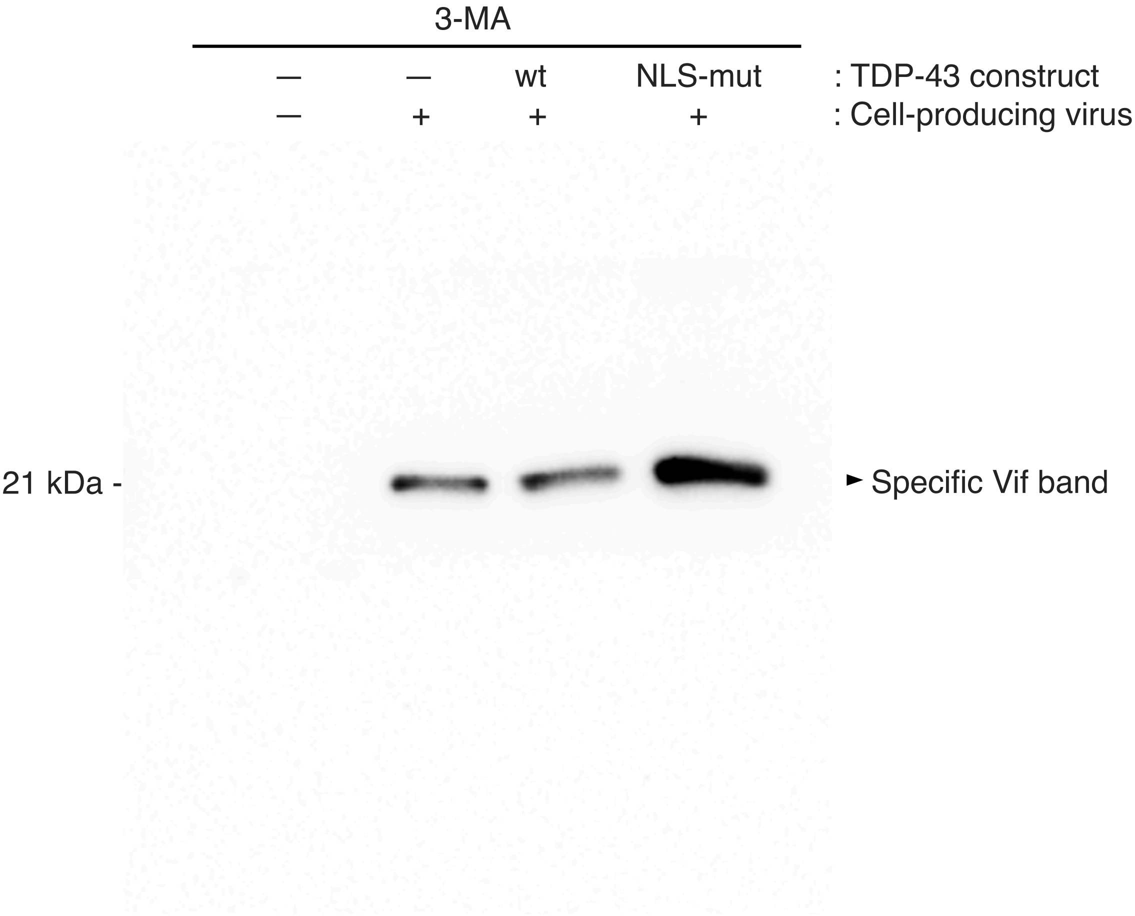

Figure S4. Replicate 1 Total α-tubulin complete gel Western-blot associated with Figure 4A

Cabrera-Rodríguez, R., *et al.*

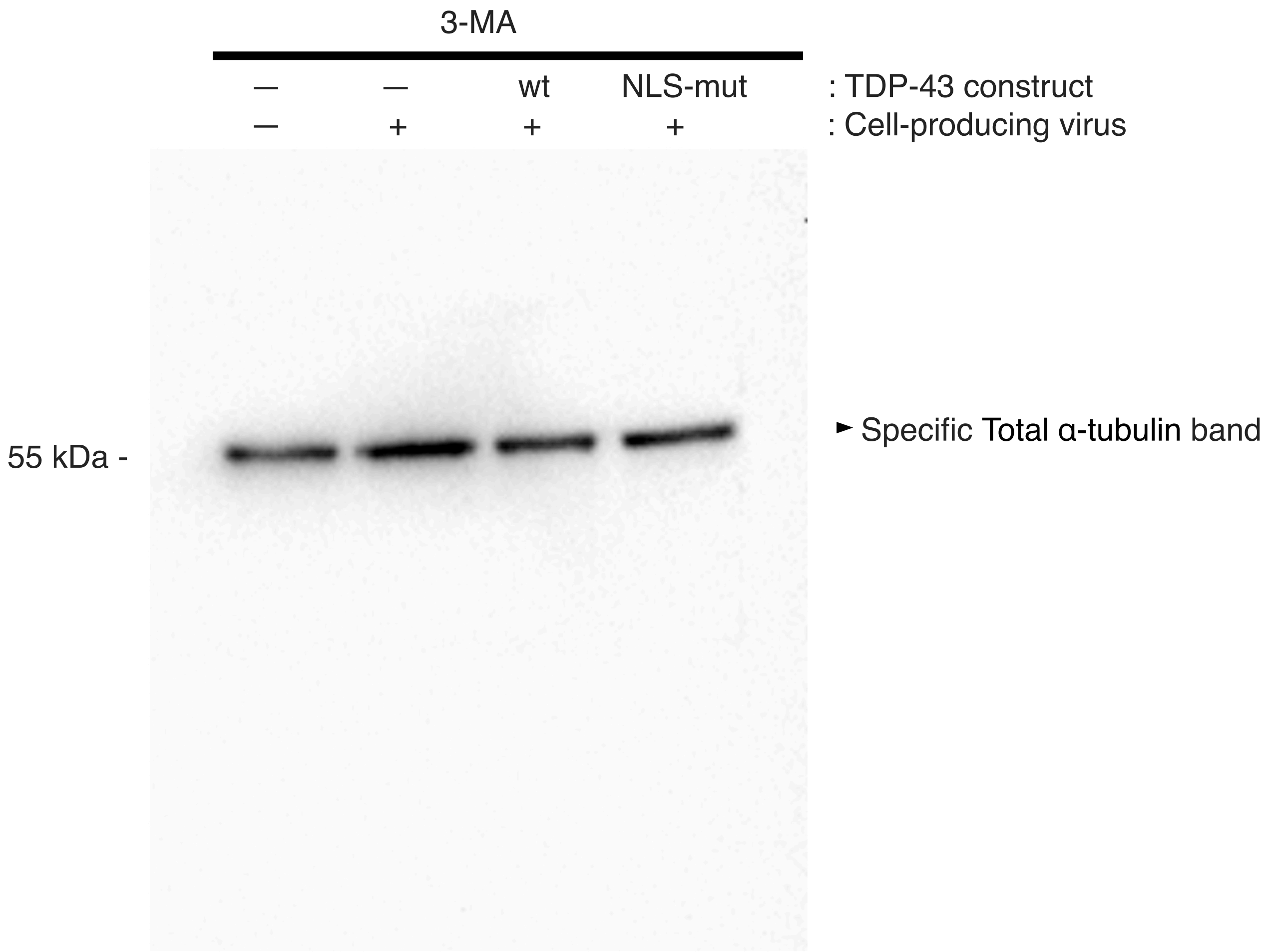

Figure S4. Replicate 2 HDAC6 complete gel   Western-blot associated with Figure 4A
Cabrera-Rodríguez, R., *et al.*

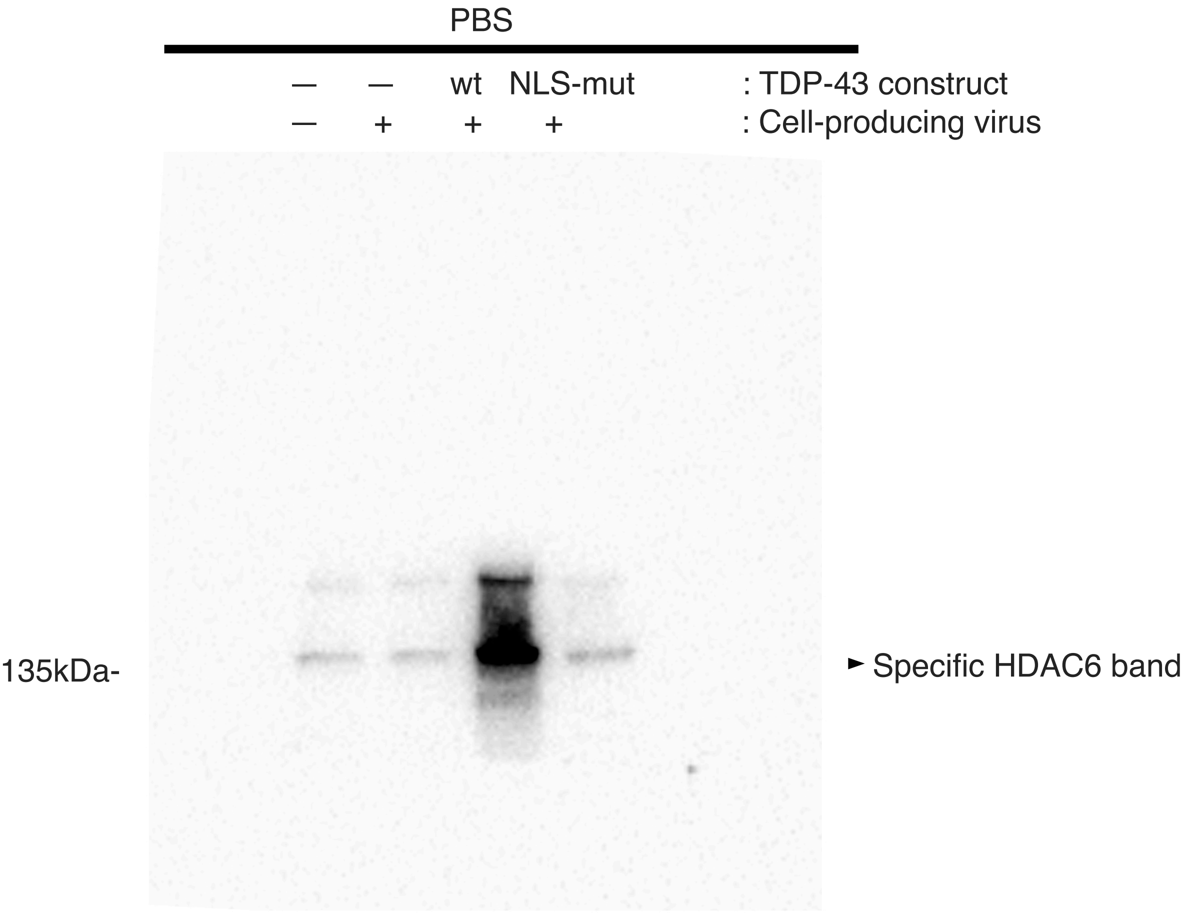

Figure S4. Replicate 2 p62 complete gel Western-blot associated with Figure 4A
Cabrera-Rodríguez, R., *et al.*

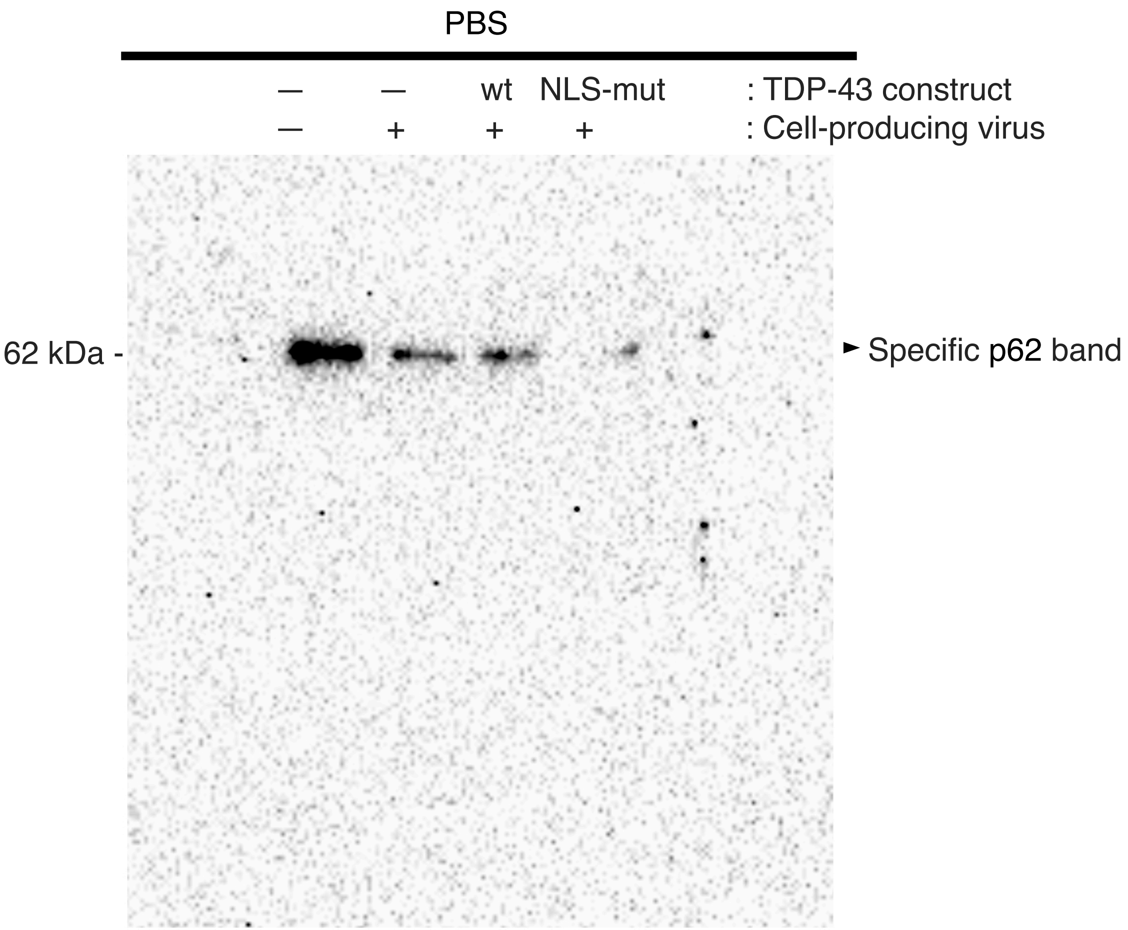

Figure S4. Replicate 2 TDP-43 complete gel   Western-blot associated with Figure 4A
Cabrera-Rodríguez, R., *et al.*

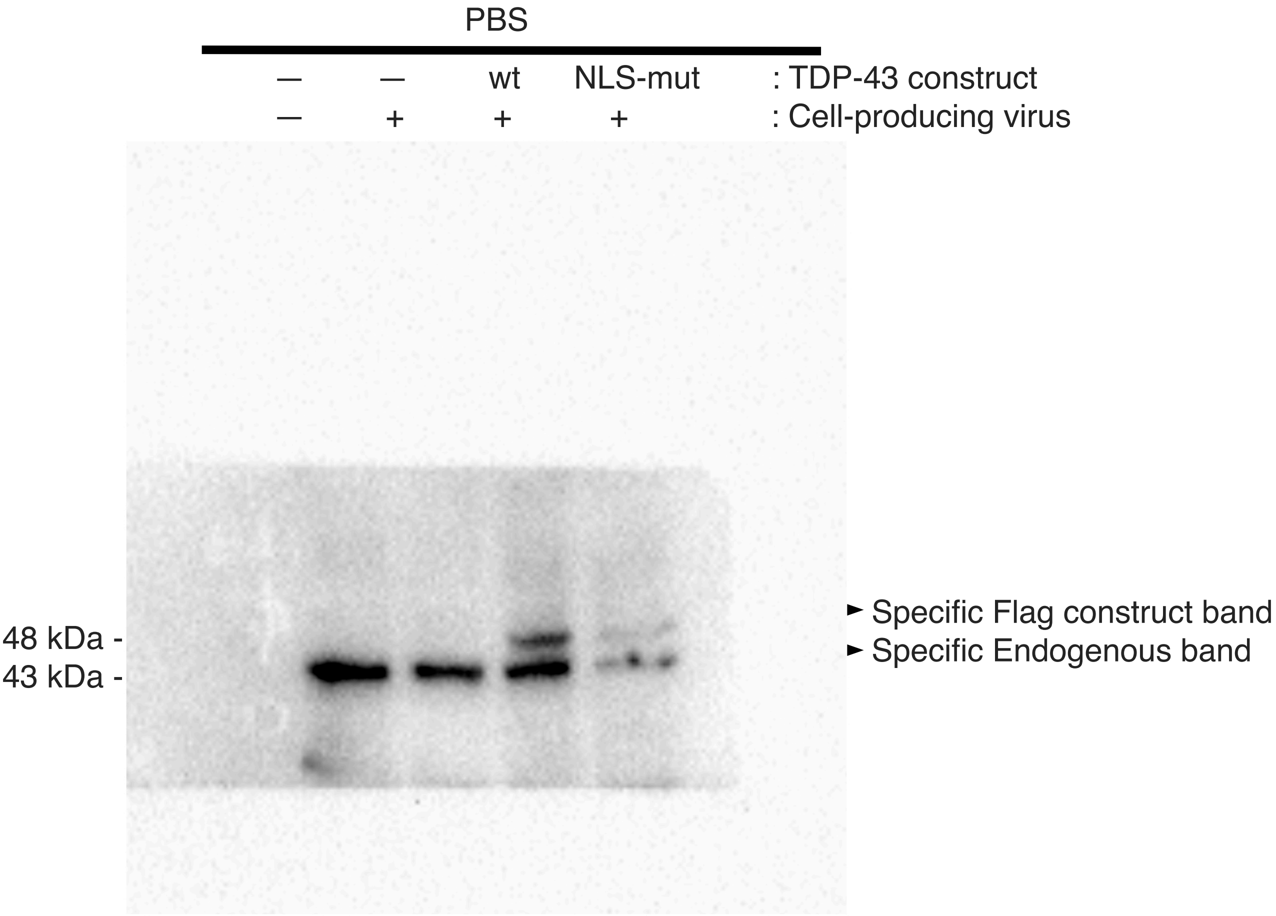

Figure S4. Replicate 2 $Pr55^{Gag}$ complete gel Western-blot associated with Figure 4A

Cabrera-Rodríguez, R., *et al.*

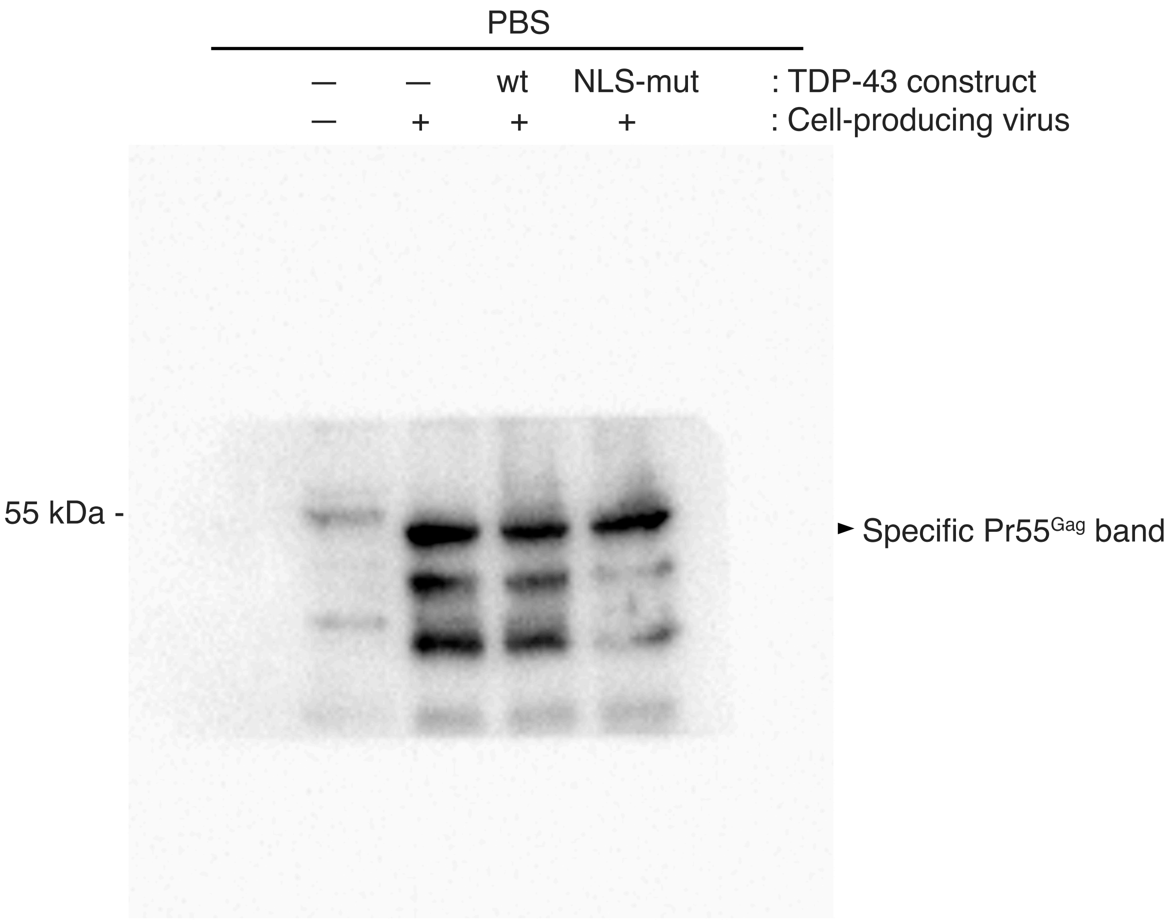

Figure S4. Replicate 2 Vif complete gel   Western-blot associated with Figure 4A
Cabrera-Rodríguez, R., *et al.*

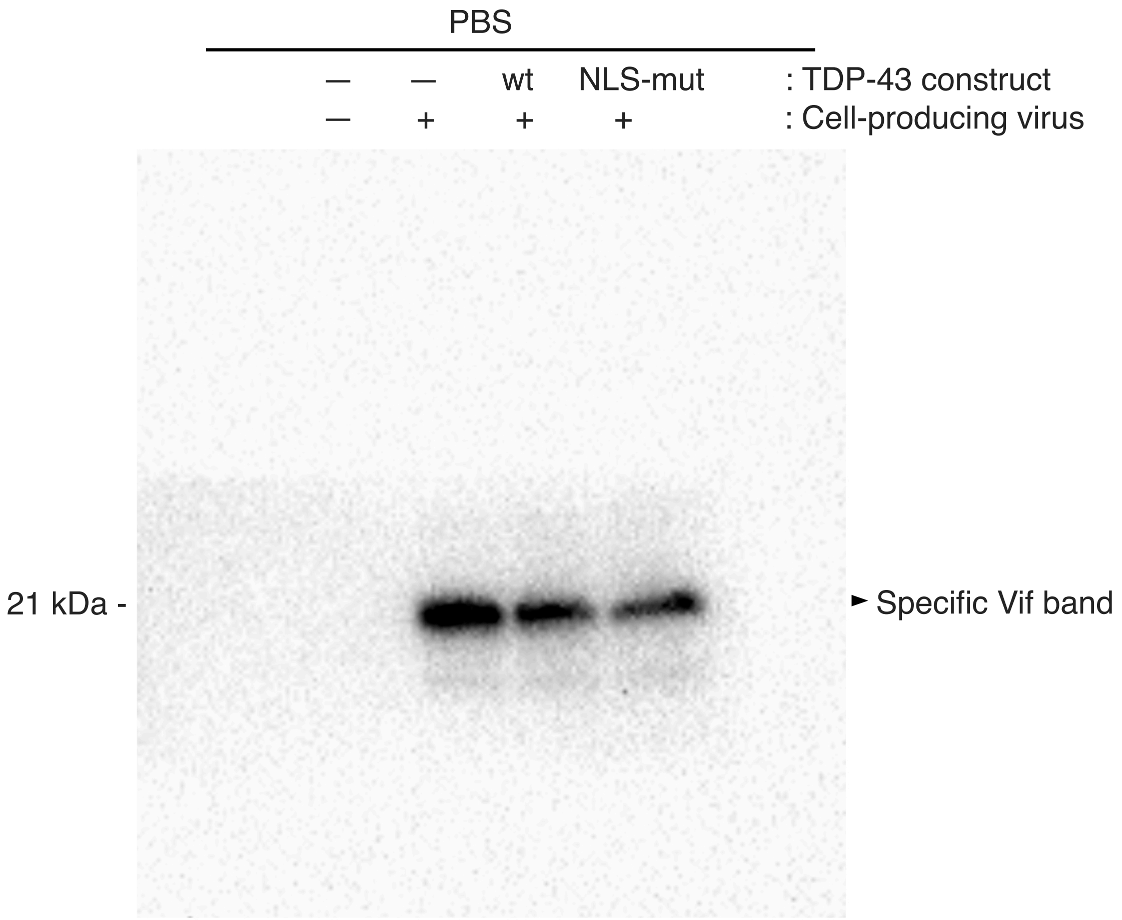

Figure S4. Replicate 2 Total α-tubulin complete gel   Western-blot associated with Figure 4A

Cabrera-Rodríguez, R., *et al.*

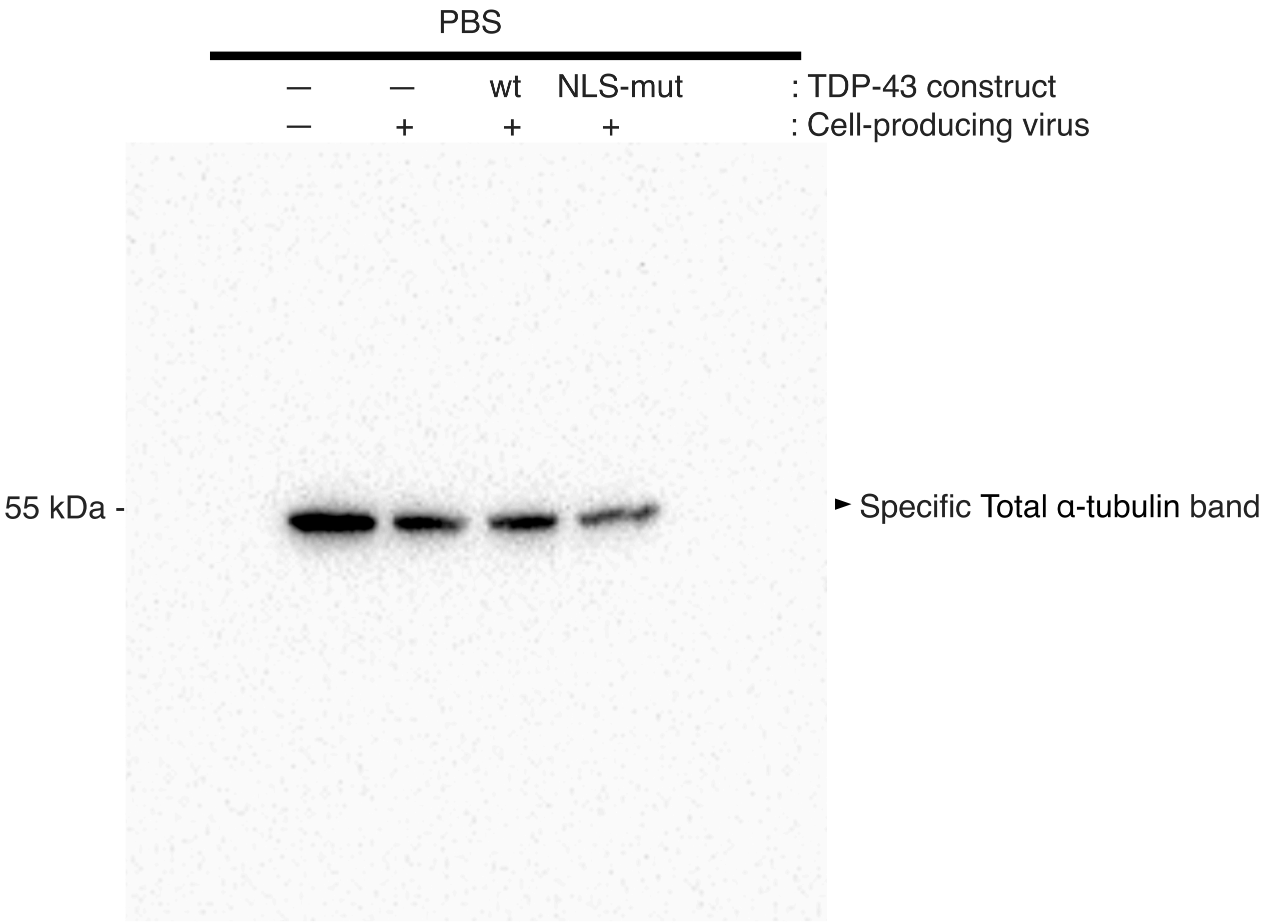

Figure S4. Replicate 2 HDAC6 complete gel Western-blot associated with Figure 4A
Cabrera-Rodríguez, R., *et al.*

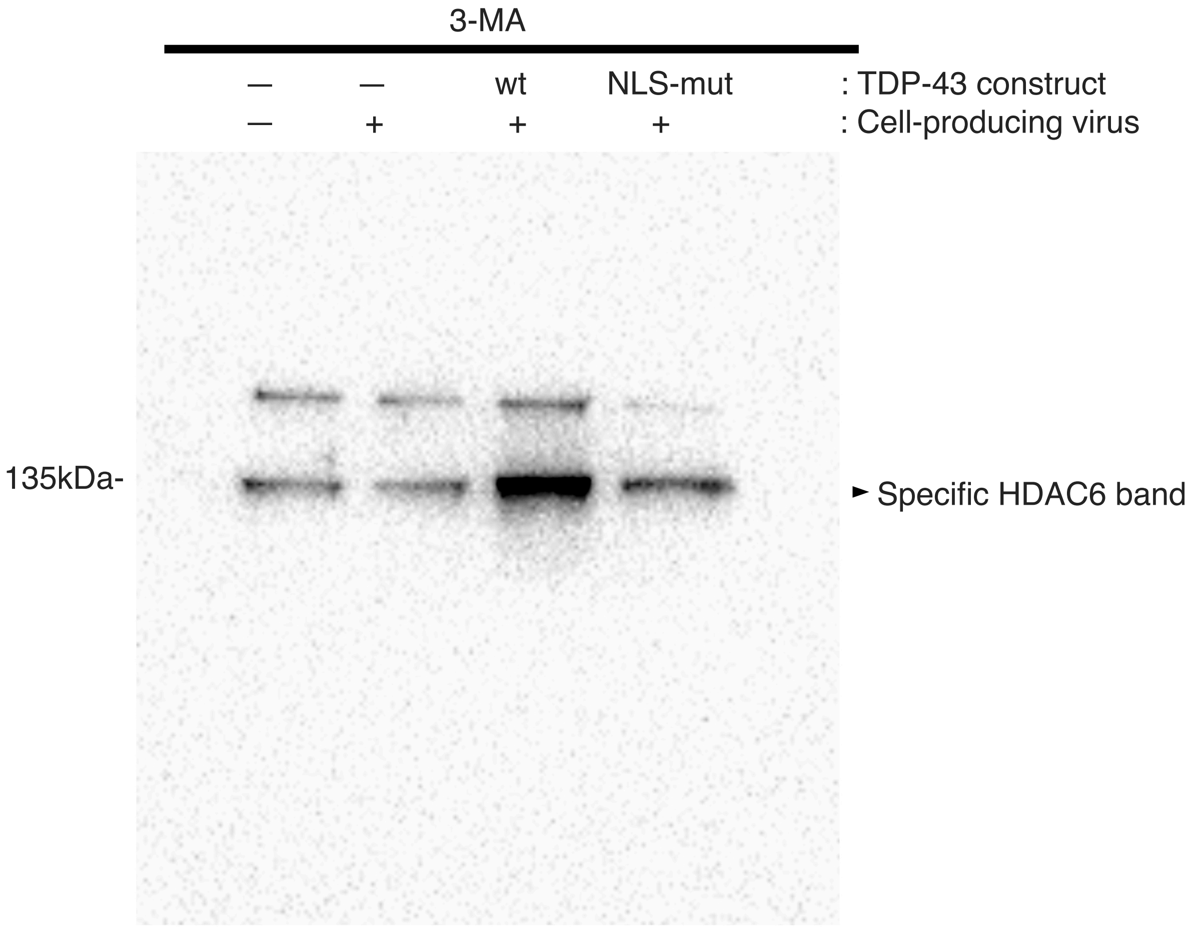

Figure S4. Replicate 2 p62 complete gel   Western-blot associated with Figure 4A
Cabrera-Rodríguez, R., *et al.*

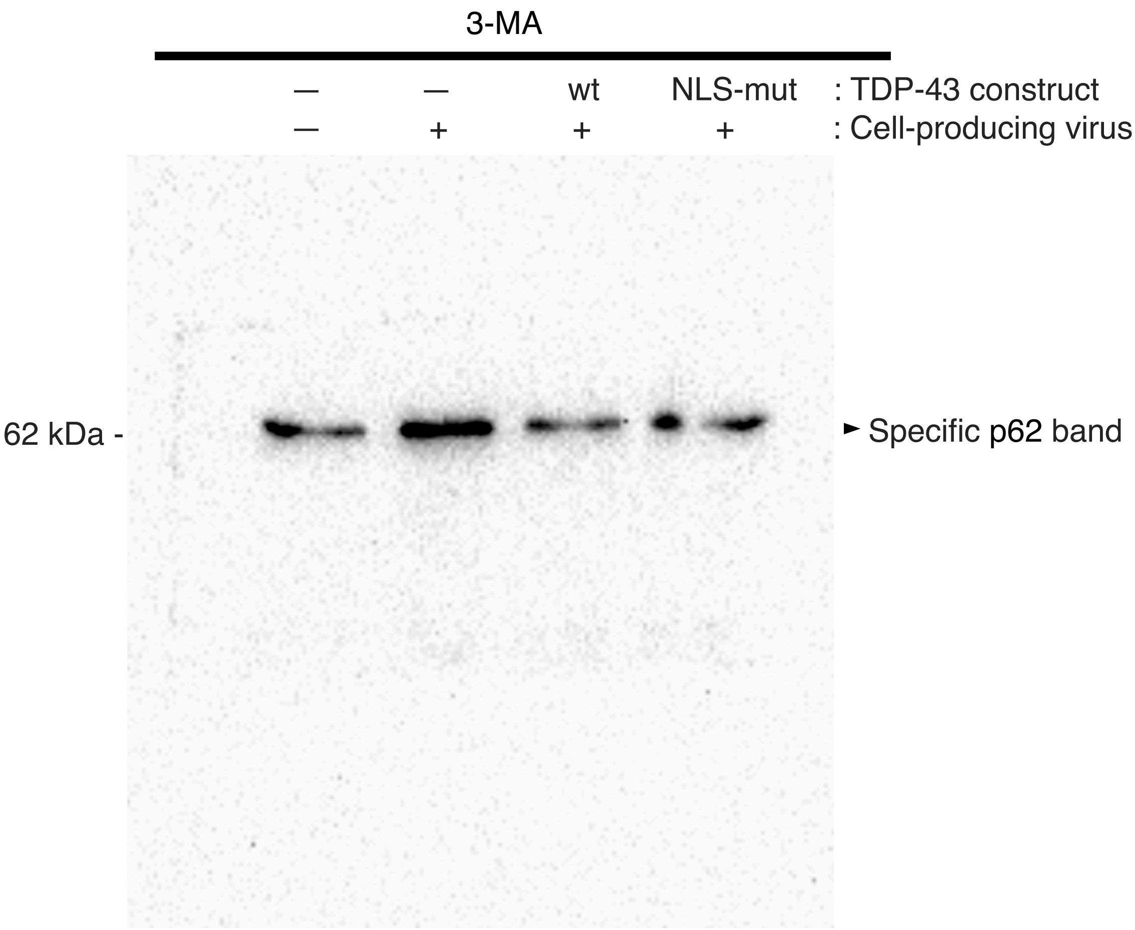

Figure S4. Replicate 2 TDP-43 complete gel Western-blot associated with Figure 4A

Cabrera-Rodríguez, R., *et al.*

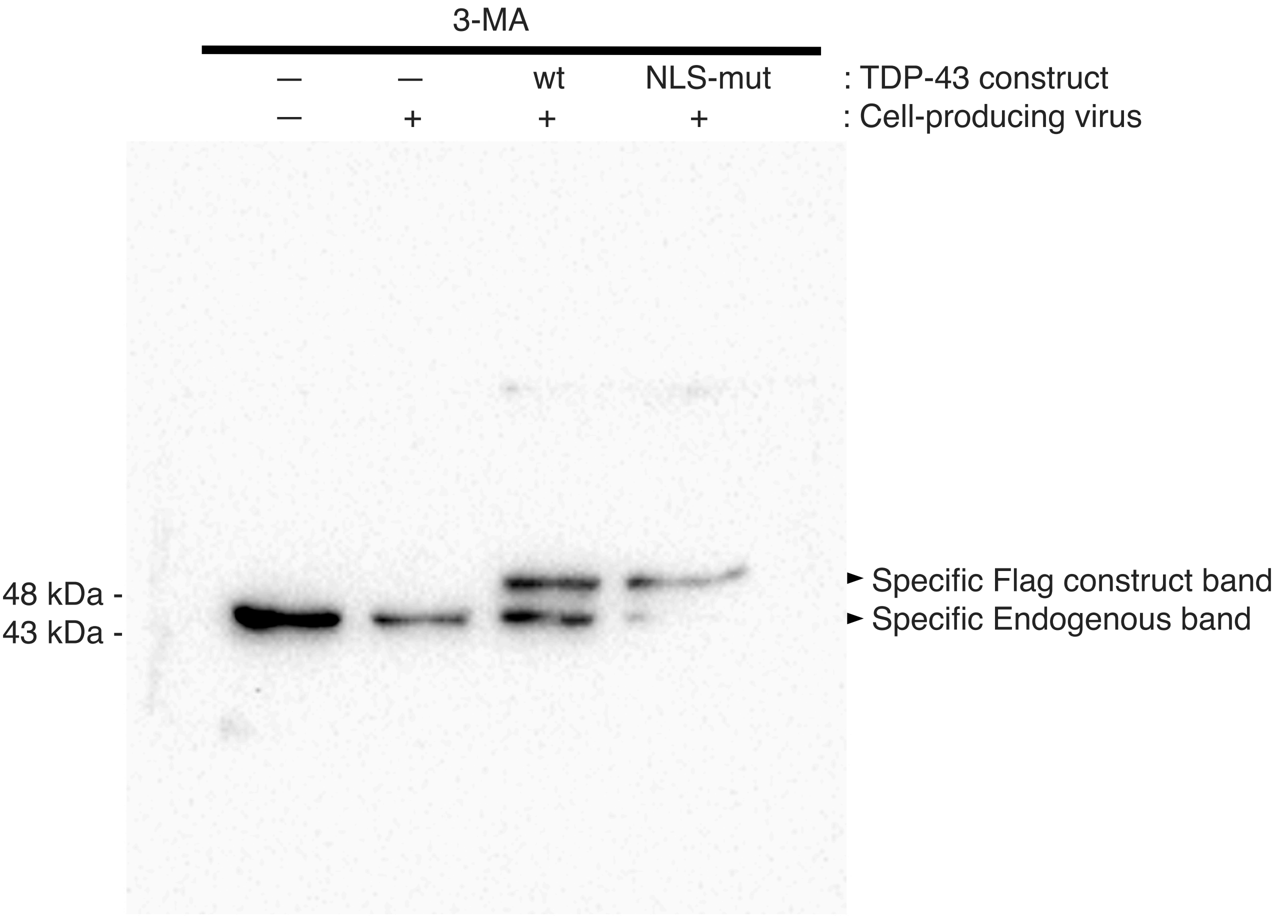

Figure S4. Replicate 2 $Pr55^{Gag}$ complete gel Western-blot associated with Figure 4A

Cabrera-Rodríguez, R., *et al.*

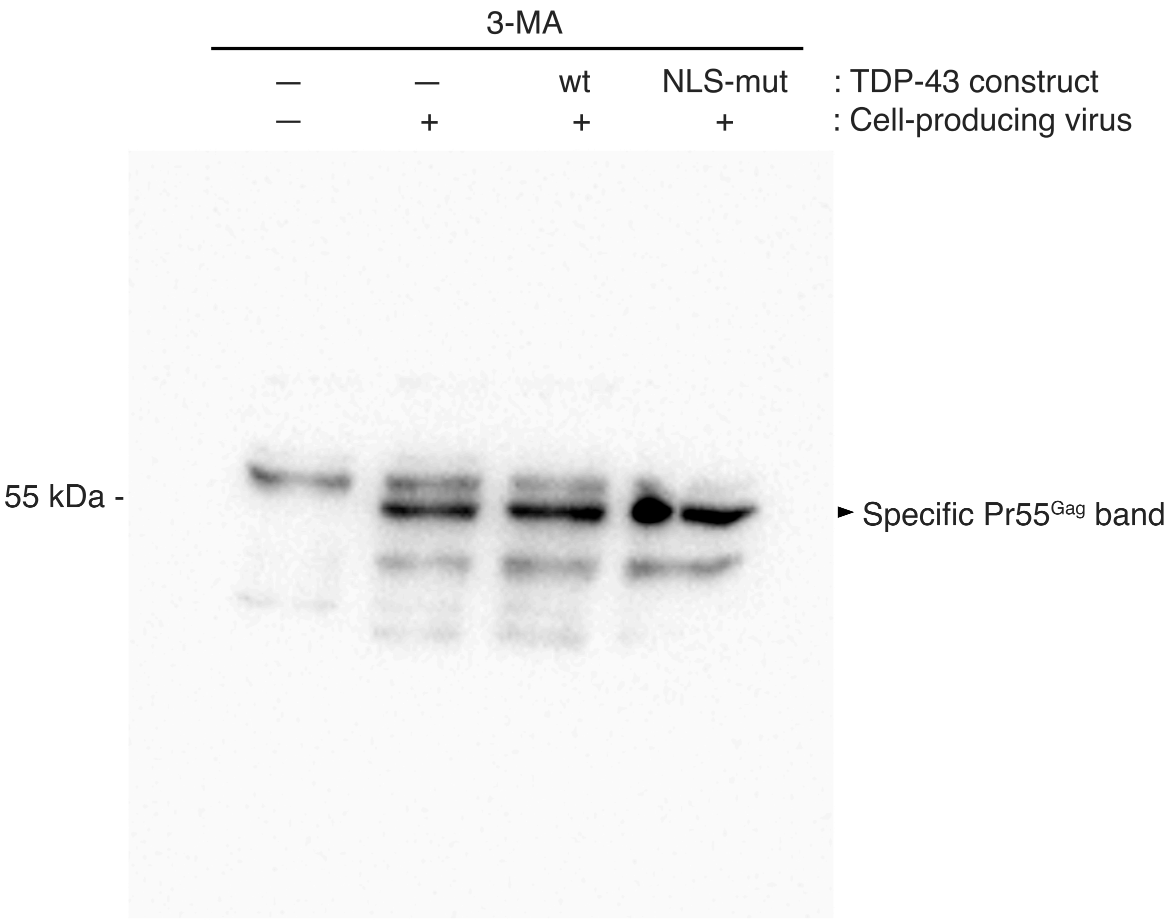

Figure S4. Replicate 2 Vif complete gel   Western-blot associated with Figure 4A
Cabrera-Rodríguez, R., *et al.*

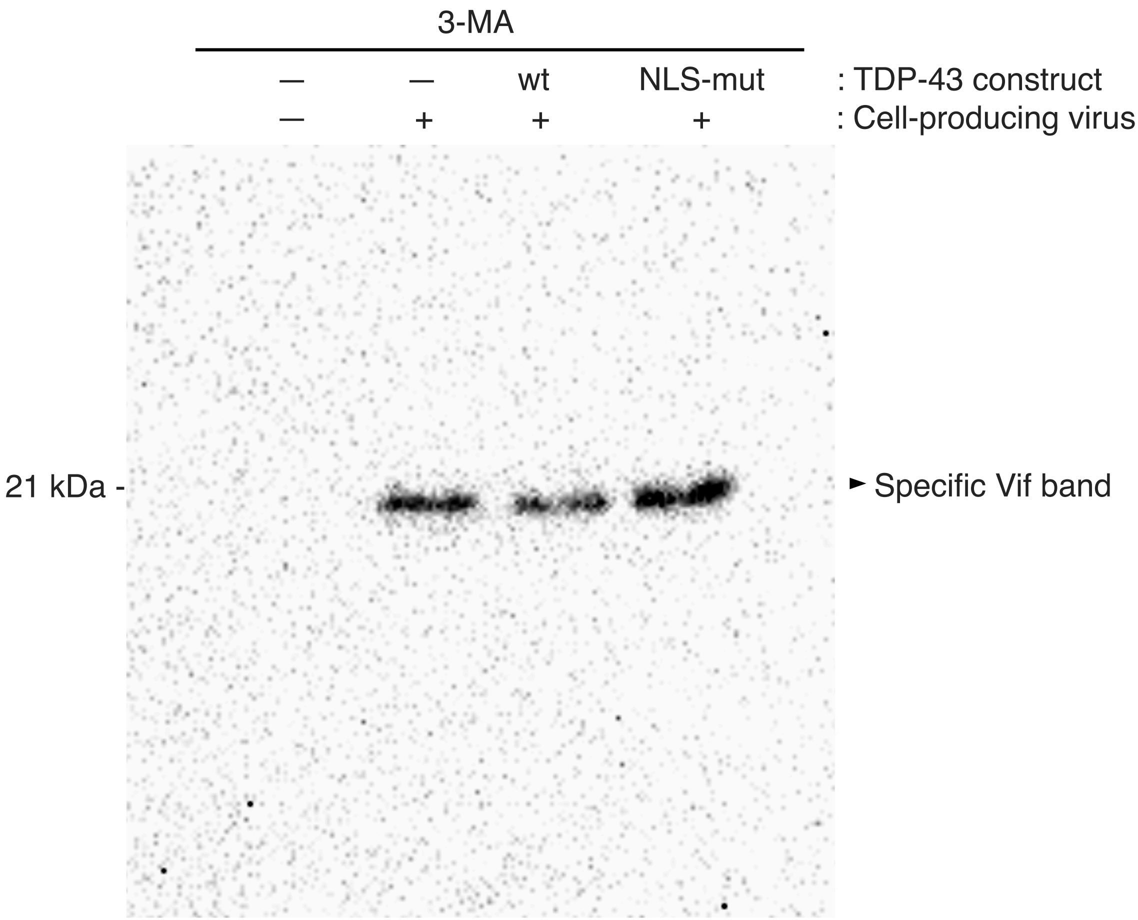

Figure S4. Replicate 2 Total α-tubulin complete gel   Western-blot associated with Figure 4A

Cabrera-Rodríguez, R., *et al.*

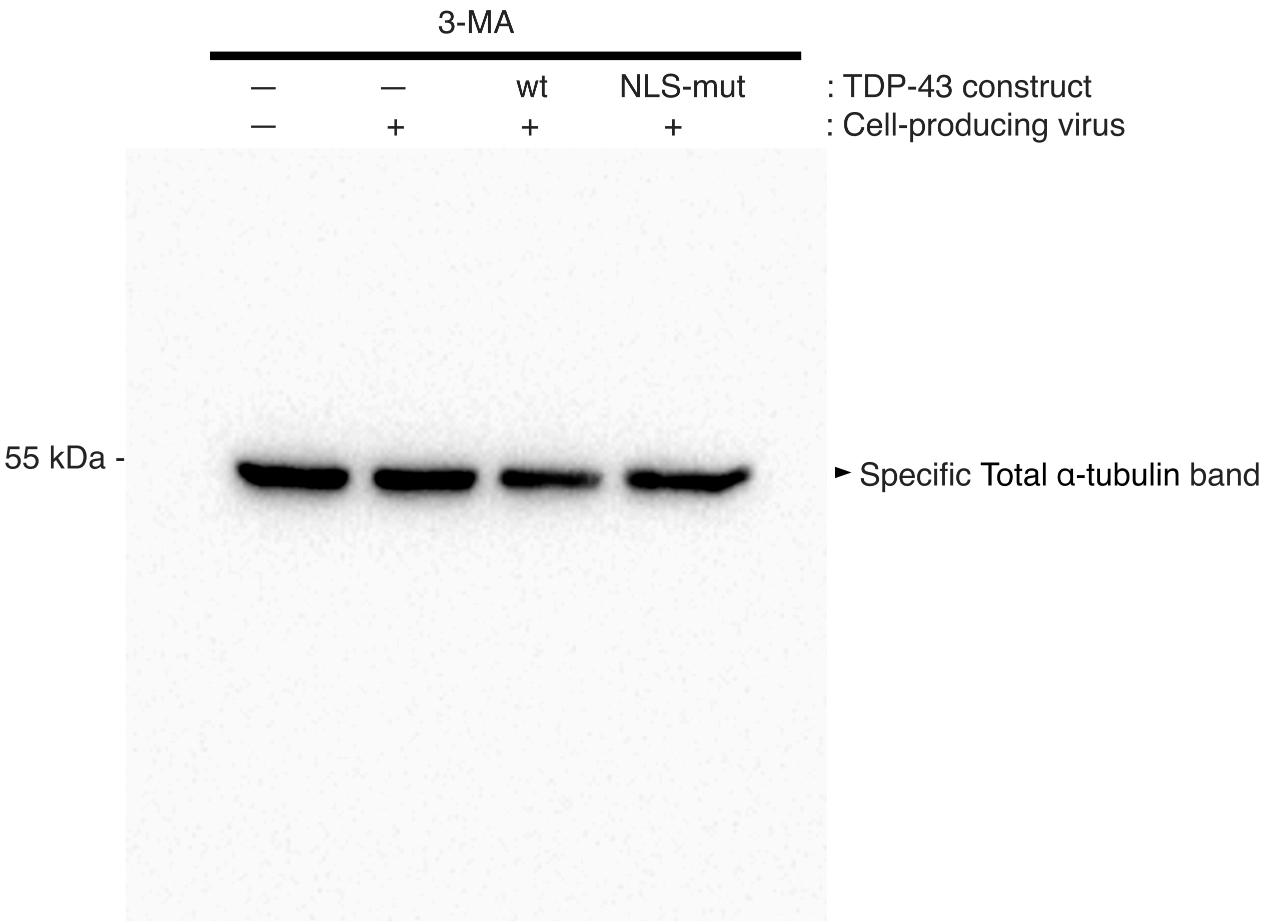

Figure S4. Replicate 3 HDAC6 complete gel Western-blot associated with Figure 4A
Cabrera-Rodríguez, R., *et al.*

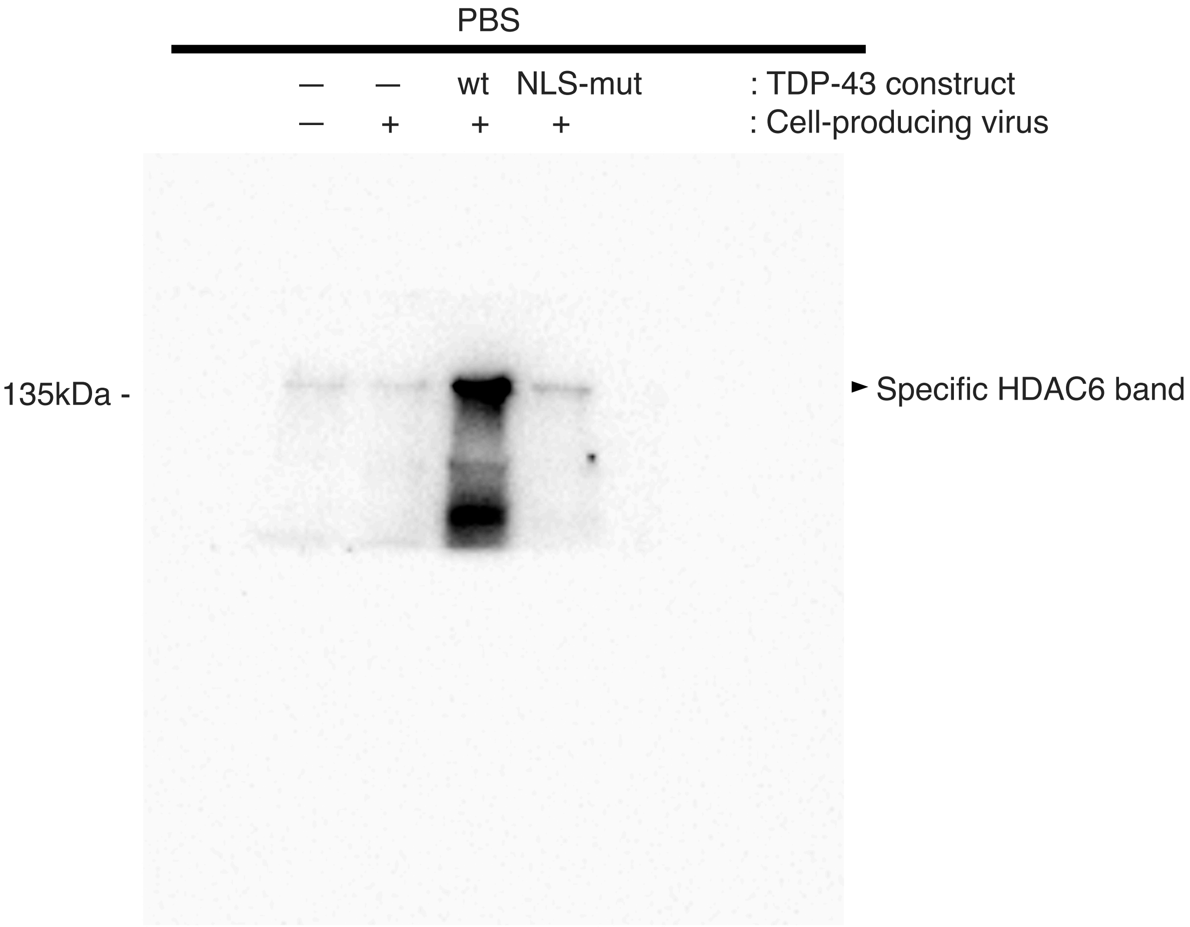

Figure S4. Replicate 3 p62 complete gel Western-blot associated with Figure 4A
Cabrera-Rodríguez, R., *et al.*

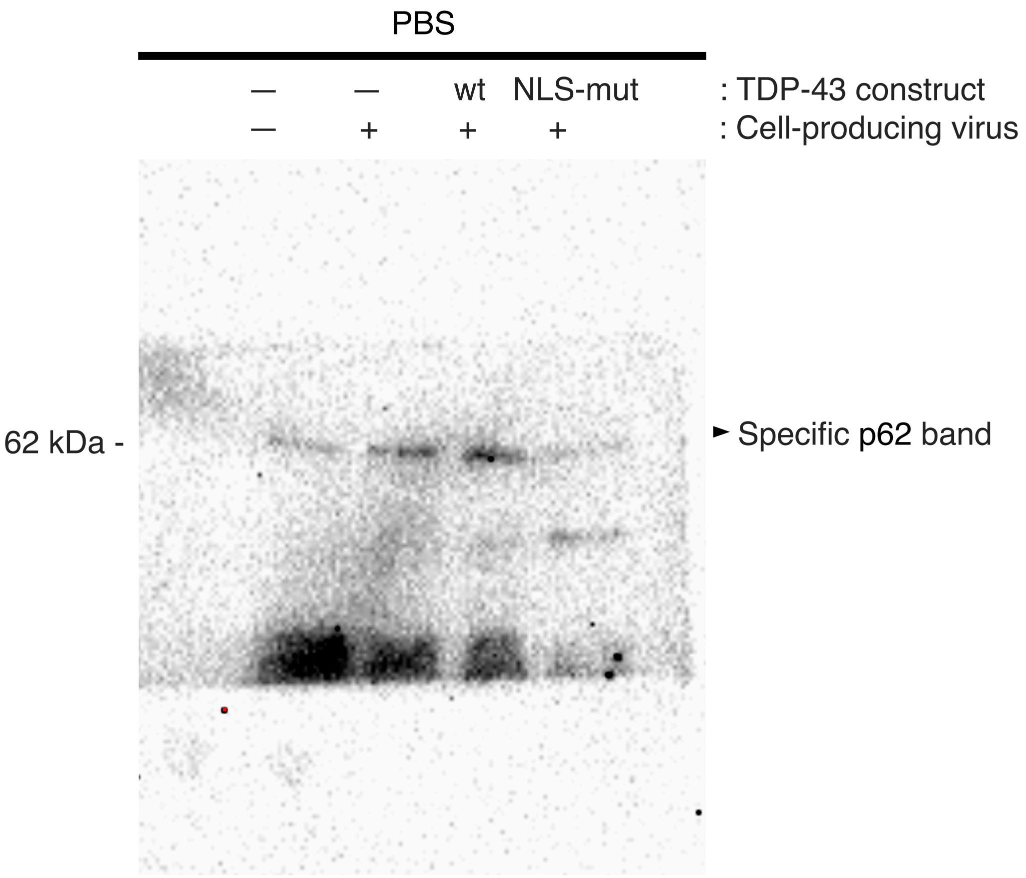

Figure S4. Replicate 3 TDP-43 complete gel Western-blot associated with Figure 4A
Cabrera-Rodríguez, R., *et al.*

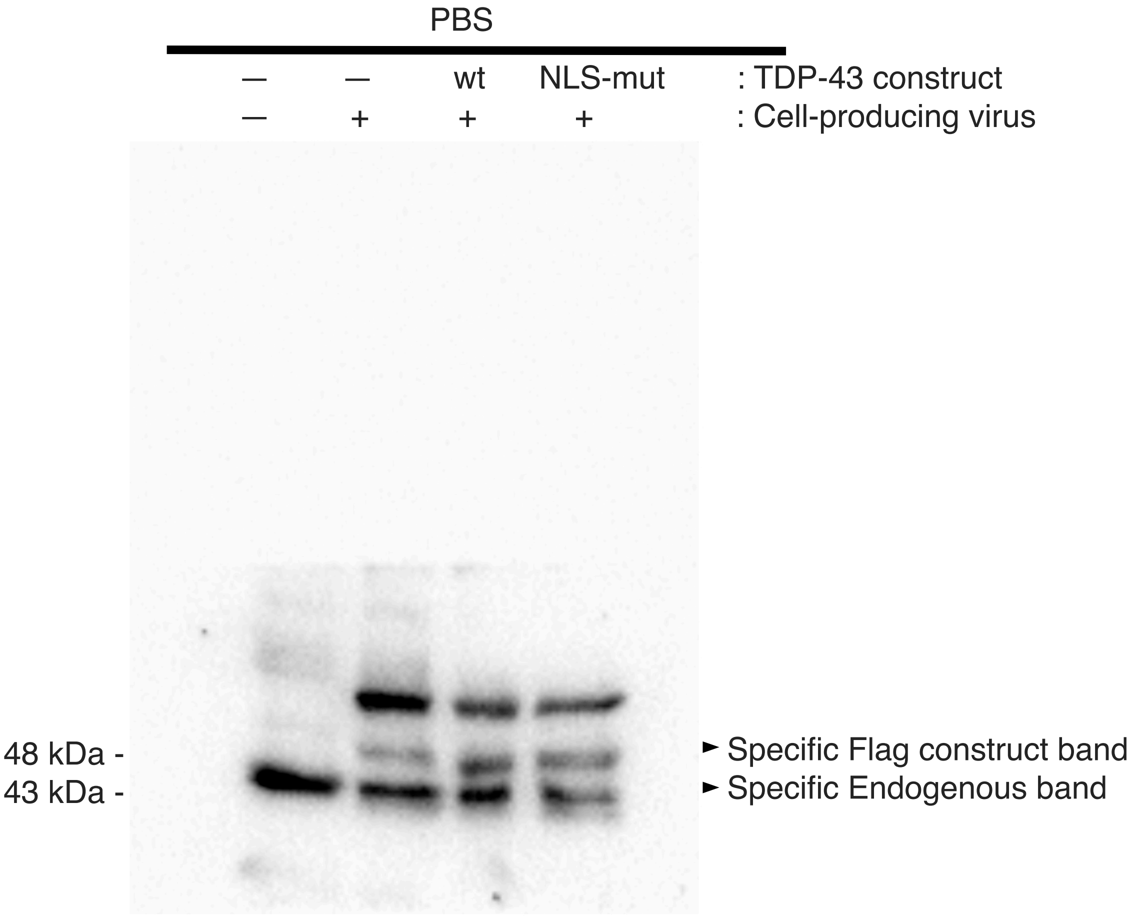

Figure S4. Replicate 3 Pr55^{Gag} complete gel Western-blot associated with Figure 4A

Cabrera-Rodríguez, R., *et al.*

PBS

—	—	wt	NLS-mut	: TDP-43 construct
—	+	+	+	: Cell-producing virus

55 kDa -

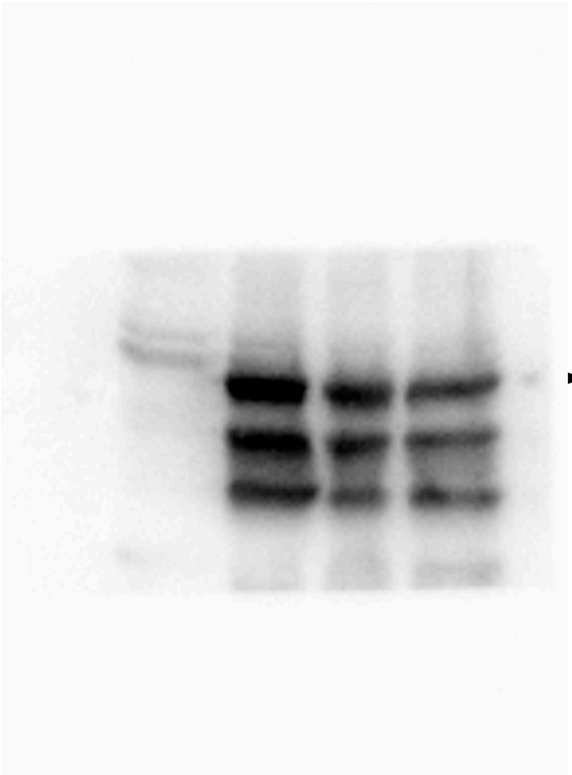

► Specific Pr55^{Gag} band

Figure S4. Replicate 3 Vif complete gel Western-blot associated with Figure 4A

Cabrera-Rodríguez, R., *et al.*

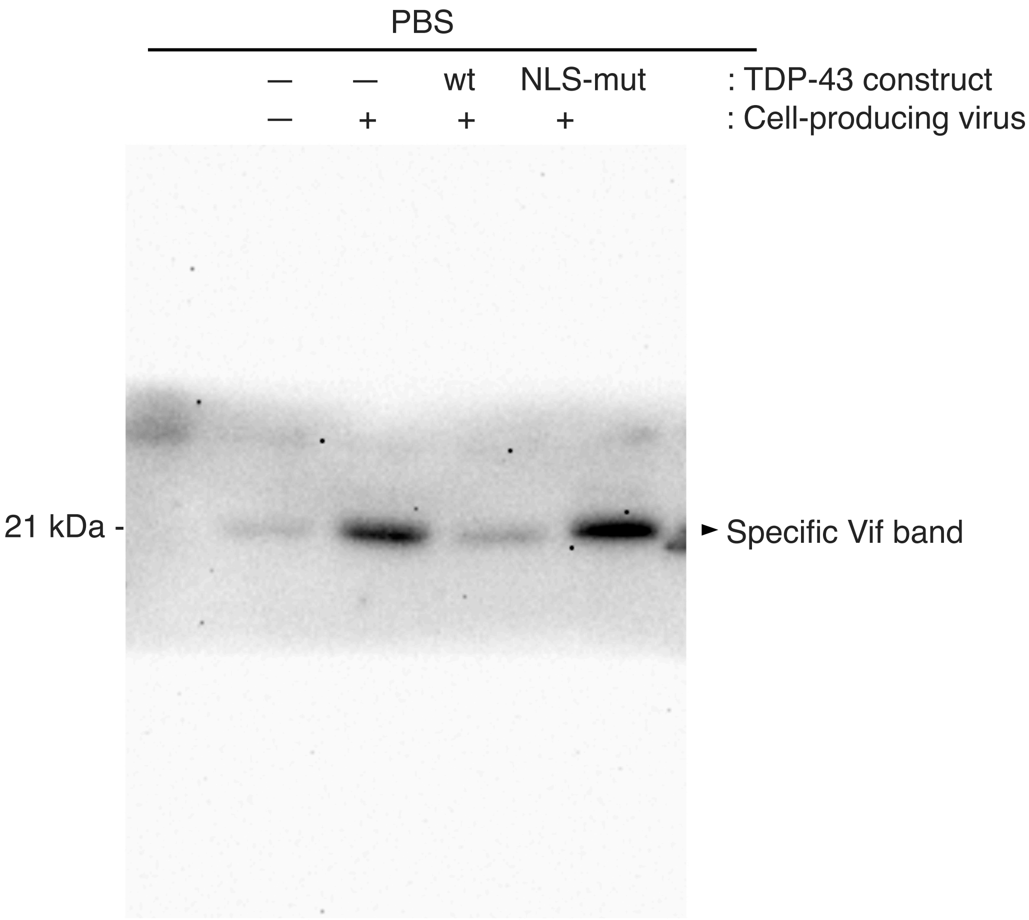

Figure S4. Replicate 3 Total α-tubulin complete gel Western-blot associated with Figure 4A

Cabrera-Rodríguez, R., *et al.*

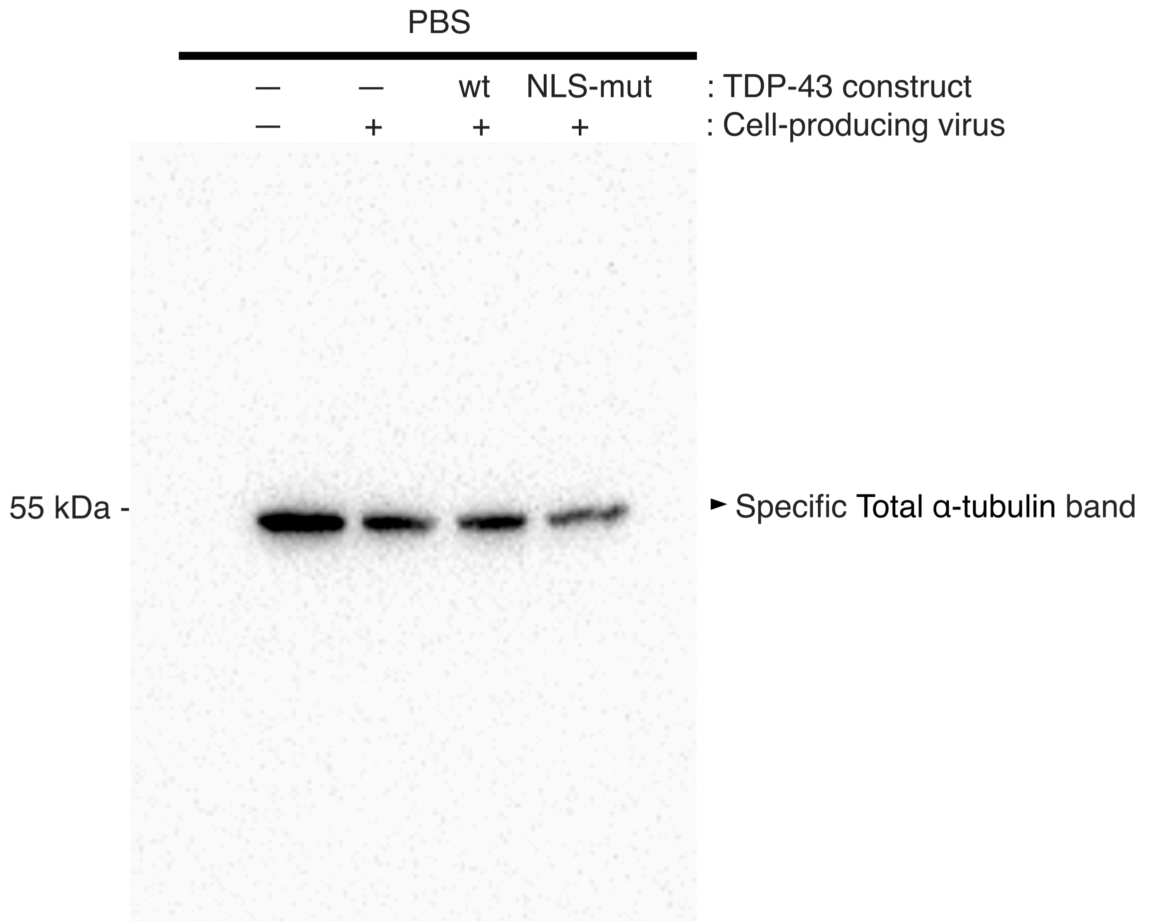

Figure S4. Replicate 3 HDAC6 complete gel Western-blot associated with Figure 4A

Cabrera-Rodríguez, R., *et al.*

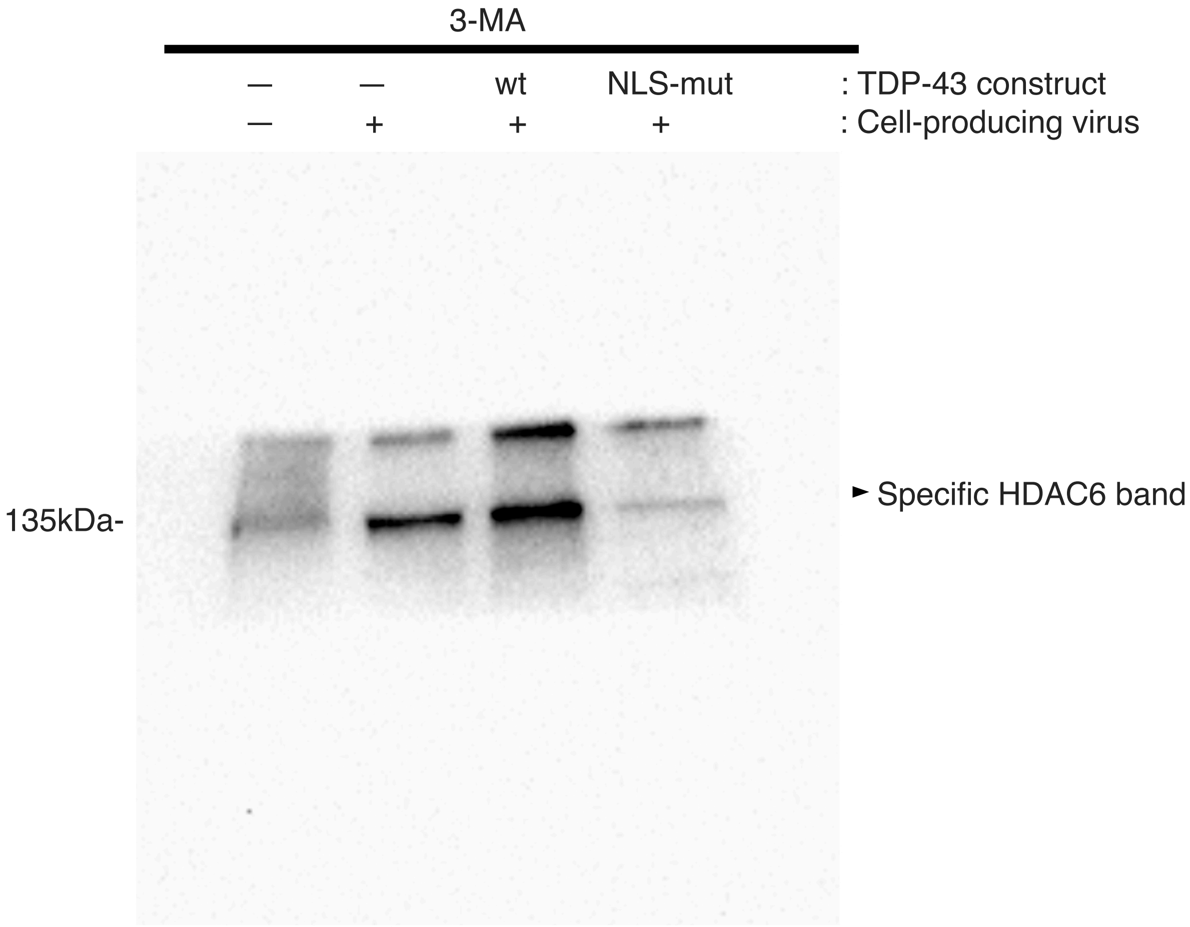

Figure S4. Replicate 3 p62 complete gel Western-blot associated with Figure 4A
Cabrera-Rodríguez, R., *et al.*

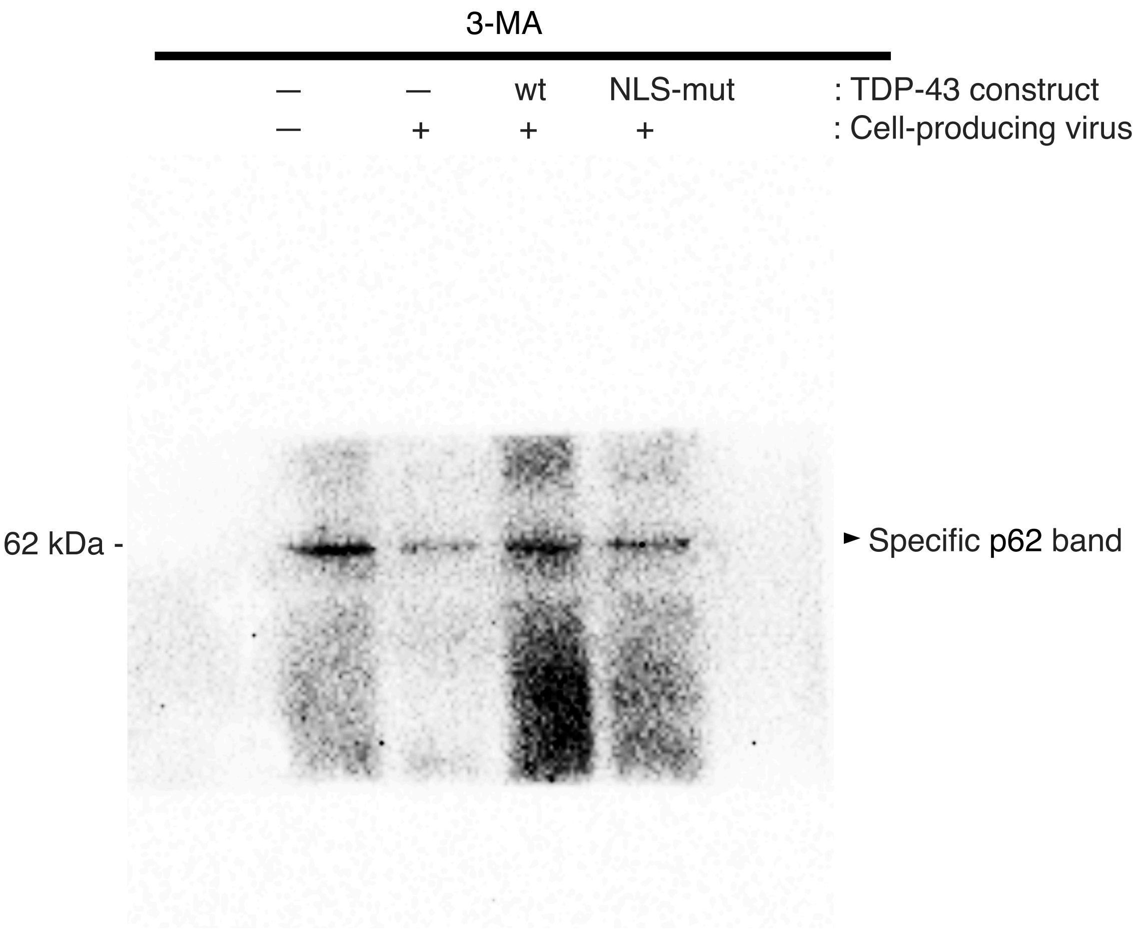

Figure S4. Replicate 3 TDP-43 complete gel Western-blot associated with Figure 4A

Cabrera-Rodríguez, R., *et al.*

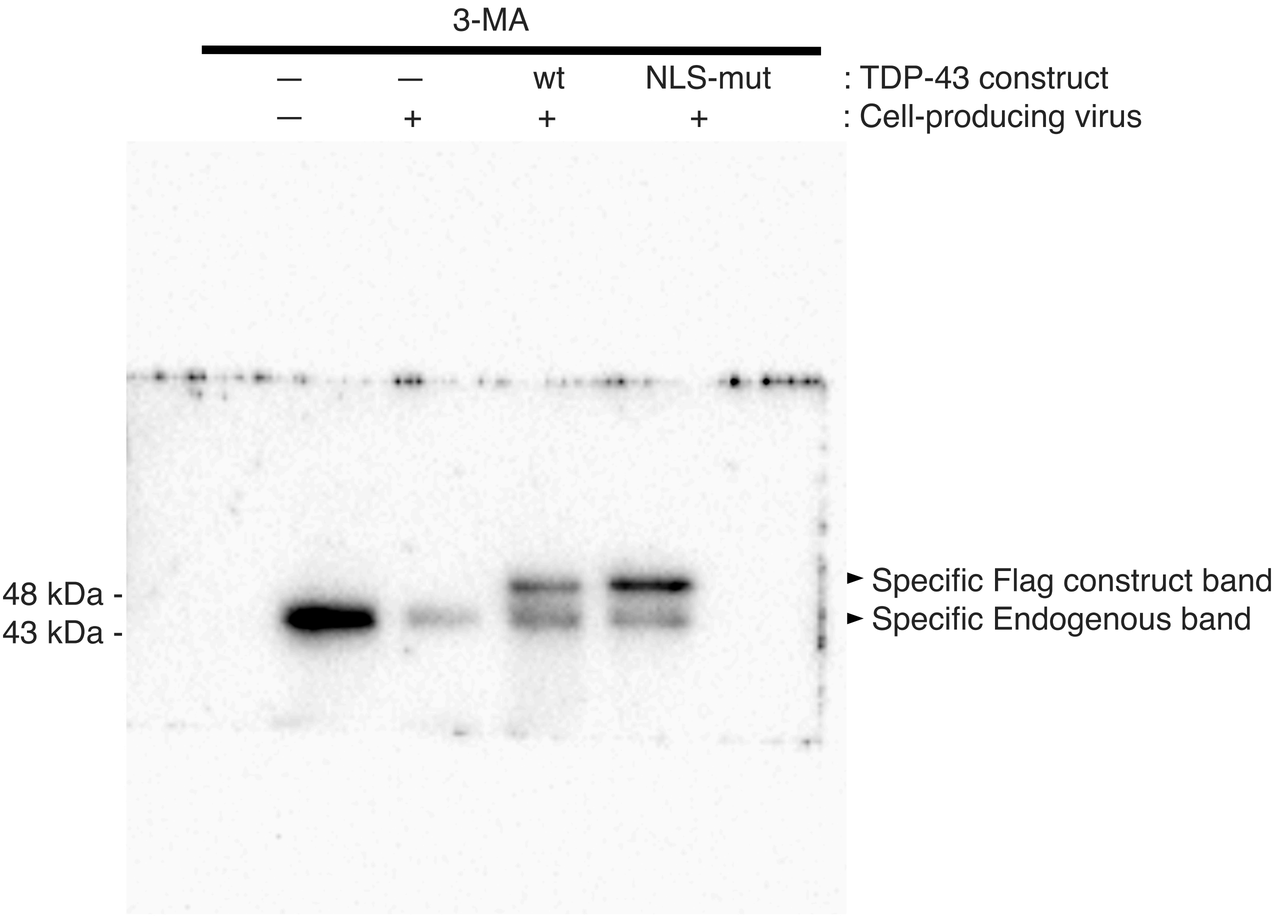

Figure S4. Replicate 3 Pr55^{Gag} complete gel   Western-blot associated with Figure 4A

Cabrera-Rodríguez, R., *et al.*

3-MA

—	—	wt	NLS-mut	: TDP-43 construct
—	+	+	+	: Cell-producing virus

55 kDa -

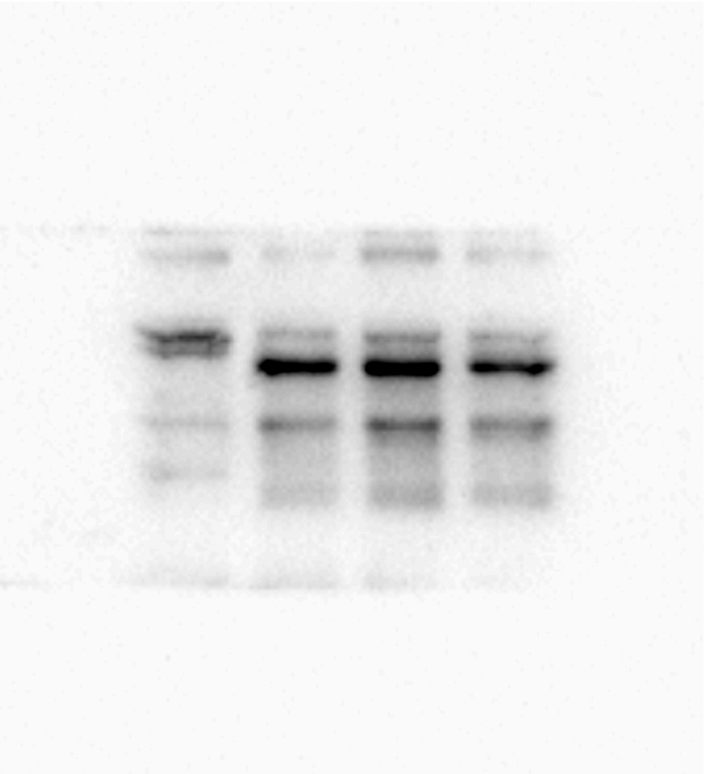

► Specific Pr55^{Gag} band

Figure S4. Replicate 3 Vif complete gel   Western-blot associated with Figure 4A
Cabrera-Rodríguez, R., *et al.*

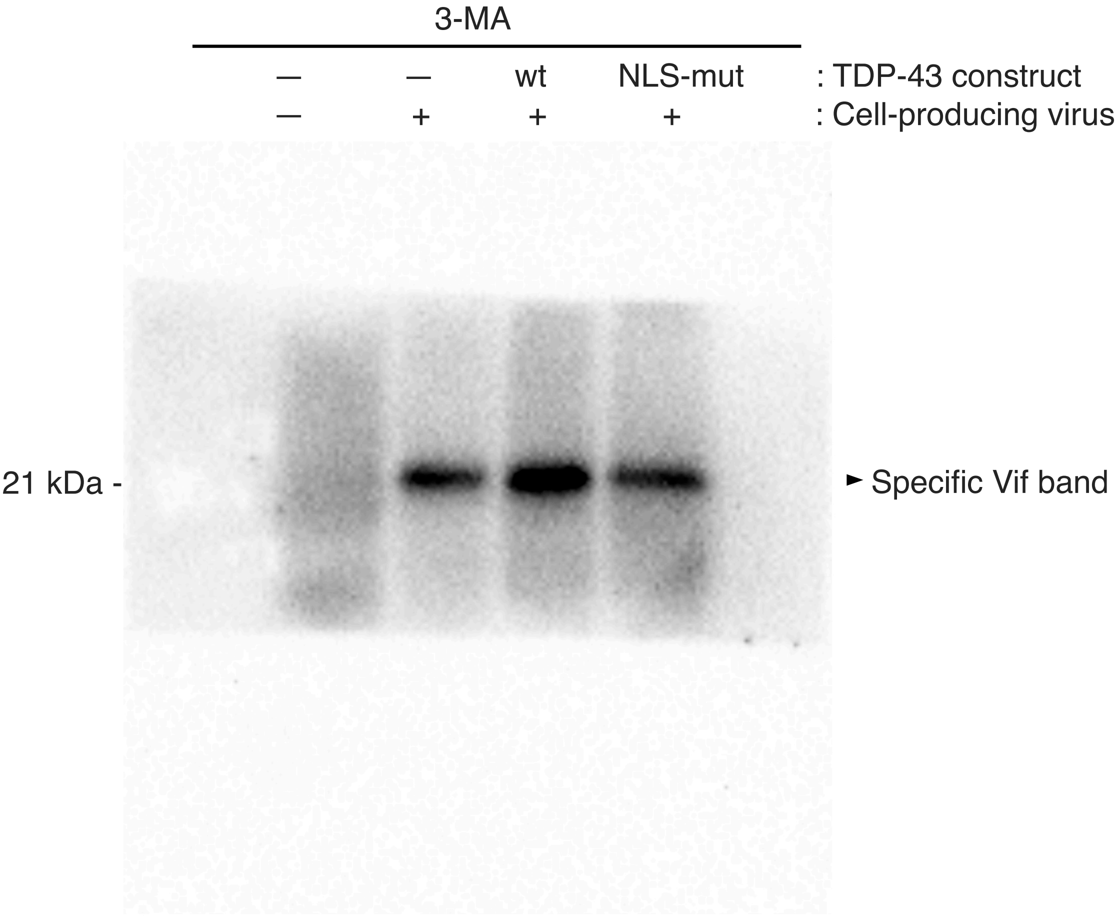

Figure S4. Replicate 3 Total α-tubulin complete gel   Western-blot associated with Figure 4A
Cabrera-Rodríguez, R., *et al.*

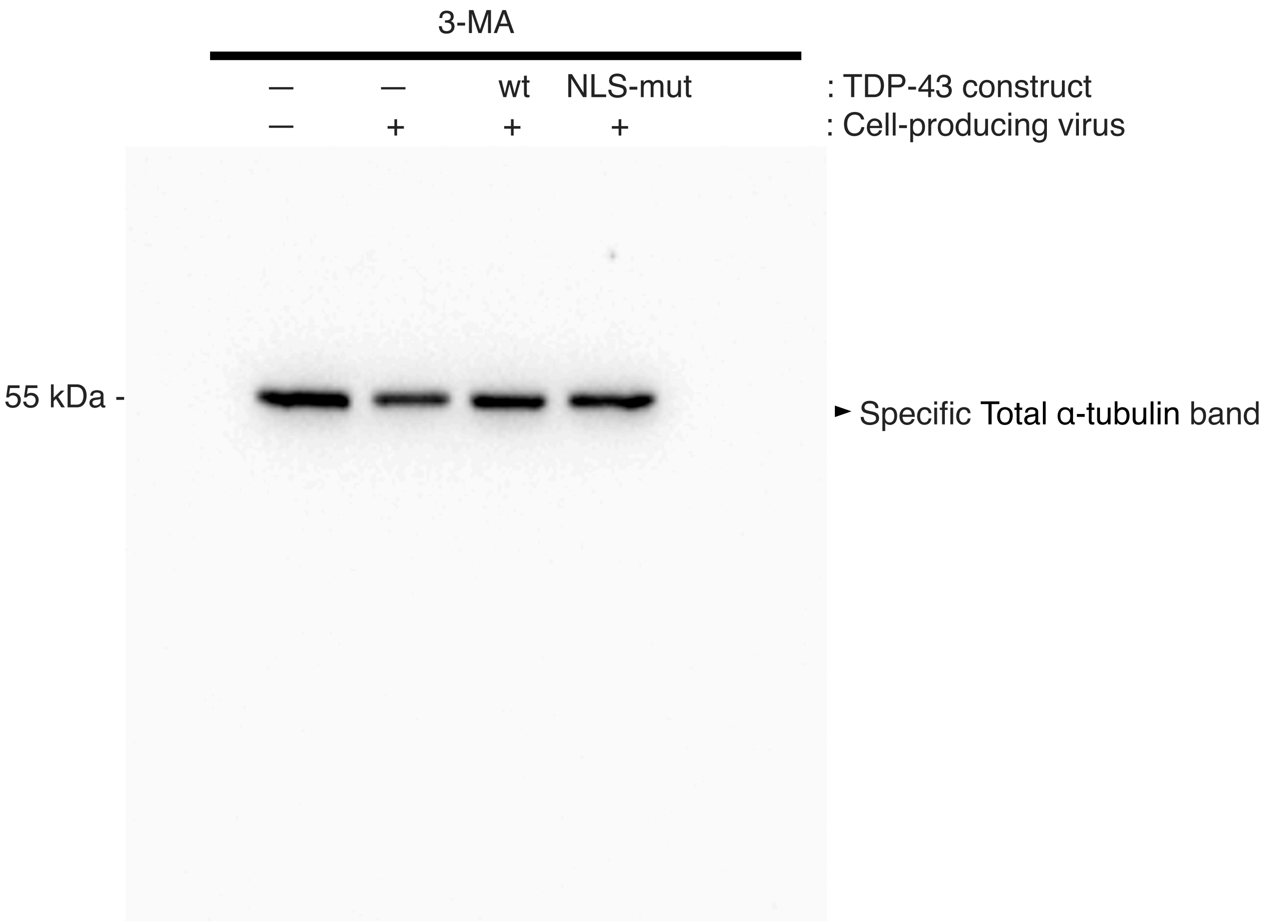

Figure S5. Western-blot replicates associated with Figure 5 Cabrera-Rodríguez R., *et al.*

Replicate 1 as figure format

A

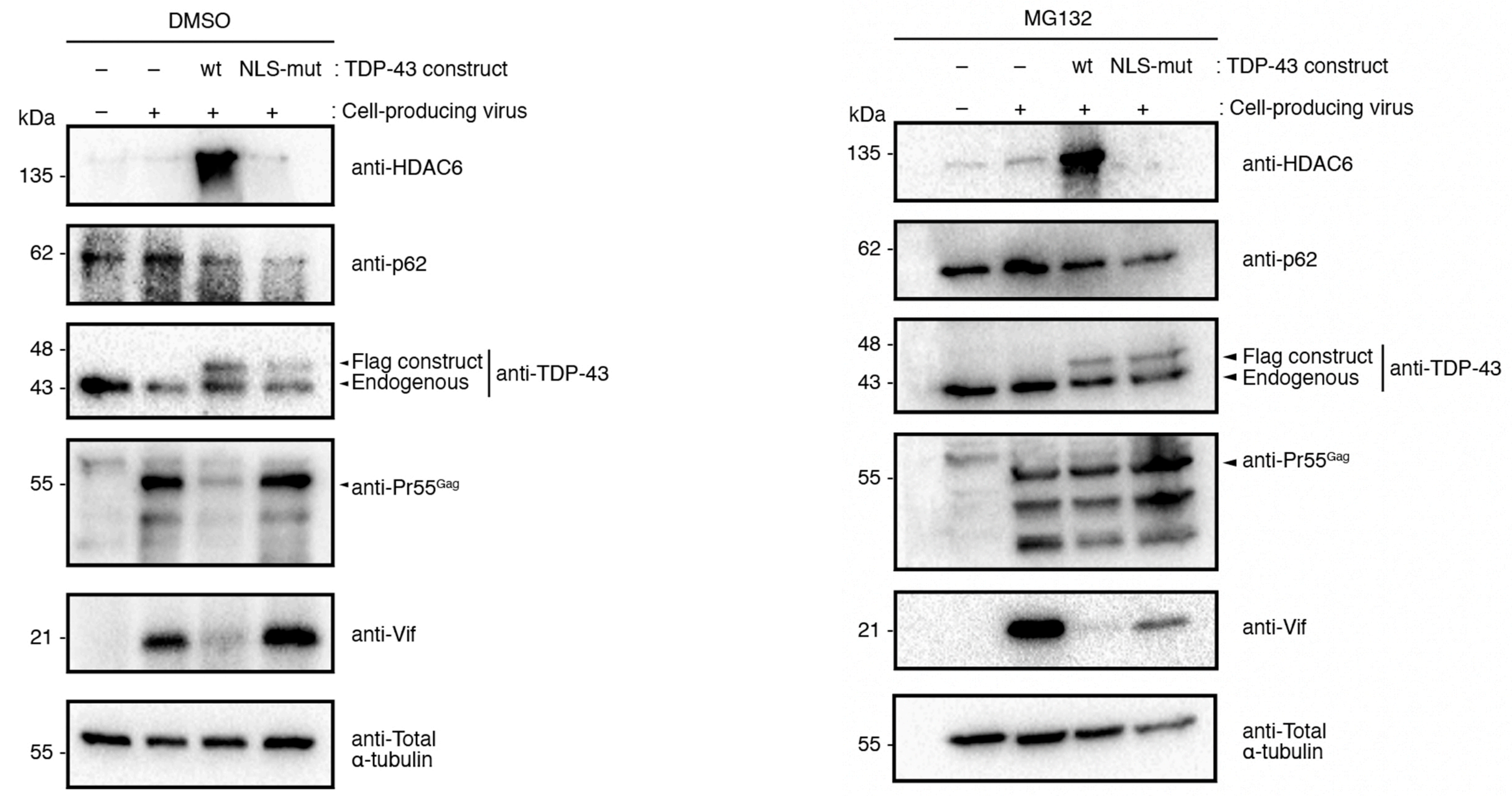

Replicate 2 as figure format

A

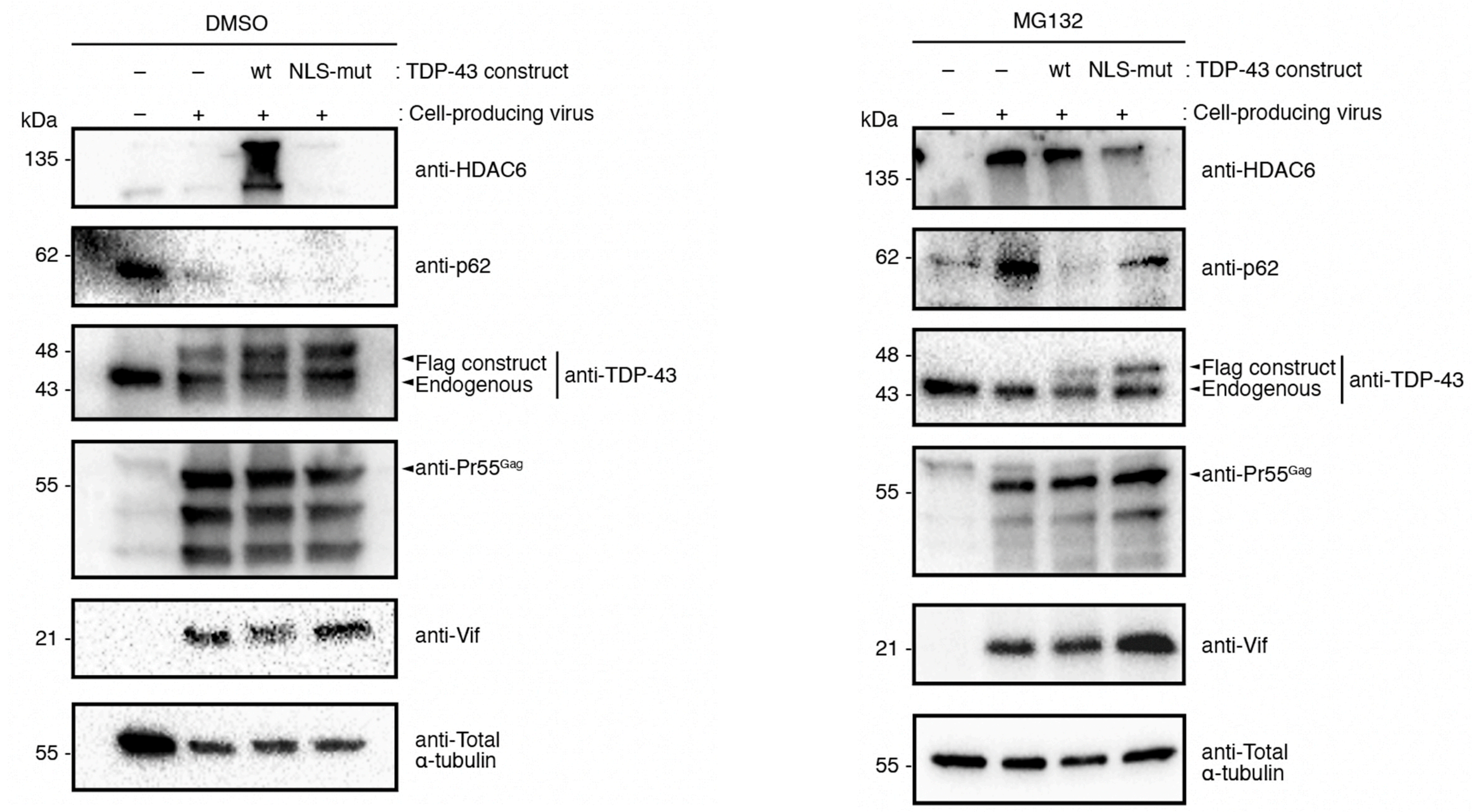

Replicate 3 as figure format

A

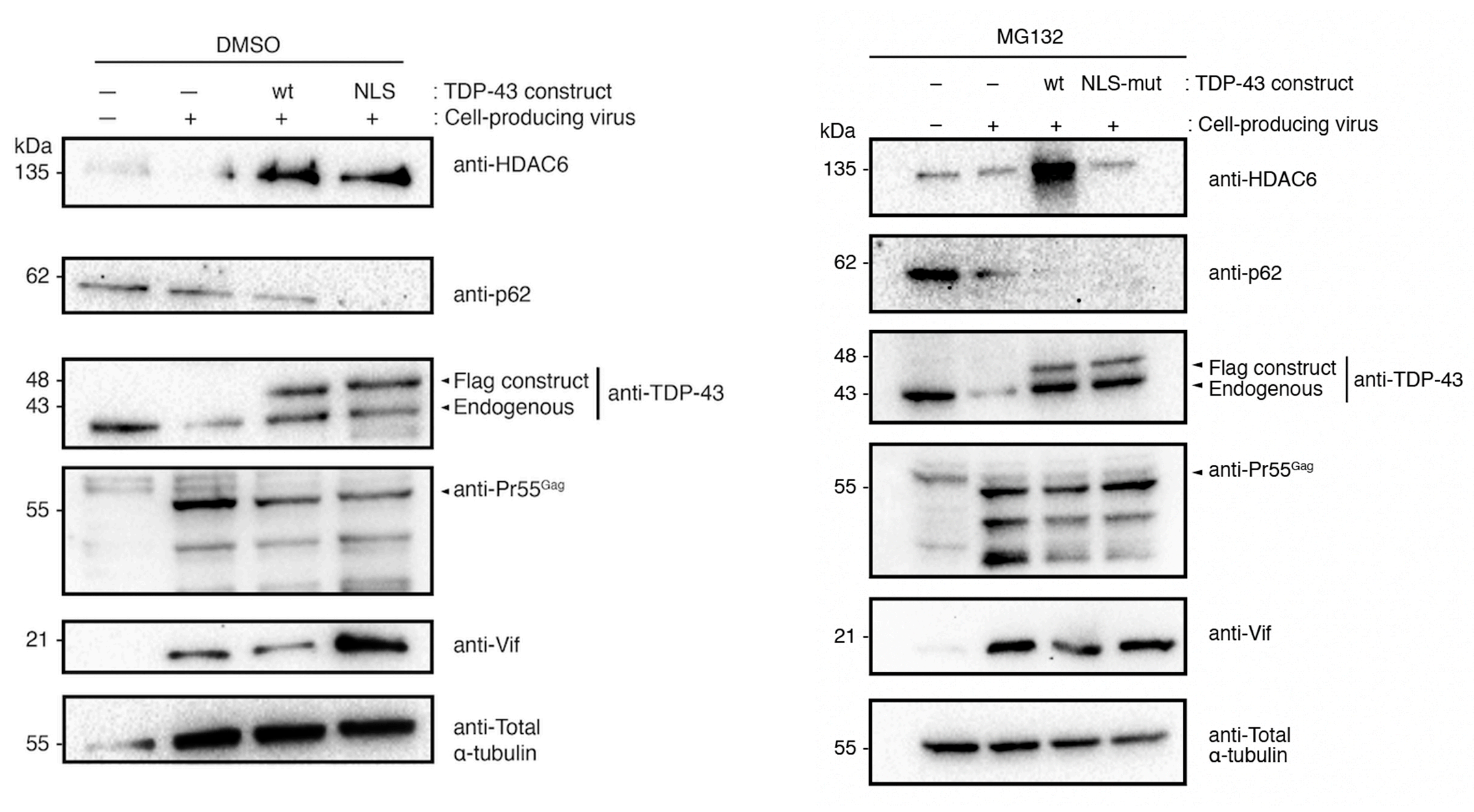

Figure S5. Replicate 1 HDAC6 complete gel Western-blot associated with Figure 5A
Cabrera-Rodríguez, R., *et al.*

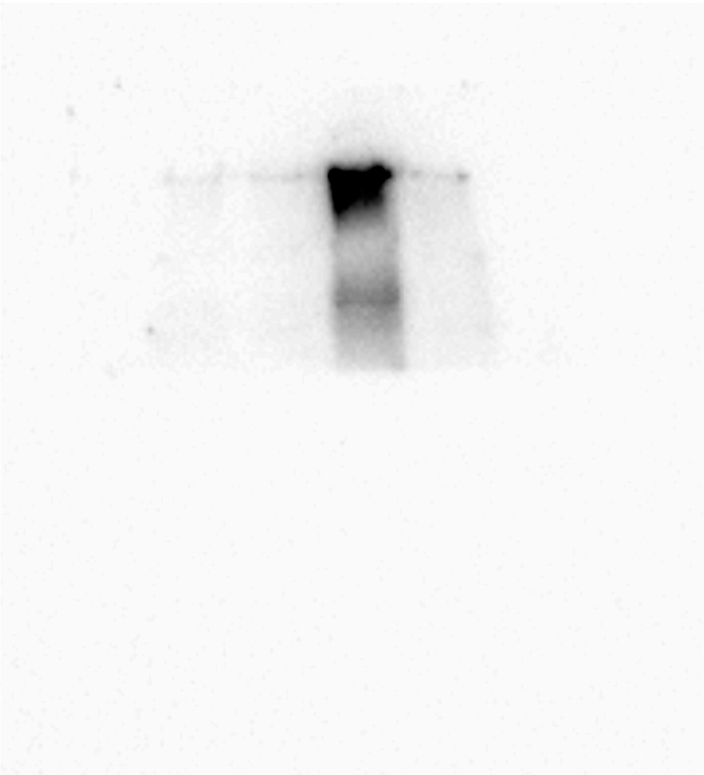

Figure S5. Replicate 1 p62 complete gel Western-blot associated with Figure 5A
Cabrera-Rodríguez, R., *et al.*

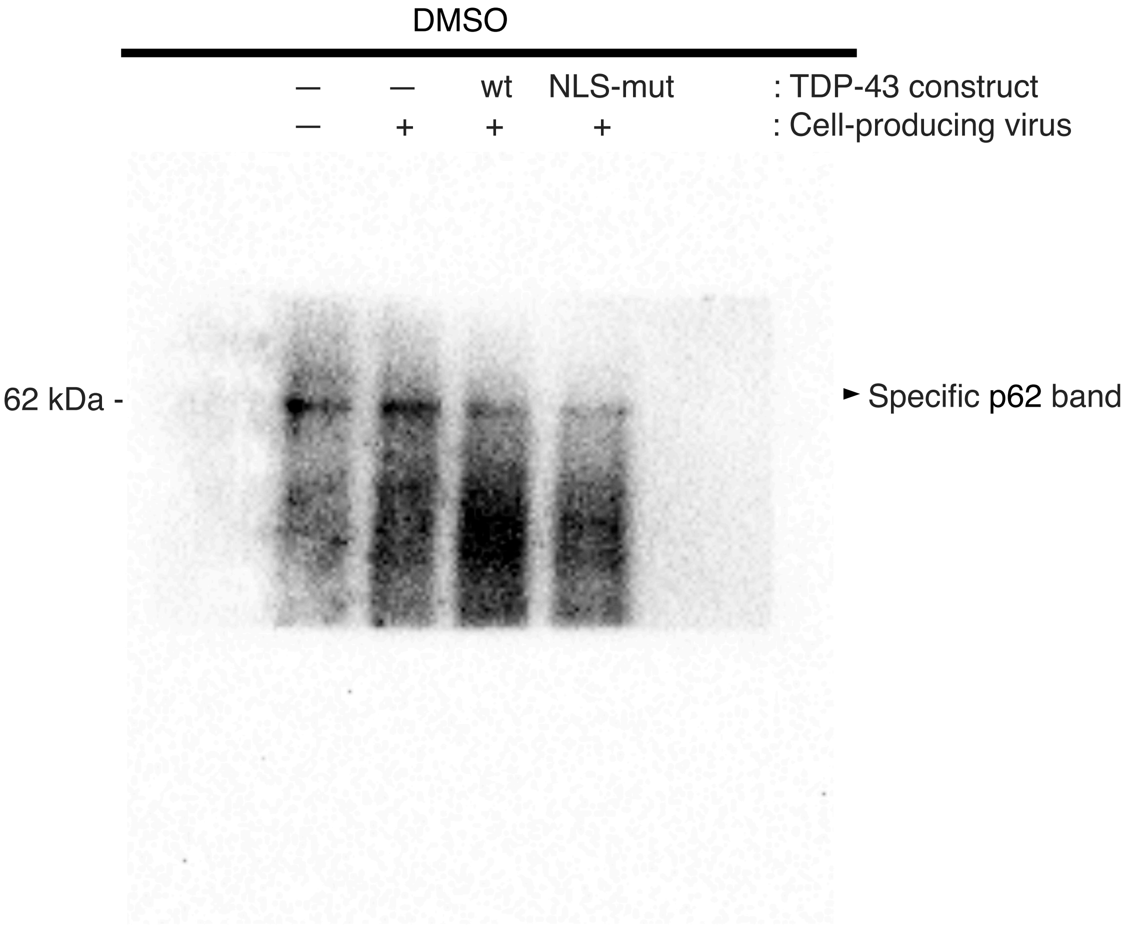

Figure S5. Replicate 1 TDP-43 complete gel   Western-blot associated with Figure 5A
Cabrera-Rodríguez, R., *et al.*

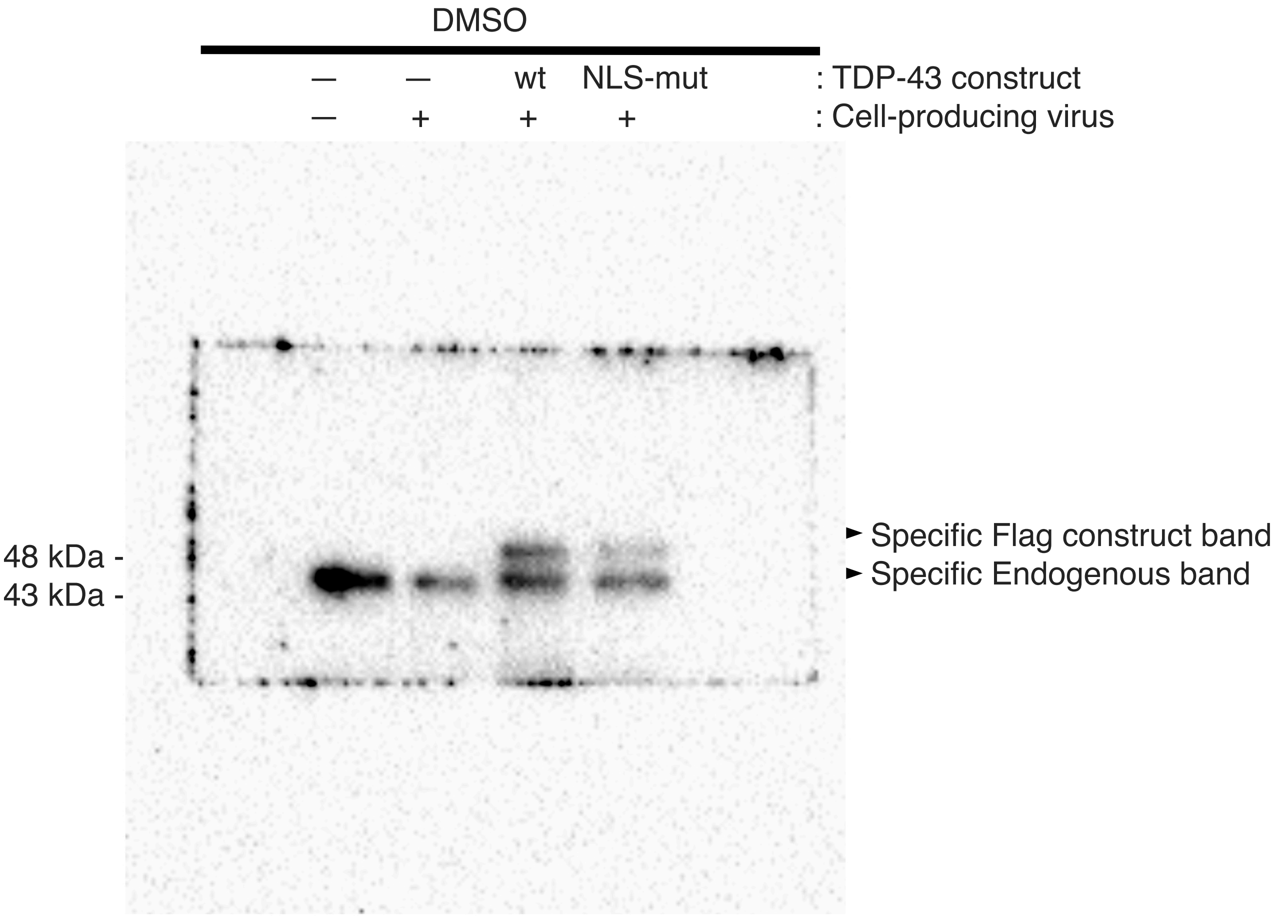

Figure S5. Replicate 1 Pr55^{Gag} complete gel Western-blot associated with Figure 5A
Cabrera-Rodríguez, R., *et al.*

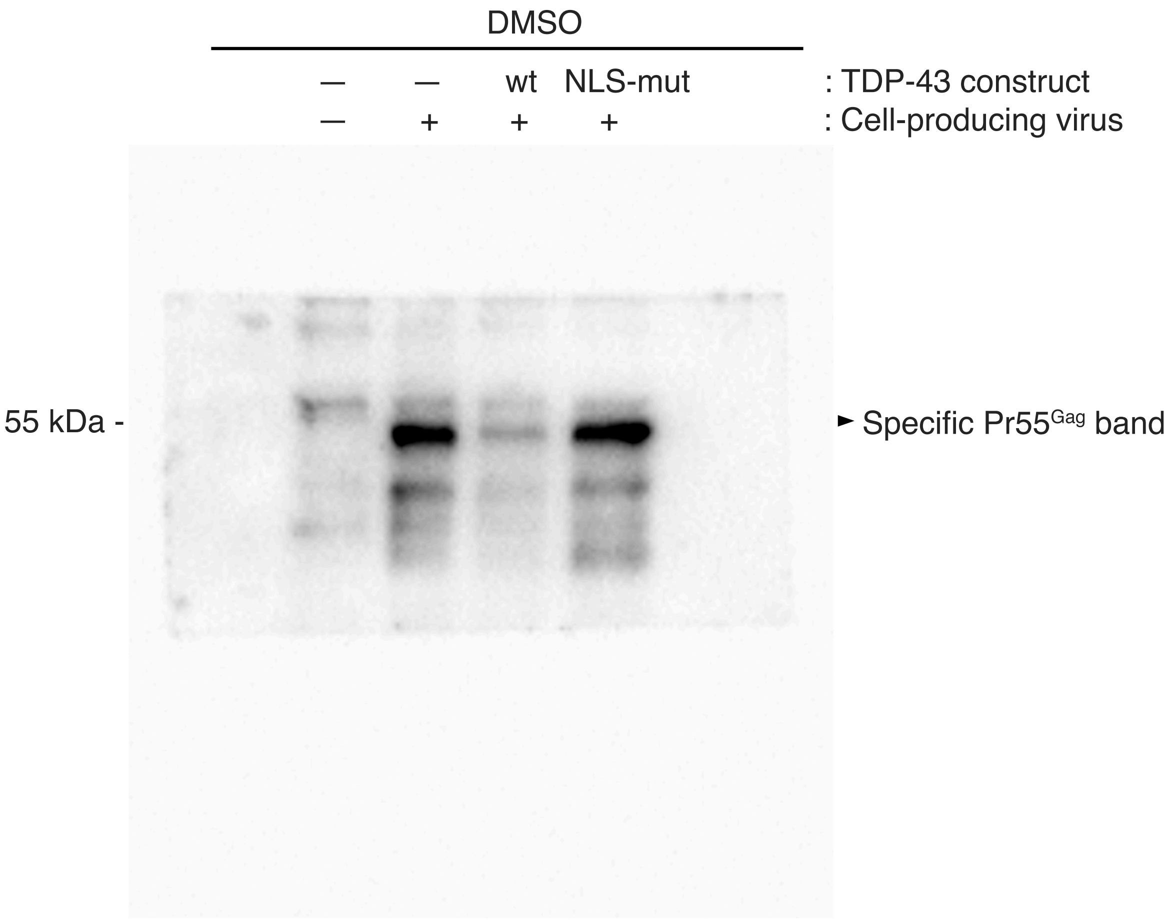

Figure S5. Replicate 1 Vif complete gel   Western-blot associated with Figure 5A
Cabrera-Rodríguez, R., *et al.*

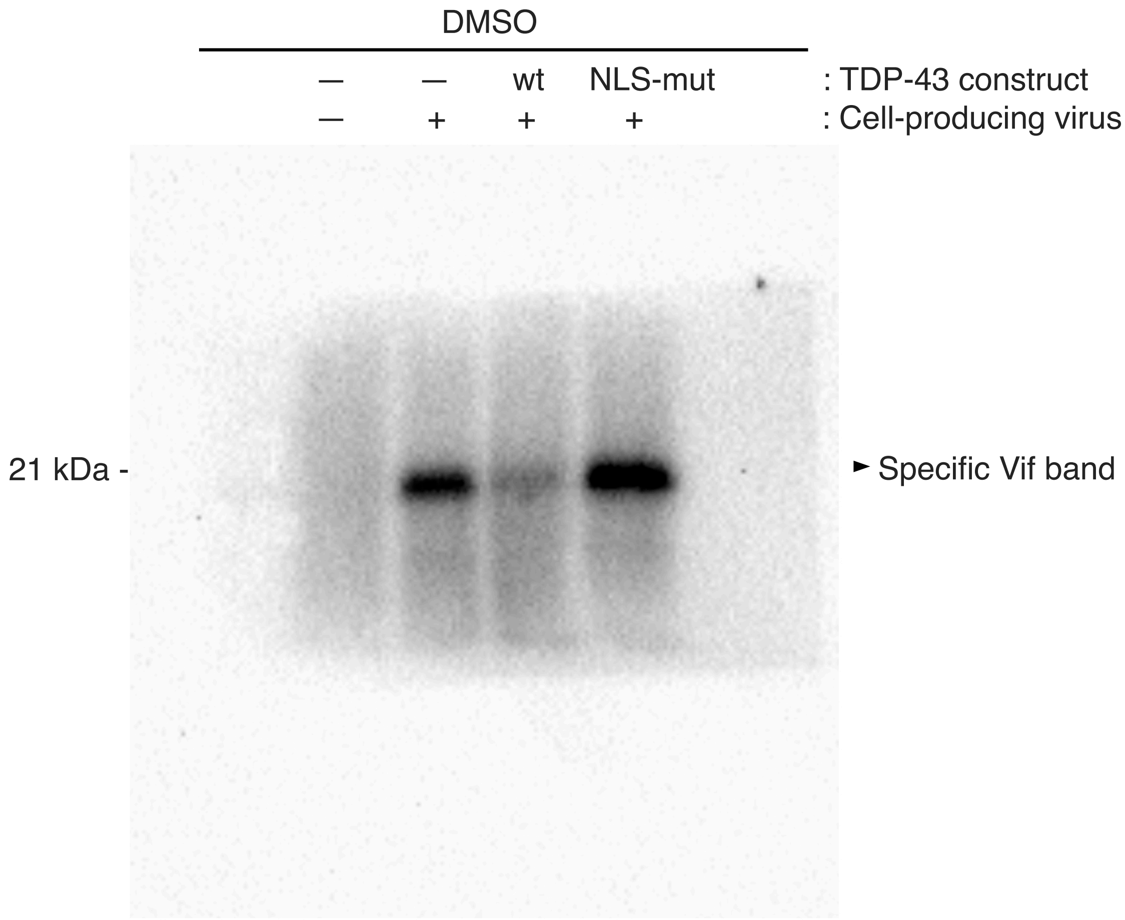

Figure S5. Replicate 1 Total α-tubulin complete gel Western-blot associated with Figure 5A

Cabrera-Rodríguez, R., *et al.*

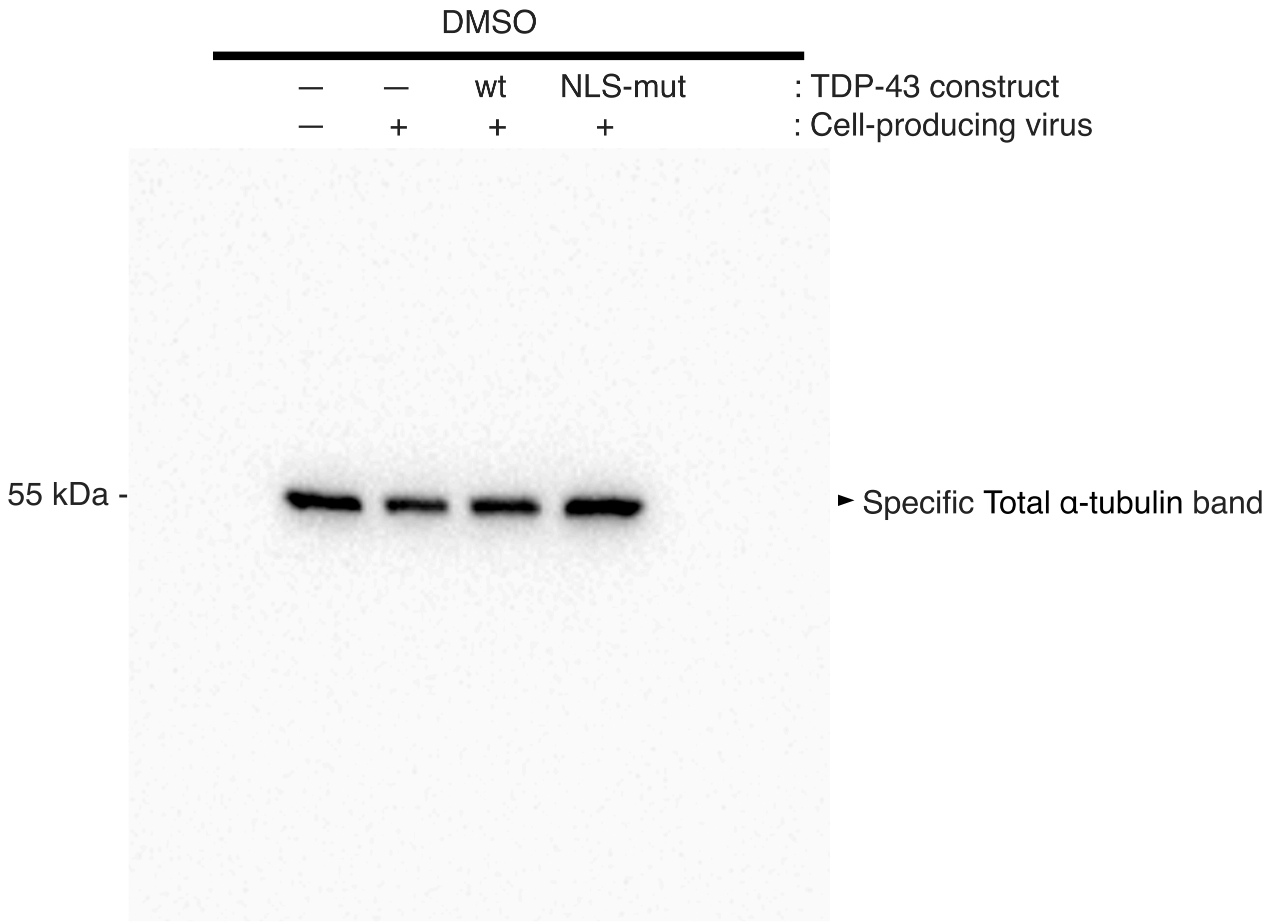

Figure S5. Replicate 1 HDAC6 complete gel Western-blot associated with Figure 5A
Cabrera-Rodríguez, R., *et al.*

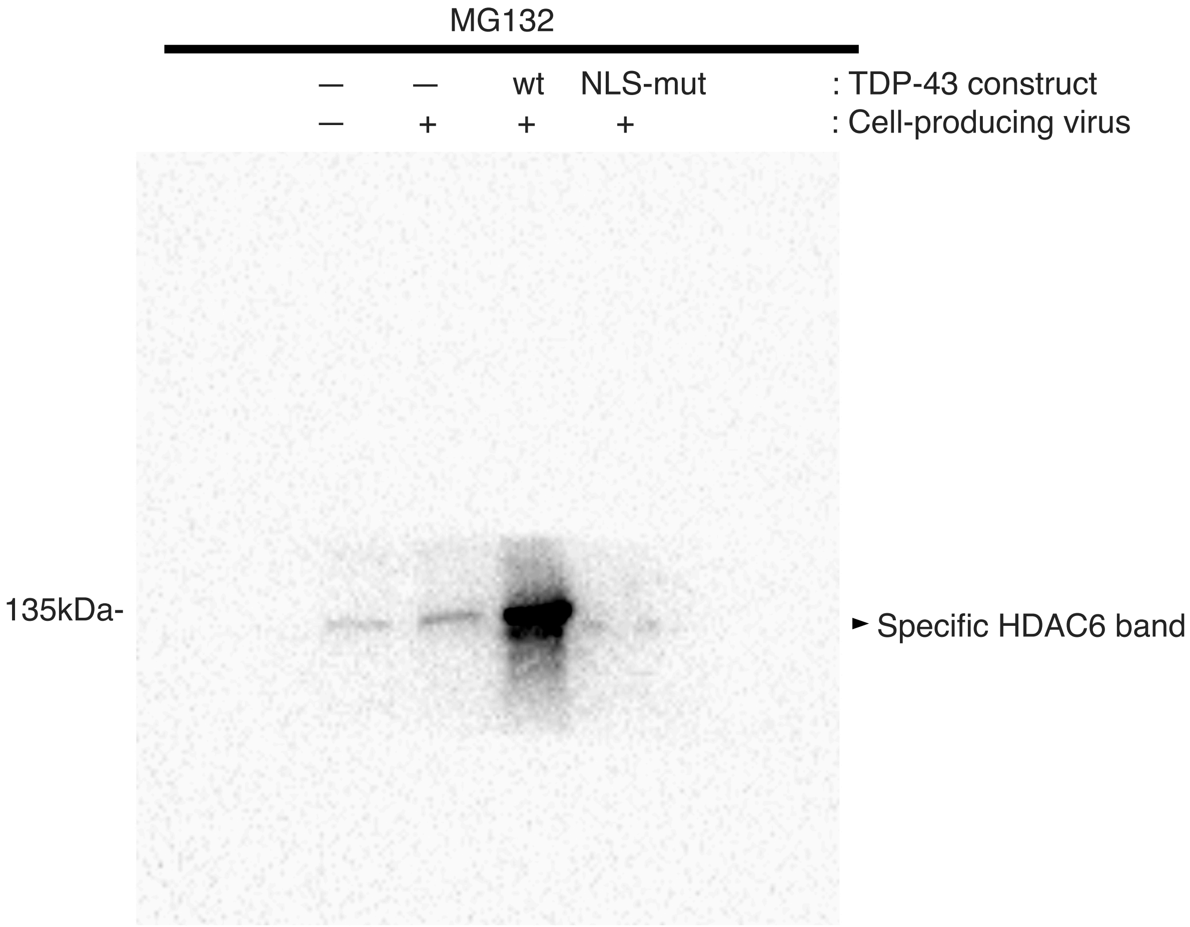

Figure S5. Replicate 1 p62 complete gel   Western-blot associated with Figure 5A
Cabrera-Rodríguez, R., *et al.*

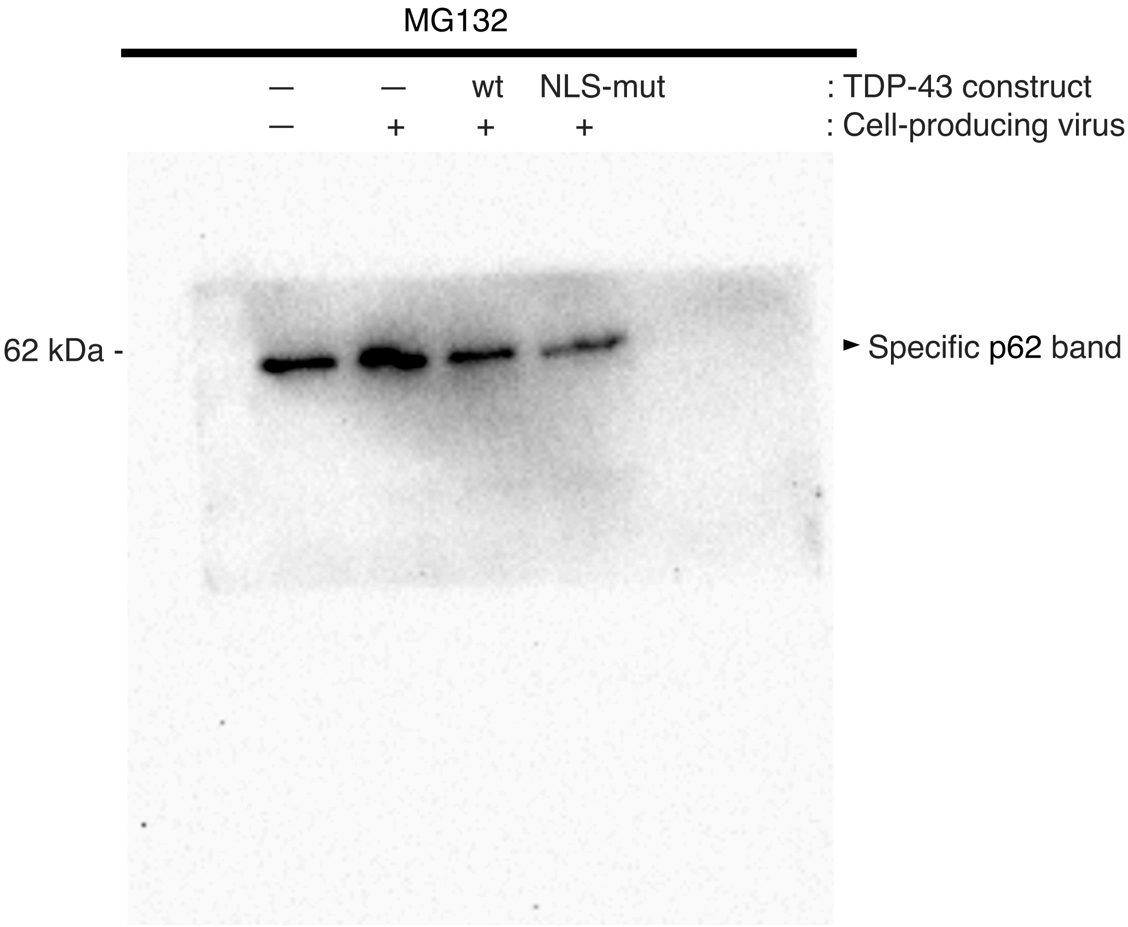

Figure S5. Replicate 1 TDP-43 complete gel Western-blot associated with Figure 5A

Cabrera-Rodríguez, R., *et al.*

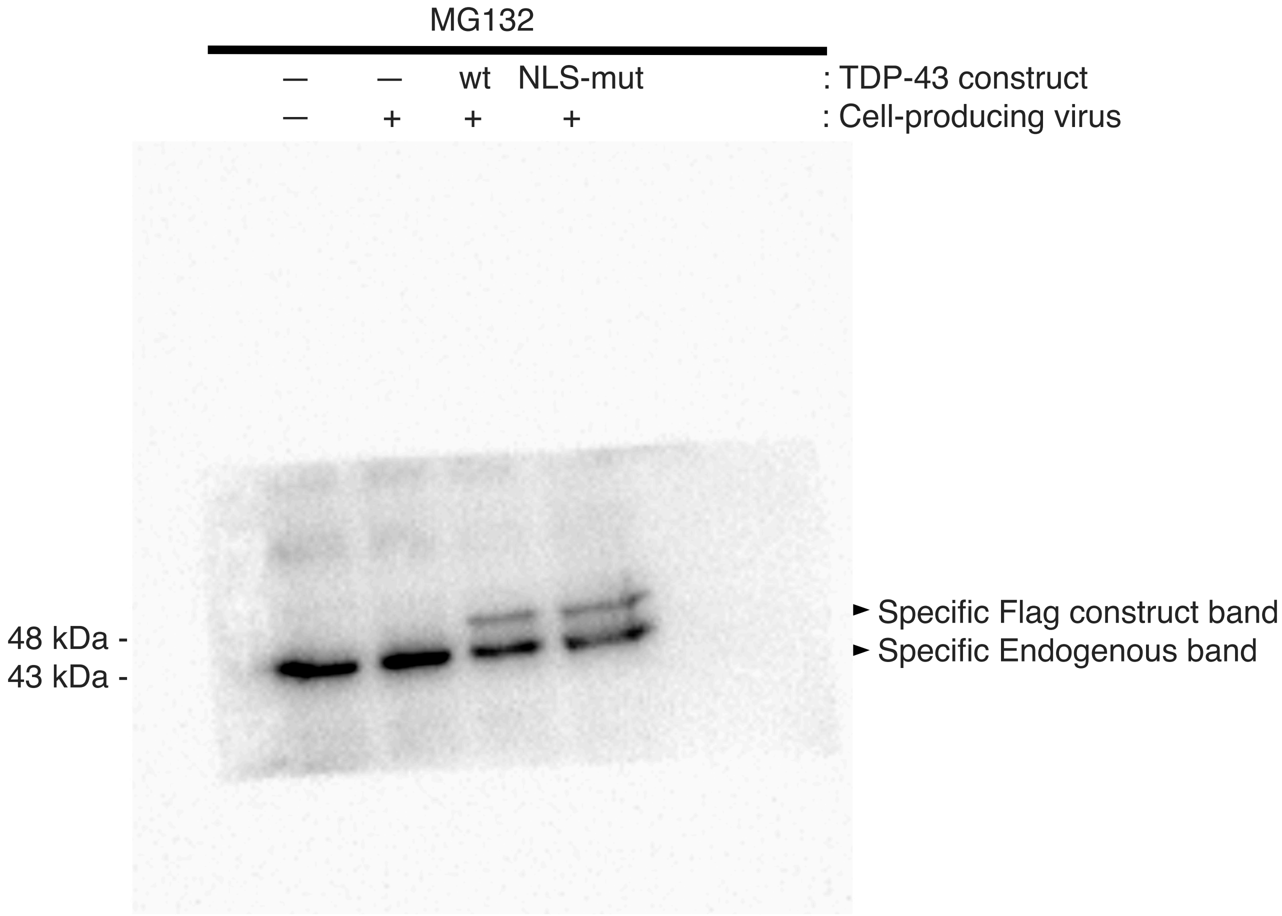

Figure S5. Replicate 1 Pr55^{Gag} complete gel   Western-blot associated with Figure 5A
Cabrera-Rodríguez, R., *et al.*

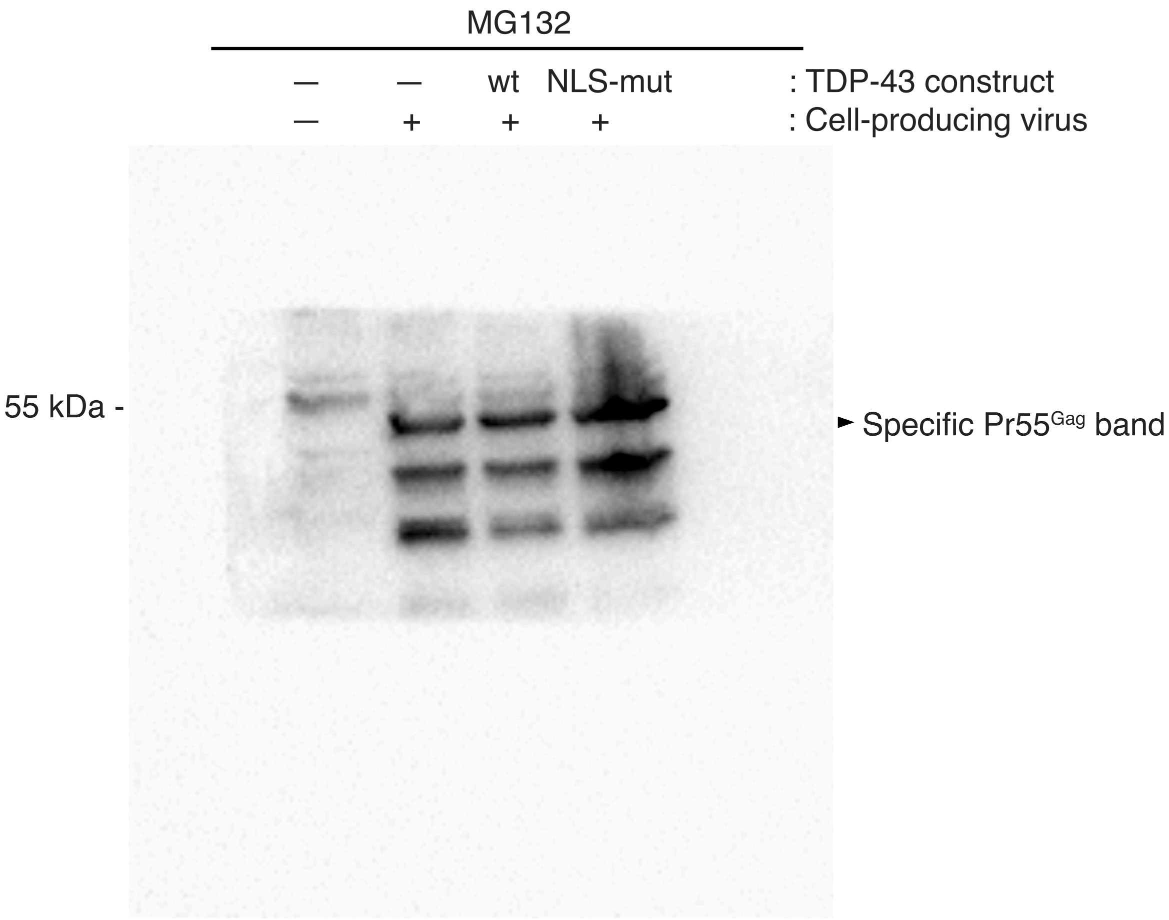

Figure S5. Replicate 1 Vif complete gel Western-blot associated with Figure 5A

Cabrera-Rodríguez, R., *et al.*

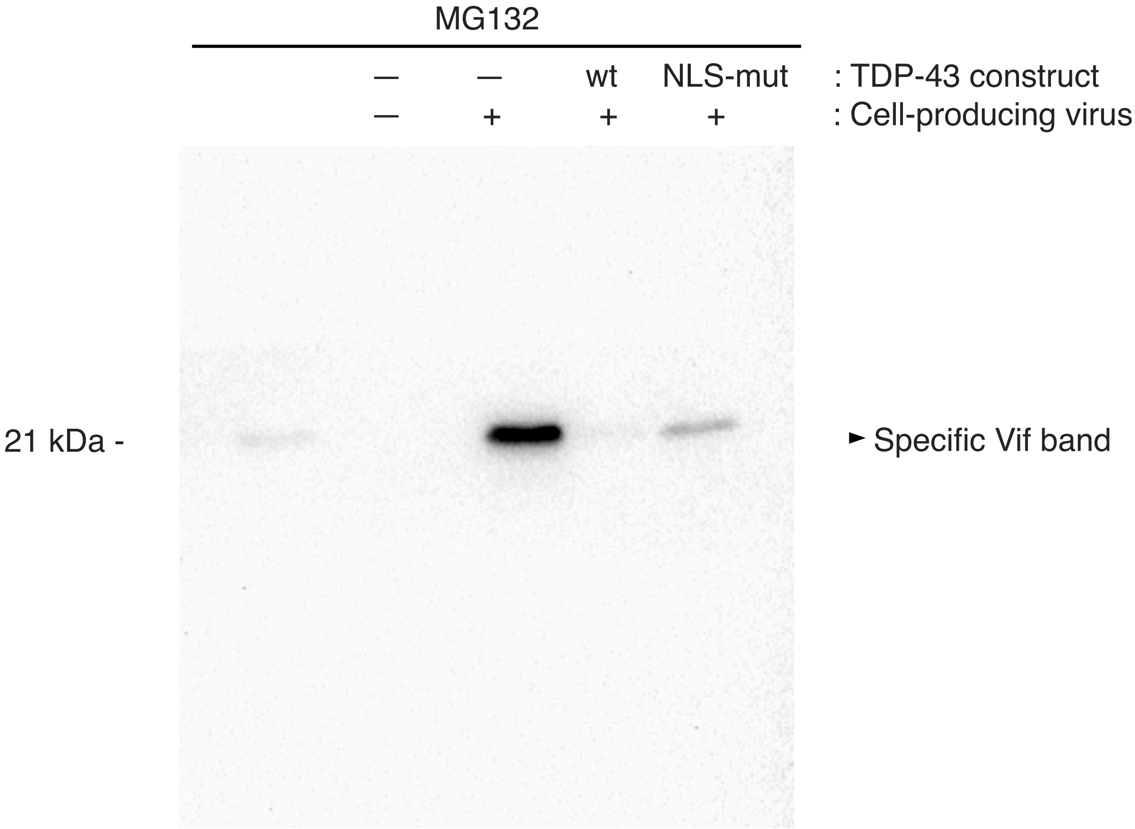

Figure S5. Replicate 1 Total α-tubulin complete gel Western-blot associated with Figure 5A
Cabrera-Rodríguez, R., *et al.*

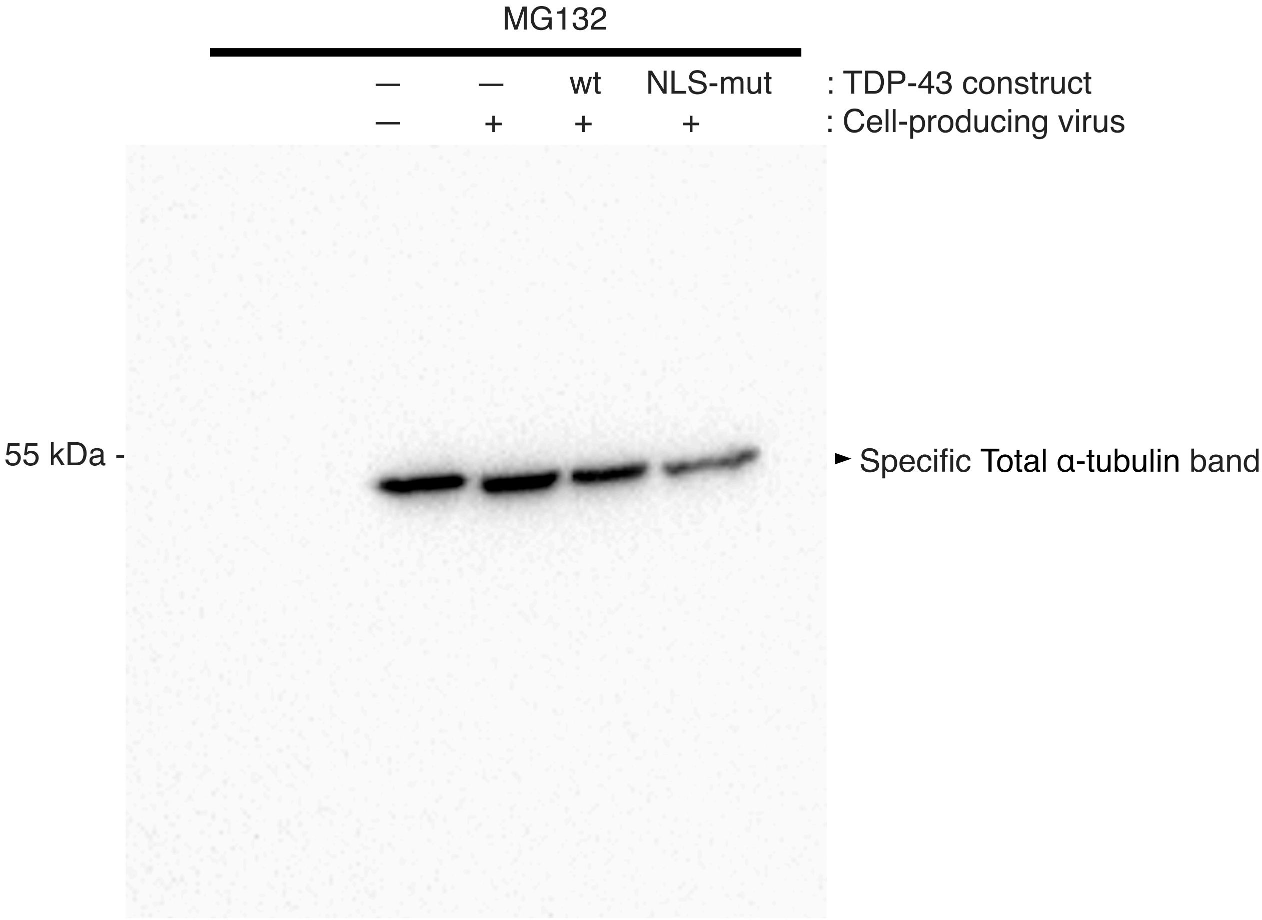

Figure S5. Replicate 2 HDAC6 complete gel   Western-blot associated with Figure 5A
Cabrera-Rodríguez, R., *et al.*

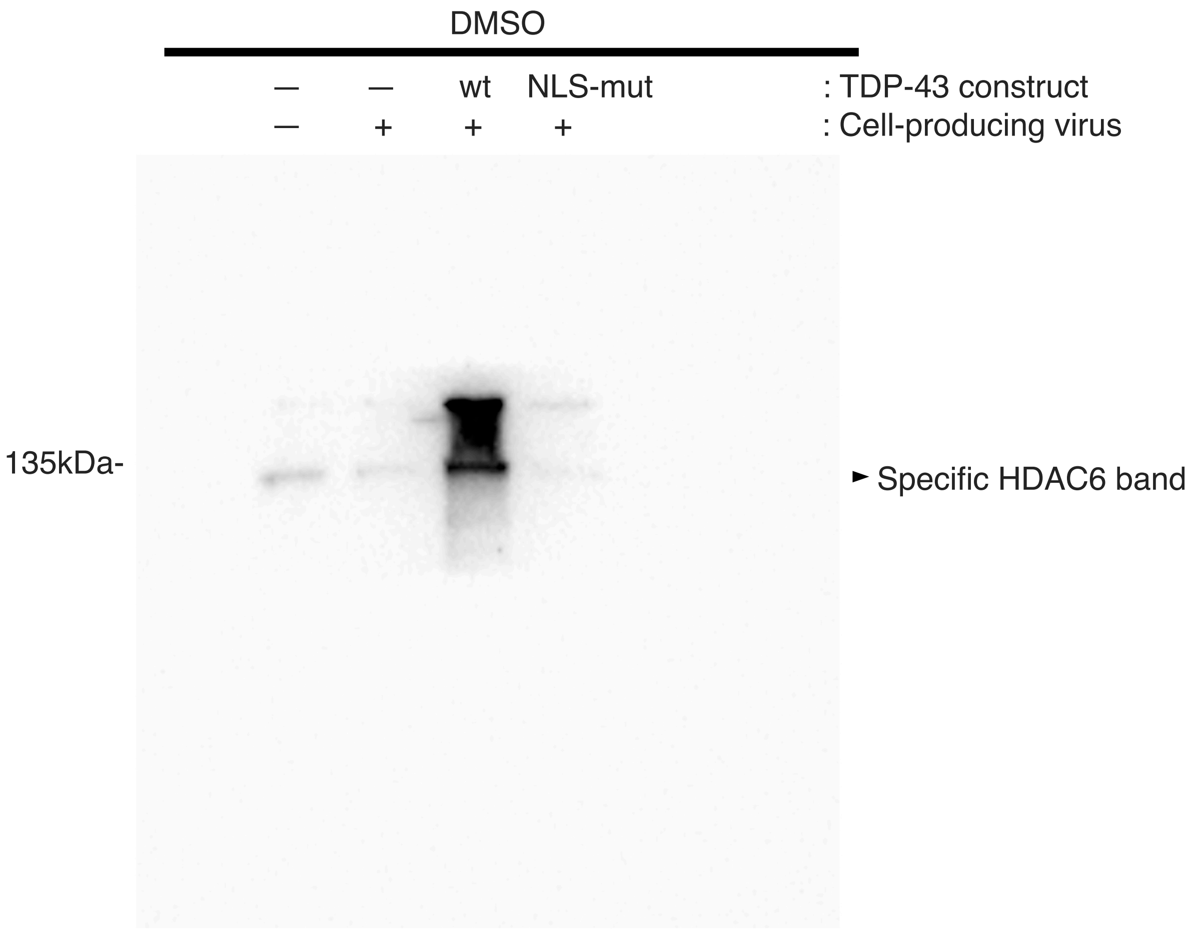

Figure S5. Replicate 2 p62 complete gel Western-blot associated with Figure 5A

Cabrera-Rodríguez, R., *et al.*

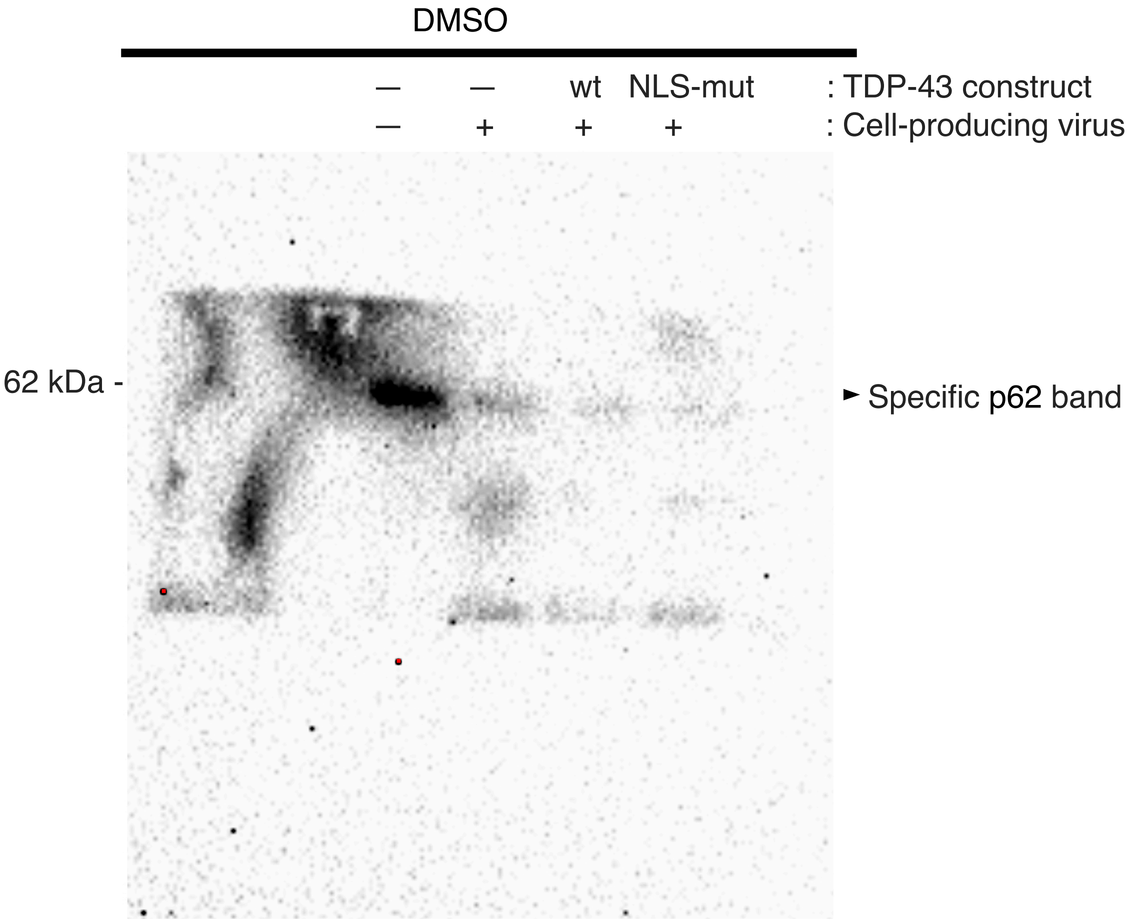

Figure S5. Replicate 2 TDP-43 complete gel   Western-blot associated with Figure 5A

Cabrera-Rodríguez, R., *et al.*

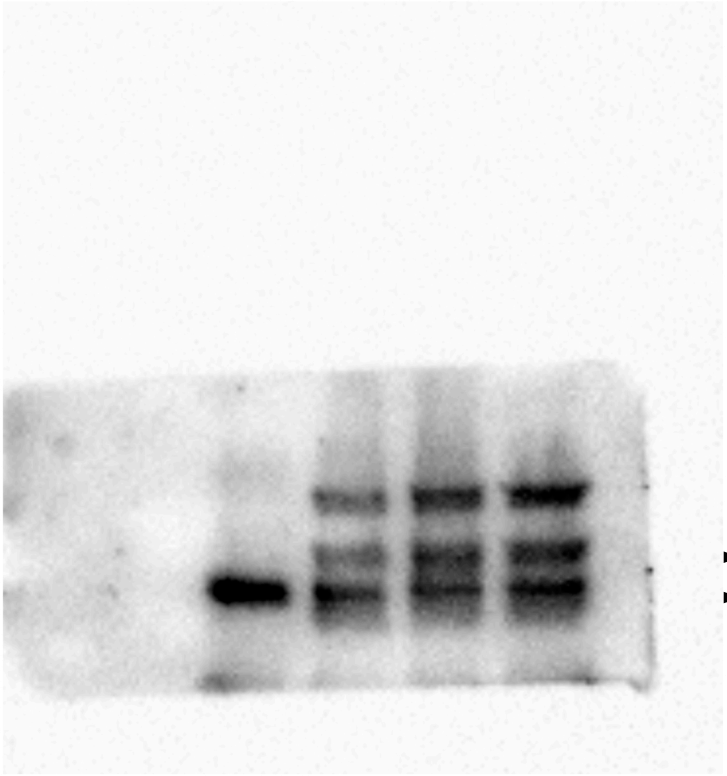

Figure S5. Replicate 2 $Pr55^{Gag}$ complete gel Western-blot associated with Figure 5A

Cabrera-Rodríguez, R., *et al.*

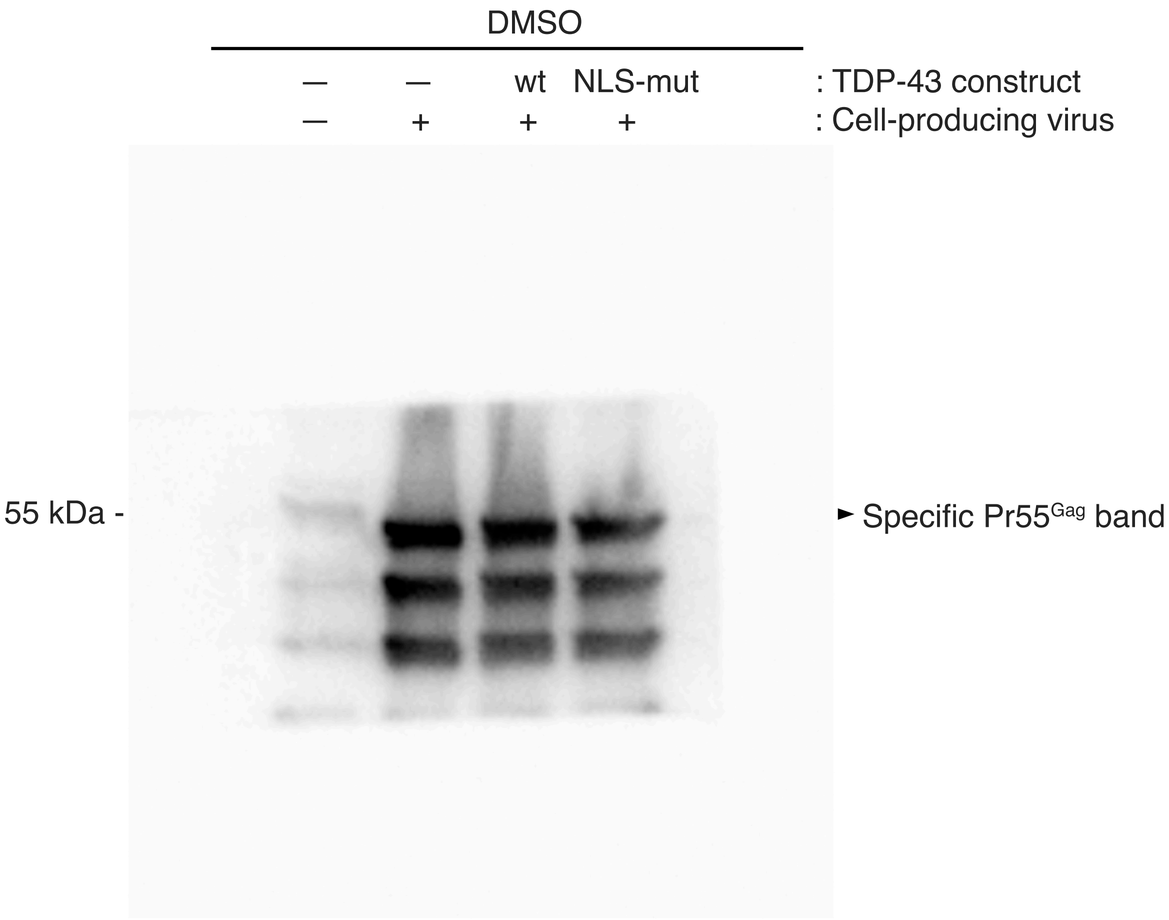

Figure S5. Replicate 2 Vif complete gel   Western-blot associated with Figure 5A
Cabrera-Rodríguez, R., *et al.*

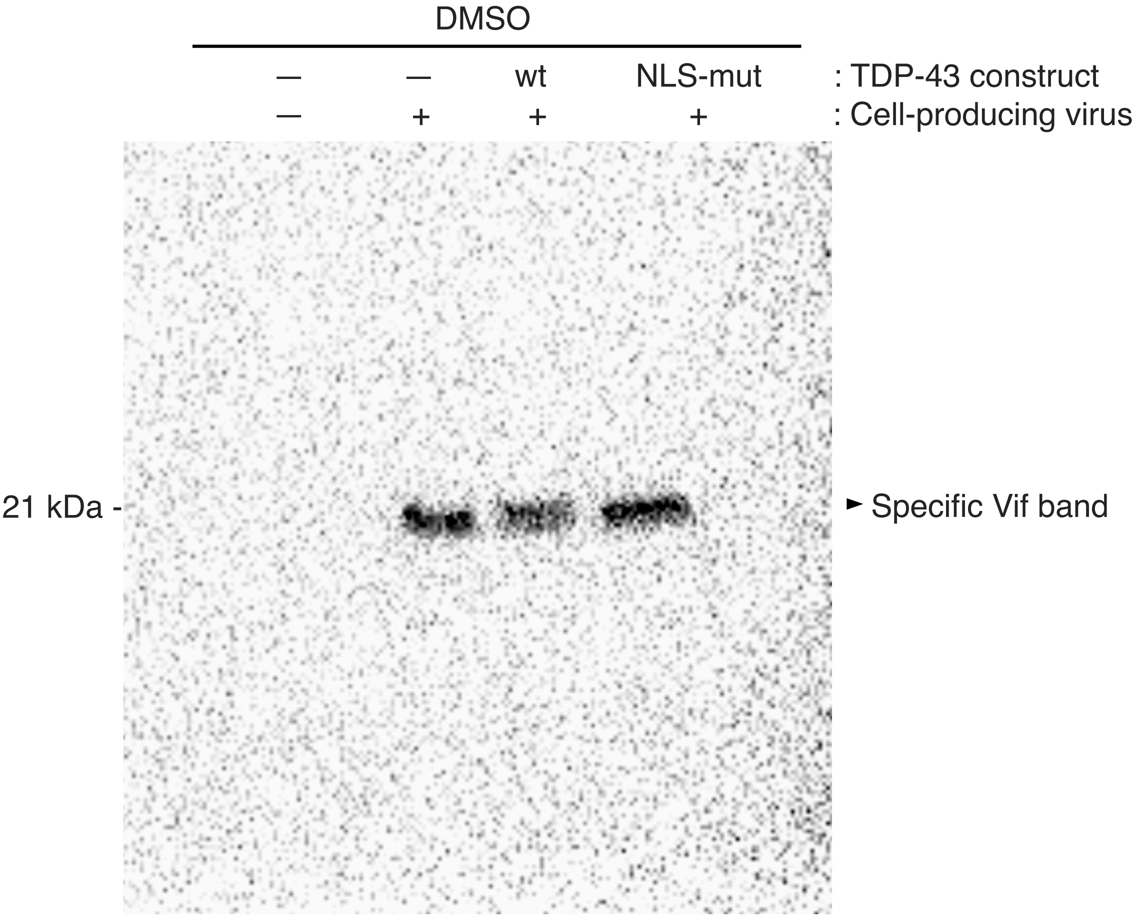

Figure S5. Replicate 2 Total α-tubulin complete gel   Western-blot associated with Figure 5A
Cabrera-Rodríguez, R., *et al.*

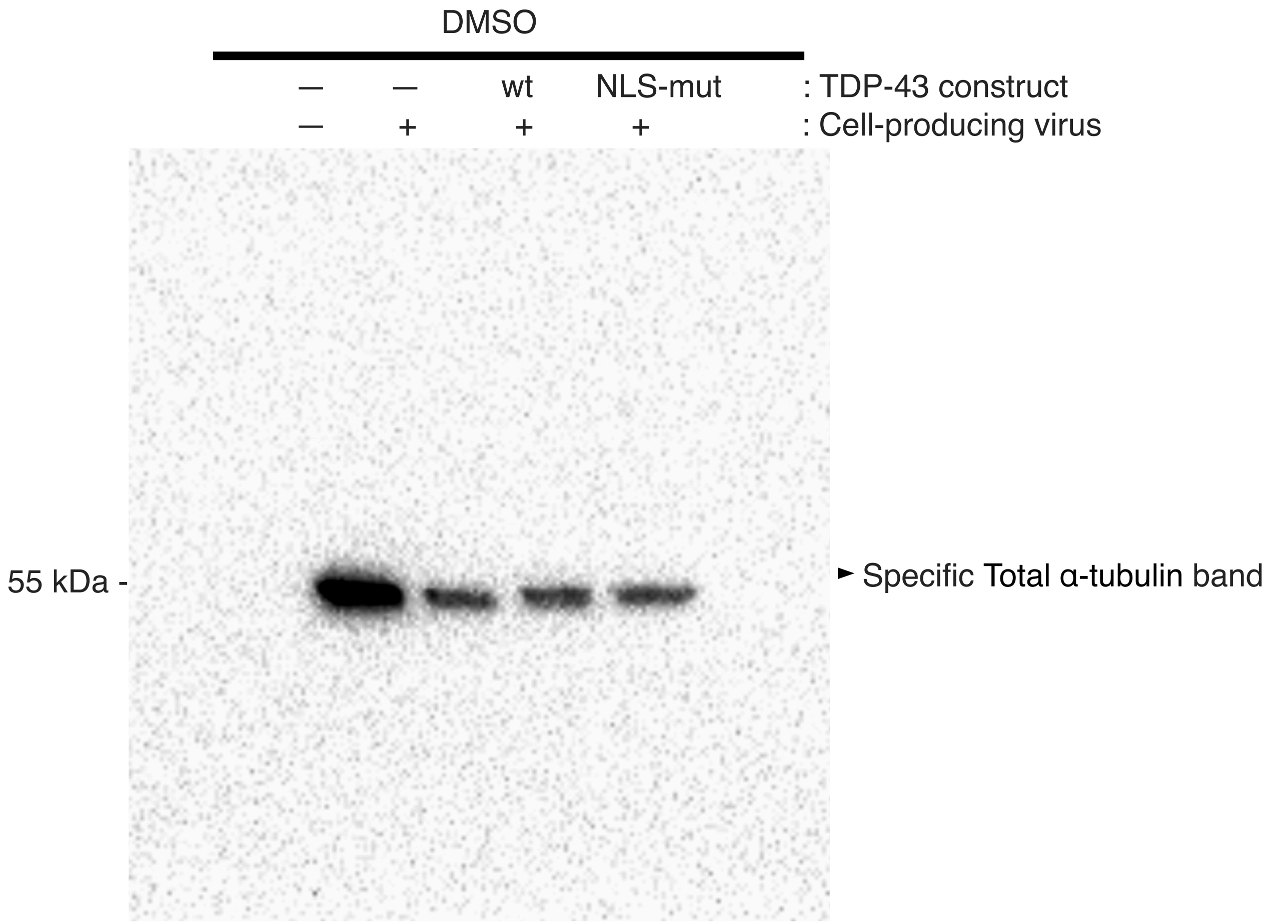

Figure S5. Replicate 2 HDAC6 complete gel Western-blot associated with Figure 5A

Cabrera-Rodríguez, R., *et al.*

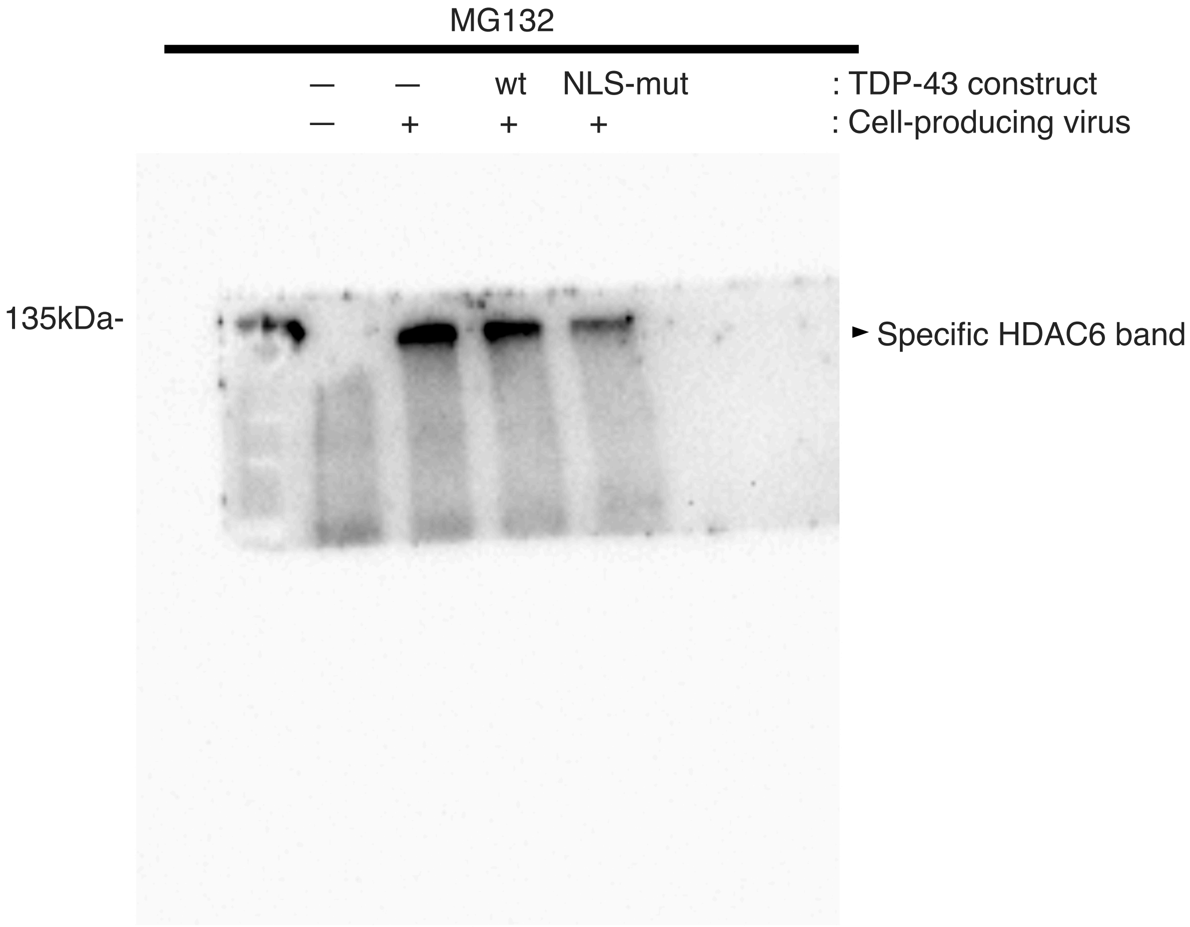

Figure S5. Replicate 2 p62 complete gel   Western-blot associated with Figure 5A

Cabrera-Rodríguez, R., *et al.*

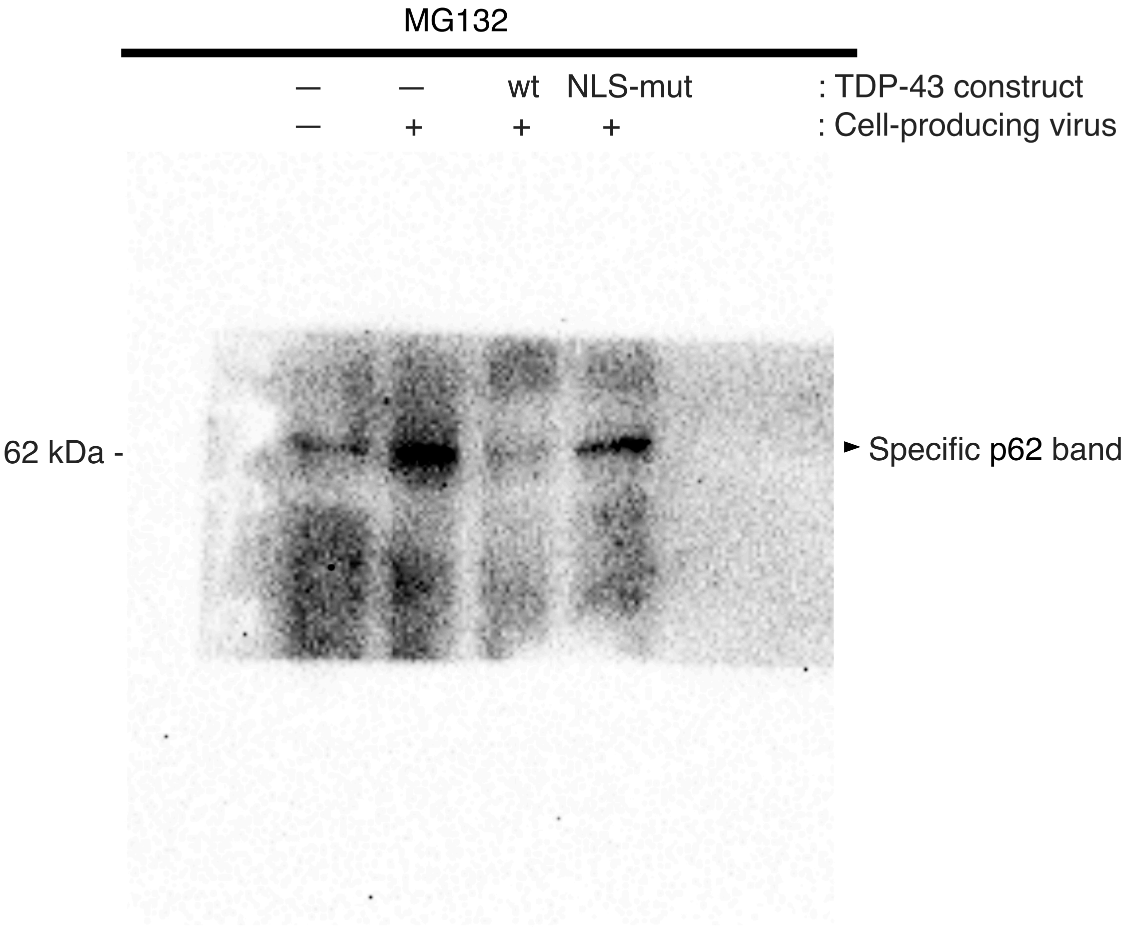

Figure S5. Replicate 2 TDP-43 complete gel Western-blot associated with Figure 5A

Cabrera-Rodríguez, R., *et al.*

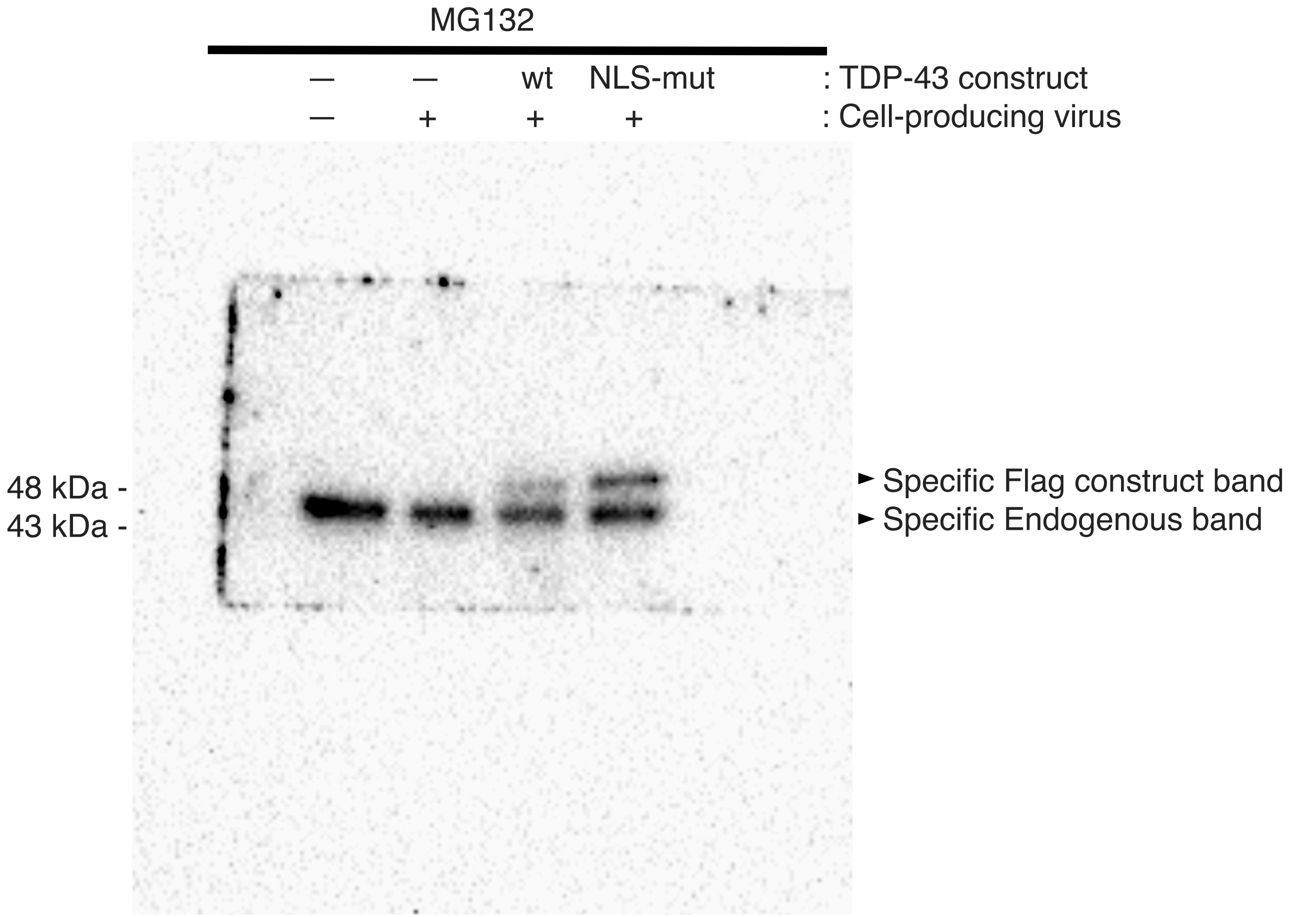

Figure S5. Replicate 2 $Pr55^{Gag}$ complete gel   Western-blot associated with Figure 5A
Cabrera-Rodríguez, R., *et al.*

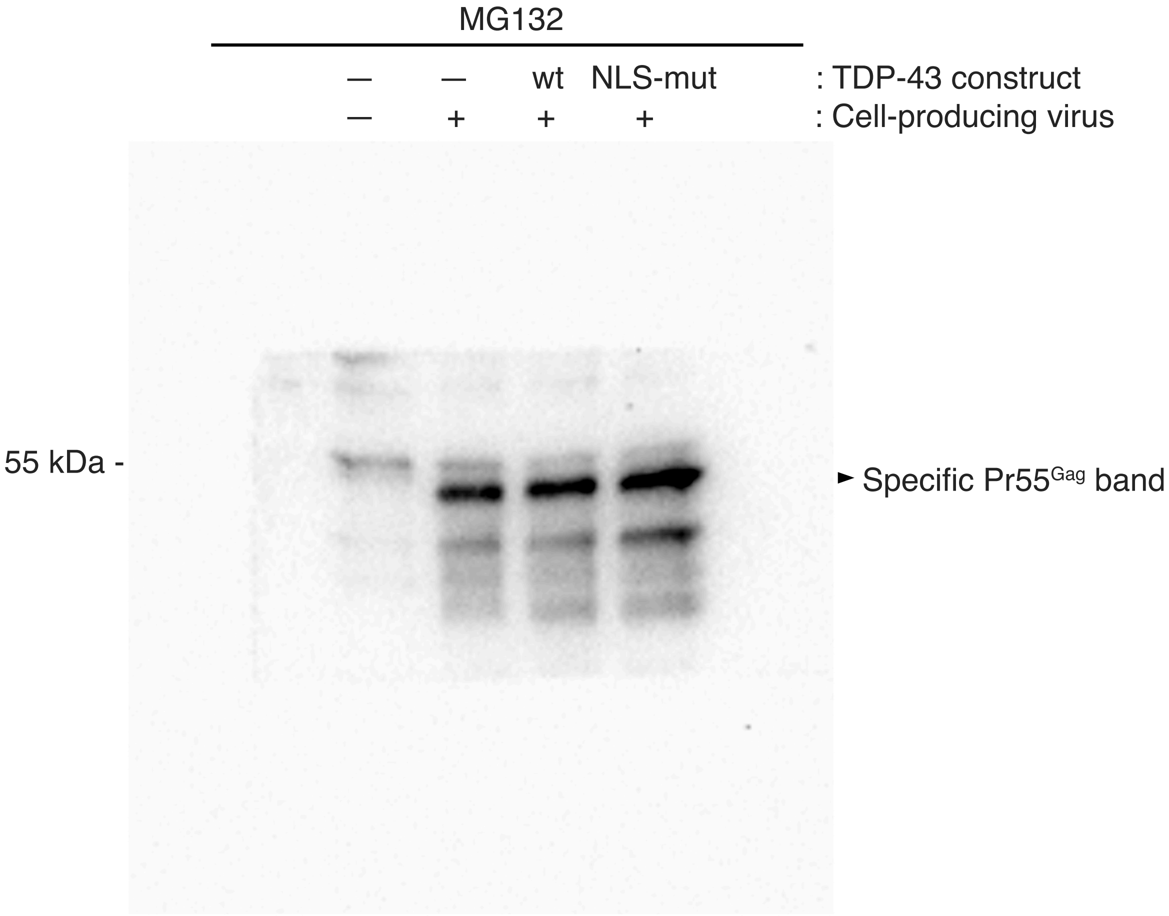

Figure S5. Replicate 2 Vif complete gel Western-blot associated with Figure 5A

Cabrera-Rodríguez, R., *et al.*

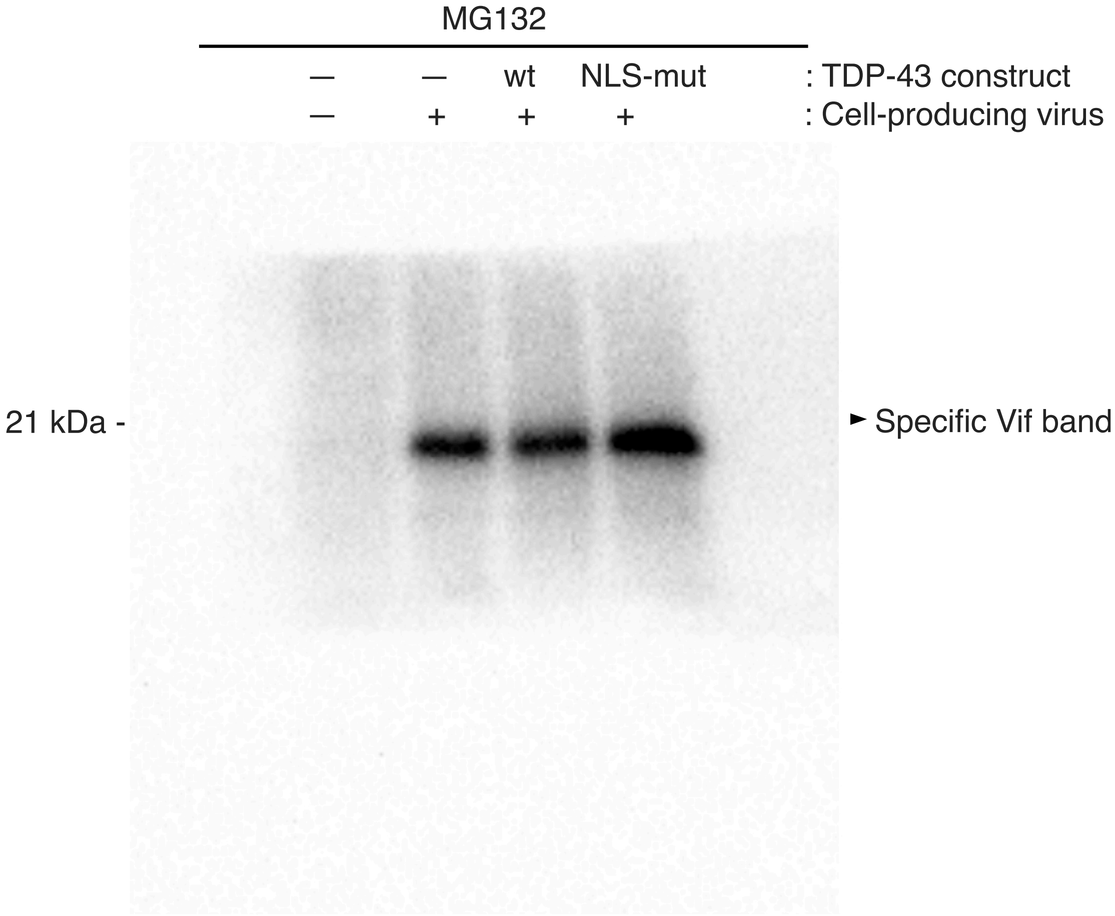

Figure S5. Replicate 2 Total α-tubulin complete gel Western-blot associated with Figure 5A

Cabrera-Rodríguez, R., *et al.*

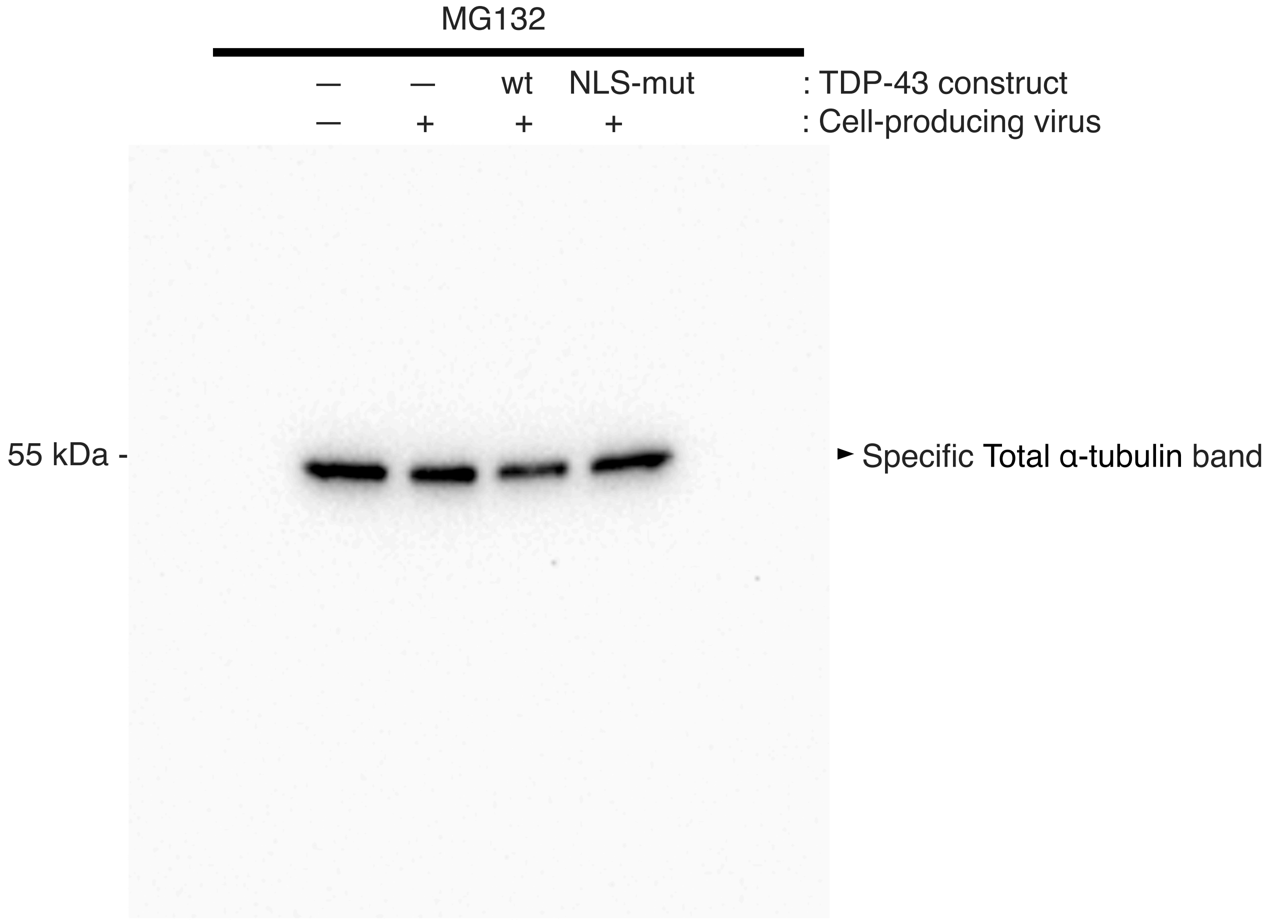

Figure S5. Replicate 3 HDAC6 complete gel Western-blot associated with Figure 5A

Cabrera-Rodríguez, R., *et al.*

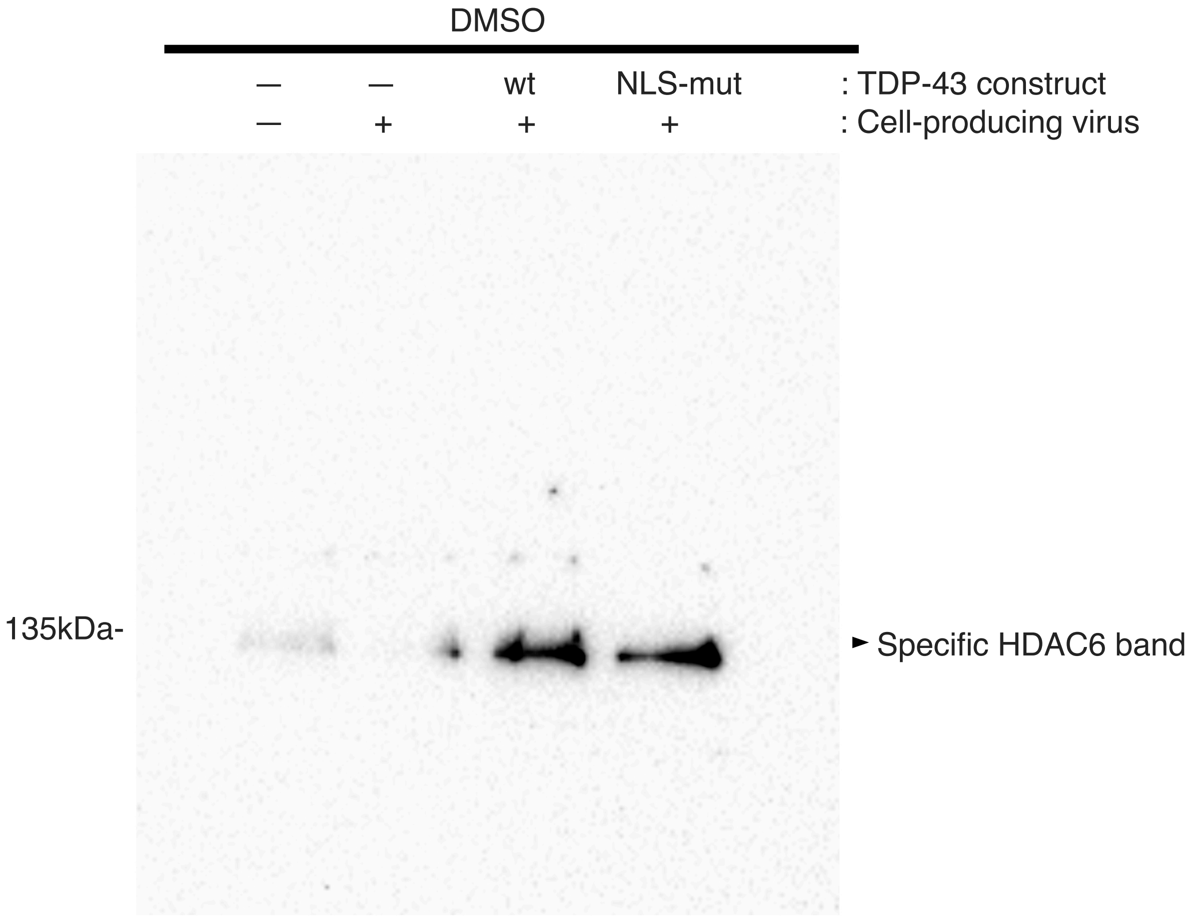

Figure S5. Replicate 3 p62 complete gel Western-blot associated with Figure 5A

Cabrera-Rodríguez, R., *et al.*

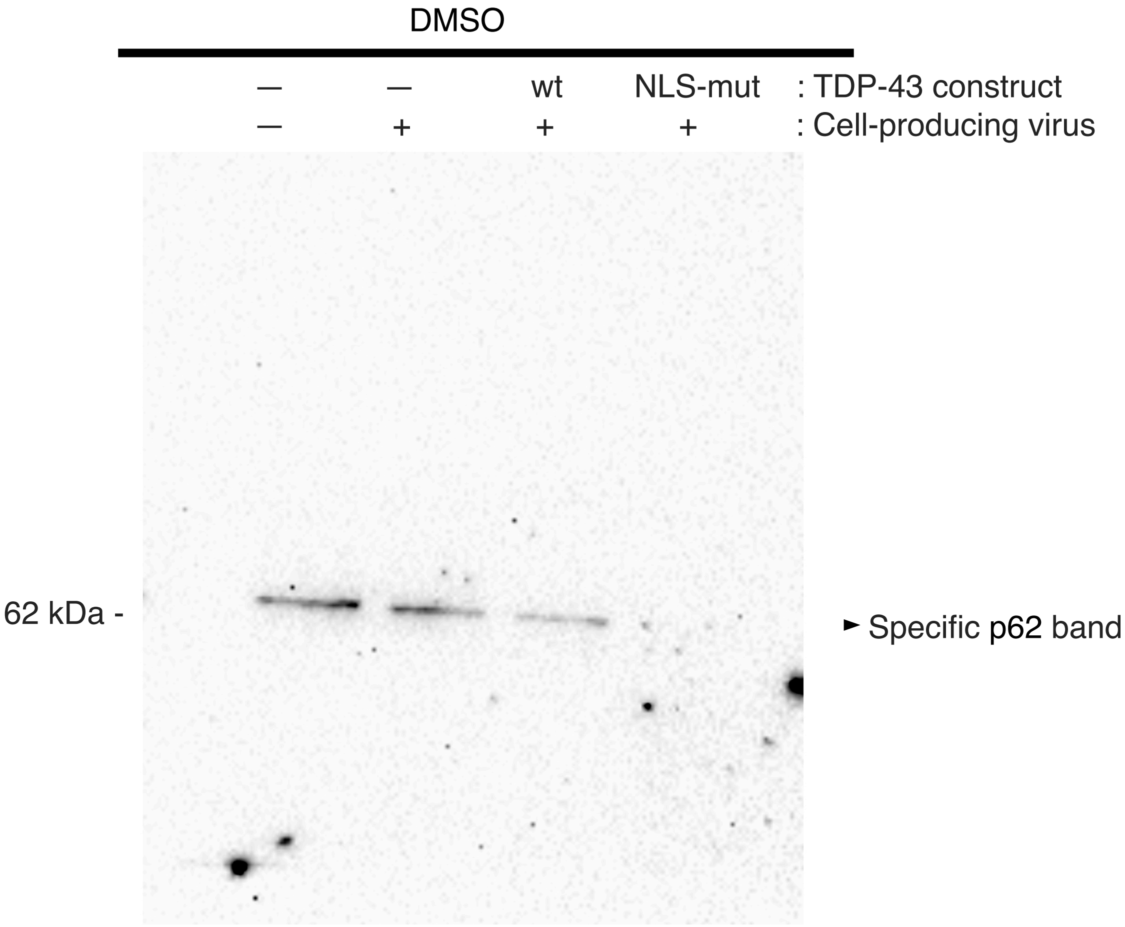

Figure S5. Replicate 3 TDP-43 complete gel   Western-blot associated with Figure 5A
Cabrera-Rodríguez, R., *et al.*

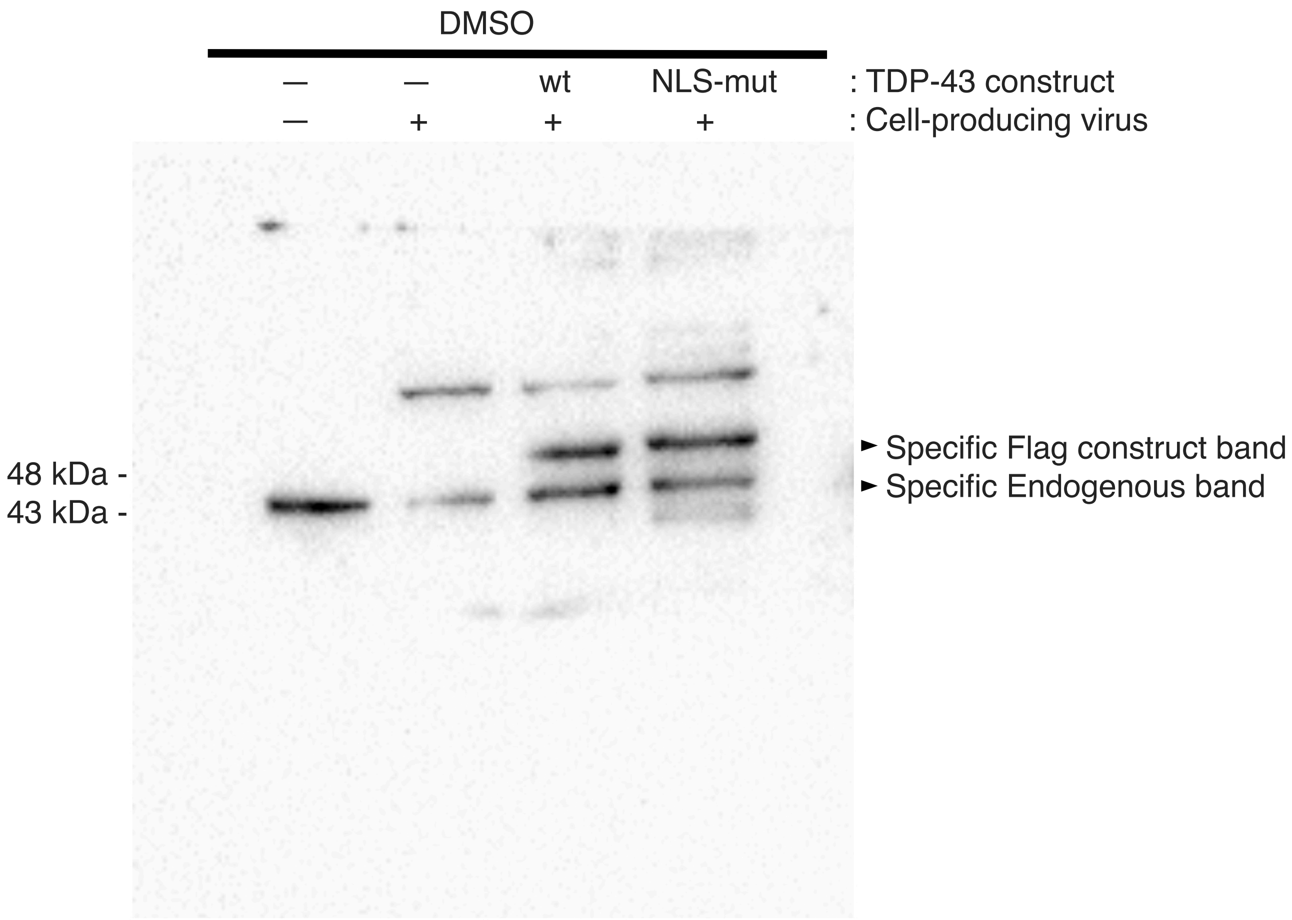

Figure S5. Replicate 3 $Pr55^{Gag}$ complete gel   Western-blot associated with Figure 5A
Cabrera-Rodríguez, R., *et al.*

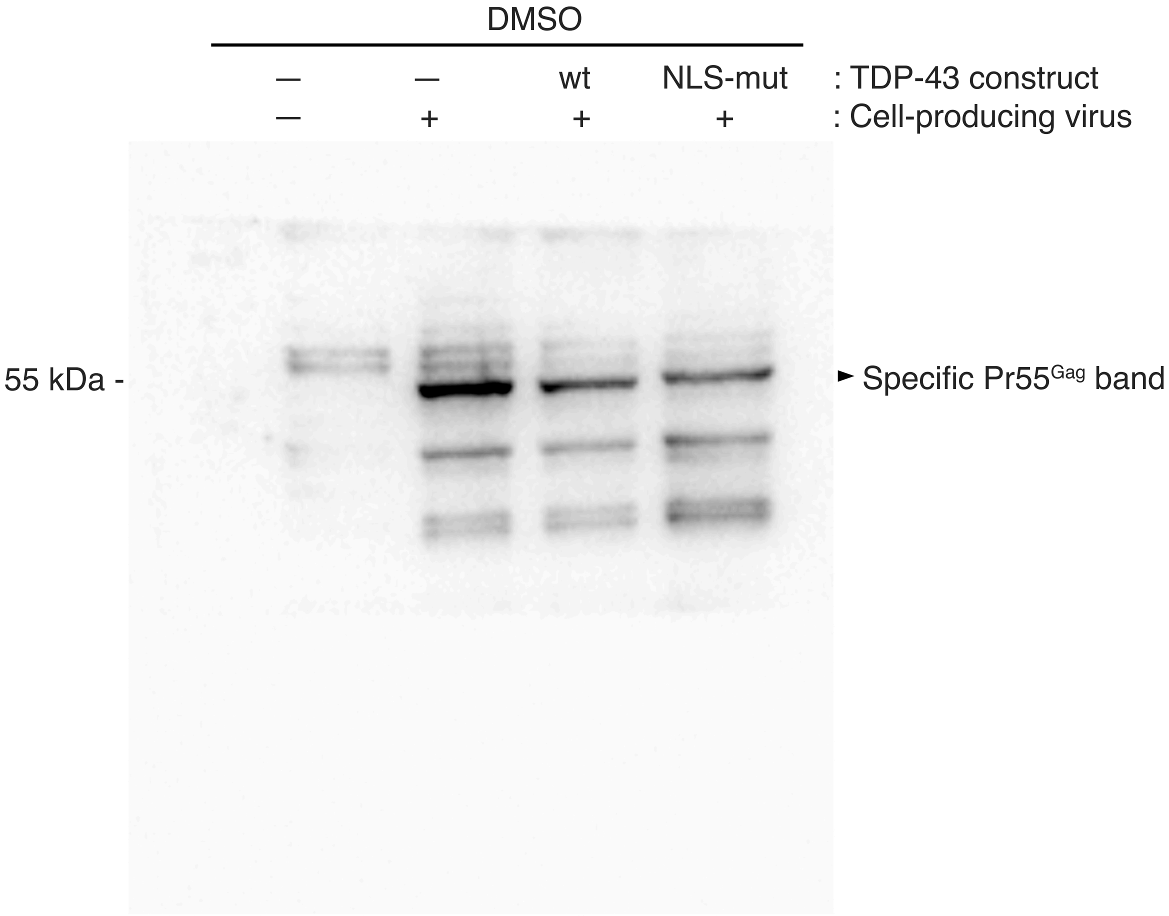

Figure S5. Replicate 3 Vif complete gel   Western-blot associated with Figure 5A
Cabrera-Rodríguez, R., *et al.*

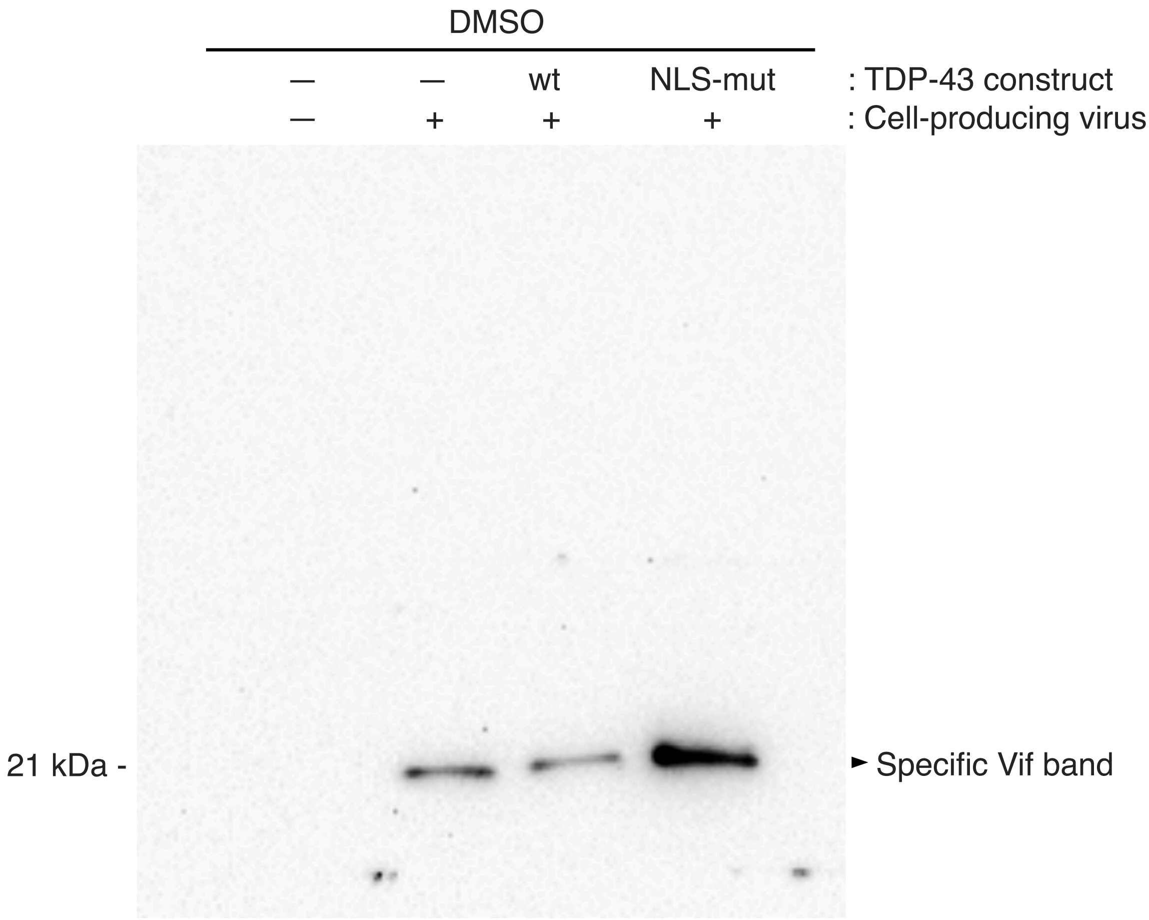

Figure S5. Replicate 3 Total α-tubulin complete gel   Western-blot associated with Figure 5A
Cabrera-Rodríguez, R., *et al.*

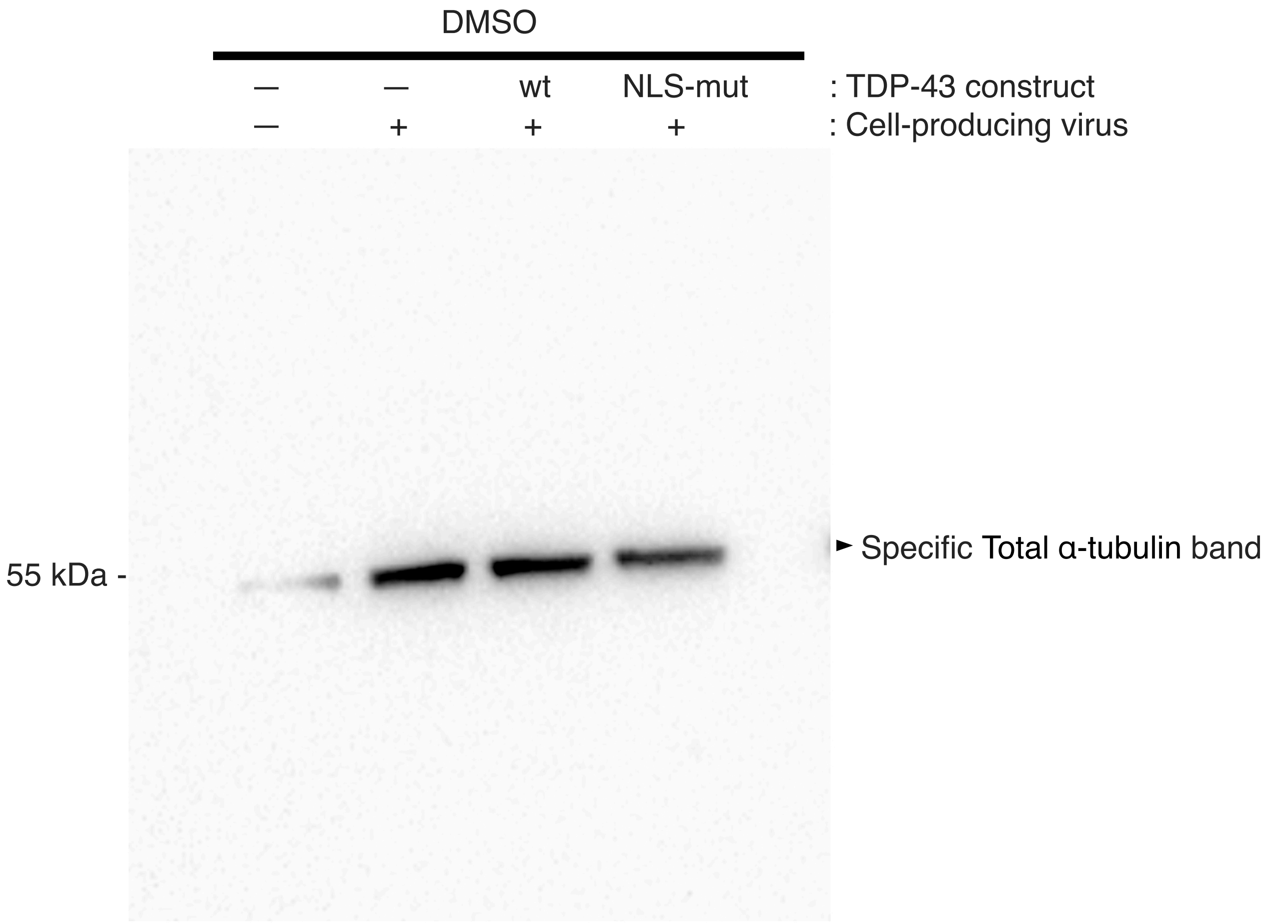

Figure S5. Replicate 3 HDAC6 complete gel   Western-blot associated with Figure 5A
Cabrera-Rodríguez, R., *et al.*

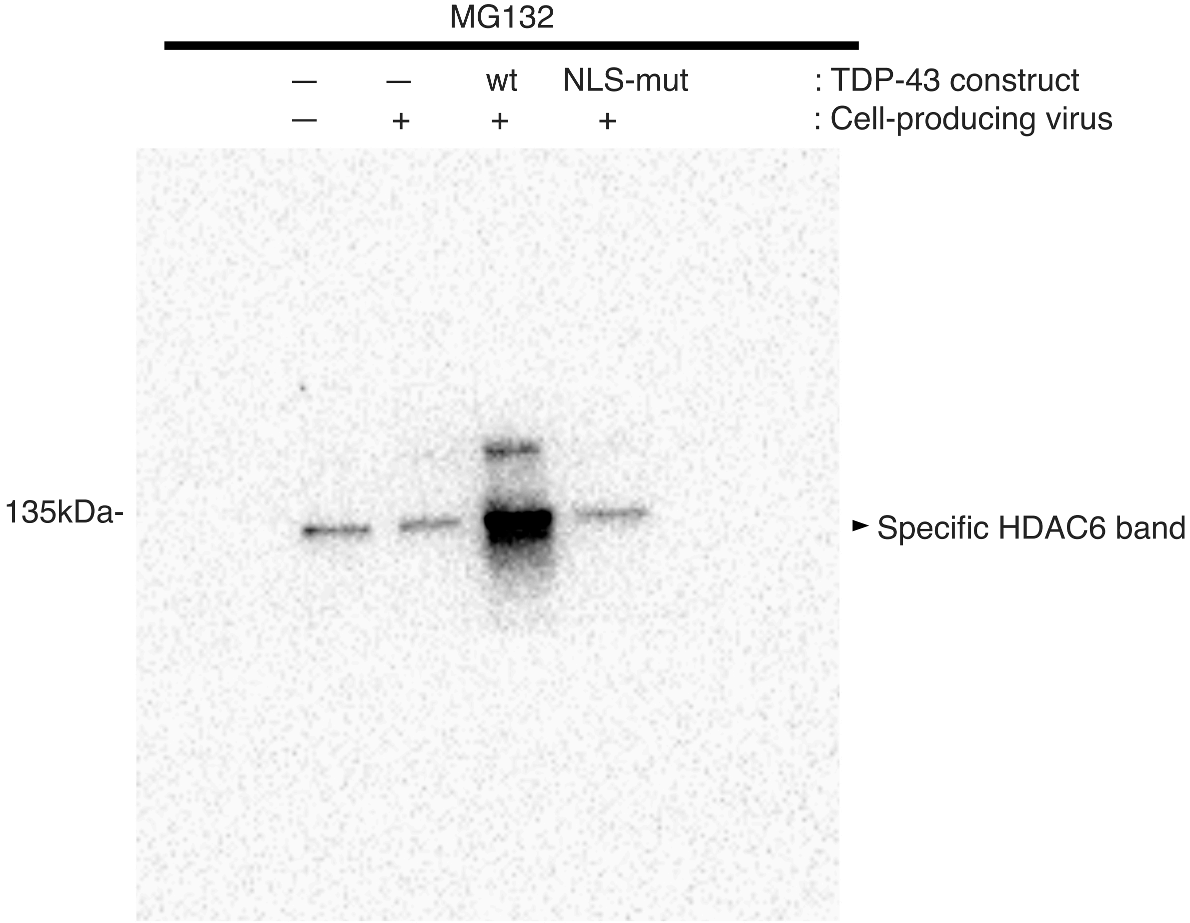

Figure S5. Replicate 3 p62 complete gel Western-blot associated with Figure 5A

Cabrera-Rodríguez, R., *et al.*

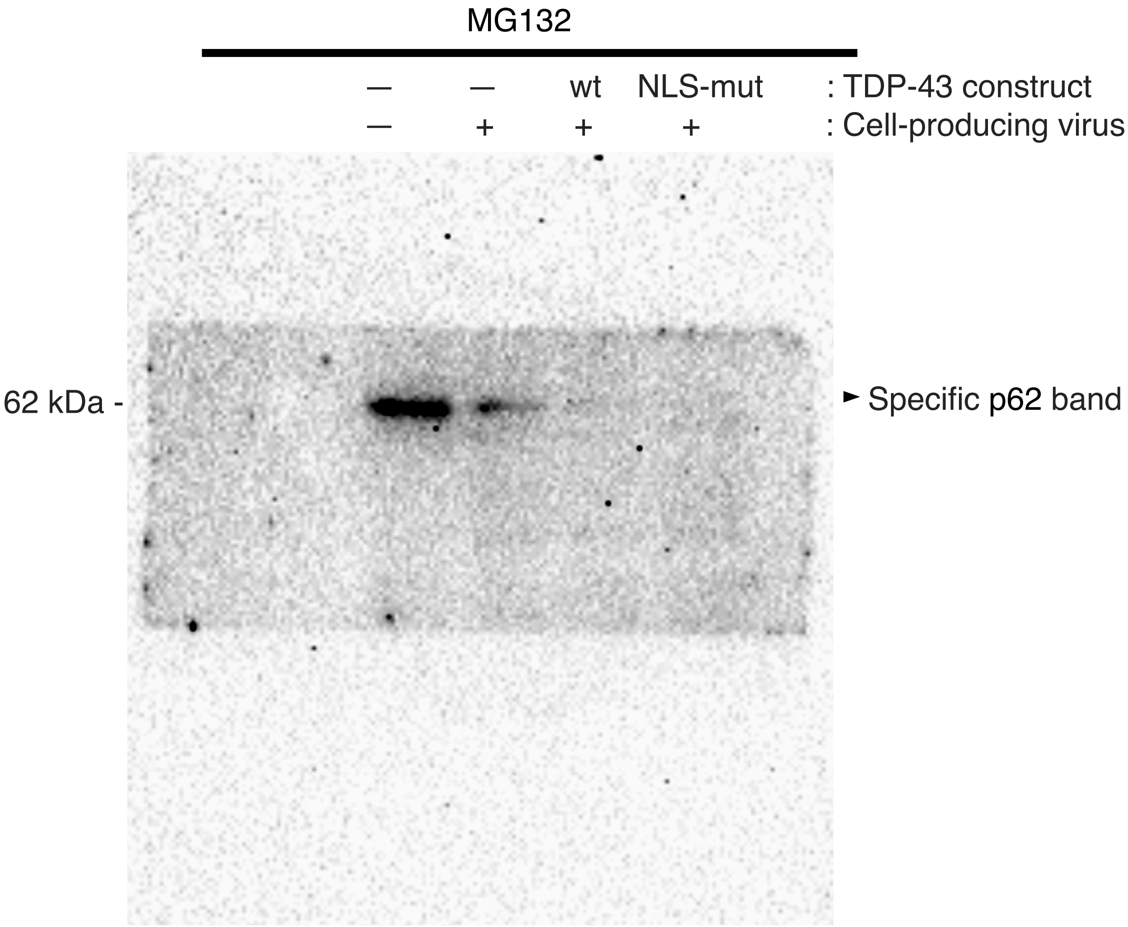

Figure S5. Replicate 3 TDP-43 complete gel   Western-blot associated with Figure 5A

Cabrera-Rodríguez, R., *et al.*

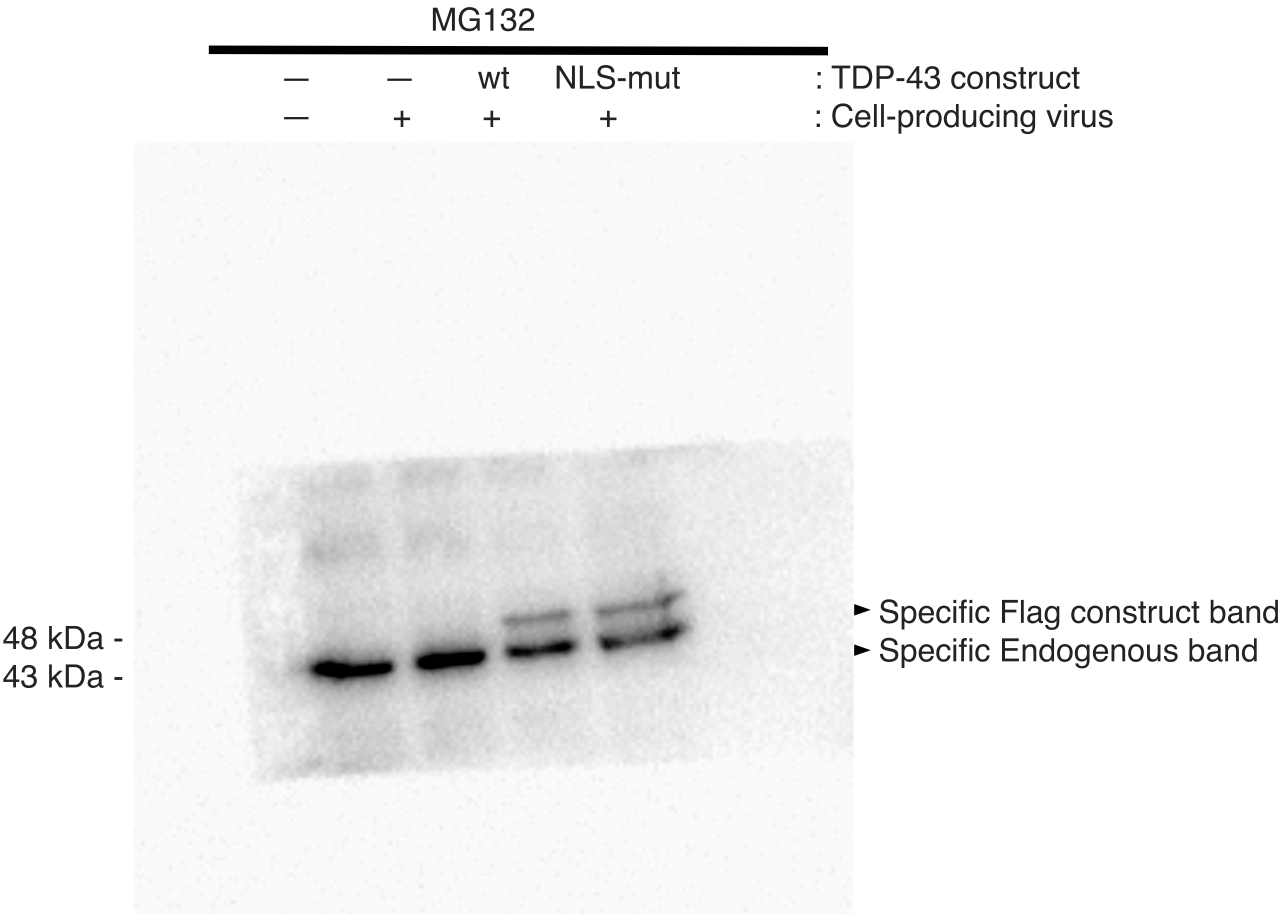

Figure S5. Replicate 3 Pr55^{Gag} complete gel Western-blot associated with Figure 5A

Cabrera-Rodríguez, R., *et al.*

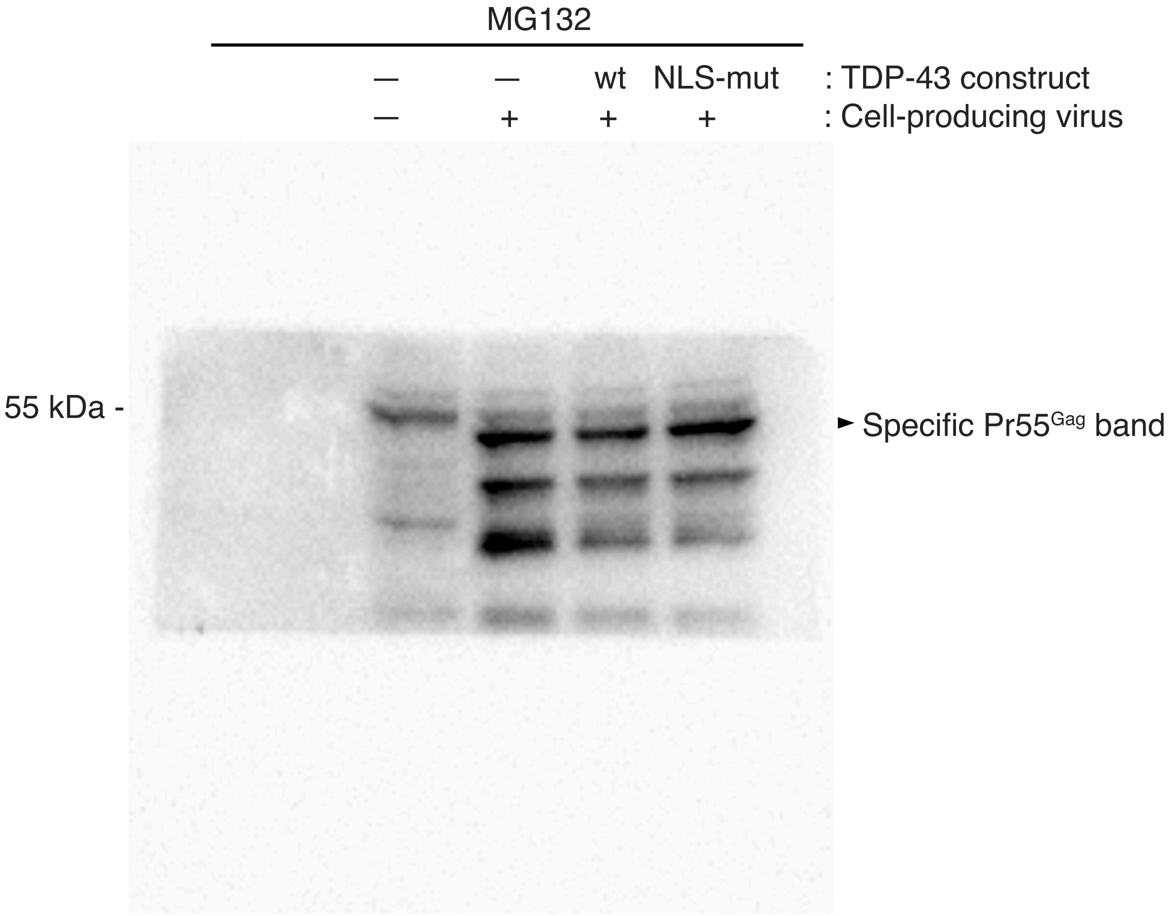

Figure S5. Replicate 3 Vif complete gel Western-blot associated with Figure 5A
Cabrera-Rodríguez, R., *et al.*

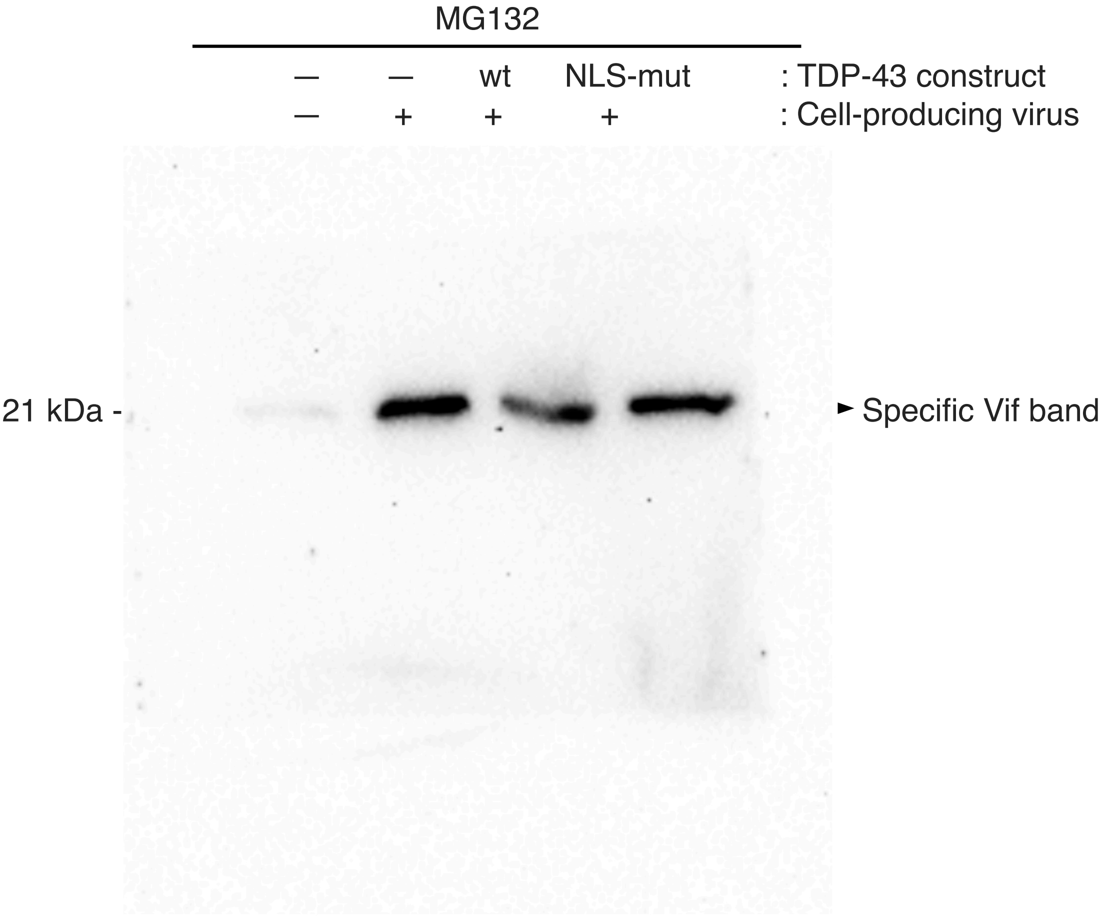

Figure S5. Replicate 3 Total α-tubulin complete gel Western-blot associated with Figure 5A

Cabrera-Rodríguez, R., *et al.*

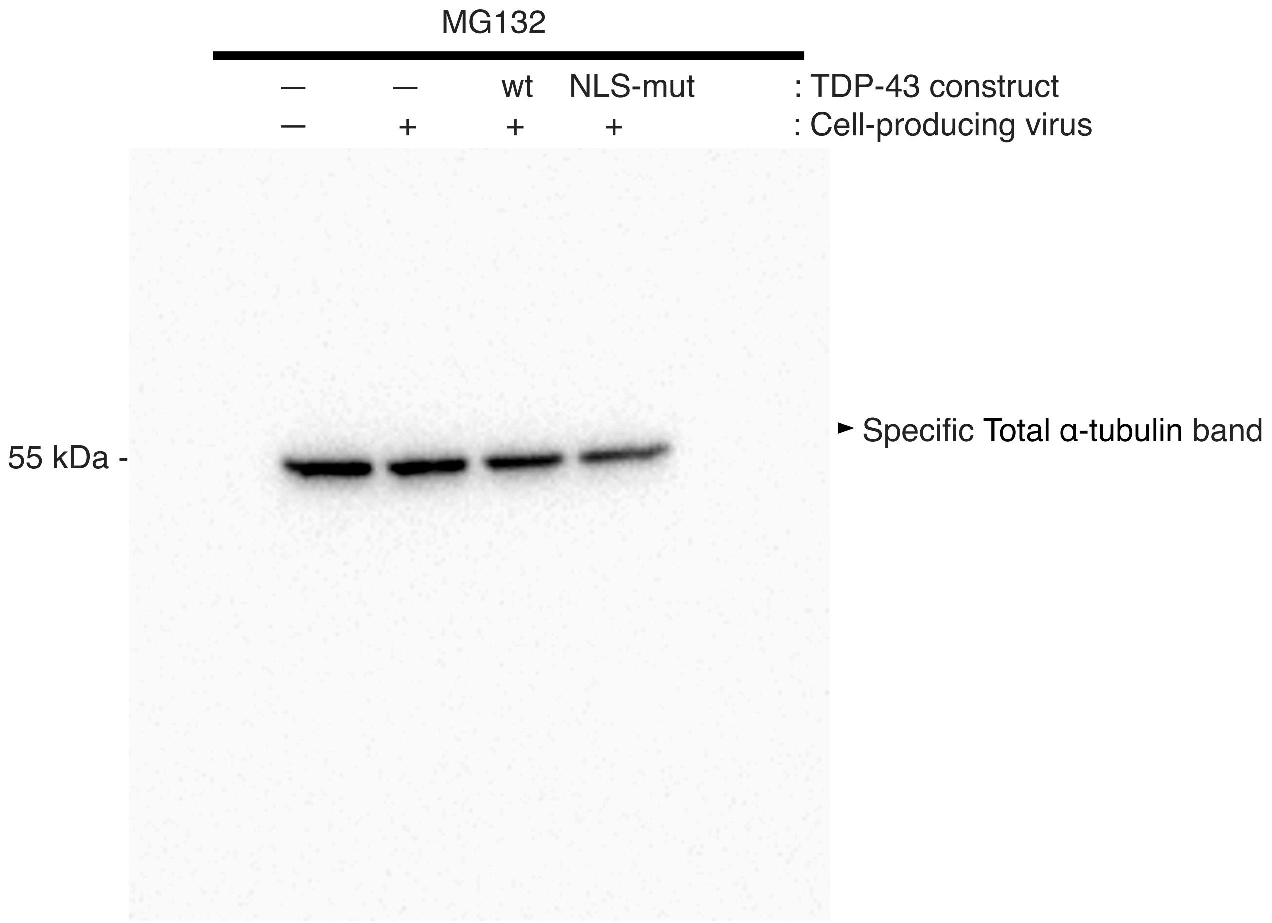

Replicate 1 as figure format

A

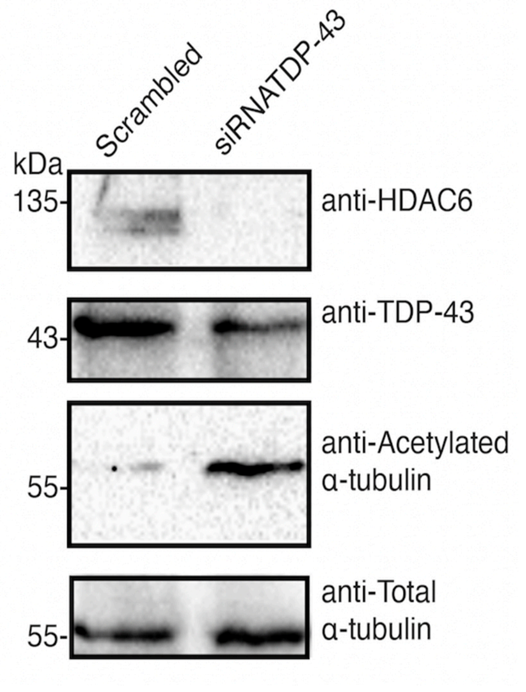

Replicate 2 as figure format

A

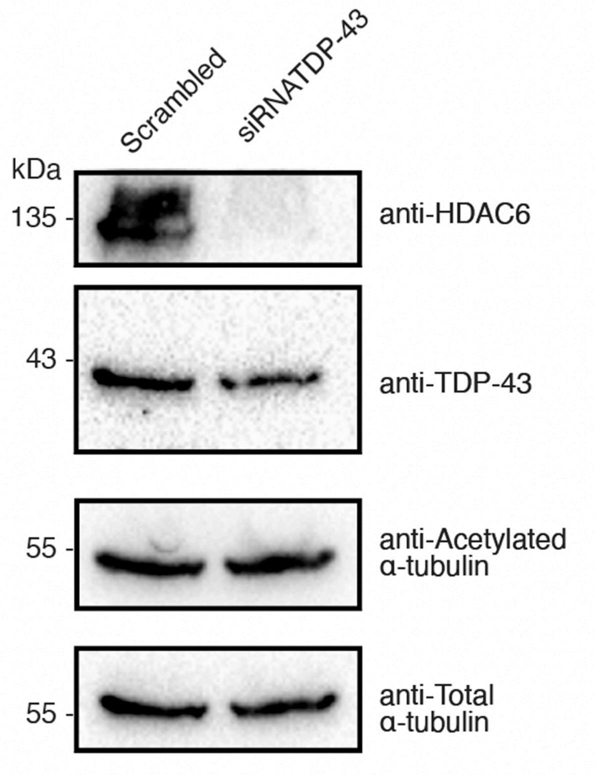

Replicate 3 as figure format

A

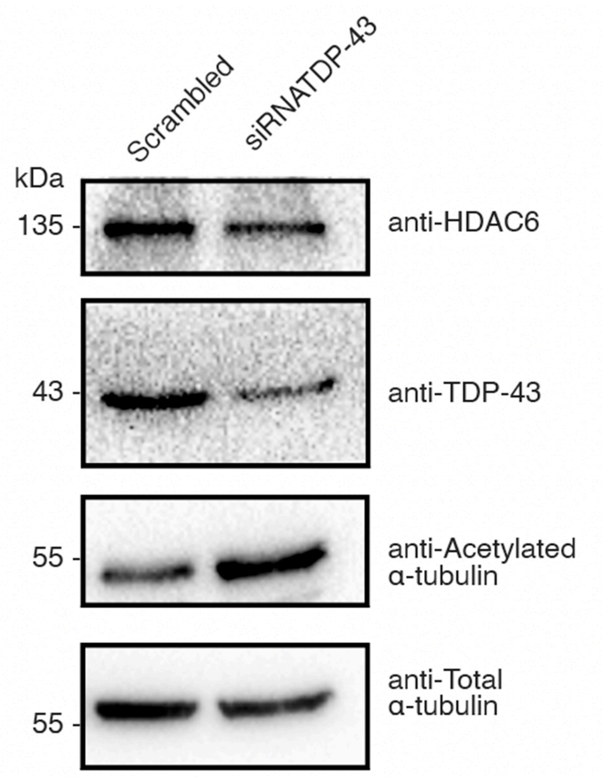

Figure S6. Replicate 1 HDAC6 complete gel Western-blot associated with Figure 6A
Cabrera-Rodríguez, R., *et al.*

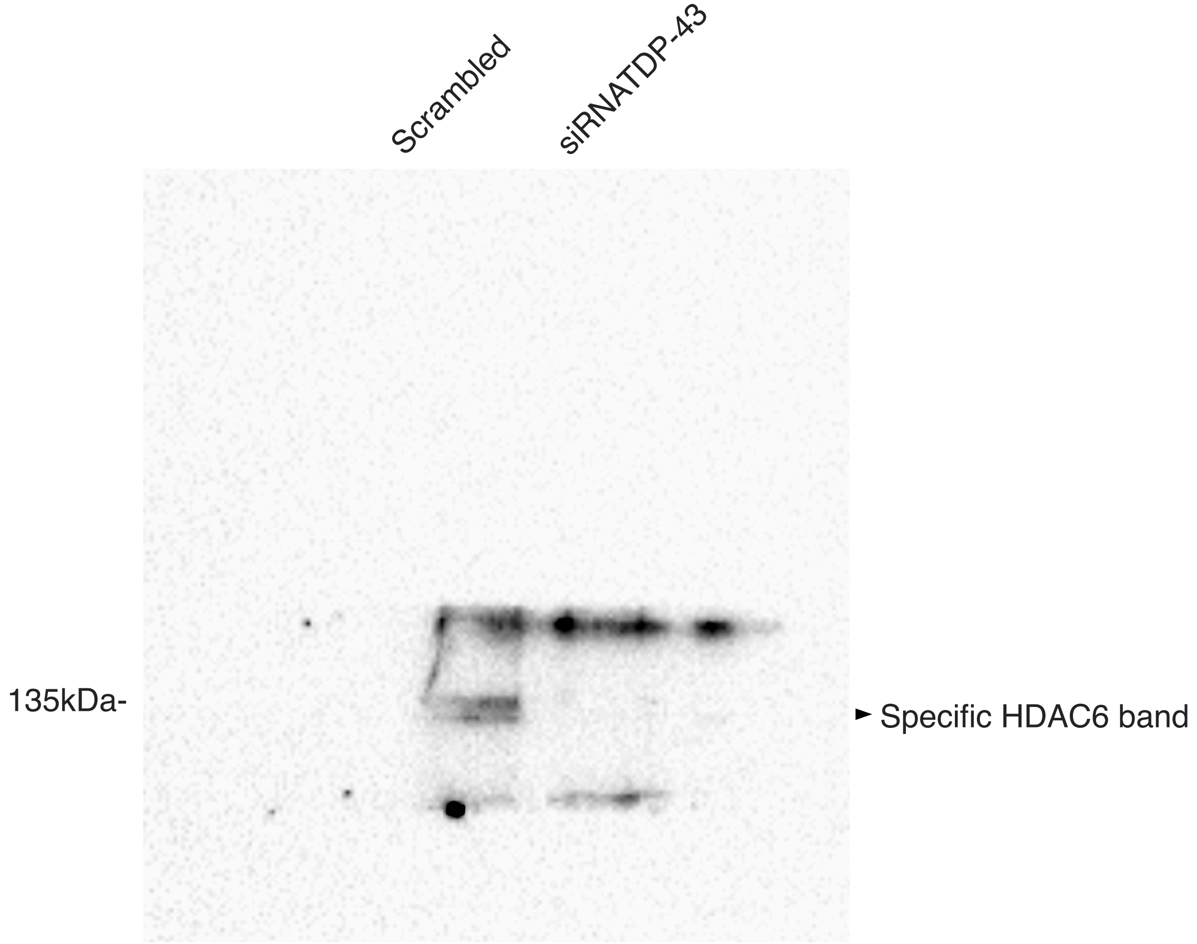

Figure S6. Replicate 1 TDP-43 complete gel Western-blot associated with Figure 6A
Cabrera-Rodríguez, R., *et al.*

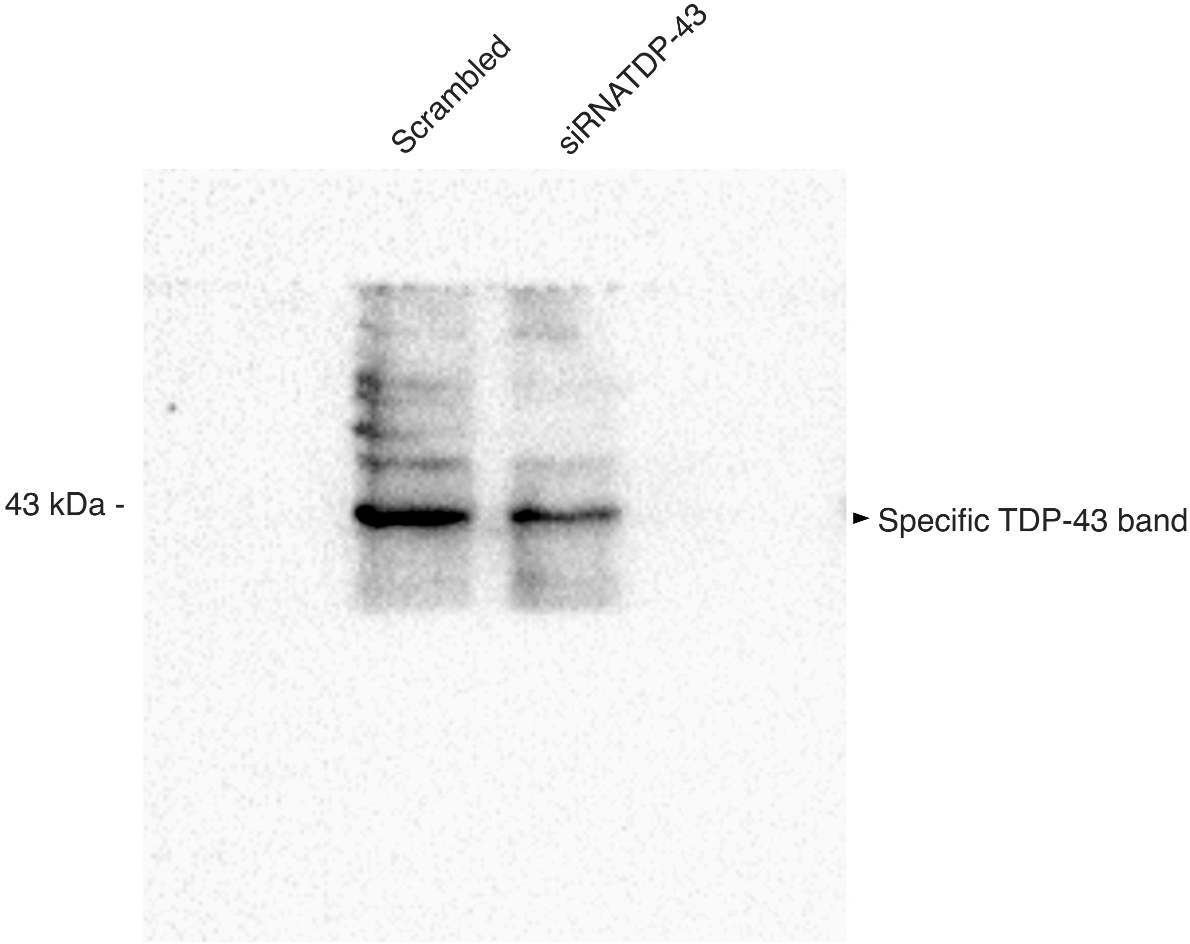

Figure S6. Replicate 1 Acetylated α-tubulin complete gel Western-blot associated with Figure 6A

Cabrera-Rodríguez, R., *et al.*

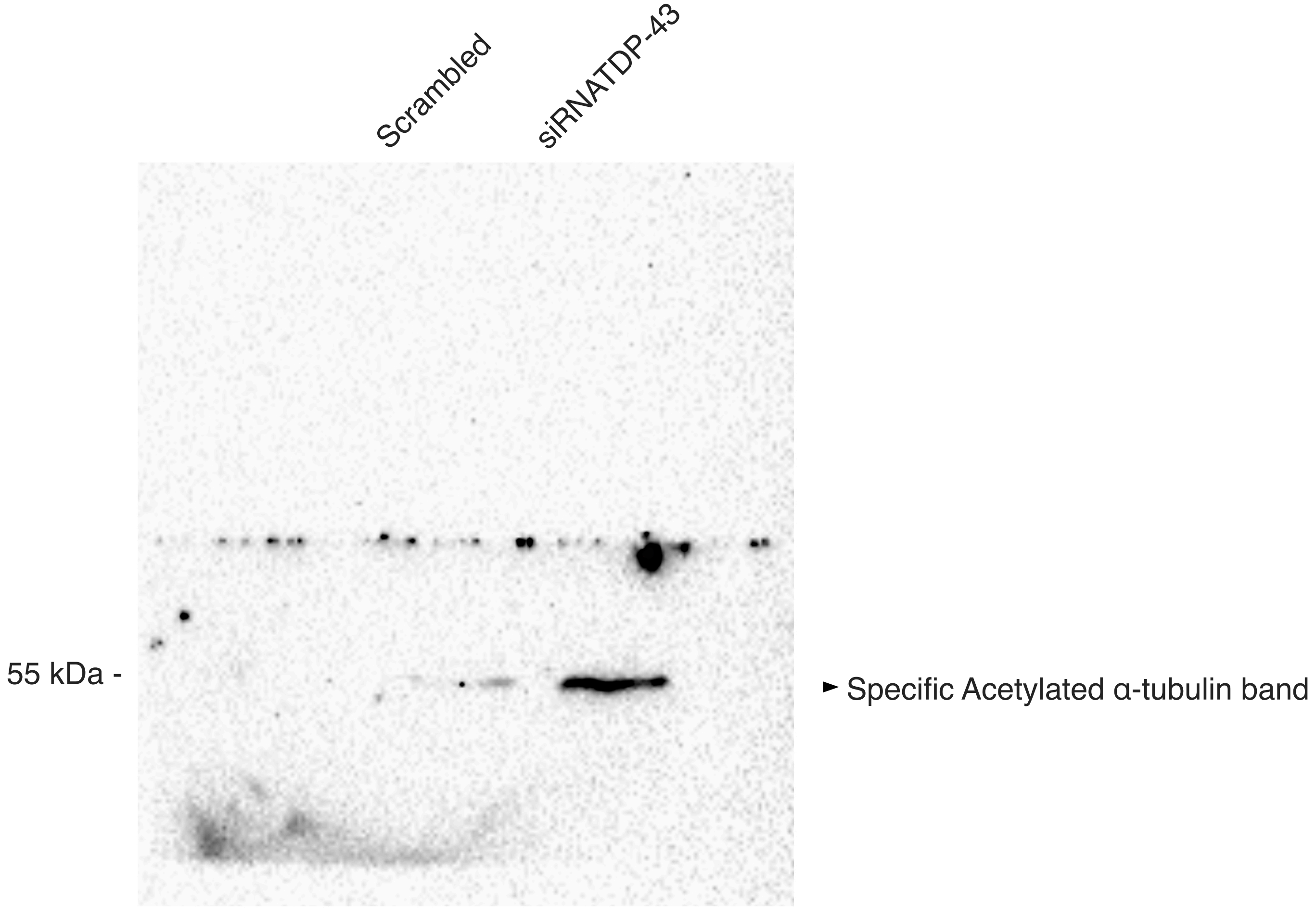

Figure S6. Replicate 1 Total α-tubulin complete gel   Western-blot associated with Figure 6A

Cabrera-Rodríguez, R., *et al.*

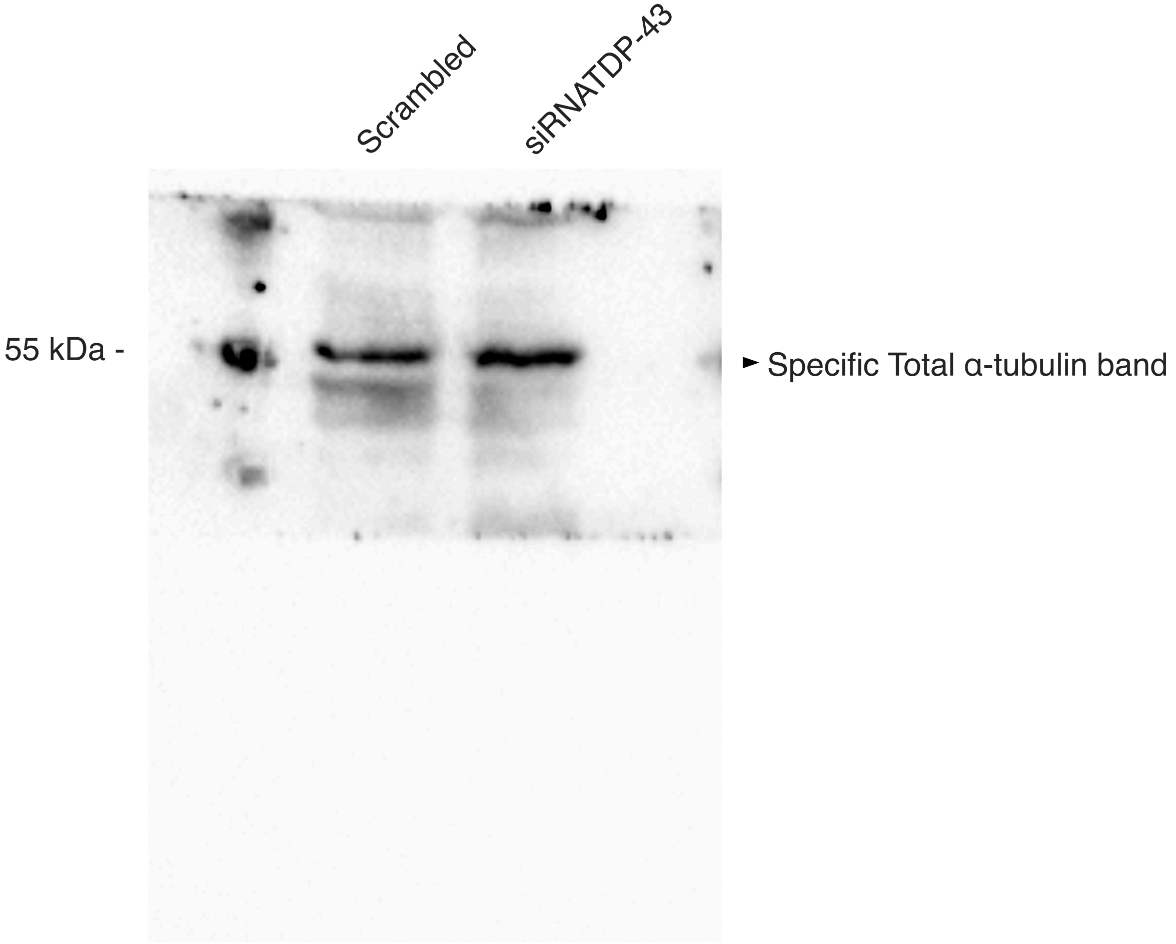

Figure S6. Replicate 2 HDAC6 complete gel   Western-blot associated with Figure 6A

Cabrera-Rodríguez, R., *et al.*

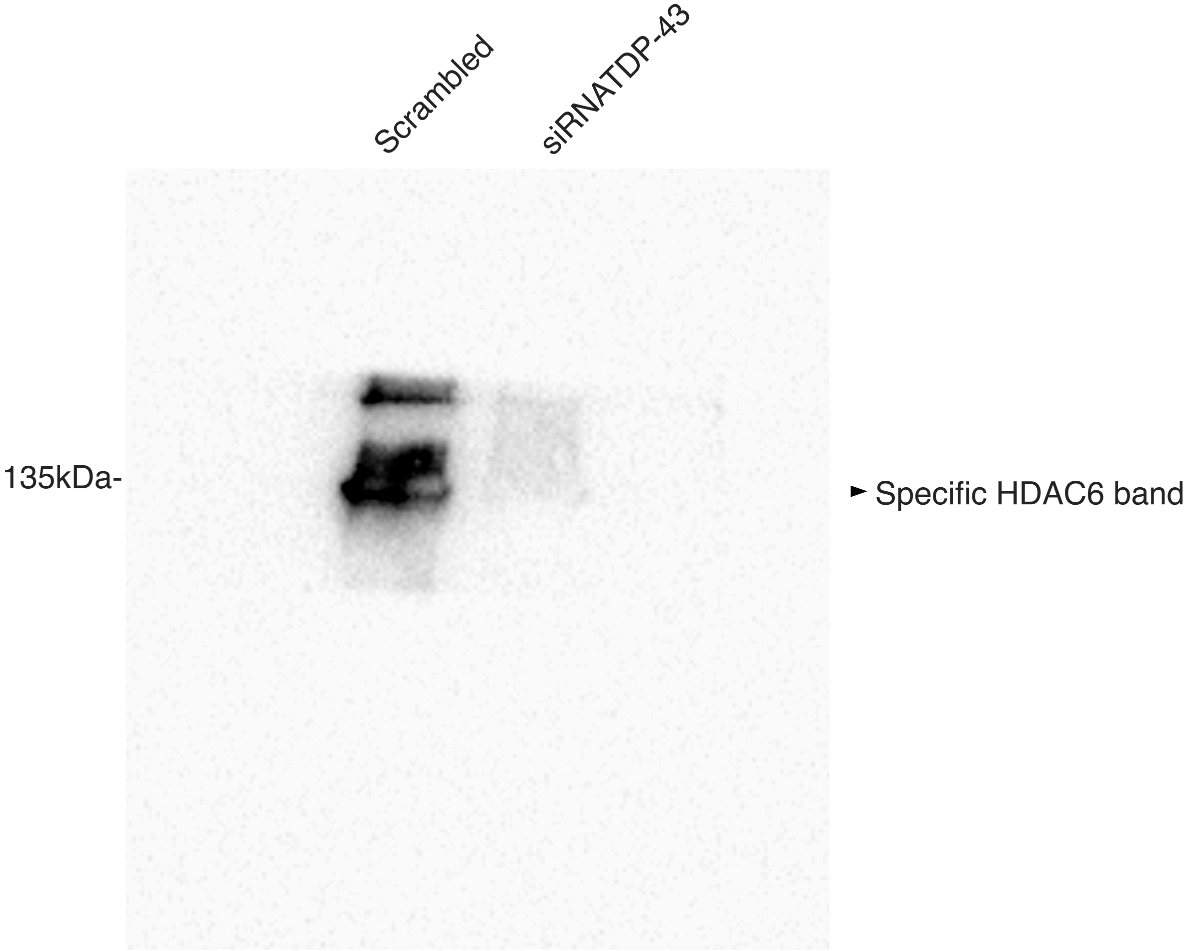

Figure S6. Replicate 2 TDP-43 complete gel Western-blot associated with Figure 6A
Cabrera-Rodríguez, R., *et al.*

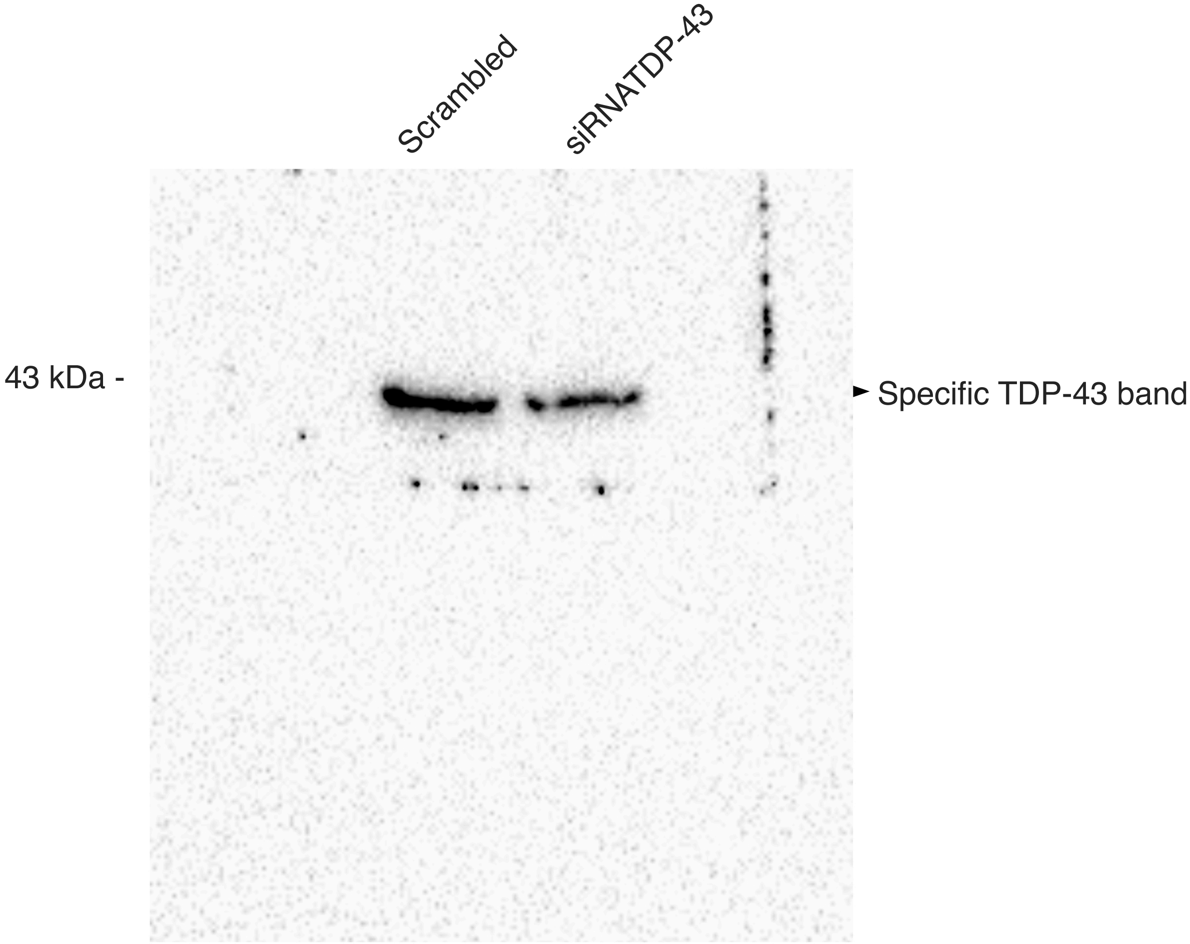

Figure S6. Replicate 2 Acetylated α-tubulin complete gel Western-blot associated with Figure 6A

Cabrera-Rodríguez, R., *et al.*

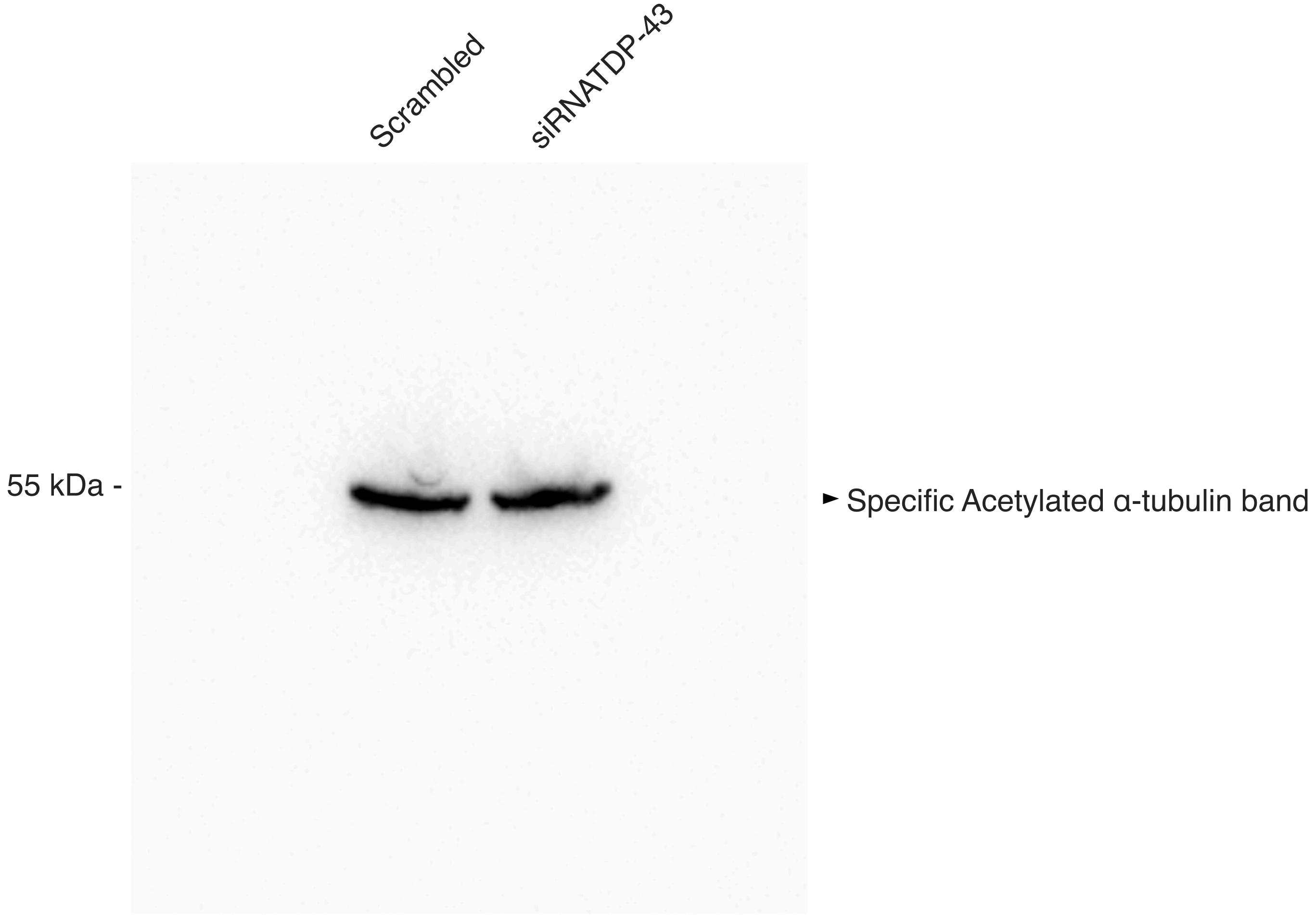

Figure S6. Replicate 2 Total α-tubulin complete gel   Western-blot associated with Figure 6A
Cabrera-Rodríguez, R., *et al.*

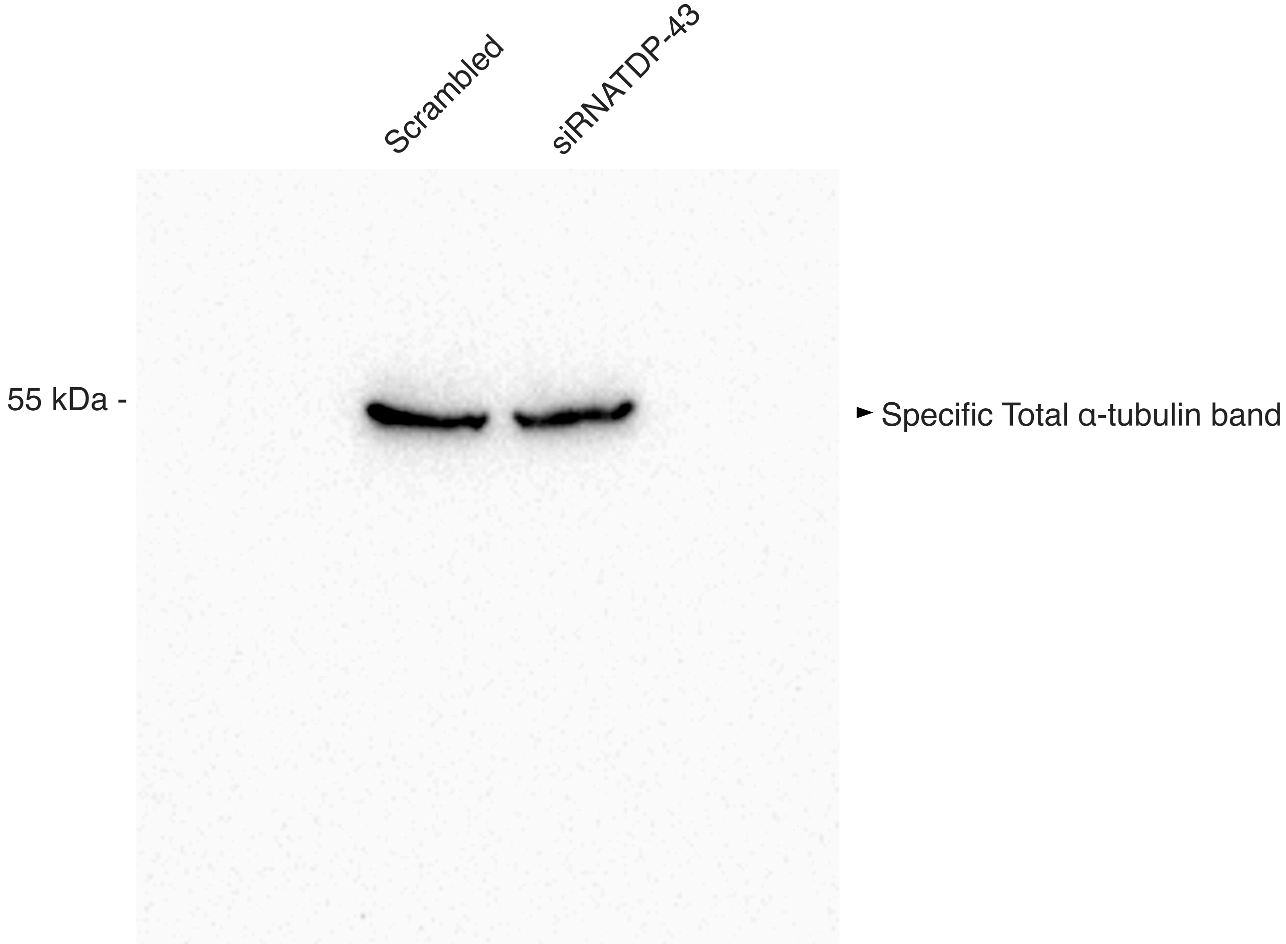

Figure S6. Replicate 3 HDAC6 complete gel Western-blot associated with Figure 6A
Cabrera-Rodríguez, R., *et al.*

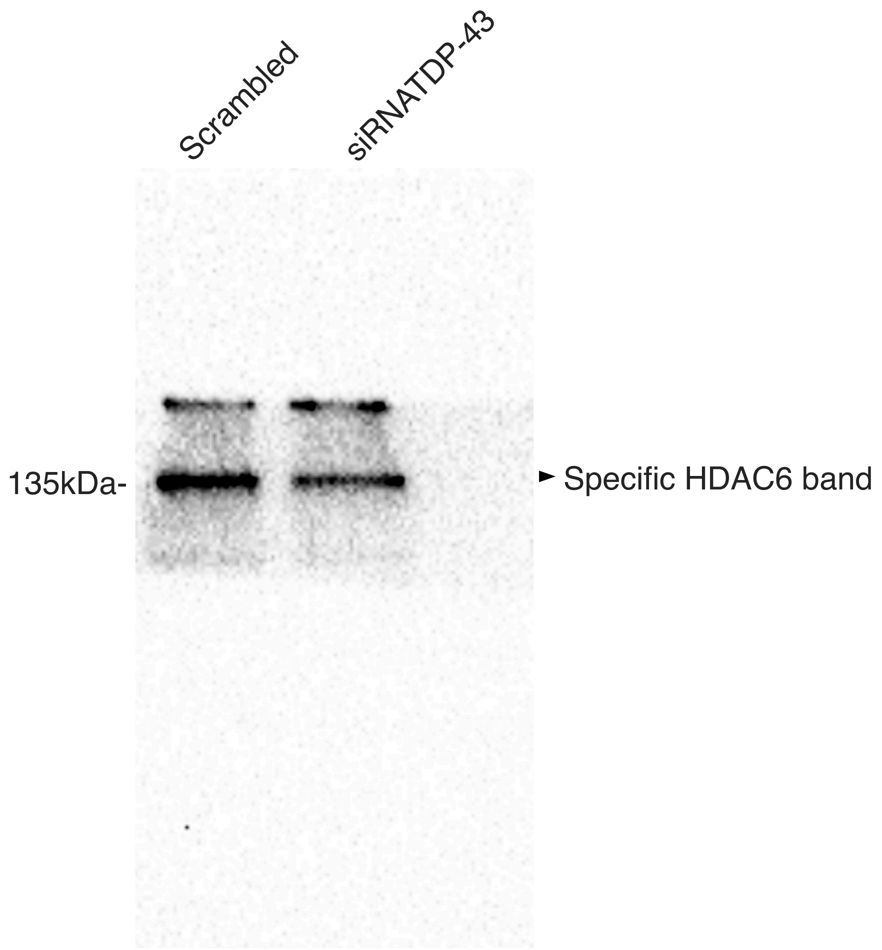

Figure S6. Replicate 3 TDP-43 complete gel Western-blot associated with Figure 6A
Cabrera-Rodríguez, R., *et al.*

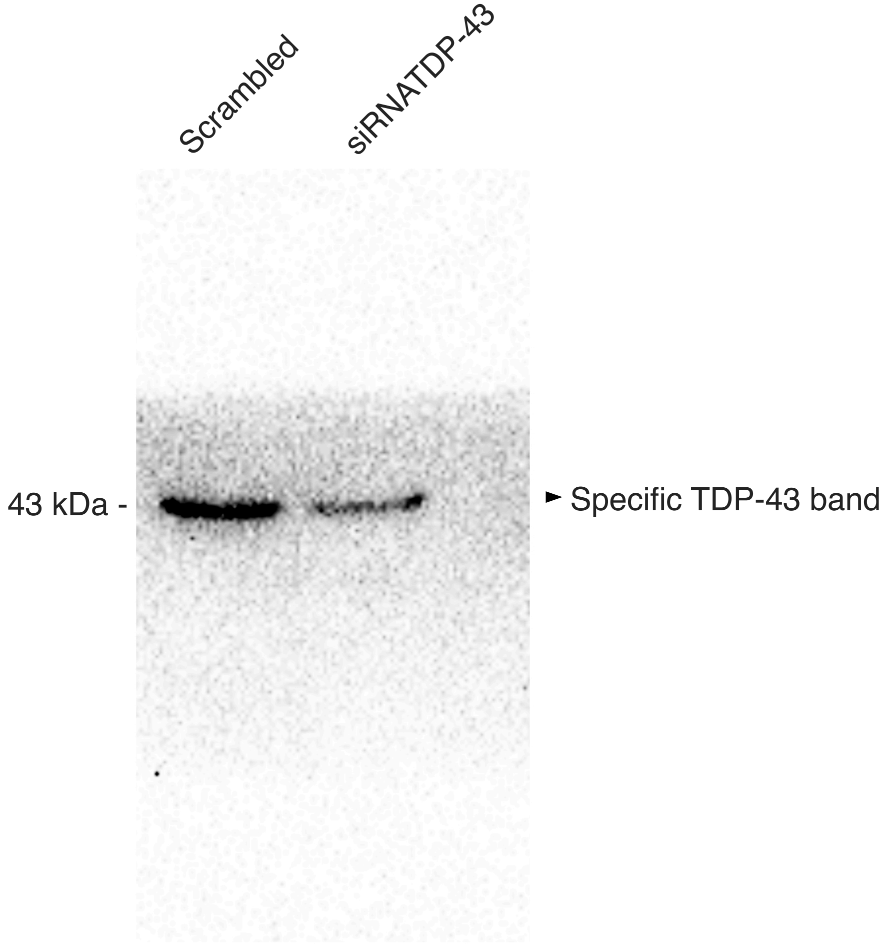

Figure S6. Replicate 3 Acetylated α-tubulin complete gel   Western-blot associated with Figure 6A

Cabrera-Rodríguez, R., *et al.*

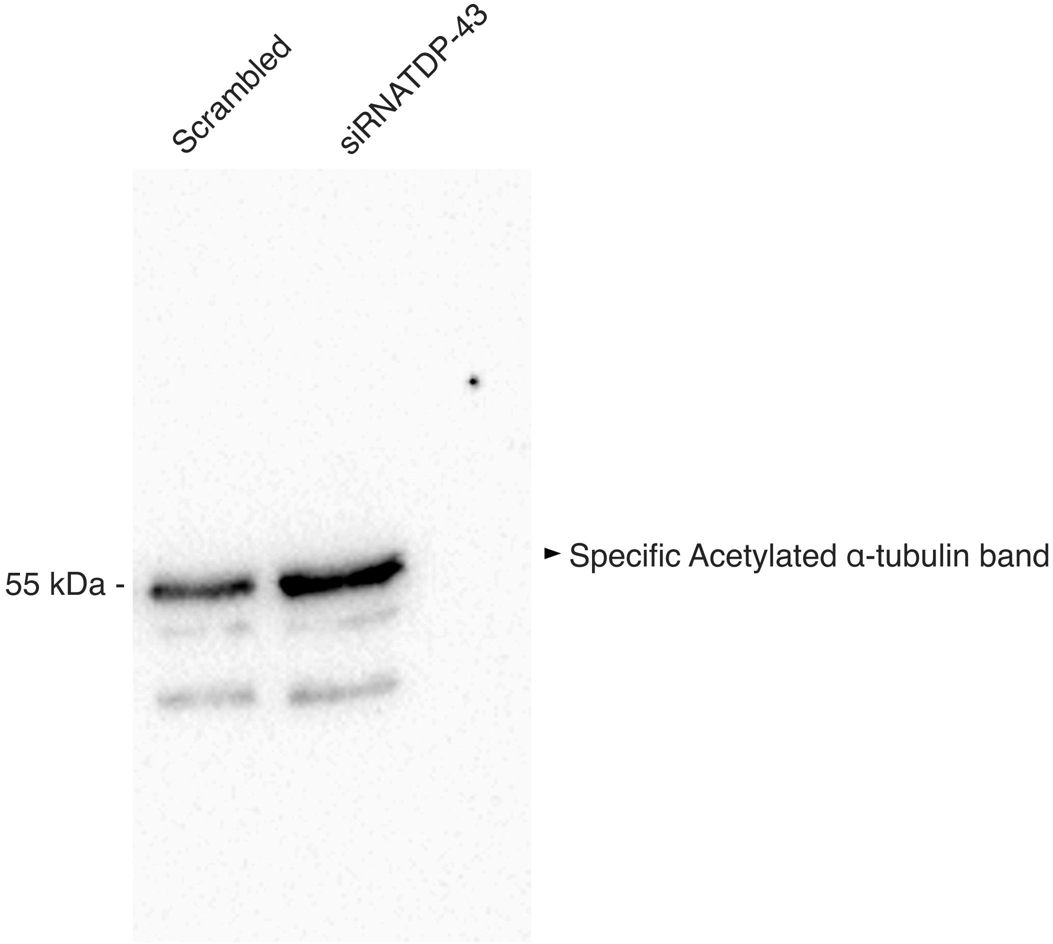

Figure S6. Replicate 3 Total α-tubulin complete gel Western-blot associated with Figure 6A

Cabrera-Rodríguez, R., *et al.*

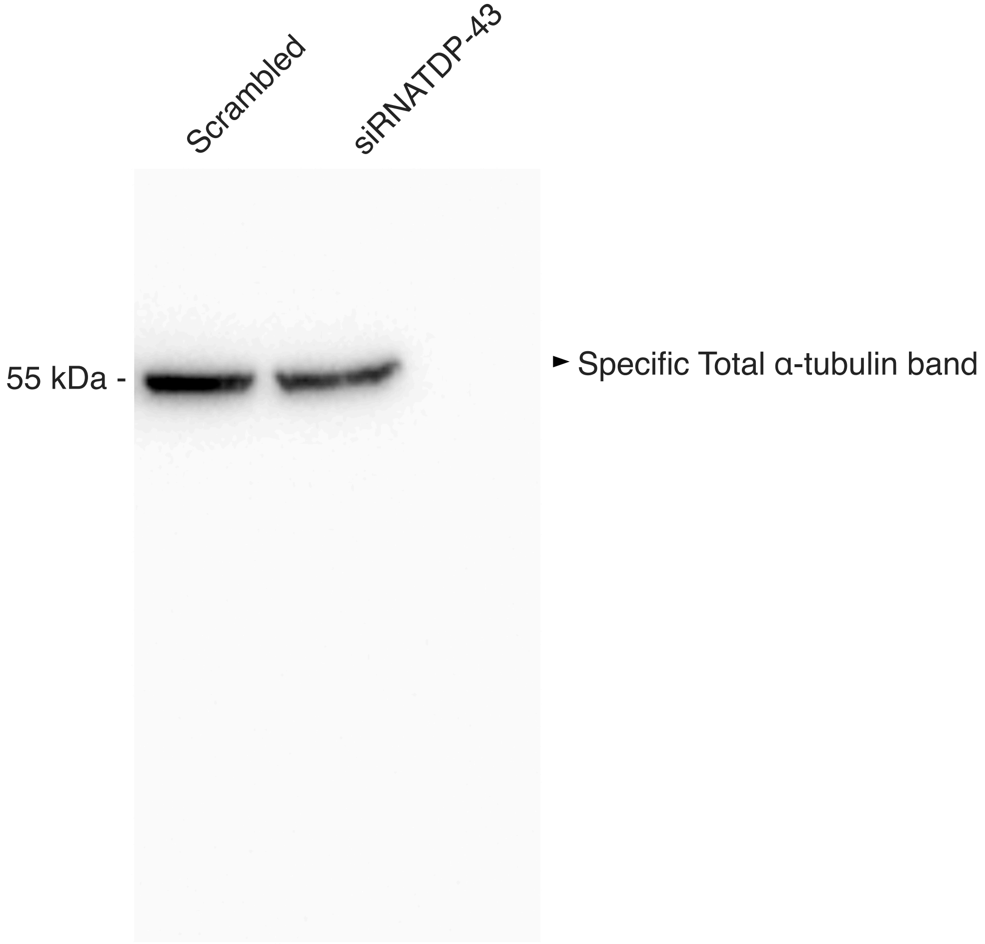

Figure S7A. Western-blot replicates associated with Figure 7A Cabrera-Rodríguez R., *et al.*

Replicate 1 as figure format

A

Lysis of cell-producing virus

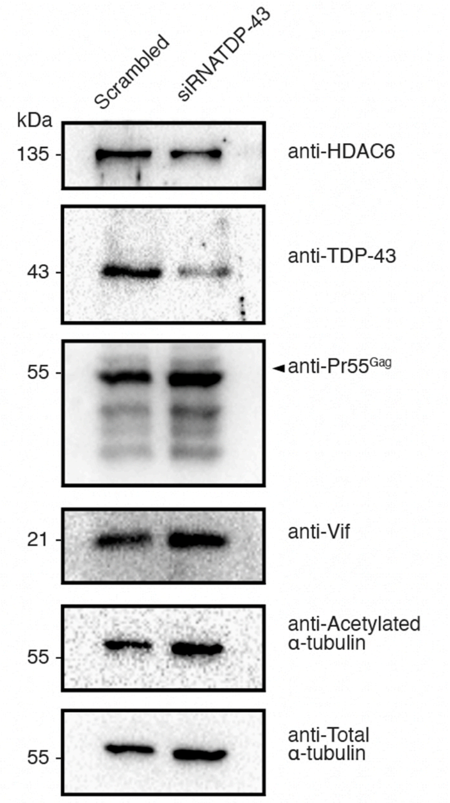

Replicate 2 as figure format

A

Lysis of cell-producing virus

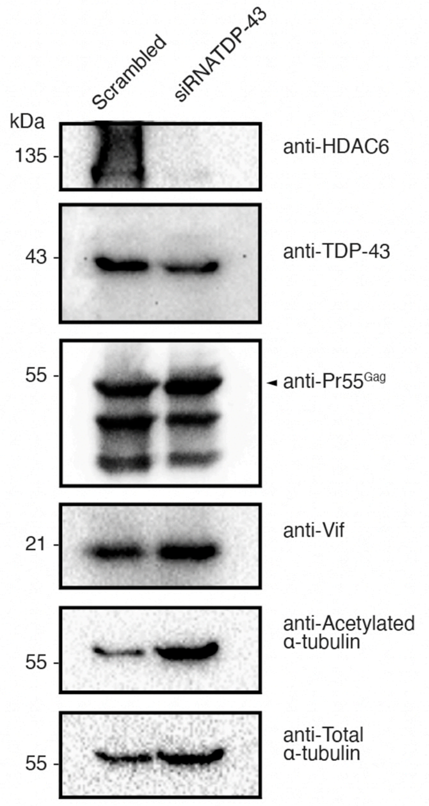

Replicate 3 as figure format

A

Lysis of cell-producing virus

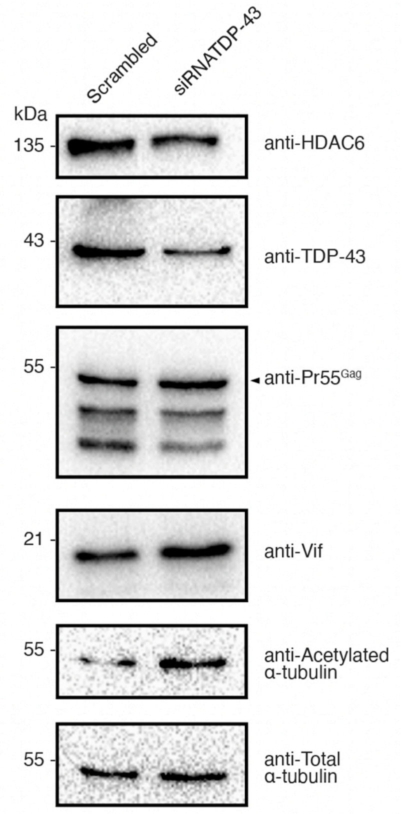

Figure S7A. Replicate 1 HDAC6 complete gel Western-blot associated with Figure 7A
Cabrera-Rodríguez, R., *et al.*

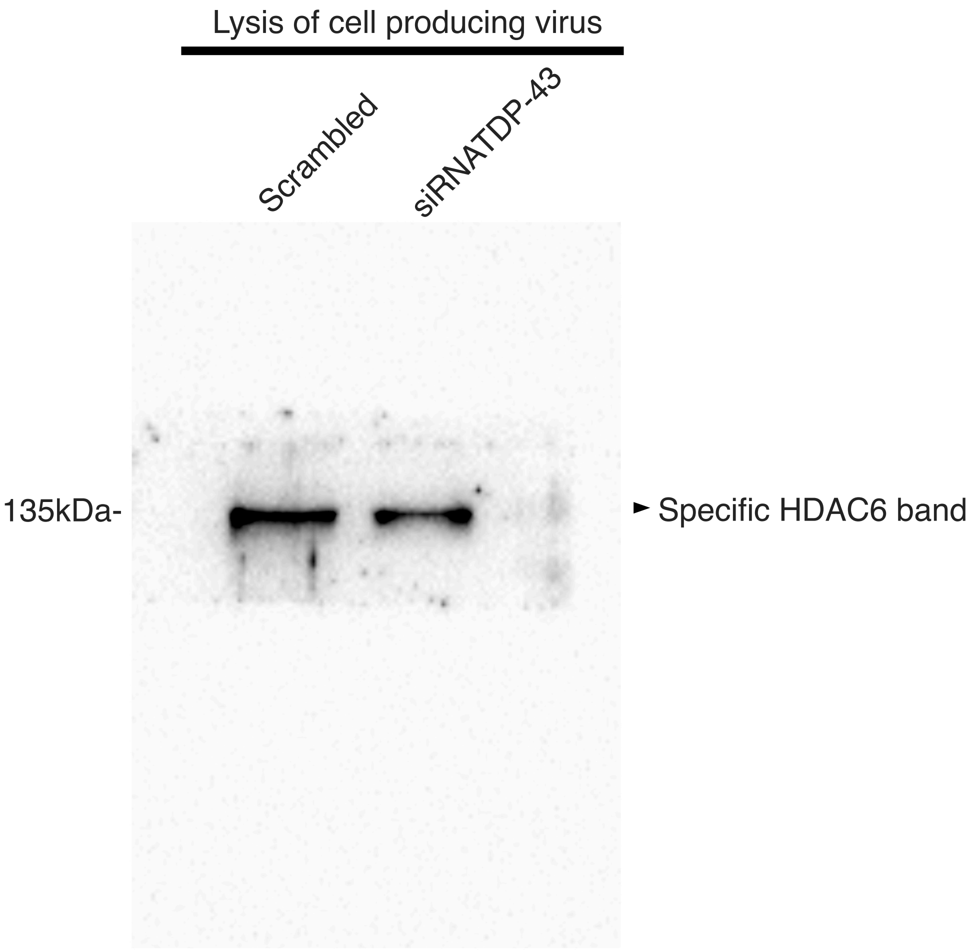

Figure S7A. Replicate 1 TDP-43 complete gel   Western-blot associated with Figure 7A
Cabrera-Rodríguez, R., *et al.*

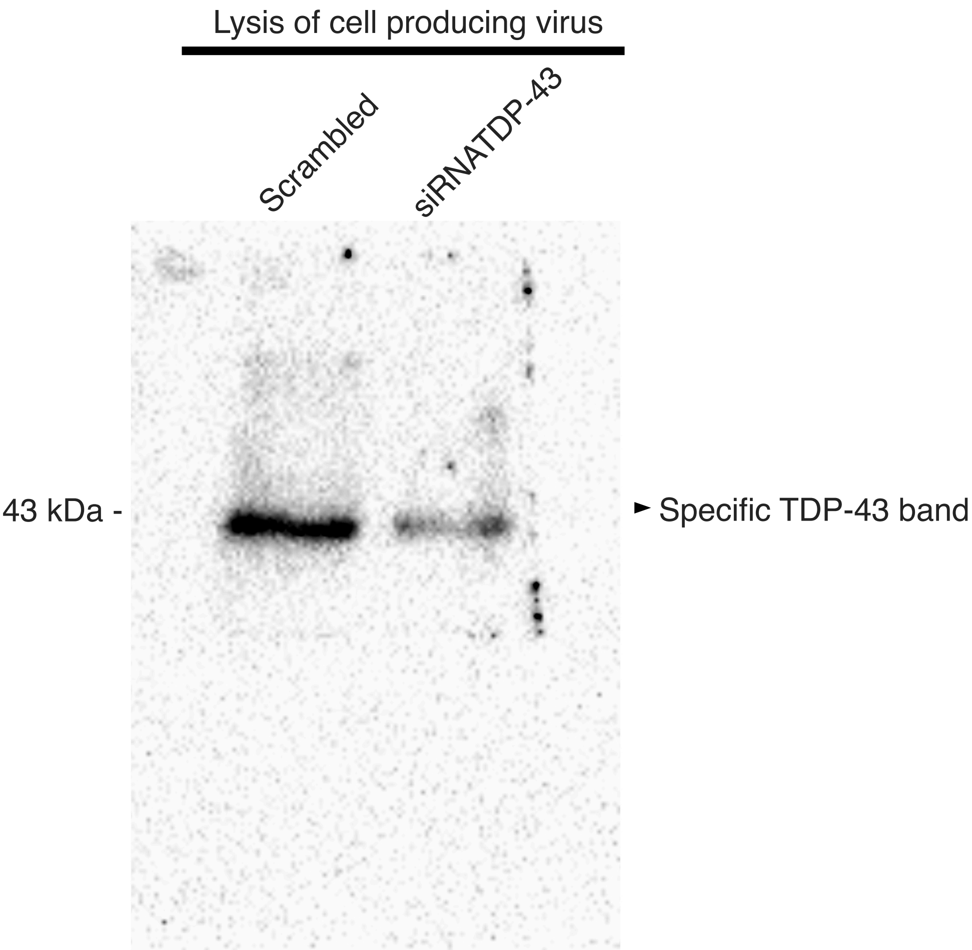

Figure S7A. Replicate 1 $Pr55^{Gag}$ complete gel Western-blot associated with Figure 7A

Cabrera-Rodríguez, R., *et al.*

Lysis of cell producing virus

Scrambled

siRNATDP-43

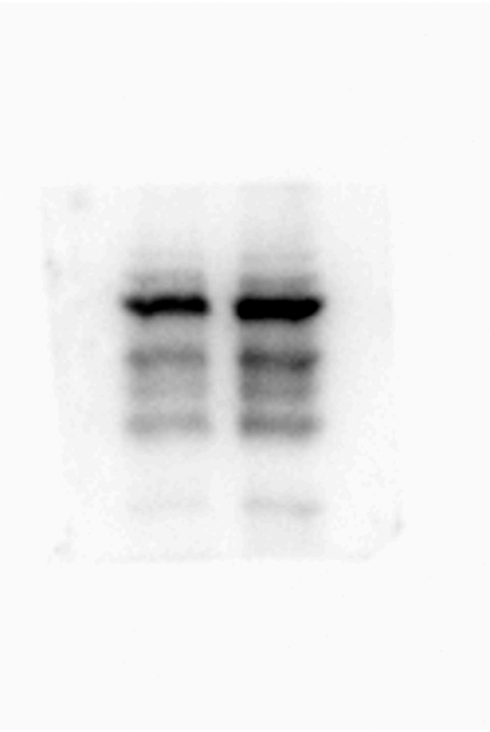

55 kDa -

► Specific $Pr55^{Gag}$ band

Figure S7A. Replicate 1 Vif complete gel Western-blot associated with Figure 7A
Cabrera-Rodríguez, R., *et al.*

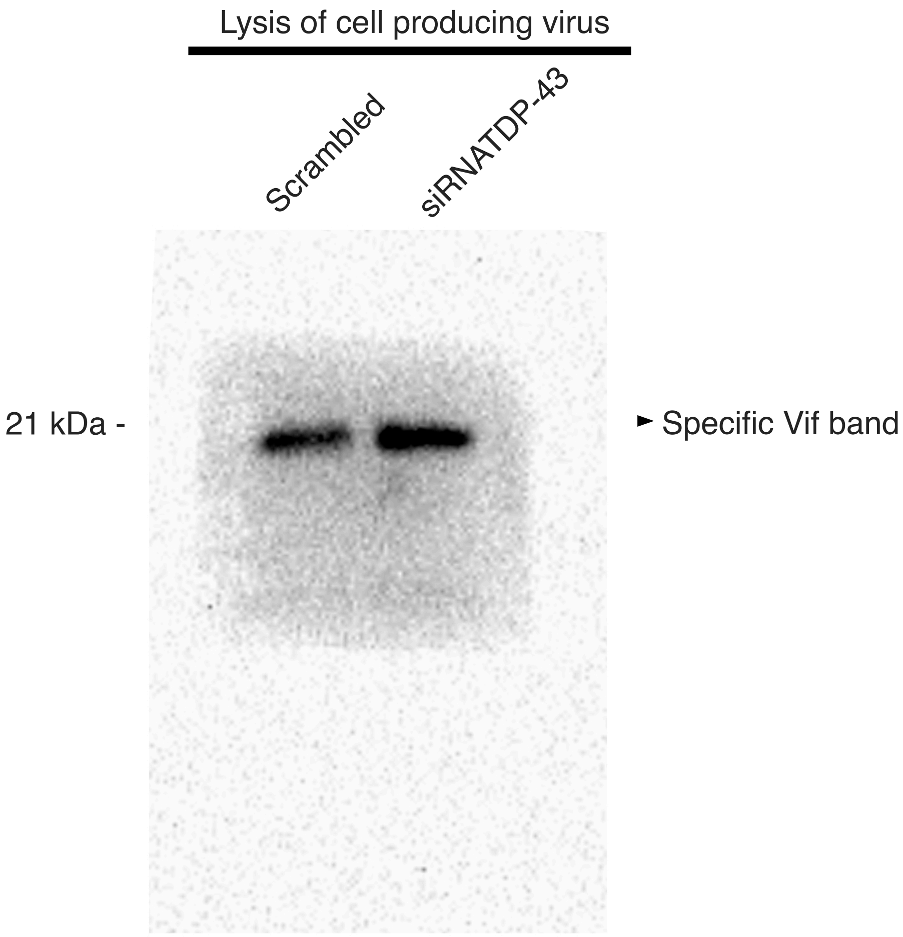

Figure S7A. Replicate 1 Acetylated α-tubulin complete gel   Western-blot associated with Figure 7A

Cabrera-Rodríguez, R., *et al.*

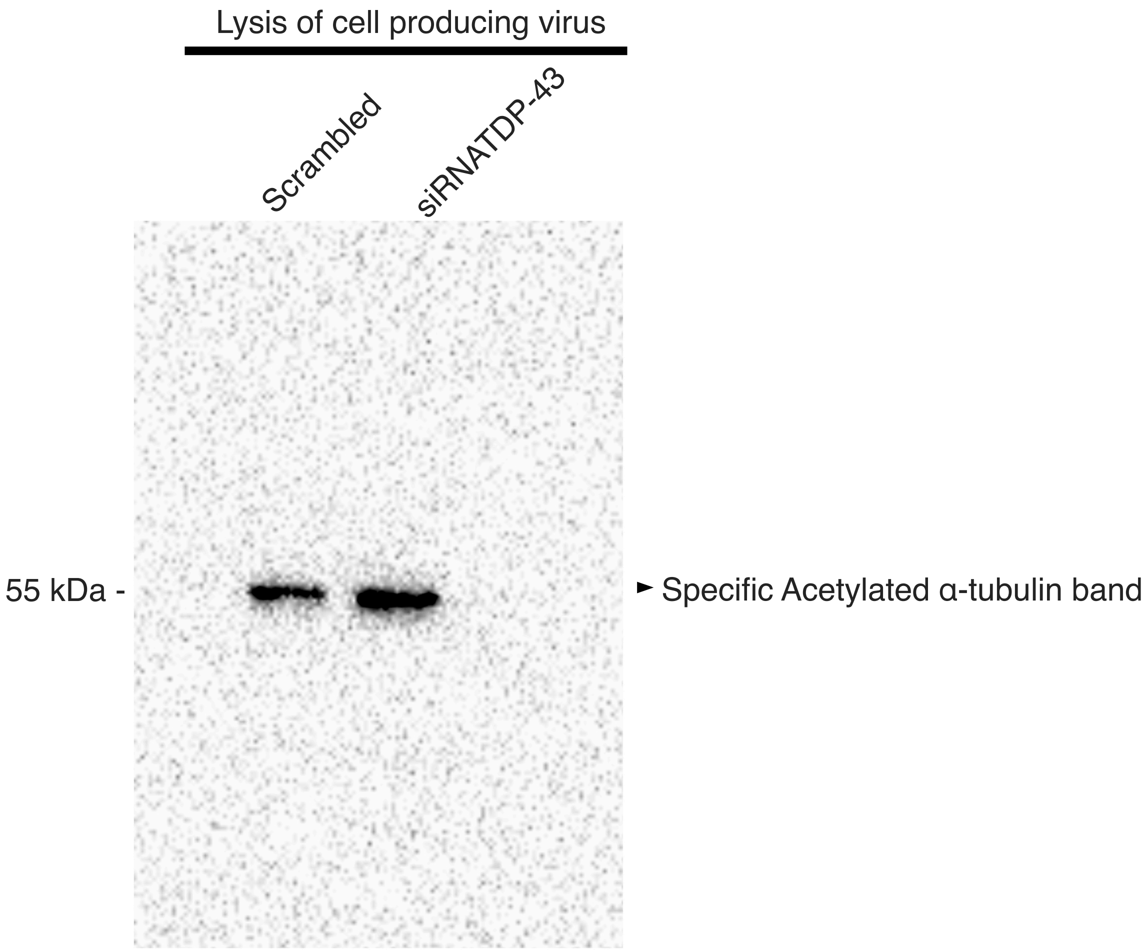

Figure S7A. Replicate 1 Total α-tubulin complete gel

Western-blot associated with Figure 7A
Cabrera-Rodríguez, R., *et al.*

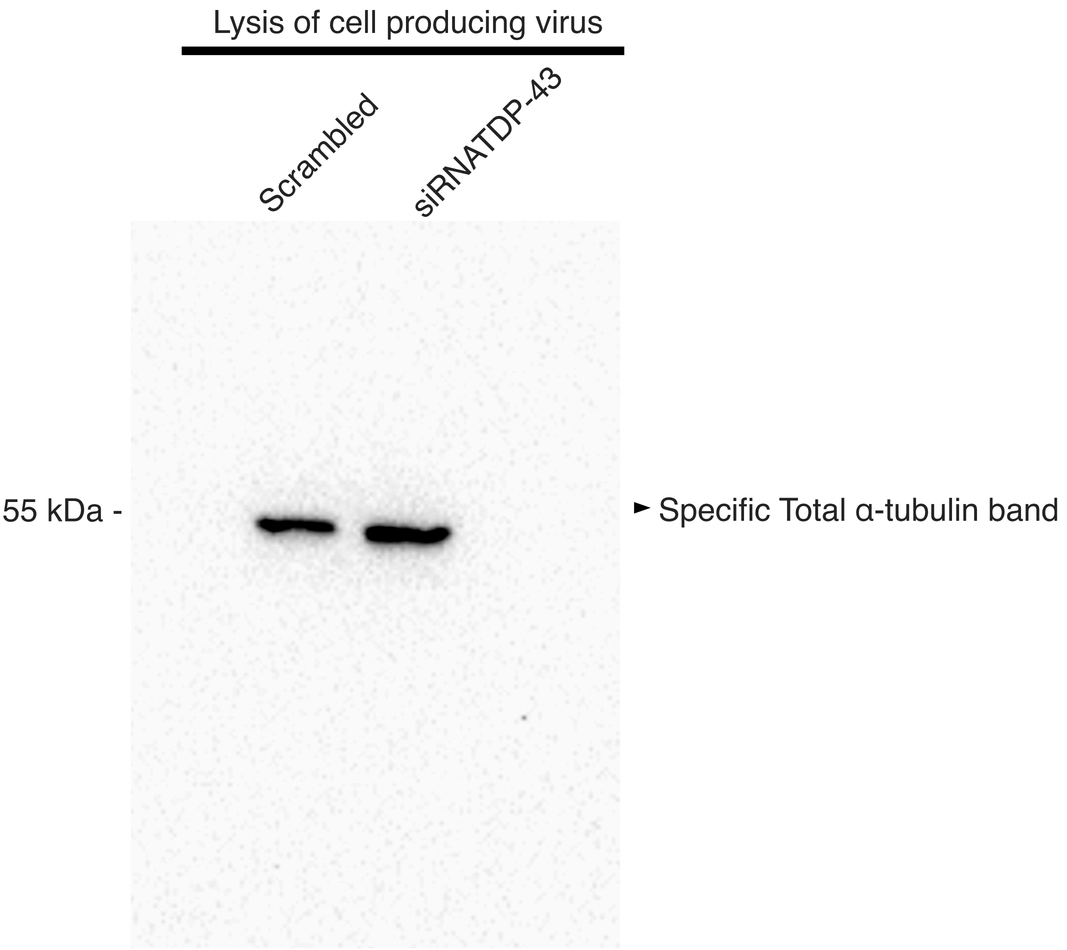

Figure S7A. Replicate 2 HDAC6 complete gel

Western-blot associated with Figure 7A
Cabrera-Rodríguez, R., *et al.*

Lysis of cell producing virus

Scrambled

siRNATDP-43

135kDa-

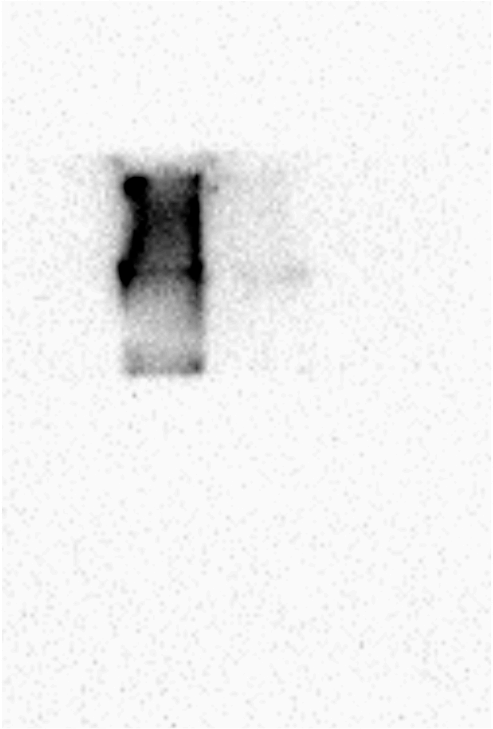

► Specific HDAC6 band

Figure S7A. Replicate 2 TDP-43 complete gel   Western-blot associated with Figure 7A
Cabrera-Rodríguez, R., *et al.*

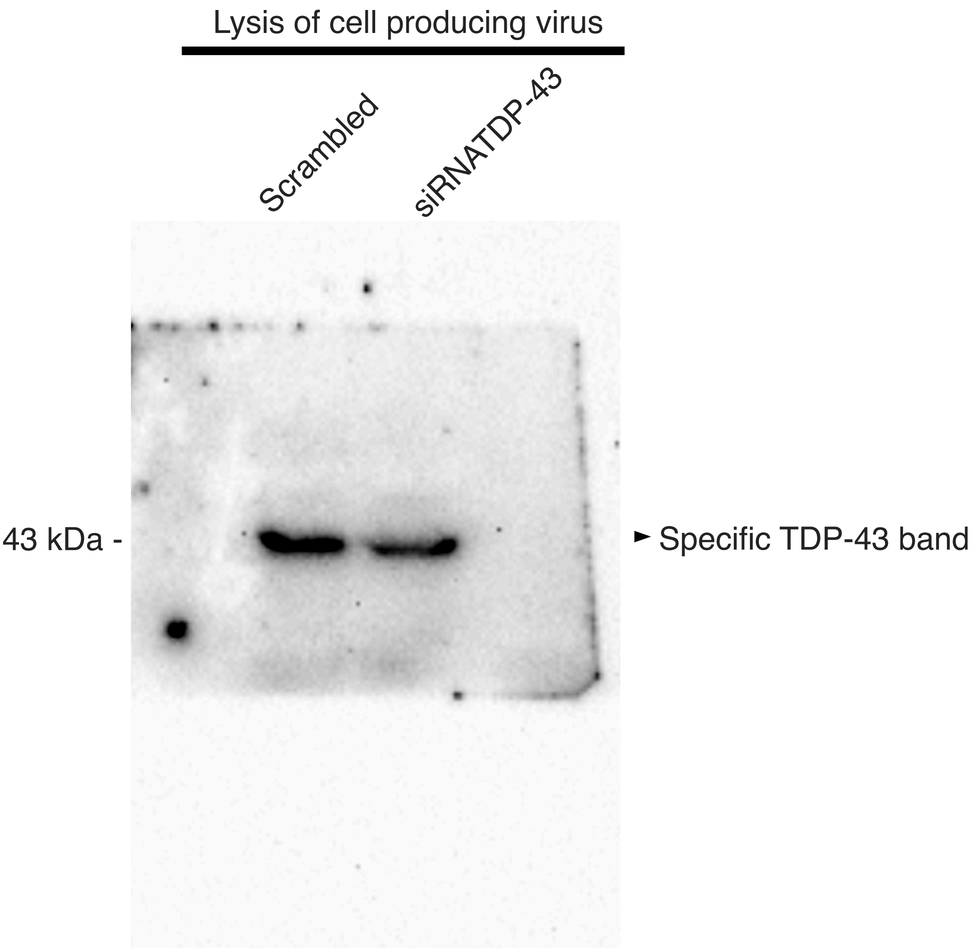

Figure S7A. Replicate 2 $Pr55^{Gag}$ complete gel Western-blot associated with Figure 7A

Cabrera-Rodríguez, R., *et al.*

Lysis of cell producing virus

Scrambled

siRNATDP-43

55 kDa -

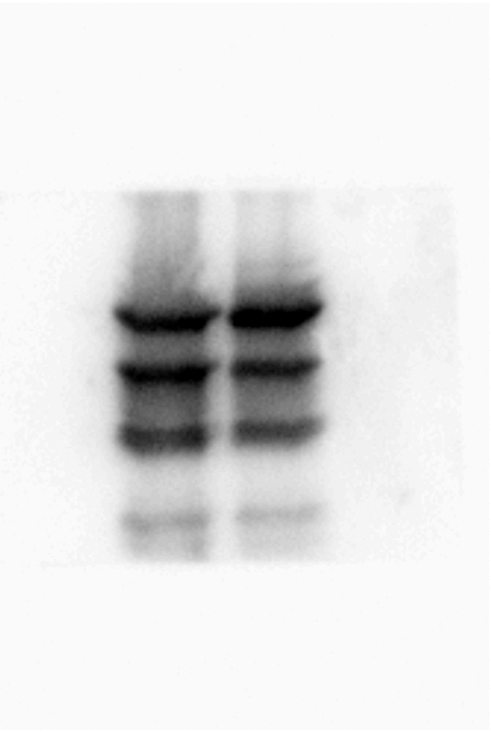

► Specific $Pr55^{Gag}$ band

Figure S7A. Replicate 2 Vif complete gel   Western-blot associated with Figure 7A

Cabrera-Rodríguez, R., *et al.*

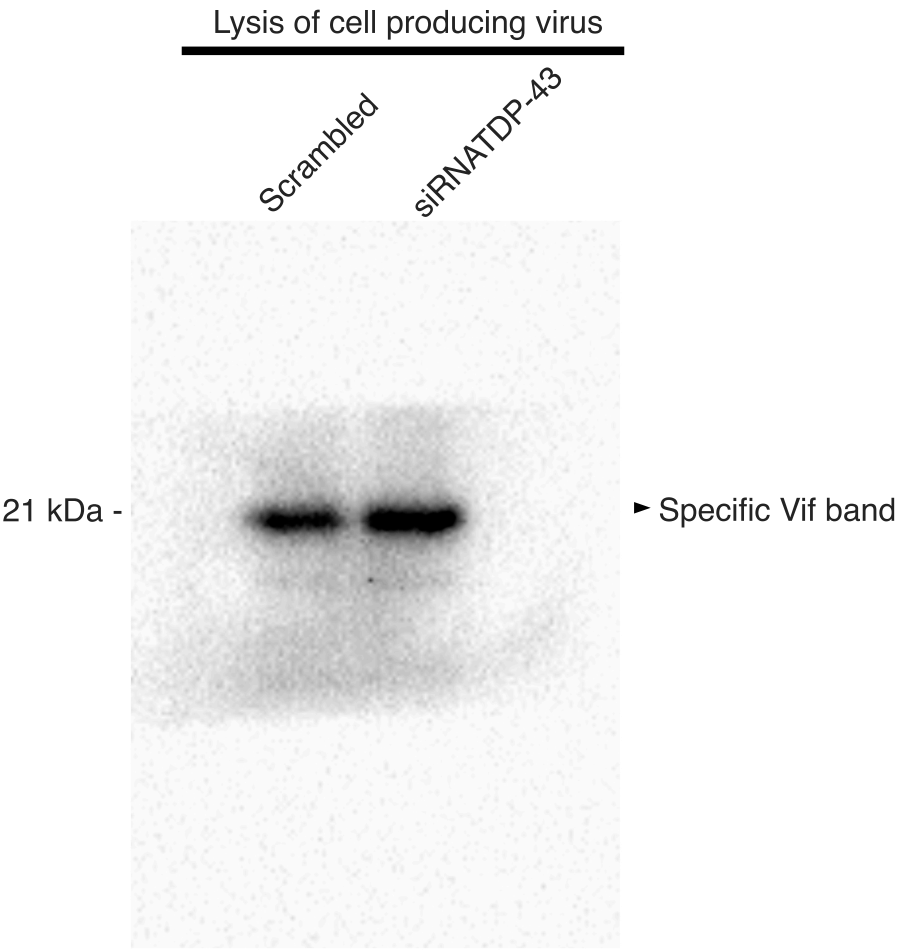

Figure S7A. Replicate 2 Acetylated α-tubulin complete gel

Western-blot associated with Figure 7A
Cabrera-Rodríguez, R., *et al.*

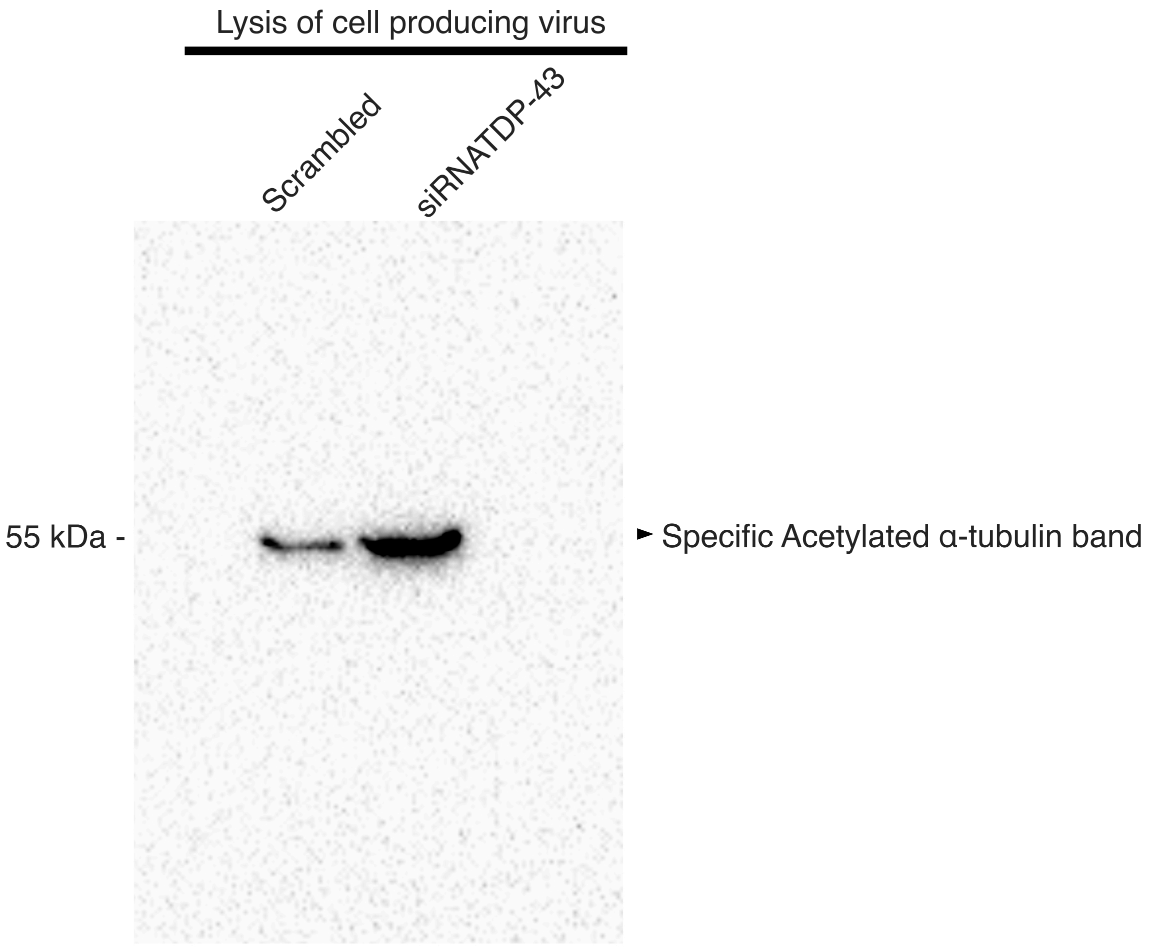

Figure S7A. Replicate 2 Total α-tubulin complete gel

Western-blot associated with Figure 7A
Cabrera-Rodríguez, R., *et al.*

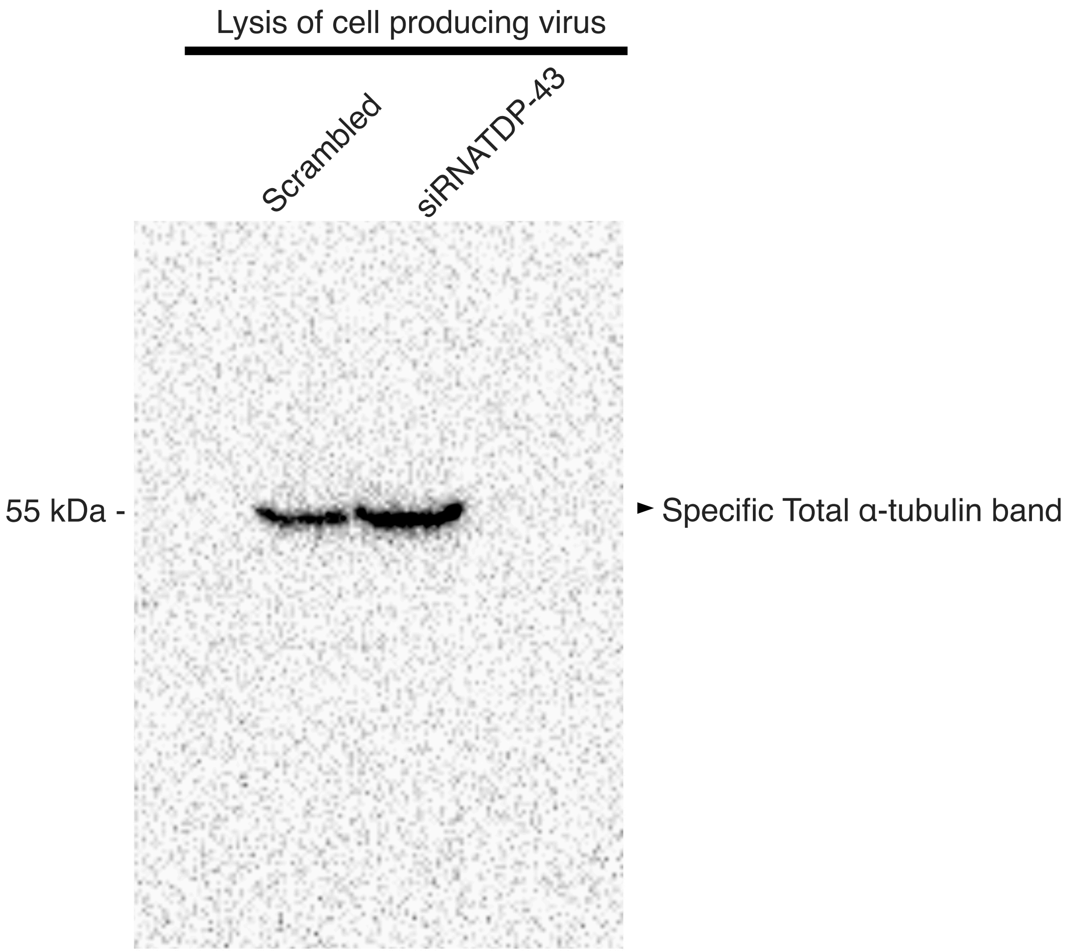

Figure S7A. Replicate 3 HDAC6 complete gel Western-blot associated with Figure 7A

Cabrera-Rodríguez, R., *et al.*

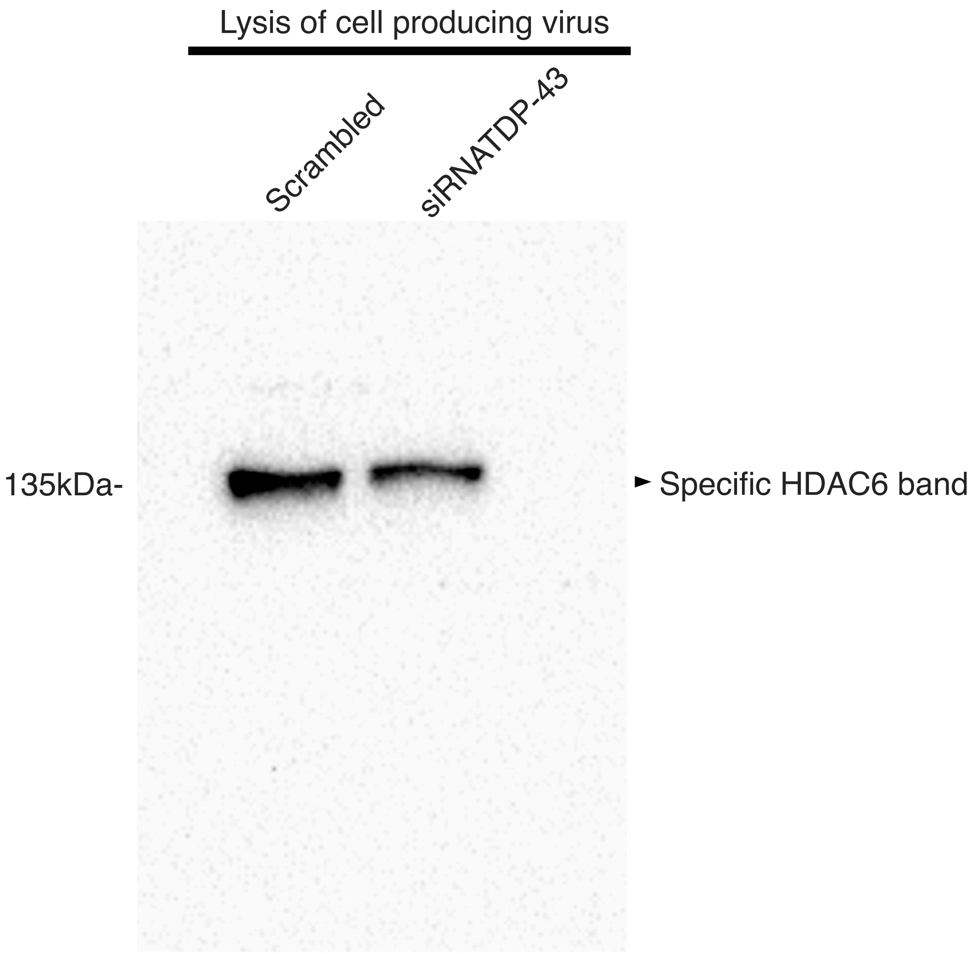

Figure S7A. Replicate 3 TDP-43 complete gel

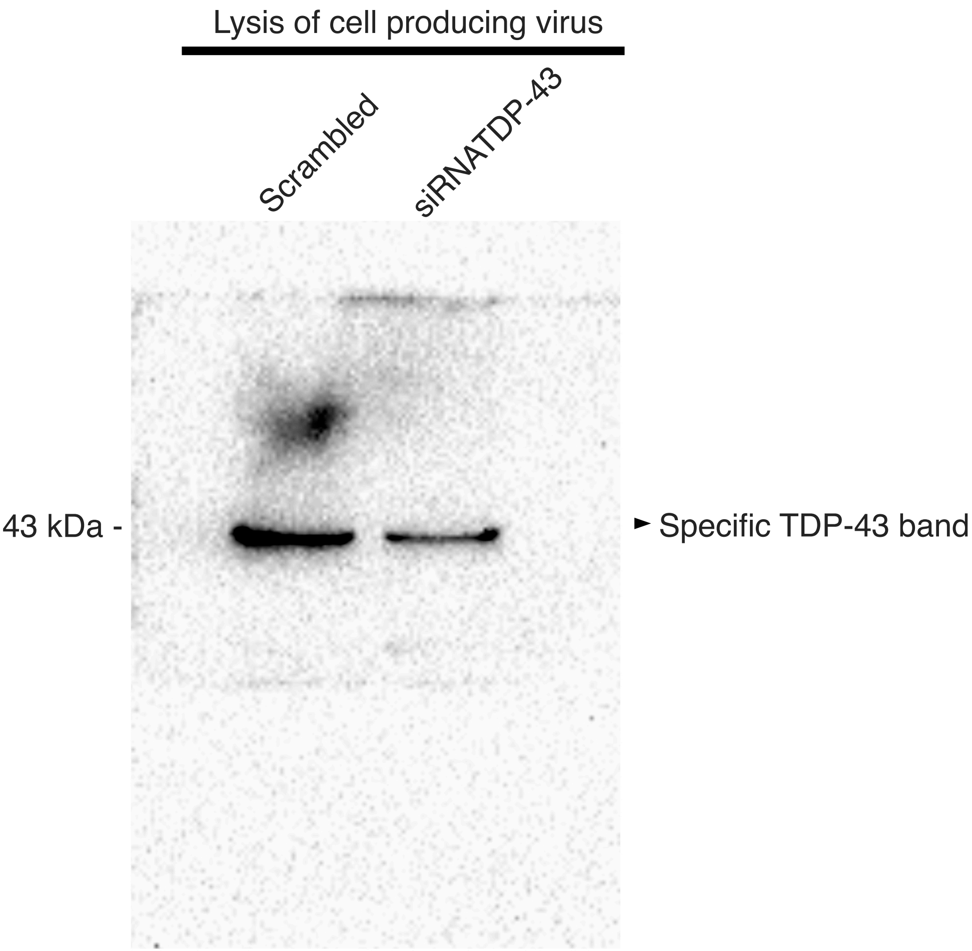

Figure S7A. Replicate 3 $Pr55^{Gag}$ complete gel   Western-blot associated with Figure 7A
Cabrera-Rodríguez, R., *et al.*

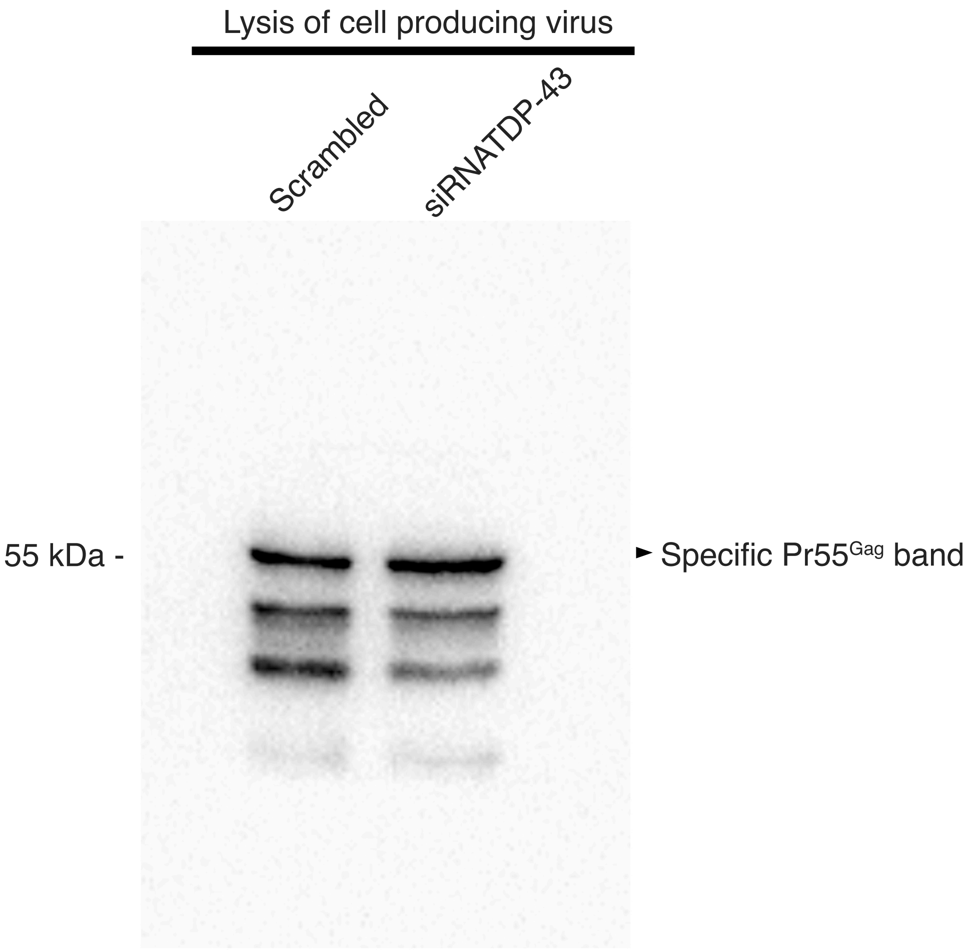

Figure S7A. Replicate 3 Vif complete gel   Western-blot associated with Figure 7A
Cabrera-Rodríguez, R., *et al.*

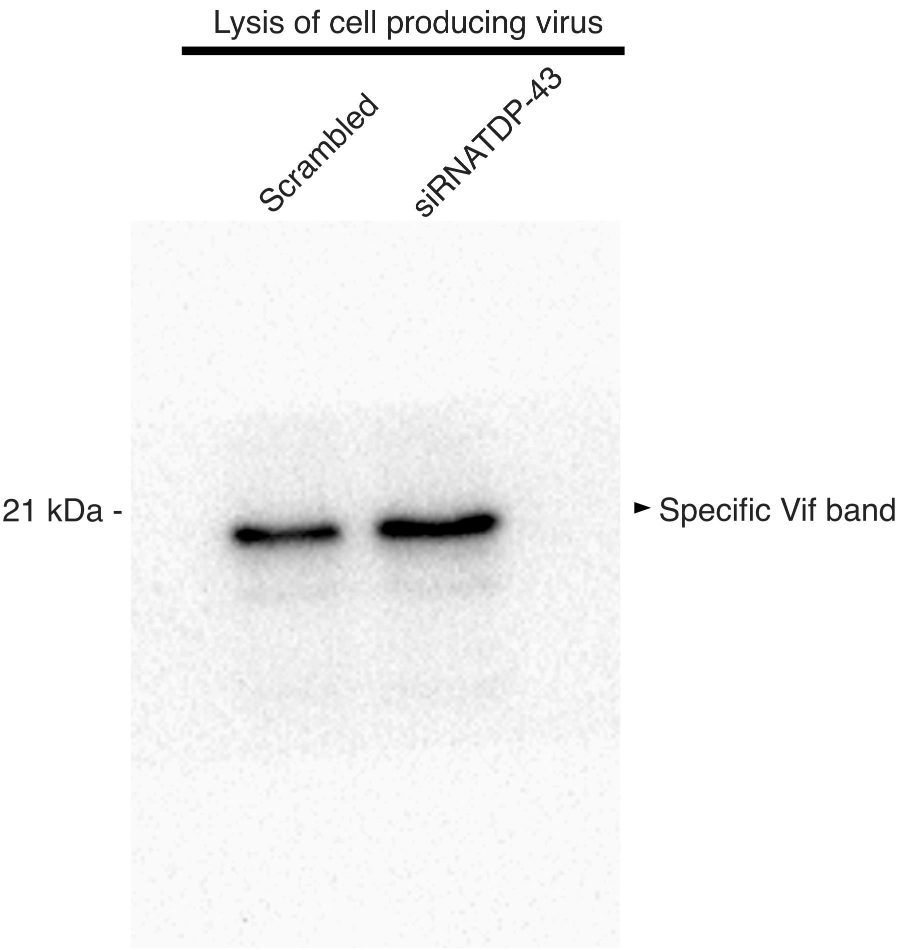

Figure S7A. Replicate 3 Acetylated α-tubulin complete gel Western-blot associated with Figure 7A

Cabrera-Rodríguez, R., *et al.*

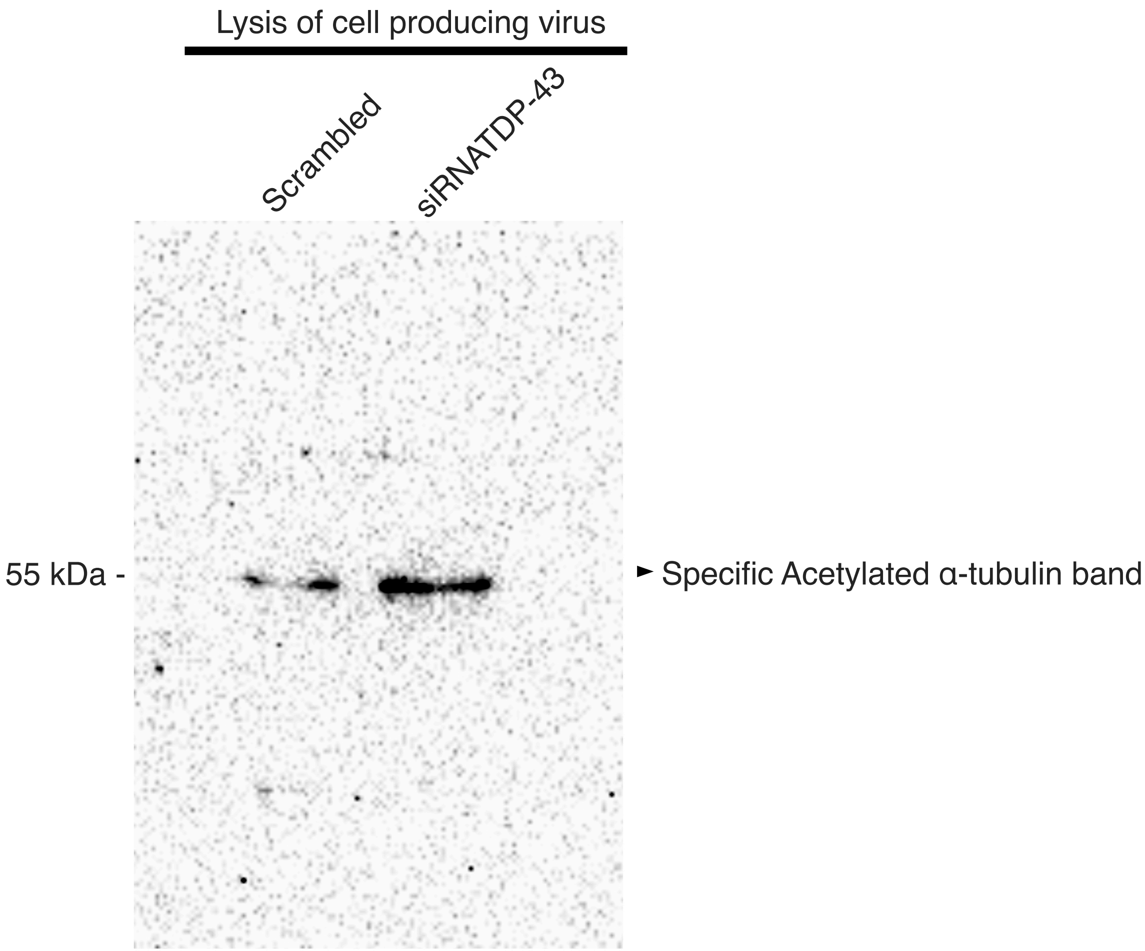

Figure S7A. Replicate 3 Total α-tubulin complete gel Western-blot associated with Figure 7A

Cabrera-Rodríguez, R., *et al.*

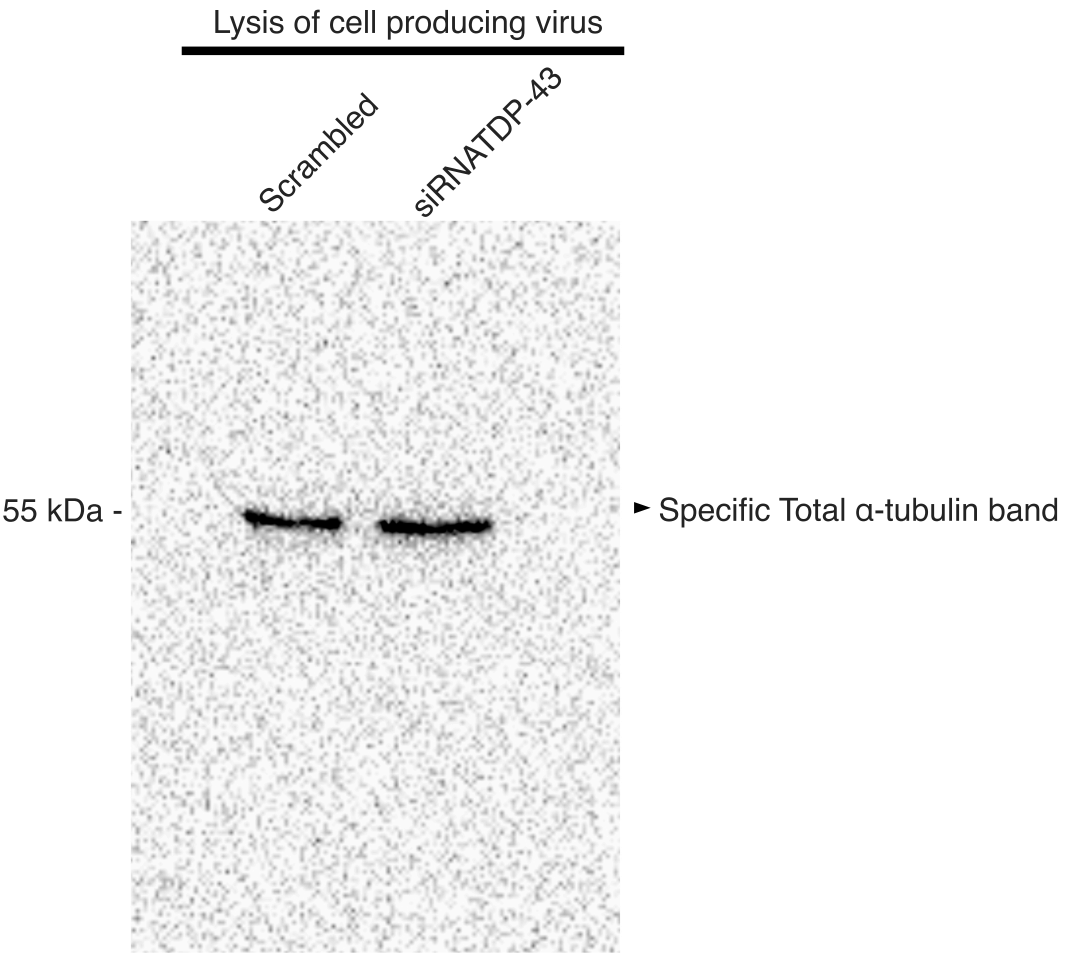

Replicate 1 as figure format

C

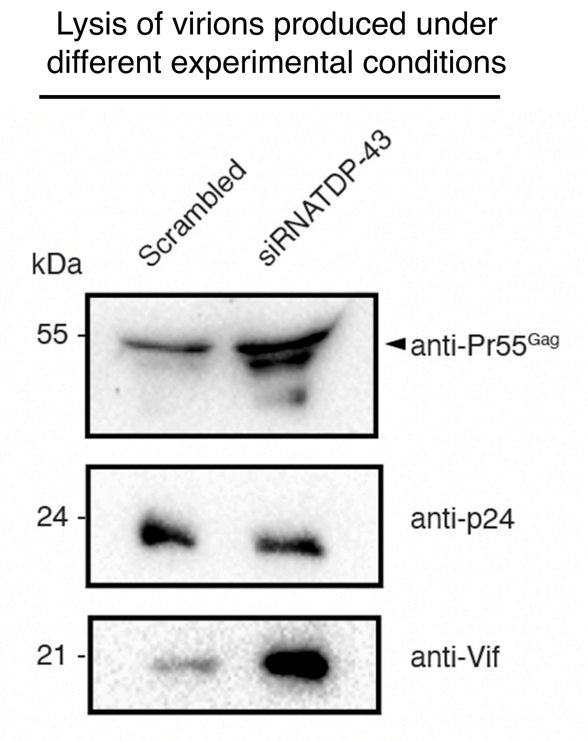

Replicate 2 as figure format

C

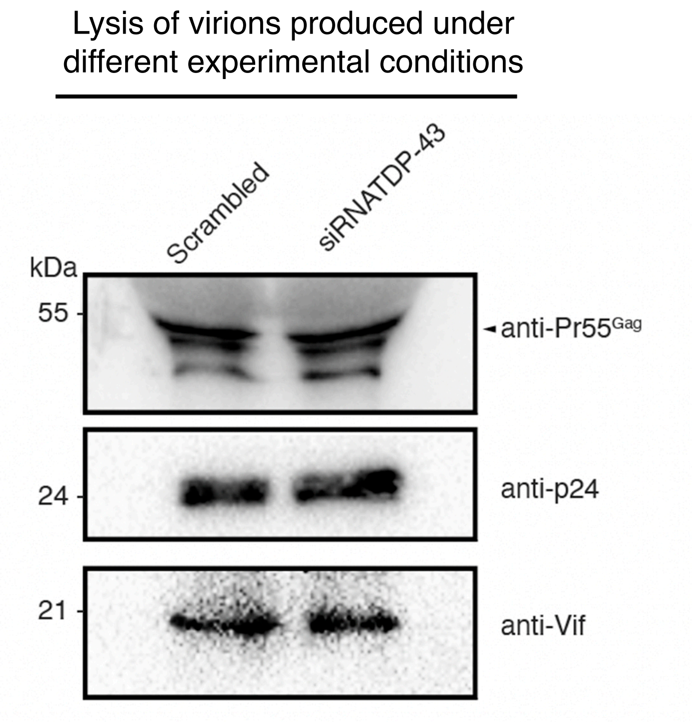

Replicate 3 as figure format

C

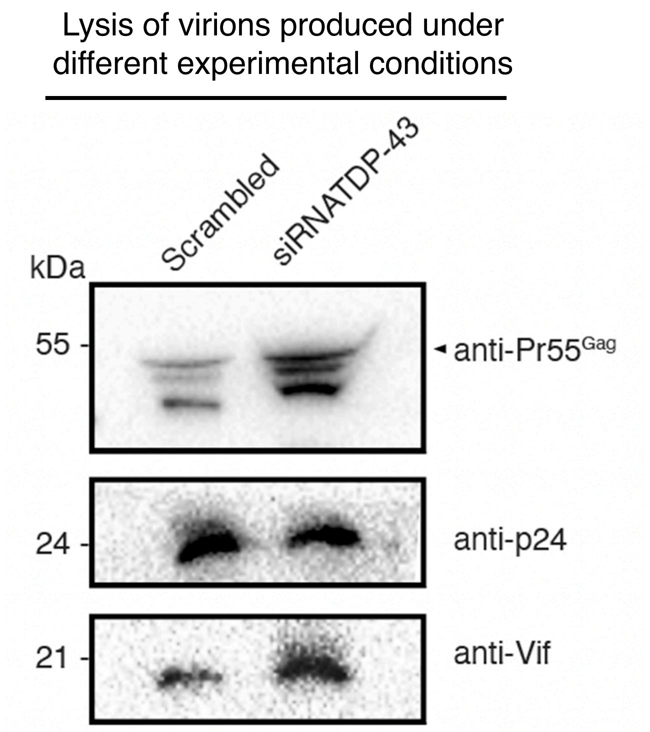

Figure S7C. Replicate 1 Pr55^{Gag} complete gel Western-blot associated with Figure 7C
Cabrera-Rodríguez, R., *et al.*

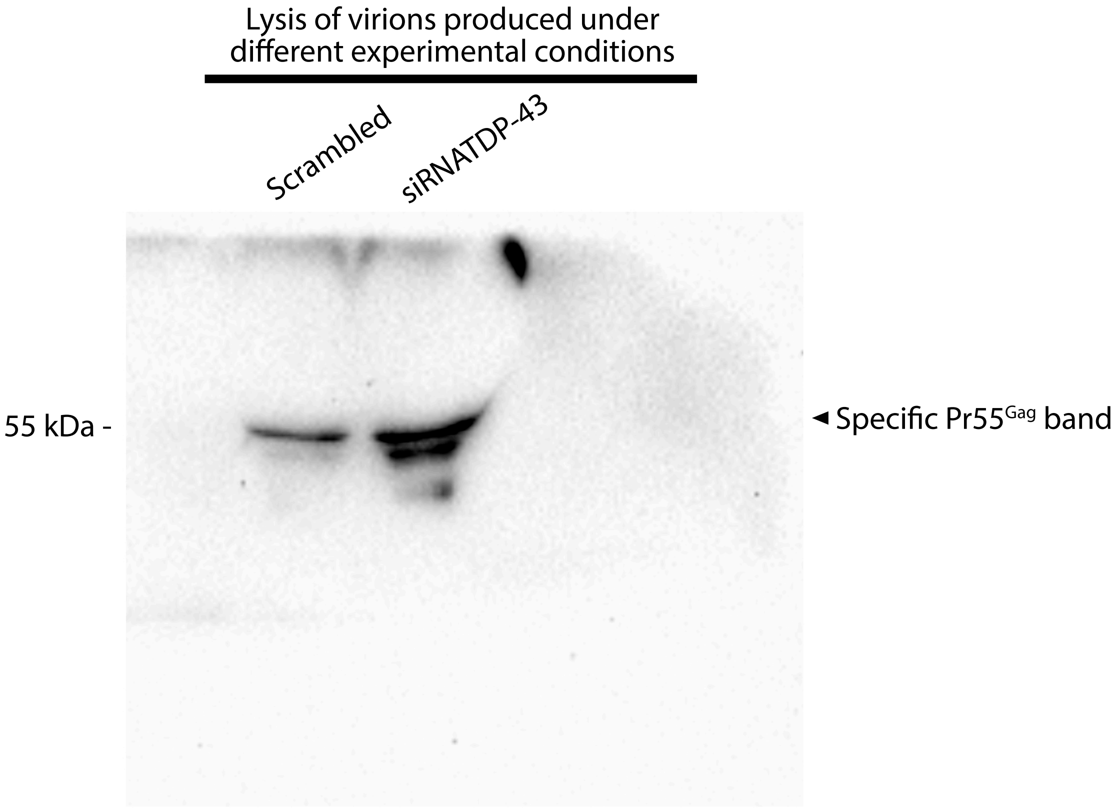

Figure S7C. Replicate 1 p24 complete gel Western-blot associated with Figure 7C

Cabrera-Rodríguez, R., *et al.*

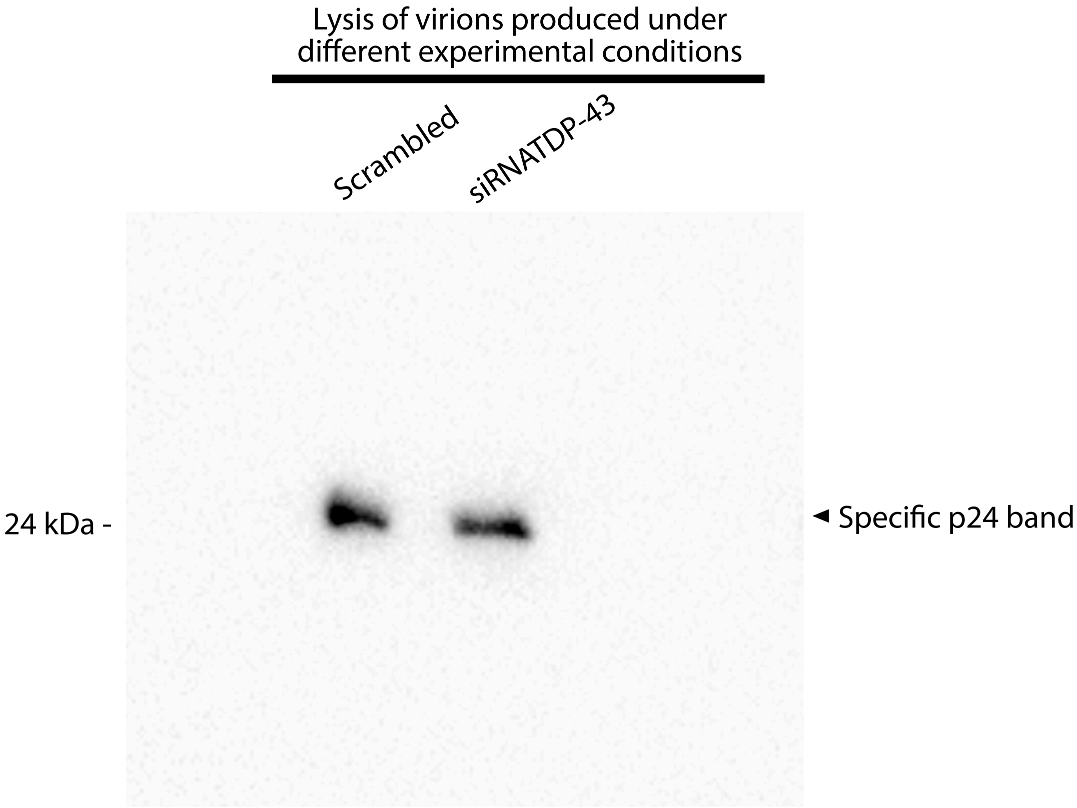

Figure S7C. Replicate 1 Vif complete gel Western-blot associated with Figure 7C
Cabrera-Rodríguez, R., *et al.*

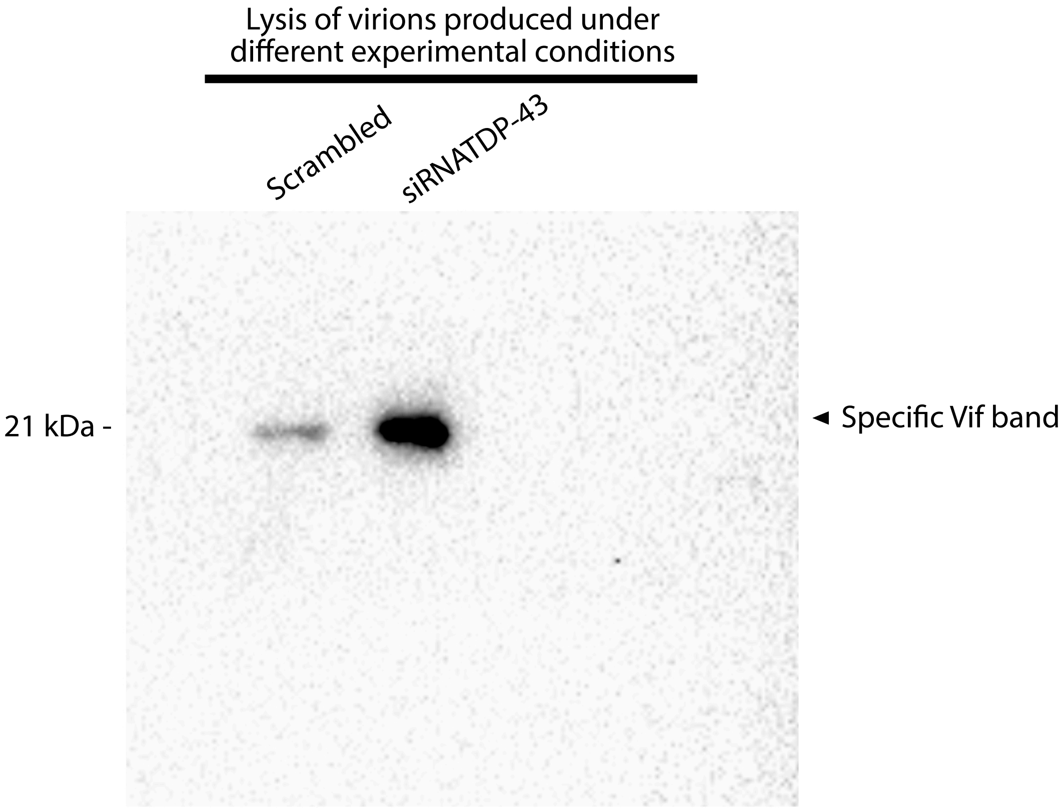

Figure S7C. Replicate 2 Pr55^{Gag} complete gel Western-blot associated with Figure 7C

Cabrera-Rodríguez, R., *et al.*

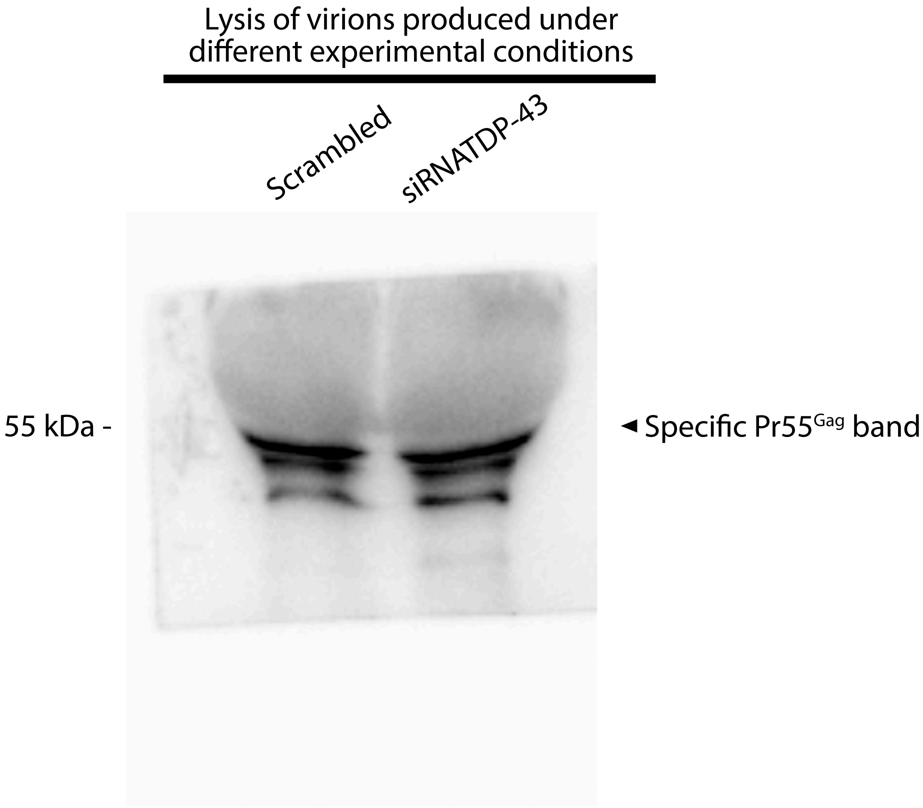

Figure S7C. Replicate 2 p24 complete gel

Western-blot associated with Figure 7C
Cabrera-Rodríguez, R., *et al.*

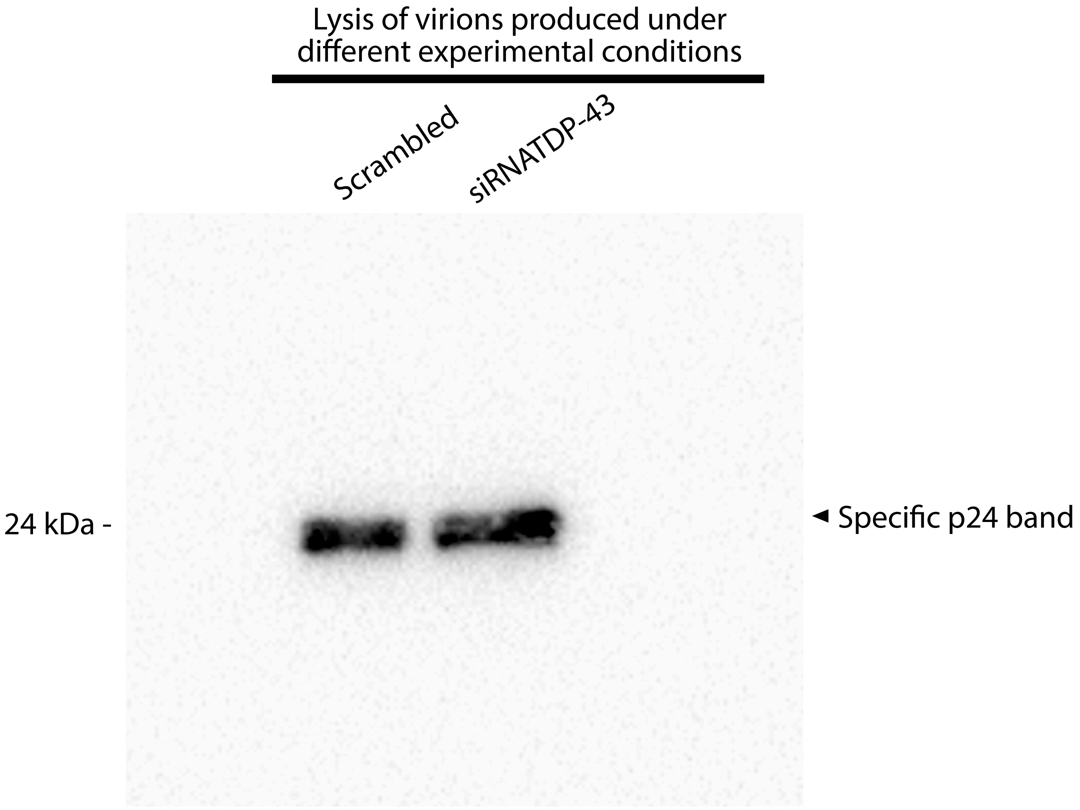

Figure S7C. Replicate 2 Vif complete gel   Western-blot associated with Figure 7C
Cabrera-Rodríguez, R., *et al.*

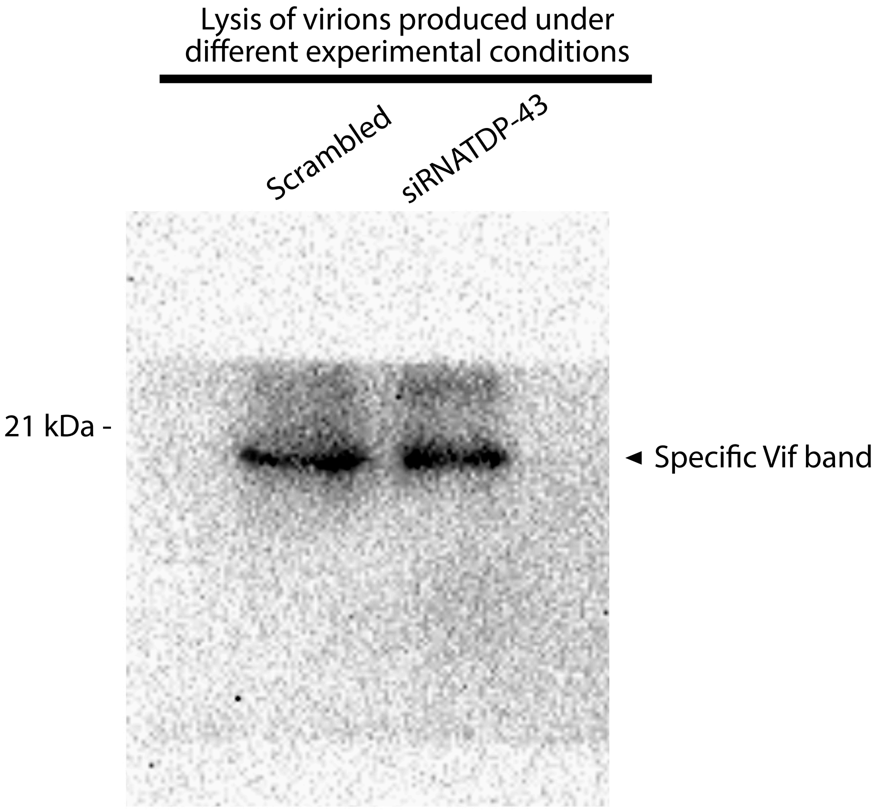

Figure S7C. Replicate 3 Pr55^{Gag} complete gel

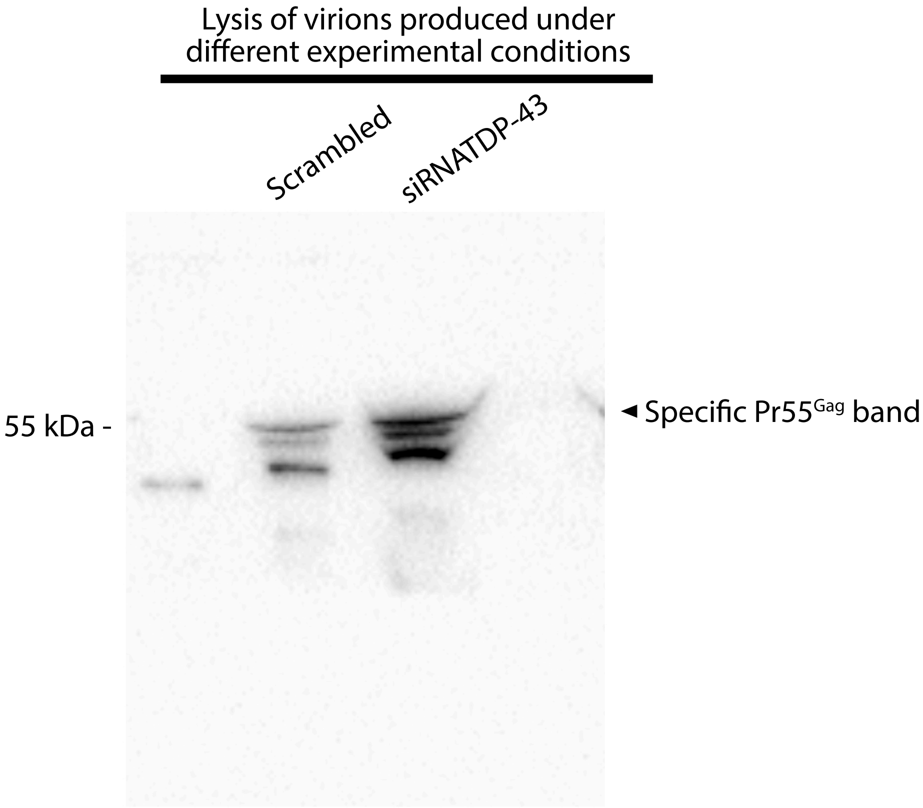

Figure S7C. Replicate 3 p24 complete gel Western-blot associated with Figure 7C
Cabrera-Rodríguez, R., *et al.*

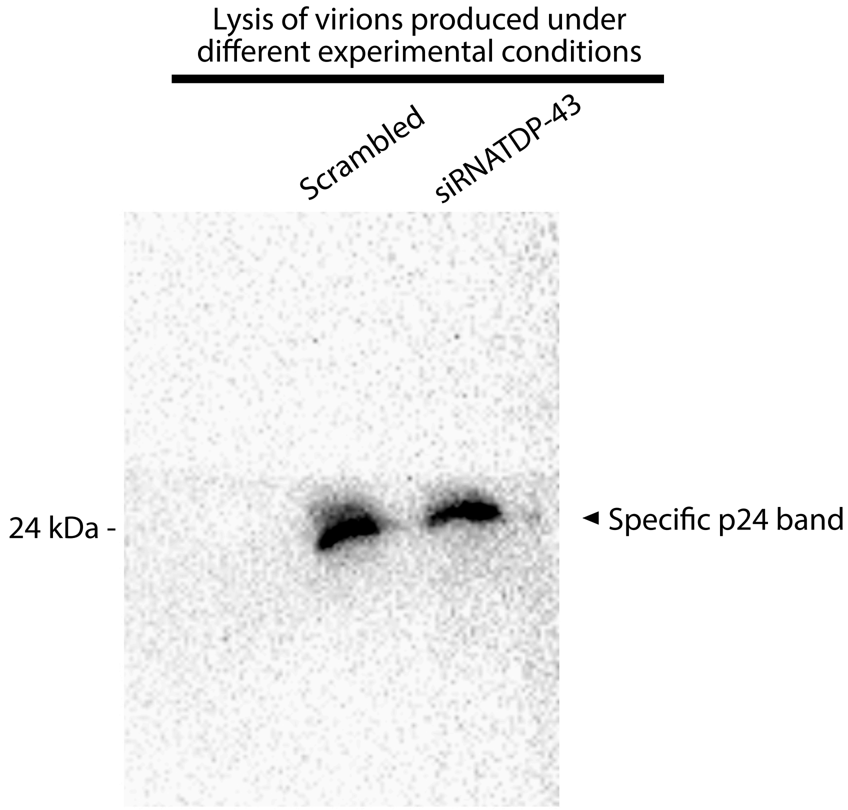

Figure S7C. Replicate 3 Vif complete gel

Western-blot associated with Figure 7C

Cabrera-Rodríguez, R., *et al.*

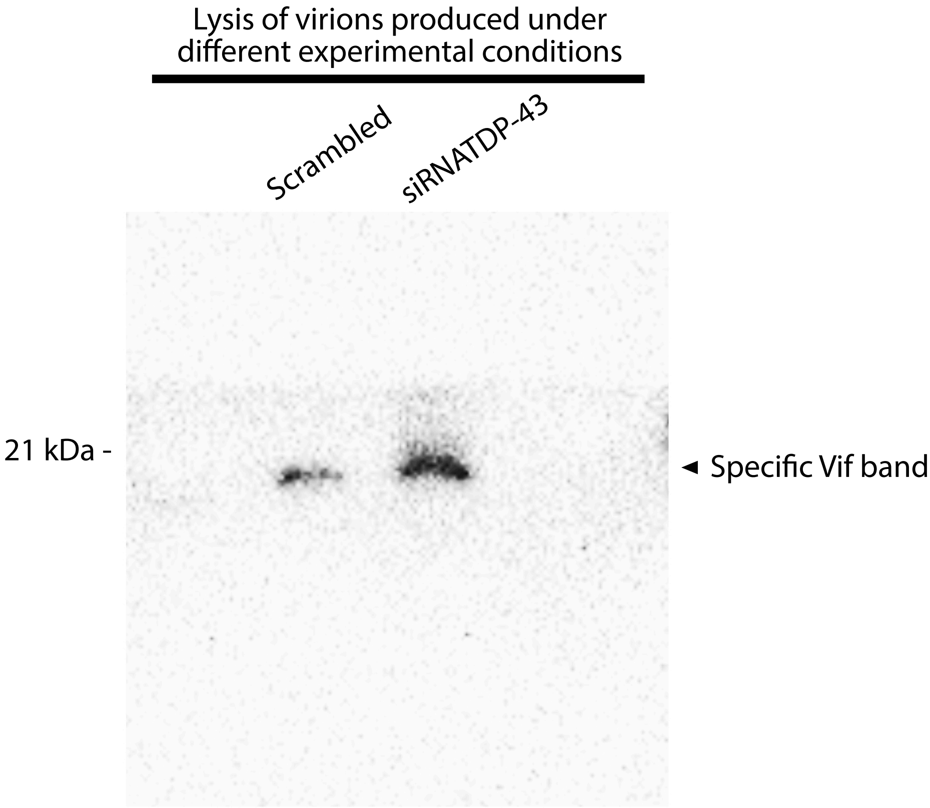

Supplement: Supplementary file 1 [file ijms-24-07658-s001.zip › ijms-2356206-supplementary.pdf]
